# Diversity of *Staphylococcus aureus* Isolates in European Wildlife

**DOI:** 10.1371/journal.pone.0168433

**Published:** 2016-12-16

**Authors:** Stefan Monecke, Dolores Gavier-Widén, Helmut Hotzel, Martin Peters, Sebastian Guenther, Alexandros Lazaris, Igor Loncaric, Elke Müller, Annett Reissig, Antje Ruppelt-Lorz, Anna C. Shore, Birgit Walter, David C. Coleman, Ralf Ehricht

**Affiliations:** 1 Institute for Medical Microbiology and Hygiene (IMMH), Technical University of Dresden, Dresden, Germany; 2 Alere Technologies GmbH, Jena, Germany; 3 InfectoGnostics Research Campus, Jena, Germany; 4 Department of Pathology and Wildlife Disease, National Veterinary Institute (SVA), Uppsala, Sweden; 5 Department of Biomedical Sciences and Veterinary Public Health, Swedish University of Agricultural Sciences (SLU), Uppsala, Sweden; 6 Friedrich-Loeffler-Institut, Federal Research Institute for Animal Health, Institute of Bacterial Infections and Zoonoses, Jena, Germany; 7 Chemisches und Veterinäruntersuchungsamt Westfalen, Standort Arnsberg, Arnsberg, Germany; 8 Institute of Microbiology and Epizootics, Veterinary Faculty, Freie Universität Berlin, Berlin, Germany; 9 Microbiology Research Unit, Dublin Dental University Hospital, University of Dublin, Trinity College Dublin, Dublin, Ireland; 10 Institute of Microbiology, University of Veterinary Medicine, Vienna, Austria; Rockefeller University, UNITED STATES

## Abstract

*Staphylococcus aureus* is a well-known colonizer and cause of infection among animals and it has been described from numerous domestic and wild animal species. The aim of the present study was to investigate the molecular epidemiology of *S*. *aureus* in a convenience sample of European wildlife and to review what previously has been observed in the subject field. 124 *S*. *aureus* isolates were collected from wildlife in Germany, Austria and Sweden; they were characterized by DNA microarray hybridization and, for isolates with novel hybridization patterns, by multilocus sequence typing (MLST). The isolates were assigned to 29 clonal complexes and singleton sequence types (CC1, CC5, CC6, CC7, CC8, CC9, CC12, CC15, CC22, CC25, CC30, CC49, CC59, CC88, CC97, CC130, CC133, CC398, ST425, CC599, CC692, CC707, ST890, CC1956, ST2425, CC2671, ST2691, CC2767 and ST2963), some of which (ST2425, ST2691, ST2963) were not described previously. Resistance rates in wildlife strains were rather low and *mecA*-MRSA isolates were rare (*n* = 6). *mecC*-MRSA (*n* = 8) were identified from a fox, a fallow deer, hares and hedgehogs. The common cattle-associated lineages CC479 and CC705 were not detected in wildlife in the present study while, in contrast, a third common cattle lineage, CC97, was found to be common among cervids. No *Staphylococcus argenteus* or *Staphylococcus schweitzeri*-like isolates were found. Systematic studies are required to monitor the possible transmission of human- and livestock-associated *S*. *aureus*/MRSA to wildlife and *vice versa* as well as the possible transmission, by unprotected contact to animals. The prevalence of *S*. *aureus*/MRSA in wildlife as well as its population structures in different wildlife host species warrants further investigation.

## Introduction

*Staphylococcus aureus* is a well-known colonizer and cause of infection among animals and it has been described from numerous domestic and wild animal species. Mammals known to harbor *S*. *aureus* include:

1. Ruminants: domestic cattle, buffalos [1], goats [2,3,4,5], ibexes (*Capra pyrenaica*, [6]), domestic sheep [7,8,9], bighorn sheep (*Ovis canadensis*, [10]), cervids (*Cervus/Capreolus* spec., [11,12,13])
2. Suids: domestic swine [14,15,16,17,18] and wild boars (*Sus scrofa*, [19])
3. Camelids: camels (*Camelus dromedarius*, [20,21])
4. Equids: horses [22,23,24,25]
5. Rhinoceroses: black rhinoceros (*Diceros bicornis*, [26])
6. Elephants: African elephants (*Loxodonta africana*, [27] and Asian elephants (*Elephas maximus*, [28])
7. Lagomorphs: domestic rabbits [29] and European brown hares (*Lepus europaeus*, [30])
8. Rodents: beavers (*Castor* spec., [31]), ground squirrels (*Citellus/Spermophilus lateralis*, [32]), red squirrels (*Sciurus vulgaris*, [33,34]), white mice [35], wood mice (*Apodemus sylvaticus*, [36]), chinchillas and guinea-pigs [37]
9. Insectivores: hedgehogs (*Erinaceus* spec., [38,39])
10. Bats: straw-coloured fruit bats (*Eidolon helvum*, [40]) and other bats [37]
11. Carnivores: domestic dogs and cats [4,25,41,42,43,44], foxes (*Vulpes* spec., [31]), minks (*Mustela vison*, [45]), raccoons (*Procyon lotor*, [31]), black bears (*Ursus americanus*, [46])
12. Pinnipeds: different species of seals (*Zalophus californianus*, *Phoca vitulina*, *Mirounga angustirostris*, *Halichoerus grypus*, [47,48]) and walruses (*Odobenus rosmarus*, [49])
13. Cetaceans: harbour porpoises (*Phocoena phocoena*, [48,50]), bottle-nosed dolphins (*Tursiops truncatu*s, [49,51]), orcas (*Orcinus orca*, [52]) and shortfinned pilot whales (*Globicephala macrorhynchus*, [53], GenBank JANQ)
14. Apes and monkeys such as chimpanzees (*Pan troglodytes*, [54]) and squirrel monkeys (*Saimiri* spec., [55])
15. Marsupials: wallabies (*Petrogale lateralis* and *xanthopus*, [56]) and White-Eared Opossum (*Didelphis albiventris*, [57]).

*S*. *aureus* is also known to occur in birds:

1. Fowl/poultry including domestic chickens [58,59,60,61,62], turkeys [61,63], Japanese quails (*Coturnix coturnix japonica*, [64,65])
2. Ciconiidae: white storks (*Ciconia ciconia*, [66])
3. Waterfowl such as mallards (*Anas platyrhynchos*, [67])
4. Diurnal raptors: griffon vultures (*Gyps fulvus*, [12]) and common buzzards (*Buteo buteo*, [68])
5. Otididae: houbara bustards (*Chlamydotis undulata*, [69,70])
6. Gulls (*Larus* spec., [71])
7. Psittacids: grey parrots (*Psittacus erithacus*, [72]), and other parrots [37]
8. Columbidae: domestic pigeons [73]
9. Perching birds (Passeriformes) including grey-crowned rosy-finches (*Leucosticte tephrocotis*, [74]), zebra finches (*Taeniopygia guttata*, [75]), chaffinches (*Fringilla coelebs*, [76]), rooks (*Corvus frugilegus*, [71,77]).

*S*. *aureus* has even been detected in the saliva of Komodo dragons (*Varanus komodoensis*, [78]) and, although it was probably acquired form a pet owner, in a turtle [37].

However, it is important to note that many reports of *S*. *aureus* in non-domestic species and exotic animals in the above list refer to captive individuals. In some cases, molecular typing confirmed that strains of human origin were transmitted to such animals [27,55,58]. Thus to date, the role of *S*. *aureus* as a possible pathogen, or colonizer, in wild populations of these animal species has not yet been studied systematically.

Two recent developments have highlighted a possible zoonotic component of *S*. *aureus* epidemiology. Firstly, in recent years livestock-associated, methicillin-resistant *S*. *aureus* strains (LA-MRSA) have emerged, especially in countries with high-density animal farming such as in the Netherlands and in Denmark. LA-MRSA strains belonging to multilocus sequence typing (MLST)-defined clonal complexes (CC) 9 [14,15,79,80,81], CC30 [82] and particularly CC398 [16,17,83,84,85] have spread in several countries and have in some cases also been implicated in human infections.

The second recent development relates to the discovery of a novel methicillin resistance gene, designated *mecC*, encoding an alternative penicillin-binding protein on a novel staphylococcal chromosome cassette *mec* (SCC*mec*) element designated SCC*mec* XI in MRSA [86,87,88]. To date, *mecC* has been identified in *S*. *aureus* isolates belonging to the animal-associated CCs 49, 130, 425, 599 and 1943; and *mecC* MRSA have been isolated from humans, mainly from different Western and Central European countries, but also from diverse animal species including cattle, sheep, hedgehogs, dogs, cats, a harbor seal (*Phoca vitulina*), a guinea pig, rabbits, rats, and a chaffinch (*Fringilla coelebs*) [39,76,86,87,89,90,91,92,93,94,95,96,97,98,99,100,101,102]. Recently, *mecC* was also identified in veterinary *Staphylococcus xylosus* [103] and *Staphylococcus stepanovicii* [104] isolates. This, as well as the observation of various *mecA* alleles in animal staphylococci [105,106] indicate that *mec* genes and, possibly, their precursors as well as other antimicrobial resistance genes might have a reservoir in animal strains of *Staphylococcus* species.

Due to the threat posed to animal and human health by the emergence of MRSA in animals as well as the paucity of published data on methicillin-susceptible *S*. *aureus* (MSSA) in wildlife, the aim of the present study was to investigate the molecular epidemiology of *S*. *aureus* in a convenience sample of European wildlife and to review what previously has been observed on this subject.

## Materials and Methods

### Isolates

A total of 2855 animals as well as a number of fecal samples (for details, see Table 1 and S1 Table) from three European countries, Austria, Germany and Sweden, were sampled. The collection encompassed 16 bird and 28 mammal species. From these 155 *S*. *aureus* isolates were recovered by culture, 124 of which were available for genotyping. That originated from nasal swabs (n = 54), skin, wound or abscess swabs (n = 18), swabs from pharynx, eyes or ears (n = 8), various post-mortem tissue samples (n = 29) or from fecal samples (n = 15). Details of the isolates recovered from the different wildlife species and their country of origin are shown in Table 1.

**Table 1:** Detection of *S*. *aureus* by country and host species.

**Order/family**	**Species**	**Animals examined (total)**	***S*. *aureus* detected (total)**	Animals examined (Germany)*	***S*. *aureus* identified (Germany)**	**Animals examined (Austria)**	***S*. *aureus* identified (Austria)**	**Animals examined (Sweden)**	***S*. *aureus* identified (Sweden)**
**Waterfowl (Anatidae)**	Mute swan, *Cygnus olor*	65	1	-	-	-	-	65	CC133-MSSA (n = 1)
	Greylag goose, *Anser anser*	1	0	1 (fecal sample, SX)	0	-	-	-	-
**Raptors (Accipitridae)**	Golden eagle, *Aquila chrysaetos*	71	3	-	-	-	-	71	CC97-MSSA and CC692-MSSA (from a single animal); *S*. *aureus*, not genotyped (n = 1) °°
	White-tailed eagle, *Haliaeetus albicilla*	4	1	3 (TH)	0	-	-	1	CC692-MSSA (n = 1)
	Red kite, *Milvus milvus*	1	1	1 (BB)	CC692-MSSA (n = 1)	-	-	-	-
**Gulls (Laridae)**	Herring Gull, *Larus argentatus*	1	0	1 (TH)	0	-	-	-	-
**Owls (Strigiformes)**	Tawny owl, *Strix aluco*	8	1	-	-	-	-	8	CC692-MSSA (n = 1)
	Barn owl, *Tyto alba*	1	0	1 (fecal sample, BB)	0	-	-	-	-
	Long-eared owl, *Asio otus*	3	0	3 (fecal sample and pellets, TH)	0	-	-	-	-
**Fowl (Phasianidae)**	Grey partridge, *Perdix perdix*	190	2	-	-	-	-	190	CC5-MSSA (n = 1); *S*. *aureus*, not genotyped (n = 1) °°
**Woodpeckers (Picidae)**	Green woodpecker, *Picus viridis*	2	2	-	-	-	-	2	CC692-MSSA (n = 1); *S*. *aureus*, not genotyped (n = 1) °°
**Perching birds (Passeriformes)**	Common magpie, *Pica pica*	29	3	-	-	-	-	29	CC692-MSSA (n = 3)
	Rook, *Corvus frugilegus*	102	9	-	-	102 (faecal samples)	CC15-MSSA (n = 1); CC88-MSSA (n = 3); ST1-MRSA-IV (n = 2)**; ST22-MRSA-IV[PVL+] (n = 3) **	-	-
	Carrion crow, *Corvus corone*	1	0	1 (SX)	0	-	-	-	-
	Great tit, *Parus major*	11	1	-	-	-	-	11	CC692-MSSA (n = 1)
	Blackbird, *Turdus merula*	1	0	1 (SX)	0	-	-	-	-
**Order/family**	**Species**	**Animals examined (total)**	***S*. *aureus* detected (total)**	Animals examined (Germany)*	***S*. *aureus* identified (Germany)**	**Animals examined (Austria)**	***S*. *aureus* identified (Austria)**	**Animals examined (Sweden)**	***S*. *aureus* identified (Sweden)**
**Insectivores**	Hedgehog, *Erinaceus europaeus*	199	6	5 (TH)	CC130-MRSA-XI (n = 1); CC599-MRSA-XI (n = 1)	-	-	194	CC130-MRSA-XI (n = 2)***; *S*. *aureus*, not genotyped (n = 2) °°
	Shrew, unidentified *Soricidae*	2	0	2 (SX, TH)	0	-	-	-	-
	Mole, *Talpa europaea*	4	0	2 (TH), 2 (NRW)	0	-	-	-	-
**Bats**	Parti-coloured bat, *Vespertilio murinus*	1	0	1 (TH)	0	-	-	-	-
**Rodents**	Bank vole, *Myodes glareolus*	N/A	4	N/A (fecal samples from an unknown number of animals)	CC49-MSSA (n = 2); ST890-MSSA (n = 1); ST1959-MSSA (n = 1)	-	-	-	-
	Brown rat, *Rattus norvegicus*	N/A	1	N/A (fecal samples from an unknown number of animals)	CC130-MSSA [*lukF-P83/lukM*+] (n = 1)	-	-	-	-
	European marmot, *Marmota marmota*	14	2	-	-	14	CC8-MSSA (n = 1); CC30-MSSA [*lukF-P83/lukM*+] (n = 1)	-	-
	Red squirrel, *Sciurus vulgaris*	1	0	1 (NRW)	0	-	-	-	-
**Carnivores**	Racoon, *Procyon lotor*	3	0	3 (BB, SX, NRW)	0	-	-	-	-
	European badger, *Meles meles*	28	4	3 (NRW), 3 (TH)	CC25-MSSA (n = 1); ST425-MSSA (n = 2)	-	-	22	ST425-MSSA (n = 1)
	Beech marten, *Martes foina*	6	0	6 (NRW)	0	-	-	-	-
	Mink, *M*.*lutreola/N*.*vison*	1	0	1 (SX)	0	-	-	-	-
	Least weasel, *Mustela nivalis*	1	0	1 (TH)	0	-	-	-	-
	Red fox, *Vulpes vulpes*	445	12	1 (SX), 1 (TH), 1 (BV), 92 (NRW)	CC1-MSSA (n = 1); CC22-MSSA (n = 1); ST425-MSSA (n = 1); CC130-MRSA-XI (n = 1)	29	CC7-MSSA (n = 1); CC8-MSSA (n = 1)	321	CC6-MSSA (n = 1); *S*. *aureus*, not genotyped (n = 5)
	Lynx, *Lynx lynx*	331	2	-	-	-	-	331	CC2767-MSSA (n = 1); *S*. *aureus*, not genotyped (n = 1)
	Wild cat, *Felis silvestris*	1	2	1	C49- and ST2963-MSSA from one animal	-	-	-	-
**Suids**	Wild boar, *Sus scrofa*	160	8	22 (NRW), 1 (TH), 1 (LS)	CC59-MSSA (n = 1); CC133-MSSA (n = 1); ST425-MSSA (n = 1)	46	CC9-MSSA (n = 1); CC97-MSSA (n = 2)	90	*S*. *aureus*, not genotyped (n = 2) °°
**Ruminants**	Moose, *Alces alces*	505	29	-	-	-	-	505	CC15-MSSA (n = 1); CC97-MSSA (n = 15); ST2691-MSSA (n = 2); *S*. *aureus*, not genotyped (n = 11) °°
	Roe deer, *Capreolus capreolus*	437	38	65 (NRW)	ST425-MSSA (n = 22); ST133-MSSA (n = 2); *S*. *aureus*, not genotyped (n = 7)	9	ST133-MSSA (n = 3)	363	CC97-MSSA (n = 4)
	Sika deer, *Cervus nippon*	4	2	4 (NRW)	ST3237-MSSA (n = 1)	—		-	-
	Red deer, *Cervus elaphus*	8	3	2 (NRW, TH)	ST425-MSSA (n = 1)	6	ST425-MSSA (n = 2)	-	-
	Fallow deer, *Dama dama*	10	3	10 (NRW)	CC1-MSSA (n = 2); CC130-MRSA-XI (n = 1)	-	-	-	-
	Reindeer, *Rangifer tarandus*	92	2	-	-	-	-	92	CC707-MSSA (n = 1); CC2767-MSSA (n = 1)
	Chamois, *Rupicapra rupicapra*	3	1	-	-	3	CC133-MSSA (n = 1)	-	-
	Mouflon, *Ovis orientalis*	31	3	2 (NRW)	CC1-MSSA (n = 2)	29	CC8-MSSA (n = 1)	-	-
**Lagomorphs**	European brown hare, *Lepus europaeus*	178	8	11 (NRW), 42 (SH), 1 (TH)	CC5-MSSA (n = 2); CC130-MRSA-XI (n = 2) °; CC398-MRSA-V/VT (n = 1)	-	-	124	ST2425-MSSA (n = 2); *S*. *aureus*, not genotyped (n = 1) °°
	Wild rabbit, *Oryctolagus cuniculus*	5	0	5 (NRW)	0	-	-	-	-
**Cetaceans**	Harbour porpoise, *Phocoena phocoena*	1	1	-	-	-	-	1	CC12-MSSA (n = 1)

* German Federal States are abbreviated as follows: Bavaria, BV; Brandenburg, BB; Lower Saxony, LS; North Rhine-Westphalia, NRW; Saxony, SX; Schleswig-Holstein, SH; Thuringia, TH

** Described in detail in [77]

*** Described in detail in [39]

° Sampled in 2012, described in detail in [104]

°° Not genotyped and not available for testing anymore. The number was provided in order to give a realistic impression of the prevalence of *S*. *aureus* in the respective host species.

Austrian isolates and isolates from the island of Pellworm (Schleswig Holstein; Germany) were obtained either during pathological examination of Austrian wildlife or recovered during studies that investigated the detection of MRSA in wildlife; methods used and geographic locations have been described previously [30,77].

The other German isolates, unless stated otherwise, were obtained by opportunistically swabbing wildlife that was found as road-kill or that had been shot by hunters. Rodent isolates were either obtained during screening of wildlife within the Network “Rodent associated pathogens” [107] or during a study on ESBL-producing *E*. *coli* in urban rats [108].

Swedish isolates were collected within the framework of the Swedish Wildlife Disease Surveillance Program and the Wildtech project (EU 7th Framework Program for Research and Technological Development, grant agreement no. 222633) in which wild animals were screened for various zoonotic pathogens.

Isolates were cultured on Columbia blood agar and Baird-Parker agar. Suspected *S*. *aureus* colonies were subcultured on Columbia blood agar and subsequently identified as *S*. *aureus* using standard procedures (catalase and coagulase/clumping factor production; VITEK II, bio-Mérieux, Nürtingen, Germany).

### Microarray procedures

All confirmed *S*. *aureus* isolates were characterized using the StaphyType DNA microarray or *S*. *aureus* Genotyping Kits 2.0 kit (Alere Technologies GmbH, Jena, Germany). This array simultaneously detects 333 *S*. *aureus* target sequences, including species markers, antimicrobial resistance and virulence-associated genes, and SCC*mec*-associated genes and typing markers allowing isolates to be assigned to MLST sequence types (STs) and/or CCs, and SCC*mec* types. The latter kit also detects SCC*mec* XI/*mecC* [39] and it was applied to all isolates tested after 2013, to all CC130 and CC599 isolates as well as to select additional isolates (see S1 Table). Protocols and procedures as well as primer and probe sequences have been previously described in detail [39,109,110]. In brief, *S*. *aureus* isolates were stored frozen in commercially available cryotubes (various brands; at -40 or -80°C), and grown on Columbia blood agar and incubated overnight at 37°C. Bacterial cells were enzymatically lysed prior to DNA preparation using commercially available spin columns (Qiagen, Hilden, Germany). Purified DNA samples were used as templates in a linear primer elongation using one primer per target. All targets were amplified simultaneously, and within this step, biotin-16-dUTP was incorporated into the resulting single-stranded amplicons. Amplicons were stringently hybridized to the microarray followed by washing and an addition of a horseradish-peroxidase-streptavidin conjugate. After further incubation and washing, hybridizations were visualized by adding a locally precipitating dye. An image of the microarray was taken and analyzed using a designated reader, software and database.

### PCRs for characterizing SCC*mec* XI

SCC*mec* XI was further characterized by PCR in three isolates (two CC130 isolates [39] and one ST599 isolate). PCR amplification targeted fragments across the entire element using overlapping primers [87] and the Expand long-template PCR System (Roche Diagnostics GmbH, Lewes, East Sussex, United Kingdom). Sizes of the resulting amplicons were compared to those of the *mecC*-positive reference strain M10/0061 [87].

### MLST and *spa*

*S*. *aureus* MLST was performed for isolates which initially could not be identified based on their array hybridization profiles as well as for some isolates that have been discussed in separate studies [30; 39; 77; 107;108]. MLST was performed according to standard protocol [111] using the tools and database provided on the *S*. *aureus* MLST website (http://saureus.mlst.net/). Novel profiles were submitted to the MLST database.

*Spa* typing was performed according to previously published protocols [112] and sequences were analyzed using RIDOM nomenclature (http://www.spaserver.ridom.de/).

MLST and *spa* data are shown in S1 Table.

### Antimicrobial susceptibility tests

Susceptibility tests were performed on selected isolates (*mecC*- or *cfr/fexA*-positives). This was done by automated agar dilution tests using the VITEK-2 device (bioMérieux) with AST-P608 test cards according to manufacturer’s instructions. Chloramphenicol was tested by disk diffusion using 30 μg disks (BD, Heidelberg, Germany) and according to the methodology and criteria of the Clinical and Laboratory Standards Institute (CLSI) [113].

## Results

### *S*. *aureus* prevalence and strains in the different host species

124 *S*. *aureus* isolates were characterized and they were assigned to 29 CCs or singleton STs. The key characteristics of each CC or ST are described below.

Table 1 summarizes the number of animals examined, the number that yielded *S*. *aureus*, and which STs or CCs were detected in the different wildlife species. Scientific/Latin names of the animal species discussed are also provided in this table.

Key markers that are characteristic for CCs, such as *agr* group or capsule type affiliations, or presence of the enterotoxin gene cluster *egc* are listed in Table 2.

**Table 2:** CC/ST characteristics of *S*. *aureus* isolates investigated.

CC/ST	*agr* group	*seh*	*egc* locus	ORF CM14	*lukD*	*lukE*	capsule type	*cna*	*sasG*	Number of isolates	Distribution by country	Distribution by host species
**CC1**	**III**	**POS**	NEG	NEG	**POS**	**POS**	**8**	**POS**	**POS**	7	AT, D	Fallow deer, mouflon, red fox, rook
**CC5**	**II**	NEG	**POS**	NEG	**POS**	**POS**	**5**	NEG	**POS**	3	D, SW	Brown hare, grey partridge
**CC6**	**I**	NEG	NEG	NEG	**POS**	**POS**	**8**	**POS**	**POS**	1	SW	Red fox
**CC7**	**I**	NEG	NEG	NEG	**POS**	**POS**	**8**	NEG	NEG	1	AT	Red fox
**CC8**	**I**	NEG	NEG	NEG	**POS**	**POS**	**5**	NEG	**POS**	3	AT	Marmot, mouflon, red fox
**CC9**	**II**	NEG	**POS**	NEG	NEG	NEG	**5**	NEG	NEG	1	AT	Wild boar
**CC12**	**II**	NEG	NEG	**POS**	**POS**	**POS**	**8**	**POS**	NEG	1	SW	Harbor porpoise
**CC15**	**II**	NEG	NEG	NEG	**POS**	**POS**	**8**	NEG	**POS**	2	AT, SW	Rook, Moose
**CC22**	**I**	NEG	**POS**	NEG	NEG	NEG	**5**	**POS**	**POS**	4	AT, D	Rook, red fox
**CC25**	**I**	NEG	**POS**	NEG	**POS**	**POS**	**5**	NEG	NEG	1	D	Badger
**CC30**	**III**	NEG	**POS**	NEG	NEG	NEG	**8**	**POS**	NEG	2	AT	Marmot, red deer
**CC49**	**II**	NEG	NEG	NEG	**POS**	**POS**	**5**	NEG	**POS**	3	D	Vole, wild cat
**CC59**	**I**	NEG	NEG	NEG	NEG	NEG	**8**	NEG	**POS**	1	D	Wild boar
**CC88**	**III**	NEG	NEG	NEG	**POS**	**POS**	**8**	NEG	**POS**	3	AT	Rook
**CC97**	**I**	NEG	NEG	NEG	**POS**	**POS**	**5**	NEG	**POS**	24	AT, D, SW	Golden eagle, wild boar, moose, roe deer
**CC130**	**III**	NEG	NEG	NEG	**POS**	AMB	**8**	NEG	NEG	8	D, SW	Brown rat, hedgehog, brown hare, red fox, fallow deer
**CC133**	**I**	NEG	NEG	NEG	**POS**	AMB	**8**	NEG	NEG	8	AT, D, SW	Mute swan, wild boar, roe deer, chamois
**CC398**	**I**	NEG	NEG	NEG	NEG	NEG	**5**	**POS**	NEG	1	D	Brown hare
**ST425**	**II**	NEG	NEG	NEG	**POS**	NEG	**5**	NEG	Variable	29	AT, D, SW	Badger, red fox, wild boar, red deer, roe deer
**CC599**	**I**	NEG	NEG	NEG	**POS**	**POS**	**5**	NEG	NEG	1	D	Hedgehog
**CC692**	**I**	NEG	NEG	NEG	**POS**	**POS**	**5**	NEG	NEG	9	D, SW	Red kite, golden and white-tailed eagle, tawny owl, green woodpecker, common magpie, great tit
**CC707**	**III**	NEG	NEG	**POS**	AMB	**POS**	**5**	**POS**	NEG	1	SW	Reindeer
**ST890**	**IV**	NEG	NEG	NEG	NEG	**POS**	**8**	**POS**	NEG	1	D	Bank vole
**CC1956**	**IV**	NEG	NEG	NEG	**POS**	**POS**	**5**	**POS**	NEG	1	D	Bank vole
**ST2425**	**IV**	NEG	NEG	**POS**	**POS**	**POS**	**8**	**POS**	NEG	2	SW	Brown Hare
**ST2691**	**II**	NEG	NEG	NEG	**POS**	AMB	**8**	NEG	NEG	2	SW	Moose
**CC2767**	**I**	NEG	NEG	NEG	Variable	NEG	**8**	**POS**	NEG	2	SW	Lynx, reindeer
**ST2963**	**IV**	NEG	NEG	NEG	**POS**	NEG	**8**	**POS**	NEG	1	D	Wild cat
**ST3237**	**IV**	NEG	NEG	NEG	**POS**	AMB	**8**	NEG	NEG	1	D	Sika deer

Abbreviations are AMB, ambiguous; AT, Austria; D, Germany; SW, Sweden. For Latin names of host species, see Table 1.

Table 3 shows the overall prevalence of resistance genes among the isolates.

**Table 3:** Prevalence of resistance genes in 124 *S*. *aureus* isolates from wildlife.

Gene	Number of isolates	Percentage
***mecA***	**6**	**4.8**
***mecC***	**8**	**6.5**
***blaZ* from SCC*mec* XI**	**8**	**6.5**
***blaZ***	**24**	**19.4**
***erm*(A)**	**1**	**0.8**
***erm*(B)**	**1**	**0.8**
***erm*(C)**	**1**	**0.8**
***lnu*(A)**	0	0.0
***msr*(A)**	0	0.0
***mefA***	0	0.0
***mph*(C)**	0	0.0
***vat-/vga* genes**	0	0.0
***aacA-aphD***	**3**	**2.4**
***aadD***	**3**	**2.4**
***aphA3***	**2**	**1.6**
***sat***	**2**	**1.6**
***dfrS1***	**3**	**2.4**
***fusB***	0	0.0
***fusC***	0	0.0
***mupA***	0	0.0
***tet*(K)**	**6**	**4.8**
***tet*(M)**	**1**	**0.8**
***cat***	**3**	**2.4**
***cfr***	**1**	**0.8**
***fexA***	**1**	**0.8**
***qacA***	0	0.0
***qacC***	0	0.0
***vanA***	0	0.0

### Clonal complex 1

Five CC1-MSSA isolates were identified, two from fallow deer, two from mouflon, and one from a red fox. They did not harbor *mecA*, *mecC* or any other resistance genes. Virulence associated markers included *seh* (but no other enterotoxin genes). None of the isolates had evidence for the presence of lysogenic beta-hemolysin converting phages as all isolates were *hlb*-positive and were negative for *sea*, *see*, *sep*, *chp*, *sak* and *scn*.

Two CC1-MRSA-IV isolates were identified from Austrian rooks [77] that carried *blaZ*, *tetK*, *apha3* and *sat* (kana-/neomycin and streptothricin resistance).

CC1 is a common lineage among humans including both MSSA and community-acquired (CA-) MRSA; and CC1 is also frequently found in cattle [114,115]. A strain that closely resembled the MRSA from rooks was frequently found among humans in Romania [116].

### Clonal complex 5

Three CC5-MSSA were identified, one from a Swedish grey partridge and two from hares from the island of Pellworm. The two hare isolates carried *sea* and the immune evasion complex (IEC) genes *scn*, *chp* and *sak*, whereas the partridge isolate lacked these genes. Genes encoding other enterotoxins or PVL were absent. None of the resistance genes covered was detected. CC5 is a common and widespread lineage in humans. It has recently [58] spread into domestic poultry (see Introduction) where it is now be commonly encountered [61]. A further dissemination to wild fowl such as partridges appears plausible.

### Clonal complex 6

A single CC6-MSSA isolate was identified in a Swedish red fox. The hybridization pattern of the isolate was identical to the predicted hybridization pattern for clinical CC6-MSSA isolates 394_SAUR (GenBank JVIV) and C9 (GenBank LDVH). It harbored the beta-lactamase operon as well as the IEC genes *sea*, *sak* and *scn*.CC6 has been recovered occasionally from humans including butchers [117] and it has been implicated in episodes of food-poisoning [118]. It was previously identified in camels from Dubai, where it carried a different *sea* allele [21], as well as in non-human primates and various domestic animals [4,119].

### Clonal complex 7

A single CC7-MSSA isolate was recovered from an Austrian red fox. It carried the IEC genes *sea*-N315, *scn* and *sak* genes. The isolate was indistinguishable from previously described human CC7-MSSA isolates [120,121].

### Clonal complex 8

Several CC8-MSSA isolates were identified in Austrian animals including a red fox, a marmot and a mouflon. The fox isolate lacked antimicrobial resistance genes but harbored the *sak* and *scn* genes. The other two isolates were positive for *blaZ*, enterotoxins *sed*, *sej*, *ser*, as well as for the IEC genes *sea*, *sak* and *scn*. CC8 is a common lineage among humans [120,121] and several MRSA clones [109] originated from that lineage. It is not typically considered a zoonotic lineage although CC8-MRSA have been found in horses [24], wildlife [122] and whales [53].

### Clonal complex 9

A single CC9-MSSA isolate was recovered from an Austrian wild boar. The isolate was positive for the *egc* locus but lacked other relevant toxin genes. It was positive for the tetracycline resistance gene *tet*(K) but other antimicrobial resistance genes were absent. CC9-MSSA has been found in wild chimpanzees in Ivory Coast [54] but we are not aware of any reports from European wildlife. However, CC9 livestock-associated MRSA are frequently associated with domestic pigs (see Introduction) so that a presence of CC9-MSSA in wild boar is perhaps not surprising.

### Clonal complex 12

A single CC12-MSSA isolate was recovered from a Swedish harbor porpoise. The beta-lactamase operon (*blaZ/R/I*) was present, enterotoxin genes and IEC genes were absent, although they are frequently but not always found in human isolates of that lineage [120,121]. Apart from humans, CC12 has previously been found in chickens [60] and swine [123]. *S*. *aureus* has previously been reported in porpoises (see Introduction), but isolates belonged to other sequence types some of which were unique (CC1762), while others are known to be associated with humans or terrestrial mammals [48].

### Clonal complex 15

Two isolates of CC15-MSSA were identified in an Austrian rook and a Swedish moose. They were essentially identical to previously characterized human isolates [120,121]. As in these human isolates, the *sak* gene was absent although, *scn* and *chp* were detected. Furthermore, the isolates lacked enterotoxin and PVL genes or *lukM/lukF*-P83. The beta-lactamase gene was present. CC15 is a widespread and common human lineage [120,121]. It has occasionally been reported from companion animals [124], primates [119] and mice [36].

### Clonal complex 22

A single CC22-MSSA isolate was found in a fox from Germany. It harbored *blaZ* as well as the IEC genes *chp*, *scn* and *sak*. Essentially it was indistinguishable from previously described human isolates. Three CC22-MRSA isolates from Austrian rooks have previously been described [77]. In short, they carried PVL genes as well as *blaZ*, *aacA-aphD*, *aadD* and *dfrS1*. CC22 is a common “human lineage” but has also been found in companion animals with close contacts to humans [125,126] and in wild roe deer [11]. CC22 MRSA are extremely common in Western Europe, but the rook isolates were not identical to the ubiquitous Barnim/UK-EMRSA-15 epidemic strain differing in SCC*mec* subtype (IVc rather than IVh; [77]) and presence of PVL genes. They were identical to a strain observed in several Middle Eastern countries and people with Middle Eastern travel history [109,127,128,129,130].

### Clonal complex 25

A single isolate of CC25-MSSA was identified from a German badger. It carried the *blaZ* operon, the enterotoxin B gene, *edinB*, *etD* and IEC genes *sak*, *scn* and *chp*. It was essentially identical to human isolates as previously characterized [120,121].

### Clonal complex 30

One isolate of CC30-MSSA was found in an Austrian marmot. It carried the leukocidin genes *lukM/lukF-P83*. Surprisingly, it was positive for the multidrug resistance gene *cfr* that confers the PhLOPSa resistance phenotype, *i*.*e*., resistance to phenicols, lincosamides, oxazolidiones, pleuromutulin and streptogramin A compounds, and the phenicol resistance gene *fexA*. It was resistant to the oxazolidinone agent linezolid (MIC > 8 μg/mL) as well as to clindamycin and chloramphenicol. Another isolate originated from Austrian deer. It only harbored *blaZ*.

CC30 is a common human lineage [120,121,131,132] from which several MRSA [109] and toxigenic strains evolved [133,134]. CC30 has previously been found in animals, including camels [21] and pigs [82]. It also has been detected in Spanish white storks [66] and a Portuguese buzzard [68].

### Clonal complex 49

CC49-MSSA isolates were recovered from two fecal samples of voles from Bavaria and from a road-killed wildcat from North Rhine-Westphalia. They carried leukocidin genes *lukM/lukF*-P83, but lacked enterotoxin genes. These animal isolates differed from previously described human isolates [120,132,135] and one genome sequence (Tager 104, GenBank AVBR [136]) in the presence of *lukM/lukF*-P83 and the absence of IEC genes associated with beta-hemolysin converting phages. They lacked any antimicrobial resistance or SCC*mec* associated markers.

This CC has been found in humans [120,132,135] but appears to be rare. Previously, CC49-MSSA have also been observed to cause an outbreak among red squirrels on the Isle of Wight and the island of Jersey that died from exudative, ulcerative dermatitis and superficial staphylococcal pyoderma [34]. This squirrel strain was found to be *lukM*-positive [33] like the isolates described in the present study, but unlike the human isolates mentioned above. This might indicate host specific adaptions related to the presence of a phage. CC49-MRSA-V has been observed in Swiss livestock [137] and CC49-MRSA-XI have been sequenced (NCBI BioSamples SAMEA1463364, SAMEA1463346).

### Clonal complex 59

One CC59-MSSA was isolated from a shot Thuringian wild boar. The isolate carried *erm*(C) and *blaZ* but lacked enterotoxin and PVL genes or *lukF-P83/lukM*. It carried IEC genes *chp* and *scn* but *sak* was not detected. CC59 is widespread in humans and several MRSA strains originate from this lineage [109,138,139,140]. It has been detected in workers in contact with livestock or animal carcasses [141,142] and retail food [143] as well as in pets [144] but we are not aware of reports to date from wildlife.

### Clonal complex 88

Three isolates of CC88-MSSA were identified from Austrian rooks. They lacked relevant toxin genes. All three harbored *blaZ* and the *cat* gene (encoding chloramphenicol resistance). CC88 is a widespread human lineage from which MRSA strains evolved that are common in Australia [145] and Sub-Saharan Africa [119,146,147,148,149]. It has been described from laboratory mice [35], from retail food [143] and from pigs [150].

### Clonal complex 97

Twenty isolates of CC97-MSSA from Swedish wildlife were identified. One originated from a golden eagle, four from roe deer and the rest were from moose. Two isolates were found in roe deer from Germany, one suffering from botfly infestation, pyogranulomatous dermatitis and pneumonia, the other one from necrotic/gangrenous spondylodiscitis and pericarpitis. Two additional isolates originated from Austrian wild boar. All these isolates appeared identical to the sequenced reference strain Newbould 305 (GenBank AKYW, a bovine strain from Canada). They did not carry relevant resistance or toxin genes. The two Austrian isolates carried an isolated recombinase homologue *ccrAA* and reacted with new experimental probes (*opp3B*; ACSQ01000050.1 [4287:4313] and *opp3C*-C427 ACSQ01000050.1 [5150:5174]; [129]) indicating the presence of a type 3 ACME element.

CC97 has been reported from roe deer before, from the Italian Alps [11]. Besides, CC97 is frequently detected in humans [120], pigs [123,151], sheep [152] as well as in cattle [2,152,153], with the latter being phylogenetically related to deer species. Given the phylogenetic relationship between cervids and bovids, it can be postulated that this lineage is transmitted between both, or that it co-evolved with them. The eagle mentioned above might have been infected when scavenging.

### Clonal complex 130

One CC130-MSSA was found in a fecal sample of a brown rat having the MLST profile ST2024 and the *spa* type t8403. Its array hybridization profile was essentially identical to predicted patterns for the sequenced strains O11 (GenBank AEUQ) and O46 (GenBank AEUR). It carried *lukF-P83/lukM* as well as *edinB* and *etD*2 [39]. It differed from O11 and O46 in the presence of cadmium resistance genes *cadD/cadX* (*cadD*; BX571858.1, pSAS [8203:8231] and *cadX;* BX571858.1, pSAS [8797:8827]).

CC130-MRSA-XI was identified in two Swedish hedgehogs; details have been reported separately [39]. Among the German samples, this strain was detected once each in a hedgehog, a red fox and a fallow deer as well as in two hares [104]. The isolates harbored *mecC*, the SCC*mec* XI-associated beta-lactamase gene and an arsenic resistance operon (*arsB*; FR823292.1, [29339:29366:r] and *arsC*; FR823292.1, [28080:28103:r]; [129]); they lacked *lukF-P83/lukM* but carried *edinB* and *etD2* [39].

CC130-MSSA appears to be a common lineage in sheep and a major cause of infections in these animals (see [154], where it is named CC700), but we are not aware of observations in humans. Previously, CC130-MRSA-XI have been found in humans as well as in a wider variety of wild and domestic animals (see references quoted in the Introduction). Although there are no systematic studies yet available for most parts of the world, they seem largely to be geographically restricted to Western Europe. CC130-MRSA-XI in humans is rather rare. In Germany, about one out of 1,000 MRSA isolates belong to that strain [91]. In Denmark, it accounts for 2.7% of human MRSA infections. A zoonotic link of CC130-MRSA-XI appears to be possible [94] and domestic animals such as small ruminants might serve as hosts.

### Clonal complex 133

This strain was found in a wild boar, roe deer, chamois and a mute swan. The isolates did not carry any resistance markers. Isolates also lacked *tst1*, enterotoxin genes *sec/sel* and *lukF-P83/lukM* although these genes are known to frequently occur in CC133 (see genome sequence of ED133, GenBank CP001996 and [2,155]). CC133-MSSA were previously detected in wild boar [19] and in Griffon vultures [12]. This strain was also frequently observed in small domestic ruminants, *i*.*e*., goats and sheep [2,3,154], but also in domestic pigs [123], cats, dogs, [156], cattle [155] and donkeys [157]. It could have been transmitted from wild to domestic ruminants, or *vice versa*, and the few reported human cases [116] could be related to contact to animals or animal products.

### Clonal complex 398

One CC398-MRSA-V isolate was detected in a hare. It carried *blaZ*, *erm*(B), *tet*(K) and *tet*(M) but lacked relevant enterotoxin genes, PVL and *lukF-P83/lukM*. CC398 is a livestock-associated lineage with MSSA frequently been detected in poultry. CC398-MRSA-V was first discovered in the Netherlands in 2006 [16,17,83,84,85] and subsequently spread across several livestock species and humans in most of Western Europe. It was also occasionally detected in wildlife [13].

### Sequence type 425

ST425 was (with 29 isolates) the most common lineage observed in this study. This included several isolates from roe deer, three isolates from red deer, one isolate each from wild boar and red fox as well as three isolates from badgers. For one German badger, generalized ulcerations were recorded.

Two of three badger isolates (including the one with the ulcerations) as well as one roe deer isolate carried the enterotoxin E gene *see*. These isolates had a truncated *hlb* gene although other IEC genes were absent. The other isolates lacked any genes from beta-hemolysin converting phages, *see* or *sea*, and they had an un-truncated *hlb*. One roe deer isolate was positive for the beta-lactamase operon, but other resistance markers were not found. No *mecC*-positive isolate was identified.

ST425 is a lineage that has been found in wild and domestic animals. Previously it was noted in wild boar from Germany [19] and red deer from Spain [12] as well as in humans. Patterns of transmission might be assumed in which domestic and wild ruminants might get infected by ingesting carnivore excretions, or in which carnivores might be infected by scavenging or by ingesting, *i*.*e*., wild berries (a common behavior in foxes and badgers) that might be contaminated with ruminant feces. The recent emergence of ST425-MRSA-XI warrants further monitoring. Such strains were reported from cattle in the United Kingdom [86] as well as from wildlife (fallow deer, wild boar) and environmental samples from Spain [158].

### Clonal complex 599

This strain was found once, in a road-killed hedgehog from Thuringia. It carried SCC*mec* XI including *mecC*, *blaZ* and arsenic resistance determinants. All but one of the previously described PCRs [87] for the characterization of SCC*mec* XI yielded products of the expected sizes. The PCR with primers arsCM10/0061-F1: 5'-GACCACTCTTTACCTGCT-3'/tnp1S2 R1: 5'-AGATCATGGAAAACCGATCA-3', however, failed to amplify. These findings suggest that i) the SCC*mec* XI element was identical to those of CC130-MRSA-XI isolates [39,87] and ii) that the adjacent genomic (non-SCC) region was different. Phenotypically, the isolate was penicillin-, oxacillin- and cefoxitin-resistant.

Additionally, *cadD/cadX* cadmium resistance genes were detected (*cadD*; BX571858.1, pSAS [8203:8231] and *cadX;* BX571858.1, pSAS [8797:8827]). Regarding virulence markers, the isolate tested positive for *tst1*, *sec*, *sel*, *“seU2”* (an enterotoxin gene ALWH01000034.1, positions 21908 to 22660*)*, the gamma-hemolysin locus and *lukD/E* while IEC genes, *cna* and *sasG* were absent. This isolate was virtually identical to a human isolate from The Netherlands [96].

CC599-MRSA-XI have previously been detected in cattle [159] and in a domestic cat [160]. To the best of our knowledge, there are no descriptions of CC599-MRSA-XI in wildlife, and to date no reports of CC599-MSSA at all.

### Clonal complex 692

Swedish isolates of this strain included three from common magpies as well as one each from a great tit, a green woodpecker, a golden and a white-tailed eagle as well as from a tawny owl. Additionally, one isolate from bullous eruption at the knee of a red kite from Germany was tested. Enterotoxin genes were not detected, and resistance genes were absent.

This lineage has, according to the MLST database, previously been observed in a domestic pigeon from Turkey and a chicken from Northern Ireland. A CC692 chicken isolate from South Korea was sequenced recently (K12S0375, GenBank: JYGF) but this was a MRSA. Generally, CC692 appears to be a bird-related lineage although it recently was observed in a grey seal (*Halichoerus grypus*) that may have preyed upon marine birds [48], in slaughterhouse workers [142] as well as in Australian wallabies [56].

### Clonal complex 707

One isolate belonging to CC707 was identified in a Swedish reindeer. It did not carry any resistance genes or IEC genes, and the genes, *tst1*, *sek*, *seq*, *ccrA/B2* and the *kdp* locus were absent although they are commonly found in CC707 isolates (Strain 21235 GenBank AFTQ and author´s unpublished observations). CC707 have, to the best of our knowledge, not been reported previously from animals.

### Sequence type 890

One isolate of this strain was identified, from a vole fecal sample and a *spa* type t1773 (04-82-17-25-17) was determined. The isolate carried the *blaZ* gene. The MLST database shows one entry only (http://saureus.mlst.net/sql/fulldetails.asp?id=1709&send=33), an isolate from a human from France. Wildlife observations of ST890 appear not to have been reported previously.

### Clonal complex 1956

One isolate of this lineage was identified from vole feces. MLST was performed identifying ST1959. The isolate lacked enterotoxin, and PVL genes, *lukF*-P83/*lukM*, IEC genes as well as any resistance markers. According to the MLST database, ST1959 has previously been found in a beaver (*Castor fiber*). The CC’s predicted founder, ST1956, was detected previously in a red squirrel and the related ST1960 was isolated from a human in Poland.

### Sequence type 2425

This novel ST (6-158-6-2-7-26-5) was identified from two isolates from European brown hares from Sweden. For one of them, abscesses in multiple organs were recorded. Isolates lacked any resistance genes but harbored *tst1*, *sec*, *sel* and ORF CM14. No resistance genes were detected. There are no related entries in the MLST database and therefore, no CC assignment is possible.

### Sequence type 2691

This was a novel singleton ST (6-79-12-2-7-13-153) identified in isolates from two Swedish moose. One suffered from a large abscess on the right lateral hock, accompanied by inflammation of the joint and loss of articular cartilage. The other one was found dead with pneumonia and sepsis. Isolates did not harbor any resistance genes. Enterotoxin and IEC were not detected.

### Clonal complex 2767

A novel, previously undescribed, ST (102-146-6-18-7-50-2) was identified twice, from the liver of a lynx and from an eye of a reindeer, both from Sweden. It was submitted to the MLST database and assigned ST2279. One isolate (from reindeer) harbored protease genes *splA* and *splB* as well as *lukD* which the other one lacked. Resistance, toxin and IEC genes were not detected. Later entries to the MLST database include two related STs (ST2767; 102-146-6-18-7-50-48 and ST3212; 102-146-416-18-7-50-48) that also originated from wildlife (*i*.*e*., from wild boars from Spain and Italy). ST2767 is the predicted founder of this clonal complex, hence the designation as CC2767.

### Sequence type 2963

This was a new singleton sequence type. Its MLST profile was 6-79-6-2-13-50-48. It was isolated from the same road-killed wildcat from North Rhine-Westphalia as mentioned above (see CC49). Enterotoxin, IEC and resistance genes were not detected.

### Sequence type 3237

One ST3237 isolate (6-380-6-18-62-70-406) was found in a cachectic sika deer from North Rhine-Westphalia. Enterotoxin genes, IEC genes, *cna* and all resistance markers tested were absent but *edinB* was present. The MLST database includes one ST3237 isolate (http://saureus.mlst.net/sql/fulldetails.asp?id=5984&send=225) that originated from an unspecified animal, from a geographically close area, Lower Saxony. Another related ST was ST2671 (6-380-6-18-62-70-304) that has been reported from Spanish red deer (http://saureus.mlst.net/sql/fulldetails.asp?id=5037&send=283).

## Discussion and Conclusions

The European wildlife isolates described in this study showed a high degree of diversity. The study is limited by the “opportunistic” mode of sampling used since the samples derived from animals necropsied within the frame of passive wildlife disease surveillance and from road-kill or hunted wildlife.

Thus, the present study, together with several others mentioned above, provides a lot of anecdotal evidence. However, there are not enough data to allow valid conclusions on prevalence and geographic distribution of *S*. *aureus*/MRSA in the different species of European wildlife as well as on possible temporal changes. This is unfortunate since host specificity and geographic distribution are important parameters for understanding ecology and epidemiology. Despite the rather anecdotal data presented here, our study has enabled the establishment of a valuable database of animal strains and their molecular characteristics. This will facilitate the rapid recognition of strains accidentally encountered in a human or livestock sample and providing a starting point for further more extensive studies. The DNA microarrays used in this study proved to be a convenient tool for rapid typing and for selecting unusual strains that might warrant further characterization such as MLST or ultimately genome sequencing.

Some of the *S*. *aureus* lineages observed in this study have been found in humans and/or in domestic animals. These lineages might have been transmitted from animals, wild or domestic, to humans. Others might have originated from humans and could have been transmitted to wildlife either indirectly by domestic animals, or by wildlife species that scavenge human offal. In this context, opportunistic and scavenging species such as martens, foxes, rats, crows/rooks, gulls and mallards could be screened as sentinels. Lineages that can be found among humans, livestock as well as wildlife include CC1, CC5, CC8, CC9, CC12, CC15, CC22, CC49, CC88, CC97, CC130, CC133 and ST425. For two lineages, CC707 and ST890, not enough data exist to speculate over host specificity and origin. Another lineage, CC692, appears to be largely restricted to birds, but can be found in birds of different orders and families. Some additional lineages have sporadically been observed in European wildlife before (ST1959, CC2767, CC2671, see above, and ST1643 [19]). Several other lineages described herein (ST2425, ST2691, ST2963) have not been observed previously. This might indicate that they do not play a role as agents of disease in humans or domestic animals and thus that their zoonotic potential might be limited. Unfortunately, no quantitative data on the presence of such “exotic” strains are available yet. Besides, many reports on *S*. *aureus* isolates in wildlife lack typing data (or refer to typing procedures that are not in use anymore) so that meaningful comparisons cannot be drawn. For most parts of the world, typing data for *S*. *aureus* animal isolates are not available even for domestic animals. For these reasons it might well be that an “exotic” or apparently rare wildlife strain might be, or become, a relevant pathogen in humans, livestock or wildlife without that being currently noticed.

Only a few markers have been proven in this and other studies to be associated with specific hosts. For instance, PVL is strongly associated with “human” strains while *lukF-P83/lukM* is associated with animal strains [5,155,161,162,163]. Human isolates usually carry lysogenic beta-hemolysin gene truncating phages, which are normally absent from ungulate strains [153,164,165]. It might be that some host-specific virulence factors were not identified because microarrays or PCRs, in contrast to genome sequencing, cannot detect “unknown” genes. However, none of the observed lineages were really “alien” (in the same sense as *S*. *argenteus* or *S*. *schweitzeri*) and all carried genetic traits (capsule types, *agr* alleles, *egc* locus, *lukD/E* etc.) as observed in human and livestock lineages, although the combinations of these traits varied (see Table 2). If no core genomic markers but only a couple of phages (see above) were associated with host specificity, an extensive potential of *S*. *aureus* for zoonotic and anthropo-zoonotic spill-overs must be assumed. This emphasizes the need for surveillance of livestock- and wildlife-associated MRSA well as a surveillance of human or livestock strains that might, for instance, infect endangered wildlife species. The observation of non-mobile genes in divergent lineages could indicate that horizontal gene transfer by recombination events (as suggested in [166]) might play a bigger role and be more common in *S*. *aureus* than previously appreciated.

Finally, it is also noteworthy which strains or lineages were *not* observed in this study.

Beside five rook isolates [77], there were no hospital- or community-associated *mecA*-MRSA, and more surprisingly, also just a single isolate from one of the known livestock-associated *mecA*-MRSA strains (CC398-MRSA-V). For the Swedish study arm, this might be attributed to the generally low prevalence of any MRSA in Sweden. For the German and Austrian samples, that might be related to the size of the study population and to the rather recent emergence of livestock-associated MRSA strains and to the fact that they are still rare in many regions.

The locally common cattle-associated lineages CC479 and CC705 [155,167] have not been found in wildlife at all while, in contrast, a third common cattle lineage, CC97, was frequently detected in cervids.

Interestingly, no *S*. *argenteus*- or *S*. *schweitzeri*-like isolates were found. This could indicate a geographical distribution strictly outside of Europe, or a presence in natural hosts that we were not able to sample. For instance, only a single bat was swabbed during the present study and it yielded no S. *aureus*, *S*. *argenteus* or *S*. *schweitzeri*. Therefore, a presence of either species in bats cannot be excluded and given extra-European experience with bats [40] this should be a focus for further study.

In conclusion, European wildlife harbors diverse lineages of *S*. *aureus*. Some are of public health or animal health interest while others appear to be rare and unique. Resistance rates in wildlife strains are rather low, which might be related to low selective pressures. This might change in the future due to environmental contamination with antibiotic compounds from hospital wastewater effluent and agriculture; and as mentioned above some common opportunistic and scavenging species might be suitable sentinels.

*mecA*-MRSA, including livestock-associated MRSA, were uncommon to virtually absent. Conversely, several *mecC*-MRSA were identified suggesting a wildlife reservoir. While most of the evidence is anecdotal, more systematic studies are required to monitor the effect of a possible influx of human- and livestock-associated *S*. *aureus*/MRSA into wildlife. Given the migratory habits of many birds, and the possibility of transmissions between wild and related domestic animals (for example between bovids and cervids, pigs and wild boars, chickens and partridges) or between predators and prey and a possible transmission by unprotected contact to tame, captive, injured or dead animals. The prevalence of *S*. *aureus*/MRSA in wildlife as well as the population structures of that pathogen in different host species warrants further investigation.

## Supporting Information

S1 Table Isolates, geographic origin, host species as well as full hybridization and typing data. (PDF)
