# Supplementary material for: Diversity of Staphylococcus aureus Isolates in European Wildlife
Source: PLoS One. 2016 Dec 16;11(12):e0168433. doi: 10.1371/journal.pone.0168433 (PMC5161505; doi:10.1371/journal.pone.0168433)
Supplement: S1 Table — (PDF) [file pone.0168433.s001.pdf]

ISOLATE ID	TYPING DATA						Country	Year of isolation	Species	Sample type	Diagnosis/Cause of death	SPECIES MARKER							REGULATORY GENES							
	CLONAL COMPLEX (by array)	STRAIN AFFILIATION (by array)	ST	MLST Profile	spa type	spa profile						Domain 1 of 23S-rRNA	glyceraldehyde 3-phosphate dehydrogenase, locus 1	katalase A	coagulase	thermostable extracellular nuclease	staphylococcal protein A	IgG-binding protein	staphylococcal accessory regulator A	histidine protein kinase, sae locus	sensor protein	accessory gene regulator allele I	accessory gene regulator allele II	accessory gene regulator allele III	accessory gene regulator allele IV	haemolysin delta
												Ribos. STAU	gapA	katA	CoA	nuc1	spa	sbi	sarA	saeS	vraS	agrI (total)	agrII (total)	agrIII (total)	agrIV (total)	hld
203228	CC0001	CC1-MSSA	N/A	N/A	N/A	N/A	Germany (NRW)	2013	Fallow deer	Nasal swab	Trauma (stitch damage)	POS	POS	POS	POS	POS	POS	POS	POS	POS	POS	NEG	NEG	POS	NEG	POS
203229	CC0001	CC1-MSSA	N/A	N/A	N/A	N/A	Germany (NRW)	2013	Fallow deer	Nasal swab	Found dead, lung and intestinal parasitosis	POS	POS	POS	POS	POS	POS	POS	POS	POS	POS	NEG	NEG	POS	NEG	POS
203233	CC0001	CC1-MSSA	N/A	N/A	N/A	N/A	Germany (NRW)	2013	Mouflon	Nasal swab	Laminitis, Trauma (shot)	POS	POS	POS	POS	POS	POS	POS	POS	POS	POS	NEG	NEG	POS	NEG	POS
220334	CC0001	CC1-MSSA	N/A	N/A	N/A	N/A	Germany	2013	Mouflon	Nasal swab	Ovine progressive adenocarcinoma	POS	POS	POS	POS	POS	POS	POS	POS	POS	POS	NEG	NEG	POS	NEG	POS
223621	CC0001	CC1-MSSA	N/A	N/A	N/A	N/A	Germany	2013	Red fox	Nasal swab	Trauma (shot)	POS	POS	POS	POS	POS	POS	POS	POS	POS	POS	NEG	NEG	POS	NEG	POS
289123	CC0001	CC1-MRSA-IV, WA MRSA-1/57	ST1	1-1-1-1-1-1-1	N/A	N/A	Austria	2013	Rook	Faecal sample	N/A	POS	POS	POS	POS	POS	POS	POS	POS	POS	POS	NEG	NEG	POS	NEG	POS
289124	CC0001	CC1-MRSA-IV, WA MRSA-1/57	ST1	1-1-1-1-1-1-1	N/A	N/A	Austria	2013	Rook	Faecal sample	N/A	POS	POS	POS	POS	POS	POS	POS	POS	POS	POS	NEG	NEG	POS	NEG	POS
275170	CC0005	CC5-MSSA	N/A	N/A	N/A	N/A	Germany (Pellworm)	2012	Brown hare	Tissue sample	Trauma (shot)	POS	POS	POS	POS	POS	POS	POS	POS	POS	POS	NEG	POS	NEG	NEG	POS
275171	CC0005	CC5-MSSA	N/A	N/A	N/A	N/A	Germany (Pellworm)	2012	Brown hare	Tissue sample	Trauma (shot)	POS	POS	POS	POS	POS	POS	POS	POS	POS	POS	NEG	POS	NEG	NEG	POS
112747	CC0005	CC5-MSSA	N/A	N/A	N/A	N/A	Sweden	2007	Grey partridge	Lung tissue	Purulent pneumonia, aerosacultis, peritonitis	POS	POS	POS	POS	POS	POS	POS	POS	POS	POS	NEG	POS	NEG	NEG	POS
112752	CC0006	CC6-MSSA	N/A	N/A	N/A	N/A	Sweden	2006	Red fox	Muscle tissue	Skin lesion, phlegmon, sepsis	POS	POS	POS	POS	POS	POS	POS	AMB	POS	POS	POS	NEG	NEG	AMB	POS
275178	CC0007	CC7-MSSA	N/A	N/A	N/A	N/A	Austria (Lower Austria)	2015	Red fox	Nasal swab	N/A	POS	POS	POS	POS	POS	POS	POS	POS	POS	POS	POS	NEG	NEG	NEG	POS
275166	CC0008	CC8-MSSA	N/A	N/A	N/A	N/A	Austria (Carinthia)	2012	Marmot	Faecal sample	N/A	POS	POS	POS	POS	POS	POS	POS	POS	POS	POS	POS	NEG	NEG	NEG	POS
275176	CC0008	CC8-MSSA	N/A	N/A	N/A	N/A	Austria (Salzburg)	2015	Mouflon	Nasal swab	Enteritis	POS	POS	POS	POS	POS	POS	POS	POS	POS	POS	POS	NEG	NEG	NEG	POS
275177	CC0008	CC8-MSSA	N/A	N/A	N/A	N/A	Austria (Lower Austria)	2015	Red fox	Nasal swab	N/A	POS	POS	POS	POS	POS	POS	POS	POS	POS	POS	POS	NEG	NEG	NEG	POS
275167	CC0009	CC9-MSSA	N/A	N/A	N/A	N/A	Austria (Burgenland)	2015	Wild boar	Skin swab	N/A	POS	POS	POS	POS	POS	POS	POS	POS	POS	POS	NEG	POS	NEG	NEG	POS
170968	CC0012	CC12-MSSA	N/A	N/A	N/A	N/A	Sweden	2012	Harbor porpoise	heart	N/A	POS	POS	POS	POS	POS	POS	POS	POS	POS	POS	NEG	POS	NEG	NEG	POS
275175	CC0015	CC15-MSSA	N/A	N/A	N/A	N/A	Austria (Lower Austria)	2010	Rook	Faecal sample	N/A	POS	POS	POS	POS	POS	POS	POS	POS	POS	POS	NEG	POS	NEG	NEG	POS
170949	CC0015	CC15-MSSA	N/A	N/A	N/A	N/A	Sweden	2005	Moose	liver abscess	Hepatitis	POS	POS	POS	POS	POS	POS	POS	POS	POS	POS	NEG	POS	NEG	NEG	POS
228658	CC0022	CC22-MSSA	N/A	N/A	N/A	N/A	Germany	2014	Red fox	Nasal swab	N/A	POS	POS	POS	POS	POS	POS	POS	POS	POS	POS	POS	NEG	NEG	AMB	POS
289125	CC0022	CC22-MRSA-IV (PVL+)	ST22	7-6-1-5-8 -8-6	N/A	N/A	Austria	2013	Rook	Faecal sample	N/A	POS	POS	POS	POS	POS	POS	POS	POS	POS	POS	POS	NEG	NEG	AMB	POS
289126	CC0022	CC22-MRSA-IV (PVL+)	ST22	7-6-1-5-8 -8-6	N/A	N/A	Austria	2013	Rook	Faecal sample	N/A	POS	POS	POS	POS	POS	POS	POS	POS	POS	POS	POS	NEG	NEG	AMB	POS
289127	CC0022	CC22-MRSA-IV (PVL+)	ST22	7-6-1-5-8 -8-6	N/A	N/A	Austria	2013	Rook	Faecal sample	N/A	POS	POS	POS	POS	POS	POS	POS	POS	POS	POS	POS	NEG	NEG	AMB	POS
131939	CC0025	CC25-MSSA	N/A	N/A	N/A	N/A	Germany	2011	Badger	Pharyngeal swab	Trauma (roadkill)	POS	POS	POS	POS	POS	POS	POS	POS	POS	POS	POS	NEG	NEG	NEG	POS
275165	CC0030	CC30-MSSA (lukF-P83/lukM+)	N/A	N/A	N/A	N/A	Austria	2012	Marmot	Intestine	N/A	POS	POS	POS	POS	POS	POS	POS	POS	POS	POS	NEG	NEG	POS	NEG	POS
275741	CC0030	CC30-MSSA	N/A	N/A	N/A	N/A	Austria	2015	Red deer	Nasal swab	N/A	POS	POS	POS	POS	POS	POS	POS	POS	POS	POS	NEG	NEG	POS	NEG	POS
124492	CC0049	CC49-MSSA (lukF-P83/lukM+)	CC49	14-16-11-2-13-12-2	t208	04-20-17-17-31-31-24-17-17-17-17-25	Germany (BY)	2010	Bank vole	Faecal sample	N/A	POS	POS	POS	POS	POS	POS	POS	POS	POS	POS	NEG	POS	NEG	NEG	POS
220616	CC0049	CC49-MSSA (lukF-P83/lukM+)	ST49	14-16-11-2-13-12-14	t208	04-20-17-17-31-31-24-17-17-17-17-25	Germany (BY)	2010	Bank vole	Faecal sample	N/A	POS	POS	POS	POS	POS	POS	POS	POS	POS	POS	NEG	POS	NEG	NEG	POS
228649	CC0049	CC49-MSSA (lukF-P83/lukM+)	N/A	N/A	N/A	N/A	Germany	2014	Wild cat	Nasal swab	Trauma (roadkill)	POS	POS	POS	POS	POS	POS	POS	POS	POS	POS	NEG	POS	NEG	NEG	POS
190994	CC0059	CC59-MSSA	N/A	N/A	N/A	N/A	Germany (TH)	2012	Wild boar	Nasal swab	Trauma (shot)	POS	POS	POS	AMB	POS	POS	POS	POS	POS	POS	POS	NEG	NEG	NEG	POS
275172	CC0088	CC88-MSSA	N/A	N/A	N/A	N/A	Austria (Lower Austria)	2013	Rook	Faecal sample	N/A	POS	POS	POS	POS	POS	POS	POS	POS	POS	POS	NEG	NEG	POS	NEG	POS
275173	CC0088	CC88-MSSA	N/A	N/A	N/A	N/A	Austria (Lower Austria)	2013	Rook	Faecal sample	N/A	POS	POS	POS	POS	POS	POS	POS	POS	POS	POS	NEG	NEG	POS	NEG	POS
275174	CC0088	CC88-MSSA	N/A	N/A	N/A	N/A	Austria (Lower Austria)	2013	Rook	Faecal sample	N/A	POS	POS	POS	POS	POS	POS	POS	POS	POS	POS	NEG	NEG	POS	NEG	POS
170971	CC0097	CC97-MSSA	N/A	N/A	N/A	N/A	Sweden	2012	Golden eagle	Joint	Emaciation, trauma	POS	POS	POS	POS	POS	POS	POS	POS	POS	POS	POS	NEG	NEG	NEG	POS
112745	CC0097	CC97-MSSA	N/A	N/A	N/A	N/A	Sweden	2004	Moose	Ear swab	Emaciation	POS	POS	POS	POS	POS	POS	POS	POS	POS	POS	POS	NEG	NEG	AMB	POS
112748	CC0097	CC97-MSSA	N/A	N/A	N/A	N/A	Sweden	2006	Moose	Ear swab	Parasitic abomasitis	POS	POS	POS	POS	POS	POS	POS	POS	POS	POS	POS	NEG	NEG	AMB	POS
112750	CC0097	CC97-MSSA	N/A	N/A	N/A	N/A	Sweden	2006	Moose	Ear swab	No lesions observed	POS	POS	POS	POS	POS	POS	POS	POS	POS	POS	POS	NEG	NEG	NEG	POS
112762	CC0097	CC97-MSSA	N/A	N/A	N/A	N/A	Sweden	2008	Moose	Lung tissue	Purulent pneumonia	POS	POS	POS	POS	POS	POS	POS	POS	POS	POS	POS	NEG	NEG	NEG	POS
112763	CC0097	CC97-MSSA	N/A	N/A	N/A	N/A	Sweden	2008	Moose	Skin swab	Ulcerative dermatitis	POS	POS	POS	POS	POS	POS	POS	POS	POS	POS	POS	NEG	NEG	AMB	POS
112764	CC0097	CC97-MSSA	N/A	N/A	N/A	N/A	Sweden	2009	Moose	Lung tissue	Parasitic pneumonia, non-purulent meningoencephalits	POS	POS	POS	POS	POS	POS	POS	POS	POS	POS	POS	NEG	NEG	AMB	POS
170945	CC0097	CC97-MSSA	N/A	N/A	N/A	N/A	Sweden	2010	Moose	Liver tissue	Sepsis	POS	POS	POS	POS	POS	POS	POS	POS	POS	POS	POS	NEG	NEG	NEG	POS
170954	CC0097	CC97-MSSA	N/A	N/A	N/A	N/A	Sweden	2010	Moose	Heart	Pericarditis, peritonitis	POS	POS	POS	POS	POS	POS	POS	POS	POS	POS	POS	NEG	NEG	NEG	POS
170958	CC0097	CC97-MSSA	N/A	N/A	N/A	N/A	Sweden	2011	Moose	Skin	Dermatitis	POS	POS	POS	POS	POS	POS	POS	POS	POS	POS	POS	NEG	NEG	NEG	POS
170963	CC0097	CC97-MSSA	N/A	N/A	N/A	N/A	Sweden	2011	Moose	Skin	Chronic dermatitis	POS	POS	POS	POS	POS	POS	POS	POS	POS	POS	POS	NEG	NEG	NEG	POS
170964	CC0097	CC97-MSSA	N/A	N/A	N/A	N/A	Sweden	2011	Moose	Skin	Parasitic otitis with secondary bacterial infection	POS	POS	POS	POS	POS	POS	POS	POS	POS	POS	POS	NEG	NEG	NEG	POS
170965	CC0097	CC97-MSSA	N/A	N/A	N/A	N/A	Sweden	2011	Moose	Skin	Parasitic dermatitis	POS	POS	POS	POS	POS	POS	POS	POS	POS	POS	POS	NEG	NEG	NEG	POS
170966	CC0097	CC97-MSSA	N/A	N/A	N/A	N/A	Sweden	2011	Moose	Skin	Chronic dermatitis	POS	POS	POS	POS	POS	POS	POS	POS	POS	POS	POS	NEG	NEG	NEG	POS
170974	CC0097	CC97-MSSA	N/A	N/A	N/A	N/A	Sweden	2012	Moose	Skin	Otitis externa, parasitic otitis	POS	POS	POS	POS	POS	POS	POS	POS	POS	POS	POS	NEG	NEG	NEG	POS
170976	CC0097	CC97-MSSA	N/A	N/A	N/A	N/A	Sweden	2012	Moose	skin	Hyperplastic perivascular dermatitis, purulent pleuritis	POS	POS	POS	POS	POS	POS	POS	POS	POS	POS	POS	NEG	NEG	NEG	POS
112744	CC0097	CC97-MSSA	N/A	N/A	N/A	N/A	Sweden	2004	Roe deer	Lung tissue	Pneumonia	POS	POS	POS	POS	POS	POS	POS	POS	POS	POS	POS	NEG	NEG	NEG	POS
112749	CC0097	CC97-MSSA	N/A	N/A	N/A	N/A	Sweden	2006	Roe deer	Abscess	Abscess	POS	POS	POS	POS	POS	POS	POS	POS	POS	POS	POS	NEG	NEG	AMB	POS
112757	CC0097	CC97-MSSA	N/A	N/A	N/A	N/A	Sweden	2007	Roe deer	Skin abscess	Skin abscess	POS	POS	POS	POS	POS	POS	POS	POS	POS	POS	POS	NEG	NEG	AMB	POS
170973	CC0097	CC97-MSSA	N/A	N/A	N/A	N/A	Sweden	2012	Roe deer	Lung tissue	Pneumonia	POS	POS	POS	POS	POS	POS	POS	POS	POS	POS	POS	NEG	NEG	NEG	POS
207756	CC0097	CC97-MSSA	N/A	N/A	N/A	N/A	Germany (NRW)	2013	Roe deer	Nasal swab	Found dead, lung and intestinal parasites	POS	POS	POS	POS	POS	POS	POS	POS	POS	POS	POS	NEG	NEG	AMB	POS
206171	CC0097	CC97-MSSA	N/A	N/A	N/A	N/A	Germany (NRW)	2013	Roe deer	Nasal swab	Found dead, multiple abscesses; lung and intestinal parasitosis	POS	POS	POS	POS	POS	POS	POS	POS	POS	POS	POS	NEG	NEG	AMB	POS
275169	CC0097	CC97-MSSA-[ccrAA]	N/A	N/A	N/A	N/A	Austria (Burgenland)	2015	Wild boar	Skin swab	N/A	POS	POS	POS	POS	POS	POS	POS	POS	POS	POS	POS	NEG	NEG	NEG	POS
275168	CC0097	CC97-MSSA-[ccrAA]	N/A	N/A	N/A	N/A	Austria (Burgenland)	2015	Wild boar	Skin swab	N/a	POS	POS	POS	POS	POS	POS	POS	POS	POS	POS	POS	NEG	NEG	NEG	POS
118320	CC0130	CC130-MSSA (lukF-P83/M+)	ST2024	6- 57-45-2-219-58-52	t8403	04-82-17-25-25-25-16-17	Germany (Berlin)	2010	Brown rat	Faecal sample	N/A	POS	POS	POS	POS	POS	POS	POS	POS	POS	POS	NEG	NEG	POS	NEG	POS
203236	CC0130	CC130-MRSA-XI	N/A	N/A	N/A	N/A	Germany (NRW)	2013	Fallow deer	Nasal swab	Trauma (shot), enteritis	POS	POS	POS	POS	POS	POS	POS	POS	POS	POS	NEG	NEG	POS	NEG	POS
204204	CC0130	CC130-MRSA-XI	N/A	N/A	N/A	N/A	Germany (NRW)	2013	Red fox	Nasal swab	Blunt Trauma, found dead	POS	POS	POS	POS	POS	POS	POS	POS	POS	POS	NEG	NEG	POS	NEG	POS
289122	CC0130	CC130-MRSA-XI	N/A	N/A	N/A	N/A	Germany (TH)	2016	Hedgehog	Nasal swab	Live and apparently healthy individual	POS	POS	POS	POS	POS	POS	POS	POS	POS	POS	NEG	NEG	POS	NEG	POS
225779	CC0130	CC130-MRSA-XI	ST2944	6-57-45-2-7-318-52	N/A	N/A	Germany (Pellworm)	2012	Brown hare	Tissue sample	Trauma (shot)	POS	POS	POS	POS	POS	POS	POS	POS	POS	POS	NEG	NEG	POS	NEG	POS
225780	CC0130	CC130-MRSA-XI	ST2944	6-57-45-2-7-318-52	N/A	N/A	Germany (Pellworm)	2012	Brown hare	Tissue sample	Trauma (shot)	POS	POS	POS	POS	POS	POS	POS	POS	POS	POS	NEG	NEG	POS	NEG	POS
170959	CC0130	CC130-MRSA-XI	ST130	6-57-45-2-7-58-52	t843	04-82-17-25-17-25-25-16-17	Sweden /Gotland	2011	Hedgehog	Skin lesion	Diffuse dermatitis, diarrhoea; euthanised	POS	POS	POS	POS	POS	POS	POS	POS	POS	POS	NEG	NEG	POS	NEG	POS
179029	CC0130	CC130-MRSA-XI	ST130	6-57-45-2-7-58-52	t5771	04-17-25-16-17	Sweden	2003	Hedgehog	Kidney	Found dead	POS	POS	POS	POS	POS	POS	POS	POS	POS	POS	NEG	NEG	AMB	NEG	POS
116254	CC0133	CC133-MSSA	N/A	N/A	N/A	N/A	Germany (Lower Saxony)	2013	Wild boar	Nasal swab	Trauma (shot)	POS	POS	POS	POS	POS	POS	POS	POS	POS	POS	POS	NEG	NEG	NEG	POS
207761	CC0133	CC133-MSSA	N/A	N/A	N/A	N/A	Germany (NRW)	2013	Roe deer	Nasal swab	Trauma (shot), cachexia, lung and intestinal parasitosis	POS	POS	POS	POS	POS	POS	POS	POS	POS	POS	POS	NEG	NEG	NEG	POS
213103	CC0133	CC133-MSSA	N/A	N/A	N/A	N/A	Germany (NRW)	2013	Roe deer	Nasal swab	Squamous cell carcinoma at the neck	POS	POS	POS	POS	POS	POS	POS	POS	POS	POS	POS	NEG	NEG	AMB	POS
275736	CC0133	CC133-MSSA	N/A	N/A	N/A	N/A	Austria (Salzburg)	2015	Roe deer	Nasal swab	N/A	POS	POS	POS	POS	POS	POS	POS	POS	POS	POS	POS	NEG	NEG	NEG	POS
275737	CC0133	CC133-MSSA	N/A	N/A	N/A	N/A	Austria (Salzburg)	2015	Roe deer	Nasal swab	N/A	POS	POS	POS	POS	POS	POS	POS	POS	POS	POS	POS	NEG	NEG	NEG	POS
275738	CC0133	CC133-MSSA	N/A	N/A	N/A	N/A	Austria (Salzburg)	2015	Roe deer	Nasal swab	N/A	POS	POS	POS	POS	POS	POS	POS	POS	POS	POS	POS	NEG	NEG	NEG	POS
275739	CC0133	CC133-MSSA	N/A	N/A	N/A	N/A	Austria (Salzburg)	2015	Chamois	Nasal swab	N/A	POS	POS	POS	POS	POS	POS	POS	POS	POS	POS	POS	NEG	NEG	NEG	POS
112755	CC0133	CC133-MSSA	ST2111	06-66-46-2-7-50-7	N/A	N/A	Sweden	2007	Mute Swan	Liver tissue	Sepsis	POS	POS	POS	POS	POS	POS	POS	POS	POS	POS	POS	NEG	NEG	NEG	POS
225778	CC0398	CC398-MRSA-V/VT	ST398	3-35-19-2-20-26-39	N/A	N/A	Germany (Pellworm)	2012	Brown hare	Tissue sample	Trauma (shot)	POS	POS	POS	POS	POS	POS	POS	POS	POS	POS	POS	NEG	NEG	NEG	POS
123159	ST0425	ST425-MSSA	N/A	N/A	N/A	N/A	Germany (TH)	2010	Badger	Nasal swab	Trauma (roadkill)	POS	POS	POS	POS	POS	POS	POS	POS	POS	POS	NEG	POS	NEG	NEG	POS
86991	ST0425	ST425-MSSA	ST425	18-33-6-20-7-50-48	undefined, related to t5386	14-44-12-17-23-44-12-17-17-17-17-23-24	Germany (TH)	2006	Badger	Nasal swab	Generalised ulcerations	POS	POS	POS	POS	POS	POS	POS	POS	POS	POS	NEG	POS	NEG	NEG	POS
112756	ST0425	ST425-MSSA	N/A	N/A	N/A	N/A	Sweden	2007	Badger	Liver tissue	No lesions observed	POS	POS	POS	POS	POS	POS	POS	POS	POS	POS	NEG	POS	NEG	NEG	POS
195207	ST0425	ST425-MSSA	N/A	N/A	N/A	N/A	Germany (NRW)	2013	Wild boar	Nasal swab	Found dead, pneumonia, lung/intestinal parasitosis, alveolar echinococcosis in liver	POS	POS	POS	POS	POS	POS	POS	POS	POS	POS	NEG	POS	NEG	NEG	POS
223609	ST0425	ST425-MSSA	N/A	N/A	N/A	N/A	Germany	2013	Red fox	Nasal swab	Trauma (shot), scabies	POS	POS	POS	POS	POS	POS	POS	POS	POS	POS	NEG	POS	NEG	NEG	POS
275740	ST0425	ST425-MSSA	N/A	N/A	N/A	N/A	Austria (Salzburg)	2013	Red deer	Nasal swab	N/A	POS	POS	POS	POS	POS	POS	POS	POS	POS	POS	NEG	POS	NEG	NEG	POS
174889	ST0425	ST425-MSSA	N/A	N/A	N/A	N/A	Germany (TH)	2012	Red deer	Nasal swab	Trauma (shot)	POS	POS	POS	POS	POS	POS	POS	POS	POS	POS	NEG	POS	NEG	NEG	POS
203230	ST0425	ST425-MSSA	N/A	N/A	N/A	N/A	Germany (NRW)	2013	Roe deer	Nasal swab	Trauma (shot), rumenitis	POS	POS	POS	POS	POS	POS	POS	POS	POS	POS	NEG	POS	NEG	NEG	POS
203242	ST0425	ST425-MSSA	N/A	N/A	N/A	N/A	Germany (NRW)	2013	Roe deer	Nasal swab	Trauma (shot), lung wurms, enteritis	POS	POS	POS	POS	POS	POS	POS	POS	POS	POS	NEG	POS	NEG	NEG	POS
203243	ST0425	ST425-MSSA	N/A	N/A	N/A	N/A	Germany (NRW)	2013	Roe deer	Nasal swab	Found dead, cause unknown	POS	POS	POS	POS	POS	POS	POS	POS	POS	POS	NEG	POS	NEG	NEG	POS
207951	ST0425	ST425-MSSA	N/A	N/A	N/A	N/A	Germany (NRW)	2013	Roe deer	Nasal swab	N/A	POS	POS	POS	POS	POS	POS	POS	POS	POS	POS	NEG	POS	NEG	NEG	POS
207981	ST0425	ST425-MSSA	N/A	N/A	N/A	N/A	Germany (NRW)	2013	Roe deer	Nasal swab	N/A	POS	POS	POS	POS	POS	POS	POS	POS	POS	POS	NEG	POS	NEG	NEG	POS
195212	ST0425	ST425-MSSA	N/A	N/A	N/A	N/A	Germany (NRW)	2012	Roe deer	Nasal swab	Very old animal, found dead, lung neoplasm, indigestion	POS	POS	POS	POS	POS	POS	POS	POS	POS	POS	NEG	POS	NEG	NEG	POS
195206	ST0425	ST425-MSSA	N/A	N/A	N/A	N/A	Germany (NRW)	2013	Roe deer	Nasal swab	Found dead, metabolic disorder/intoxication?	POS	POS	POS	POS	POS	POS	POS	POS	POS	POS	NEG	POS	NEG	NEG	POS
195202	ST0425	ST425-MSSA	N/A	N/A	N/A	N/A	Germany (NRW)	2013	Roe deer	Nasal swab	Found dead, blunt head trauma	POS	POS	POS	POS	POS	POS	POS	POS	POS	POS	NEG	POS	NEG	NEG	POS
216042	ST0425	ST425-MSSA	N/A	N/A	N/A	N/A	Germany (NRW)	2013	Roe Deer	Nasal swab	Found dead, verminous pneumonia	POS	POS	POS	POS	POS	POS	POS	POS	POS	POS	NEG	POS	NEG	NEG	POS
223608	ST0425	ST425-MSSA	N/A	N/A	N/A	N/A	Germany (NRW)	2013	Roe Deer	Nasal swab	Pyogranulomatous pneumonia	POS	POS	POS	POS	POS	POS	POS	POS	POS	POS	NEG	POS	NEG	NEG	POS
223612	ST0425	ST425-MSSA	N/A	N/A	N/A	N/A	Germany (NRW)	2013	Roe Deer	Nasal swab	Found dead, lung and intestinal parasitosis	POS	POS	POS	POS	POS	POS	POS	POS	POS	POS	NEG	POS	NEG	NEG	POS
225733	ST0425	ST425-MSSA	N/A	N/A	N/A	N/A	Germany (NRW)	2013	Roe Deer	Nasal swab	Found dead, trauma	POS	POS	POS	POS	POS	POS	POS	POS	POS	POS	NEG	POS	NEG	NEG	POS
230321	ST0425	ST425-MSSA	N/A	N/A	N/A	N/A	Germany (NRW)	2014	Roe deer	Nasal swab	Found dead, pneumonia, lung and intestinal parasitosis	POS	POS	POS	POS	POS	POS	POS	POS	POS	POS	NEG	POS	NEG	NEG	POS
232368	ST0425	ST425-MSSA	N/A	N/A	N/A	N/A	Germany (NRW)	2014	Roe deer	Nasal swab	Found dead, pulmonary mycetoma	POS	POS	POS	POS	POS	POS	POS	POS	POS	POS	NEG	POS	NEG	NEG	POS
204215	ST0425	ST425-MSSA	N/A	N/A	N/A	N/A	Germany (NRW)	2013	Roe deer	Nasal swab	Killed, cerebral abscess	POS	POS	POS	POS	POS	POS	POS	POS	POS	POS	NEG	POS	NEG	NEG	POS
204218	ST0425	ST425-MSSA	N/A	N/A	N/A	N/A	Germany (NRW)	2013	Roe deer	Nasal swab	Found dead, demodicosis, lung and intestinal parasitosis	POS	POS	POS	POS	POS	POS	POS	POS	POS	POS	NEG	POS	NEG	NEG	POS
204222	ST0425	ST425-MSSA	N/A	N/A	N/A	N/A	Germany (NRW)	2013	Roe deer	Nasal swab	Found dead, purulent osteomyelitis, bronchopneumonia	POS	POS	POS	POS	POS	POS	POS	POS	POS	POS	NEG	POS	NEG	NEG	POS
204226	ST0425	ST425-MSSA	N/A	N/A	N/A	N/A	Germany (NRW)	2013	Roe deer	Nasal swab	Trauma (shot), myositis, lung worms	POS	POS	POS	POS	POS	POS	POS	POS	POS	POS	NEG	POS	NEG	NEG	POS
204906	ST0425	ST425-MSSA	N/A	N/A	N/A	N/A	Germany (NRW)	2013	Roe deer	Nasal swab	Trauma (shot), dermal parakeratosis	POS	POS	POS	POS	POS	POS	POS	POS	POS	POS	NEG	POS	NEG	NEG	POS
204908	ST0425	ST425-MSSA	N/A	N/A	N/A	N/A	Germany (NRW)	2013	Roe deer	Nasal swab	Trauma (shot), lung and intestinal parasitosis, mild focal non suppurative encephalitis	POS	POS	POS	POS	POS	POS	POS	POS	POS	POS	NEG	POS	NEG	NEG	POS
206170	ST0425	ST425-MSSA	N/A	N/A	N/A	N/A	Germany (NRW)	2013	Roe deer	Nasal swab	Found dead; lung and intestinal parasitosis	POS	POS	POS	POS	POS	POS	POS	POS	POS	POS	NEG	POS	NEG	NEG	POS
207765	ST0425	ST425-MSSA	N/A	N/A	N/A	N/A	Germany (NRW)	2013	Roe deer	Nasal swab	Found dead, cachexia, lung and intestinal parasitosis	POS	POS	POS	POS	POS	POS	POS	POS	POS	POS	NEG	POS	NEG	NEG	POS
240048	CC599	CC599-MRSA-XI	CC599	6-158-276-2-7-32-xxx	N/A	N/A	Germany (TH)	2014	Hedgehog	Pharyngeal swab	Trauma (roadkill)	POS	POS	POS	POS	POS	POS	POS	POS	POS	POS	POS	NEG	NEG	NEG	POS
225730	CC0692	CC692-MSSA	N/A	N/A	N/A	N/A	Germany (BB)	2013	Red kite	Wound swab	Bullous eruption at the knee	POS	POS	POS	POS	POS	POS	POS	POS	POS	POS	POS	NEG	NEG	NEG	POS
112746	CC0692	CC692-MSSA	N/A	N/A	N/A	N/A	Sweden	2004	Green woodpecker	Liver tissue	Enteritis	POS	POS	POS	POS	POS	POS	POS	POS	POS	POS	POS	NEG	NEG	NEG	POS
112754	CC0692	CC692-MSSA	N/A	N/A	N/A	N/A	Sweden	2007	Common magpie	Abscess	Avian pox	POS	POS	POS	POS	POS	POS	POS	POS	POS	POS	POS	NEG	NEG	NEG	POS
112758	CC0692	CC692-MSSA	N/A	N/A	N/A	N/A	Sweden	2007	Great tit	Liver tissue	Avian pox	POS	POS	POS	POS	POS	POS	POS	POS	POS	POS	POS	NEG	NEG	AMB	POS
112759	CC0692	CC692-MSSA	N/A	N/A	N/A	N/A	Sweden	2008	Common magpie	Liver tissue	Avian pox	POS	POS	POS	POS	POS	POS	POS	POS	POS	POS	POS	NEG	NEG	AMB	POS
124745	CC0692	CC692-MSSA	ST692	12-89-1-1-4-5-90	N/A	N/A	Sweden	2008	Whitetailed Eagle	Spleen tissue	Sepsis	POS	POS	POS	POS	POS	POS	POS	POS	POS	POS	POS	NEG	NEG	AMB	POS
170952	CC0692	CC692-MSSA	N/A	N/A	N/A	N/A	Sweden	2010	Tawny owl	Tongue	Necrotizing pharyngitis	POS	POS	POS	POS	POS	POS	POS	POS	POS	POS	POS	NEG	NEG	AMB	POS
170953	CC0692	CC692-MSSA	N/A	N/A	N/A	N/A	Sweden	2010	Common magpie	Spleen	Granulomatous stomatitis	POS	POS	POS	POS	POS	POS	POS	POS	POS	POS	POS	NEG	NEG	NEG	POS
170970	CC0692	CC692-MSSA	N/A	N/A	N/A	N/A	Sweden	2012	Golden eagle	Joint	Emaciation, trauma	POS	POS	POS	POS	POS	POS	POS	POS	POS	POS	POS	NEG	NEG	NEG	POS
170950	CC0707	CC707-MSSA	N/A	N/A	N/A	N/A	Sweden	2010	Reindeer	Eye	Chronic keratitis	POS	POS	POS	POS	POS	POS	POS	POS	POS	POS	NEG	NEG	POS	NEG	POS
124948	ST0890	ST890-MSSA	ST890	18-33-45-18-109-14-2	t1773	04-82-17-25-17	Germany (BY)	2010	Bank vole	Faecal sample	N/A	POS	POS	POS	NEG	POS	POS	POS	POS	POS	POS	NEG	NEG	NEG	POS	POS
220618	CC1956	CC1956-MSSA	ST1959	6-291-6-2-7-225-2	t3058	14-44-34-17-34-50-17	Germany (MV)	2010	Bank vole	Faecal sample	N/A	POS	POS	POS	POS	POS	POS	POS	POS	POS	POS	NEG	NEG	NEG	POS	POS
112742	ST2425	ST2425-MSSA	ST2425	6-158-6-2-7-26-5	N/A	N/A	Sweden	2003	Brown hare	Lung	Multiple abscesses in lungs, heart, lymph nodes, co-infection with Francisella tularensis	POS	POS	POS	AMB	POS	POS	POS	POS	POS	POS	NEG	NEG	NEG	POS	POS
170957	ST2425	ST2425-MSSA	N/A	N/A	N/A	N/A	Sweden	2011	Brown hare	Spleen	Multiple abscesses in internal organs, mesenterium, spleen	POS	POS	POS	NEG	POS	POS	POS	POS	POS	POS	NEG	NEG	NEG	POS	POS
170947	ST2691	ST2691-MSSA	ST2691	6-79-12-2-7-13-153	N/A	N/A	Sweden, Skane	2010	Moose, female calf	Abscess	Large abscess on the right hock	POS	POS	POS	POS	POS	POS	POS	POS	POS	POS	NEG	POS	NEG	NEG	POS
170961	ST2691	ST2691-MSSA	ST2691	6-79-12-2-7-13-153	N/A	N/A	Sweden, Hallstahammar	2011	Moose, male calf	Skin	Found dead with pneumonia and sepsis	POS	POS	POS	POS	POS	POS	POS	POS	POS	POS	NEG	POS	NEG	NEG	POS
112753	CC2767	ST2279-MSSA	ST2279	102-146-6-18-7-50-2	N/A	N/A	Sweden	2006	Lynx	Liver tissue	Sarcoptic mange	POS	POS	POS	POS	POS	POS	POS	POS	POS	POS	POS	NEG	NEG	POS	POS
112761	CC2767	ST2279-MSSA	ST2279	102-146-6-18-7-50-2	N/A	N/A	Sweden	2008	Reindeer	Eye	Keratitis	POS	POS	POS	POS	POS	POS	POS	POS	POS	POS	POS	NEG	NEG	AMB	POS
228651	ST2963	ST2963-MSSA	ST2963	6-79-6-2-13-50-48	N/A	N/A	Germany (NRW)	2014	Wild cat	Nasal swab	Trauma (roadkill)	POS	POS	POS	POS	POS	POS	POS	POS	POS	POS	NEG	NEG	NEG	POS	POS
203244	ST3237	ST3237-MSSA	ST3237	6-380-6-18-62-70-406	N/A	N/A	Germany (NRW)	2013	Sika deer	Nasal Swab	Found dead	POS	POS	POS	POS	POS	POS	POS	POS	POS	POS	NEG	NEG	NEG	POS	POS

			METHICILLIN RESISTANCE AND SCCmec TYPING																					METHICILLIN RESISTANCE AND SCCmec TYPING						
ISOLATE ID	CLONAL COMPLEX (by array)	STRAIN AFFILIATION (by array)	alternate penicillin binding protein 2, defining MRSA	truncated signal transducer protein MecR1	glycerophosphoryl diester phosphodi-esterase, associated with mecA	cassette chromosome recombinase genes A/B-1		plasmin-sensitive surface protein	hypothetical protein from SCCmec elements	cassette chromosome recombinase genes A/B-2		potassium-trans-locating ATPase A, chain 2	potassium-transporting ATPase B, chain 1	potassium-trans-locating ATPase C, chain 2	sensor kinase protein	KDP operon transcrip-tional regulatory protein	methicillin-resistance regulatory protein	signal transducer protein MecR1	homolog of xylose repressor, associated with SCCmec-elements	cassette chromosome recombinase genes A/B-3		mercury resistance operon		cassette chromosome recombinase genes "ccrAA" (hypothetical) and ccrC			cassette chromosome recombinase genes A/B-4		SCCmec XI	
			mecA	delta_mecR	ugpQ	ccrA-1	ccrB-1	plsSCC (COL)	Q9XB68-dcs	ccrA-2	ccrB-2	kdpA-SCC	kdpB-SCC	kdpC-SCC	kdpD-SCC	kdpE-SCC	mecI	mecR	xylR	ccrA-3	ccrB-3	merA	merB	ccrAA (MRSAZH47)_probe 1	ccrAA (MRSAZH47)_probe 2	ccrC (85-2082)	ccrA-4	ccrB-4	mecC	blaZ-SCCmec XI
203228	CC0001	CC1-MSSA	NEG	NEG	NEG	NEG	AMB	NEG	NEG	NEG	NEG	NEG	NEG	NEG	NEG	NEG	NEG	NEG	AMB	NEG	NEG	NEG	NEG	NEG	NEG	NEG	NEG	NEG	0	0
203229	CC0001	CC1-MSSA	NEG	NEG	NEG	NEG	AMB	NEG	NEG	NEG	NEG	NEG	NEG	NEG	NEG	NEG	NEG	POS	POS	NEG	NEG	NEG	NEG	NEG	NEG	NEG	NEG	NEG	0	0
203233	CC0001	CC1-MSSA	NEG	NEG	NEG	NEG	NEG	NEG	NEG	NEG	NEG	NEG	NEG	NEG	NEG	NEG	NEG	NEG	NEG	NEG	NEG	NEG	NEG	NEG	NEG	NEG	NEG	NEG	0	0
220334	CC0001	CC1-MSSA	NEG	NEG	NEG	NEG	NEG	NEG	NEG	NEG	NEG	NEG	NEG	NEG	NEG	NEG	NEG	NEG	NEG	NEG	NEG	NEG	NEG	NEG	NEG	NEG	NEG	NEG	NEG	NEG
223621	CC0001	CC1-MSSA	NEG	NEG	NEG	NEG	NEG	NEG	POS	NEG	NEG	NEG	NEG	NEG	NEG	NEG	NEG	NEG	NEG	NEG	NEG	NEG	NEG	NEG	NEG	NEG	NEG	NEG	NEG	NEG
289123	CC0001	CC1-MRSA-IV, WA MRSA-1/57	POS	POS	POS	NEG	NEG	NEG	NEG	POS	POS	NEG	NEG	NEG	NEG	NEG	NEG	NEG	NEG	NEG	NEG	NEG	NEG	NEG	NEG	NEG	NEG	NEG	NEG	NEG
289124	CC0001	CC1-MRSA-IV, WA MRSA-1/57	POS	POS	POS	NEG	NEG	NEG	NEG	POS	POS	NEG	NEG	NEG	NEG	NEG	NEG	NEG	NEG	NEG	NEG	NEG	NEG	NEG	NEG	NEG	NEG	NEG	NEG	NEG
275170	CC0005	CC5-MSSA	NEG	NEG	NEG	NEG	NEG	NEG	NEG	NEG	NEG	NEG	NEG	NEG	NEG	NEG	NEG	NEG	NEG	NEG	NEG	NEG	NEG	NEG	NEG	NEG	NEG	NEG	NEG	NEG
275171	CC0005	CC5-MSSA	NEG	NEG	NEG	NEG	NEG	NEG	NEG	NEG	NEG	NEG	NEG	NEG	NEG	NEG	NEG	NEG	NEG	NEG	NEG	NEG	NEG	NEG	NEG	NEG	NEG	NEG	NEG	NEG
112747	CC0005	CC5-MSSA	NEG	NEG	NEG	NEG	NEG	NEG	NEG	NEG	NEG	NEG	NEG	NEG	NEG	NEG	NEG	NEG	NEG	NEG	NEG	NEG	NEG	NEG	NEG	NEG	NEG	NEG	0	0
112752	CC0006	CC6-MSSA	NEG	NEG	NEG	NEG	NEG	NEG	NEG	NEG	NEG	NEG	NEG	NEG	NEG	NEG	NEG	NEG	NEG	NEG	NEG	NEG	NEG	NEG	NEG	NEG	NEG	NEG	0	0
275178	CC0007	CC7-MSSA	NEG	NEG	NEG	NEG	NEG	NEG	NEG	NEG	NEG	NEG	NEG	NEG	NEG	NEG	NEG	NEG	NEG	NEG	NEG	NEG	NEG	NEG	NEG	NEG	NEG	NEG	NEG	NEG
275166	CC0008	CC8-MSSA	NEG	NEG	NEG	NEG	NEG	NEG	NEG	NEG	NEG	NEG	NEG	NEG	NEG	NEG	NEG	NEG	NEG	NEG	NEG	NEG	NEG	NEG	NEG	NEG	NEG	NEG	NEG	NEG
275176	CC0008	CC8-MSSA	NEG	NEG	NEG	NEG	NEG	NEG	NEG	NEG	NEG	NEG	NEG	NEG	NEG	NEG	NEG	NEG	NEG	NEG	NEG	NEG	NEG	NEG	NEG	NEG	NEG	NEG	NEG	NEG
275177	CC0008	CC8-MSSA	NEG	NEG	NEG	NEG	NEG	NEG	NEG	NEG	NEG	NEG	NEG	NEG	NEG	NEG	NEG	NEG	NEG	NEG	NEG	NEG	NEG	NEG	NEG	NEG	NEG	NEG	NEG	NEG
275167	CC0009	CC9-MSSA	NEG	NEG	NEG	NEG	NEG	NEG	NEG	NEG	NEG	NEG	NEG	NEG	NEG	NEG	NEG	NEG	NEG	NEG	NEG	NEG	NEG	NEG	NEG	NEG	NEG	NEG	NEG	NEG
170968	CC0012	CC12-MSSA	NEG	NEG	NEG	NEG	NEG	NEG	NEG	NEG	NEG	NEG	NEG	NEG	NEG	NEG	NEG	NEG	NEG	NEG	NEG	NEG	NEG	NEG	NEG	NEG	NEG	NEG	0	0
275175	CC0015	CC15-MSSA	NEG	NEG	NEG	NEG	NEG	NEG	NEG	NEG	NEG	NEG	NEG	NEG	NEG	NEG	NEG	NEG	NEG	NEG	NEG	NEG	NEG	NEG	NEG	NEG	NEG	NEG	NEG	NEG
170949	CC0015	CC15-MSSA	NEG	NEG	NEG	NEG	NEG	NEG	NEG	NEG	NEG	NEG	NEG	NEG	NEG	NEG	NEG	NEG	NEG	NEG	NEG	NEG	NEG	NEG	NEG	NEG	NEG	NEG	0	0
228658	CC0022	CC22-MSSA	NEG	NEG	NEG	NEG	NEG	NEG	NEG	NEG	NEG	NEG	NEG	NEG	NEG	NEG	NEG	NEG	NEG	NEG	NEG	NEG	NEG	NEG	NEG	NEG	NEG	NEG	NEG	NEG
289125	CC0022	CC22-MRSA-IV (PVL+)	POS	POS	POS	NEG	NEG	NEG	POS	POS	POS	NEG	NEG	NEG	NEG	NEG	NEG	NEG	NEG	NEG	NEG	NEG	NEG	NEG	NEG	NEG	NEG	NEG	NEG	NEG
289126	CC0022	CC22-MRSA-IV (PVL+)	POS	POS	POS	NEG	NEG	NEG	POS	POS	POS	NEG	NEG	NEG	NEG	NEG	NEG	NEG	NEG	NEG	NEG	NEG	NEG	NEG	NEG	NEG	NEG	NEG	NEG	NEG
289127	CC0022	CC22-MRSA-IV (PVL+)	POS	POS	POS	NEG	NEG	NEG	POS	POS	POS	NEG	NEG	NEG	NEG	NEG	NEG	NEG	NEG	NEG	NEG	NEG	NEG	NEG	NEG	NEG	NEG	NEG	NEG	NEG
131939	CC0025	CC25-MSSA	NEG	NEG	NEG	NEG	NEG	NEG	NEG	NEG	NEG	NEG	NEG	NEG	NEG	NEG	NEG	NEG	NEG	NEG	NEG	NEG	NEG	NEG	NEG	NEG	NEG	NEG	0	0
275165	CC0030	CC30-MSSA (lukF-P83/lukM+)	NEG	NEG	NEG	NEG	NEG	NEG	NEG	NEG	NEG	NEG	NEG	NEG	NEG	NEG	NEG	NEG	NEG	NEG	NEG	NEG	NEG	NEG	NEG	NEG	NEG	NEG	NEG	NEG
275741	CC0030	CC30-MSSA	NEG	NEG	NEG	NEG	NEG	NEG	NEG	NEG	NEG	NEG	NEG	NEG	NEG	NEG	NEG	NEG	NEG	NEG	NEG	NEG	NEG	NEG	NEG	NEG	NEG	NEG	NEG	NEG
124492	CC0049	CC49-MSSA (lukF-P83/lukM+)	NEG	NEG	NEG	NEG	NEG	NEG	NEG	NEG	NEG	NEG	NEG	NEG	NEG	NEG	NEG	NEG	NEG	NEG	NEG	NEG	NEG	NEG	NEG	NEG	NEG	NEG	0	0
220616	CC0049	CC49-MSSA (lukF-P83/lukM+)	NEG	NEG	NEG	NEG	NEG	NEG	NEG	NEG	NEG	NEG	NEG	NEG	NEG	NEG	NEG	NEG	NEG	NEG	NEG	NEG	NEG	NEG	NEG	NEG	NEG	NEG	0	0
228649	CC0049	CC49-MSSA (lukF-P83/lukM+)	NEG	NEG	NEG	NEG	NEG	NEG	NEG	NEG	NEG	NEG	NEG	NEG	NEG	NEG	NEG	NEG	NEG	NEG	NEG	NEG	NEG	NEG	NEG	NEG	NEG	NEG	NEG	NEG
190994	CC0059	CC59-MSSA	AMB	NEG	NEG	NEG	NEG	NEG	NEG	NEG	NEG	NEG	NEG	NEG	NEG	NEG	NEG	NEG	NEG	NEG	NEG	NEG	NEG	NEG	NEG	NEG	NEG	NEG	0	0
275172	CC0088	CC88-MSSA	NEG	NEG	NEG	NEG	NEG	NEG	NEG	NEG	NEG	NEG	NEG	NEG	NEG	NEG	NEG	NEG	NEG	NEG	NEG	NEG	NEG	NEG	NEG	NEG	NEG	NEG	NEG	NEG
275173	CC0088	CC88-MSSA	NEG	NEG	NEG	NEG	NEG	NEG	NEG	NEG	NEG	NEG	NEG	NEG	NEG	NEG	NEG	NEG	NEG	NEG	NEG	NEG	NEG	NEG	NEG	NEG	NEG	NEG	NEG	NEG
275174	CC0088	CC88-MSSA	NEG	NEG	NEG	NEG	NEG	NEG	NEG	NEG	NEG	NEG	NEG	NEG	NEG	NEG	NEG	NEG	NEG	NEG	NEG	NEG	NEG	NEG	NEG	NEG	NEG	NEG	NEG	NEG
170971	CC0097	CC97-MSSA	NEG	NEG	NEG	NEG	NEG	NEG	NEG	NEG	NEG	NEG	NEG	NEG	NEG	NEG	NEG	NEG	NEG	NEG	NEG	NEG	NEG	NEG	NEG	NEG	NEG	NEG	0	0
112745	CC0097	CC97-MSSA	NEG	NEG	NEG	NEG	NEG	NEG	NEG	NEG	NEG	NEG	NEG	NEG	NEG	NEG	NEG	NEG	NEG	NEG	NEG	NEG	NEG	NEG	NEG	NEG	NEG	NEG	0	0
112748	CC0097	CC97-MSSA	NEG	NEG	NEG	NEG	NEG	NEG	NEG	NEG	NEG	NEG	NEG	NEG	NEG	NEG	NEG	NEG	NEG	NEG	NEG	NEG	NEG	NEG	NEG	NEG	NEG	NEG	0	0
112750	CC0097	CC97-MSSA	NEG	NEG	NEG	NEG	NEG	NEG	NEG	NEG	NEG	NEG	NEG	NEG	NEG	NEG	NEG	NEG	NEG	NEG	NEG	NEG	NEG	NEG	NEG	NEG	NEG	NEG	0	0
112762	CC0097	CC97-MSSA	NEG	NEG	NEG	NEG	NEG	NEG	NEG	NEG	NEG	NEG	NEG	NEG	NEG	NEG	NEG	NEG	NEG	NEG	NEG	NEG	NEG	NEG	NEG	NEG	NEG	NEG	0	0
112763	CC0097	CC97-MSSA	NEG	NEG	NEG	NEG	NEG	NEG	NEG	NEG	NEG	NEG	NEG	NEG	NEG	NEG	NEG	NEG	NEG	NEG	NEG	NEG	NEG	NEG	NEG	NEG	NEG	NEG	0	0
112764	CC0097	CC97-MSSA	NEG	NEG	NEG	NEG	NEG	NEG	NEG	NEG	NEG	NEG	NEG	NEG	NEG	NEG	NEG	NEG	NEG	NEG	NEG	NEG	NEG	NEG	NEG	NEG	NEG	NEG	0	0
170945	CC0097	CC97-MSSA	NEG	NEG	NEG	NEG	NEG	NEG	NEG	NEG	NEG	NEG	NEG	NEG	NEG	NEG	NEG	NEG	NEG	NEG	NEG	NEG	NEG	NEG	NEG	NEG	NEG	NEG	0	0
170954	CC0097	CC97-MSSA	NEG	NEG	NEG	NEG	NEG	NEG	NEG	NEG	NEG	NEG	NEG	NEG	NEG	NEG	NEG	NEG	NEG	NEG	NEG	NEG	NEG	NEG	NEG	NEG	NEG	NEG	0	0
170958	CC0097	CC97-MSSA	NEG	NEG	NEG	NEG	NEG	NEG	NEG	NEG	NEG	NEG	NEG	NEG	NEG	NEG	NEG	NEG	NEG	NEG	NEG	NEG	NEG	NEG	NEG	NEG	NEG	NEG	0	0
170963	CC0097	CC97-MSSA	NEG	NEG	NEG	NEG	NEG	NEG	NEG	NEG	NEG	NEG	NEG	NEG	NEG	NEG	NEG	NEG	NEG	NEG	NEG	NEG	NEG	NEG	NEG	NEG	NEG	NEG	0	0
170964	CC0097	CC97-MSSA	NEG	NEG	NEG	NEG	NEG	NEG	NEG	NEG	NEG	NEG	NEG	NEG	NEG	NEG	NEG	NEG	NEG	NEG	NEG	NEG	NEG	NEG	NEG	NEG	NEG	NEG	0	0
170965	CC0097	CC97-MSSA	NEG	NEG	NEG	NEG	NEG	NEG	NEG	NEG	NEG	NEG	NEG	NEG	NEG	NEG	NEG	NEG	NEG	NEG	NEG	NEG	NEG	NEG	NEG	NEG	NEG	NEG	0	0
170966	CC0097	CC97-MSSA	NEG	NEG	NEG	NEG	NEG	NEG	NEG	NEG	NEG	NEG	NEG	NEG	NEG	NEG	NEG	NEG	NEG	NEG	NEG	NEG	NEG	NEG	NEG	NEG	NEG	NEG	0	0
170974	CC0097	CC97-MSSA	NEG	NEG	NEG	NEG	NEG	NEG	NEG	NEG	NEG	NEG	NEG	NEG	NEG	NEG	NEG	NEG	NEG	NEG	NEG	NEG	NEG	NEG	NEG	NEG	NEG	NEG	0	0
170976	CC0097	CC97-MSSA	NEG	NEG	NEG	NEG	NEG	NEG	NEG	NEG	NEG	NEG	NEG	NEG	NEG	NEG	NEG	NEG	NEG	NEG	NEG	NEG	NEG	NEG	NEG	NEG	NEG	NEG	0	0
112744	CC0097	CC97-MSSA	NEG	NEG	NEG	NEG	NEG	NEG	NEG	NEG	NEG	NEG	NEG	NEG	NEG	NEG	NEG	NEG	NEG	NEG	NEG	NEG	NEG	NEG	NEG	NEG	NEG	NEG	0	0
112749	CC0097	CC97-MSSA	NEG	NEG	NEG	NEG	NEG	NEG	NEG	NEG	NEG	NEG	NEG	NEG	NEG	NEG	NEG	NEG	NEG	NEG	NEG	NEG	NEG	NEG	NEG	NEG	NEG	NEG	0	0
112757	CC0097	CC97-MSSA	NEG	NEG	NEG	NEG	NEG	NEG	NEG	NEG	NEG	NEG	NEG	NEG	NEG	NEG	NEG	NEG	NEG	NEG	NEG	NEG	NEG	NEG	NEG	NEG	NEG	NEG	0	0
170973	CC0097	CC97-MSSA	NEG	NEG	NEG	NEG	NEG	NEG	NEG	NEG	NEG	NEG	NEG	NEG	NEG	NEG	NEG	NEG	NEG	NEG	NEG	NEG	NEG	NEG	NEG	NEG	NEG	NEG	0	0
207756	CC0097	CC97-MSSA	NEG	NEG	NEG	NEG	NEG	NEG	NEG	NEG	NEG	NEG	NEG	NEG	NEG	NEG	NEG	NEG	NEG	NEG	NEG	NEG	NEG	NEG	NEG	NEG	NEG	NEG	0	0
206171	CC0097	CC97-MSSA	NEG	NEG	NEG	NEG	NEG	NEG	NEG	NEG	NEG	NEG	NEG	NEG	NEG	NEG	NEG	NEG	NEG	NEG	NEG	NEG	NEG	NEG	NEG	NEG	NEG	NEG	0	0
275169	CC0097	CC97-MSSA-[ccrAA]	NEG	NEG	NEG	NEG	NEG	NEG	NEG	NEG	NEG	NEG	NEG	NEG	NEG	NEG	NEG	NEG	NEG	NEG	NEG	NEG	NEG	NEG	POS	NEG	NEG	NEG	NEG	NEG
275168	CC0097	CC97-MSSA-[ccrAA]	NEG	NEG	NEG	NEG	NEG	NEG	NEG	NEG	NEG	NEG	NEG	NEG	NEG	NEG	NEG	NEG	NEG	NEG	NEG	NEG	NEG	NEG	POS	NEG	NEG	NEG	NEG	NEG
118320	CC0130	CC130-MSSA (lukF-P83/M+)	NEG	NEG	NEG	NEG	NEG	NEG	NEG	NEG	NEG	NEG	NEG	NEG	NEG	NEG	NEG	NEG	NEG	NEG	NEG	NEG	NEG	NEG	NEG	NEG	NEG	NEG	NEG	NEG
203236	CC0130	CC130-MRSA-XI	NEG	NEG	NEG	NEG	AMB	NEG	NEG	NEG	NEG	NEG	NEG	NEG	NEG	NEG	NEG	NEG	AMB	AMB	AMB	NEG	NEG	NEG	NEG	NEG	NEG	NEG	POS	POS
204204	CC0130	CC130-MRSA-XI	NEG	NEG	NEG	NEG	NEG	NEG	NEG	NEG	NEG	NEG	NEG	NEG	NEG	NEG	NEG	NEG	NEG	NEG	NEG	NEG	NEG	NEG	NEG	NEG	NEG	NEG	POS	POS
289122	CC0130	CC130-MRSA-XI	NEG	NEG	NEG	NEG	NEG	NEG	NEG	NEG	NEG	NEG	NEG	NEG	NEG	NEG	NEG	NEG	NEG	NEG	NEG	NEG	NEG	NEG	NEG	NEG	NEG	NEG	POS	POS
225779	CC0130	CC130-MRSA-XI	NEG	NEG	NEG	NEG	NEG	NEG	NEG	NEG	NEG	NEG	NEG	NEG	NEG	NEG	NEG	NEG	NEG	NEG	NEG	NEG	NEG	NEG	NEG	NEG	NEG	NEG	POS	POS
225780	CC0130	CC130-MRSA-XI	NEG	NEG	NEG	NEG	NEG	NEG	NEG	NEG	NEG	NEG	NEG	NEG	NEG	NEG	NEG	NEG	NEG	NEG	NEG	NEG	NEG	NEG	NEG	NEG	NEG	NEG	POS	POS
170959	CC0130	CC130-MRSA-XI	NEG	NEG	NEG	NEG	NEG	NEG	NEG	NEG	NEG	NEG	NEG	NEG	NEG	NEG	NEG	NEG	NEG	NEG	NEG	NEG	NEG	NEG	NEG	NEG	NEG	NEG	POS	POS
179029	CC0130	CC130-MRSA-XI	NEG	NEG	NEG	NEG	AMB	NEG	NEG	NEG	NEG	NEG	NEG	NEG	NEG	NEG	NEG	NEG	0	NEG	NEG	NEG	NEG	NEG	NEG	NEG	NEG	NEG	POS	POS
116254	CC0133	CC133-MSSA	NEG	NEG	NEG	NEG	NEG	NEG	NEG	NEG	NEG	NEG	NEG	NEG	NEG	NEG	NEG	NEG	NEG	NEG	NEG	NEG	NEG	NEG	NEG	NEG	NEG	NEG	0	0
207761	CC0133	CC133-MSSA	NEG	NEG	NEG	NEG	NEG	NEG	NEG	NEG	NEG	NEG	NEG	NEG	NEG	NEG	NEG	NEG	NEG	NEG	NEG	NEG	NEG	NEG	NEG	NEG	NEG	NEG	0	0
213103	CC0133	CC133-MSSA	NEG	NEG	NEG	NEG	NEG	NEG	NEG	NEG	NEG	NEG	NEG	NEG	NEG	NEG	NEG	AMB	NEG	NEG	NEG	NEG	NEG	NEG	NEG	NEG	NEG	NEG	0	0
275736	CC0133	CC133-MSSA	NEG	NEG	NEG	NEG	NEG	NEG	NEG	NEG	NEG	NEG	NEG	NEG	NEG	NEG	NEG	NEG	NEG	NEG	NEG	NEG	NEG	NEG	NEG	NEG	NEG	NEG	NEG	NEG
275737	CC0133	CC133-MSSA	NEG	NEG	NEG	NEG	NEG	NEG	NEG	NEG	NEG	NEG	NEG	NEG	NEG	NEG	NEG	NEG	NEG	NEG	NEG	NEG	NEG	NEG	NEG	NEG	NEG	NEG	NEG	NEG
275738	CC0133	CC133-MSSA	NEG	NEG	NEG	NEG	NEG	NEG	NEG	NEG	NEG	NEG	NEG	NEG	NEG	NEG	NEG	NEG	NEG	NEG	NEG	NEG	NEG	NEG	NEG	NEG	NEG	NEG	NEG	NEG
275739	CC0133	CC133-MSSA	NEG	NEG	NEG	NEG	NEG	NEG	NEG	NEG	NEG	NEG	NEG	NEG	NEG	NEG	NEG	NEG	NEG	NEG	NEG	NEG	NEG	NEG	NEG	NEG	NEG	NEG	NEG	NEG
112755	CC0133	CC133-MSSA	NEG	NEG	NEG	NEG	NEG	NEG	NEG	NEG	NEG	NEG	NEG	NEG	NEG	NEG	NEG	NEG	NEG	NEG	NEG	NEG	NEG	NEG	NEG	NEG	NEG	NEG	NEG	NEG
225778	CC0398	CC398-MRSA-V/VT	POS	NEG	POS	NEG	NEG	NEG	NEG	NEG	NEG	NEG	NEG	NEG	NEG	NEG	NEG	NEG	NEG	NEG	NEG	NEG	NEG	POS	POS	POS	NEG	NEG	NEG	NEG
123159	ST0425	ST425-MSSA	NEG	NEG	NEG	NEG	NEG	NEG	NEG	NEG	NEG	NEG	NEG	NEG	NEG	NEG	NEG	NEG	NEG	NEG	NEG	NEG	NEG	NEG	NEG	NEG	NEG	NEG	NEG	NEG
86991	ST0425	ST425-MSSA	NEG	NEG	NEG	NEG	NEG	NEG	NEG	NEG	NEG	NEG	NEG	NEG	NEG	NEG	NEG	NEG	NEG	NEG	NEG	NEG	NEG	NEG	NEG	NEG	NEG	NEG	NEG	NEG
112756	ST0425	ST425-MSSA	NEG	NEG	NEG	NEG	NEG	NEG	NEG	NEG	NEG	NEG	NEG	NEG	NEG	NEG	NEG	NEG	NEG	NEG	NEG	NEG	NEG	NEG	NEG	NEG	NEG	NEG	NEG	NEG
195207	ST0425	ST425-MSSA	NEG	NEG	NEG	NEG	NEG	NEG	NEG	NEG	NEG	NEG	NEG	NEG	NEG	NEG	NEG	NEG	NEG	NEG	NEG	NEG	NEG	NEG	NEG	NEG	NEG	NEG	NEG	0
223609	ST0425	ST425-MSSA	NEG	NEG	NEG	NEG	NEG	NEG	NEG	NEG	NEG	NEG	NEG	NEG	NEG	NEG	NEG	NEG	NEG	NEG	NEG	NEG	NEG	NEG	NEG	NEG	NEG	NEG	NEG	NEG
275740	ST0425	ST425-MSSA	NEG	NEG	NEG	NEG	NEG	NEG	NEG	NEG	NEG	NEG	NEG	NEG	NEG	NEG	NEG	NEG	NEG	NEG	NEG	NEG	NEG	NEG	NEG	NEG	NEG	NEG	NEG	NEG
174889	ST0425	ST425-MSSA	NEG	NEG	NEG	NEG	NEG	NEG	NEG	NEG	NEG	NEG	NEG	NEG	NEG	NEG	NEG	NEG	NEG	NEG	NEG	NEG	NEG	NEG	NEG	NEG	NEG	NEG	NEG	NEG
203230	ST0425	ST425-MSSA	NEG	NEG	NEG	NEG	AMB	NEG	NEG	NEG	NEG	NEG	NEG	NEG	NEG	NEG	NEG	AMB	NEG	NEG	NEG	NEG	NEG	NEG	NEG	NEG	NEG	NEG	0	0
203242	ST0425	ST425-MSSA	NEG	NEG	NEG	NEG	AMB	NEG	NEG	NEG	NEG	NEG	NEG	NEG	NEG	NEG	NEG	NEG	NEG	NEG	NEG	NEG	NEG	NEG	NEG	NEG	NEG	NEG	0	0
203243	ST0425	ST425-MSSA	NEG	NEG	NEG	NEG	AMB	NEG	NEG	NEG	NEG	NEG	NEG	NEG	NEG	NEG	NEG	AMB	AMB	NEG	NEG	NEG	NEG	NEG	NEG	NEG	NEG	NEG	0	0
207951	ST0425	ST425-MSSA	NEG	NEG	NEG	NEG	NEG	NEG	NEG	NEG	NEG	NEG	NEG	NEG	NEG	NEG	NEG	NEG	NEG	NEG	NEG	NEG	NEG	NEG	NEG	NEG	NEG	NEG	0	0
207981	ST0425	ST425-MSSA	NEG	NEG	NEG	NEG	NEG	NEG	NEG	NEG	NEG	NEG	NEG	NEG	NEG	NEG	NEG	NEG	NEG	NEG	NEG	NEG	NEG	NEG	NEG	NEG	NEG	NEG	0	0
195212	ST0425	ST425-MSSA	NEG	NEG	NEG	NEG	NEG	NEG	NEG	NEG	NEG	NEG	NEG	NEG	NEG	NEG	NEG	NEG	NEG	NEG	NEG	NEG	NEG	NEG	NEG	NEG	NEG	NEG	NEG	0
195206	ST0425	ST425-MSSA	NEG	NEG	NEG	NEG	NEG	NEG	NEG	NEG	NEG	NEG	NEG	NEG	NEG	NEG	NEG	AMB	NEG	NEG	NEG	NEG	NEG	NEG	NEG	NEG	NEG	NEG	NEG	0
195202	ST0425	ST425-MSSA	NEG	NEG	NEG	NEG	NEG	NEG	NEG	NEG	NEG	NEG	NEG	NEG	NEG	NEG	NEG	NEG	NEG	NEG	NEG	NEG	NEG	NEG	NEG	NEG	NEG	NEG	0	0
216042	ST0425	ST425-MSSA	NEG	NEG	NEG	NEG	NEG	NEG	NEG	NEG	NEG	NEG	NEG	NEG	NEG	NEG	NEG	NEG	NEG	NEG	NEG	NEG	NEG	NEG	NEG	NEG	NEG	NEG	NEG	NEG
223608	ST0425	ST425-MSSA	NEG	NEG	NEG	NEG	NEG	NEG	NEG	NEG	NEG	NEG	NEG	NEG	NEG	NEG	NEG	NEG	NEG	NEG	NEG	NEG	NEG	NEG	NEG	NEG	NEG	NEG	NEG	NEG
223612	ST0425	ST425-MSSA	NEG	NEG	NEG	NEG	NEG	NEG	NEG	NEG	NEG	NEG	NEG	NEG	NEG	NEG	NEG	NEG	NEG	NEG	NEG	NEG	NEG	NEG	NEG	NEG	NEG	NEG	NEG	NEG
225733	ST0425	ST425-MSSA	NEG	NEG	NEG	NEG	NEG	NEG	NEG	NEG	NEG	NEG	NEG	NEG	NEG	NEG	NEG	NEG	NEG	NEG	NEG	NEG	NEG	NEG	NEG	NEG	NEG	NEG	0	0
230321	ST0425	ST425-MSSA	NEG	NEG	NEG	NEG	NEG	NEG	NEG	NEG	NEG	NEG	NEG	NEG	NEG	NEG	NEG	NEG	NEG	NEG	NEG	NEG	NEG	NEG	NEG	NEG	NEG	NEG	NEG	NEG
232368	ST0425	ST425-MSSA	NEG	NEG	NEG	NEG	NEG	NEG	NEG	NEG	NEG	NEG	NEG	NEG	NEG	NEG	NEG	NEG	NEG	NEG	NEG	NEG	NEG	NEG	NEG	NEG	NEG	NEG	NEG	NEG
204215	ST0425	ST425-MSSA	NEG	NEG	NEG	NEG	NEG	NEG	NEG	NEG	NEG	NEG	NEG	NEG	NEG	NEG	NEG	NEG	NEG	NEG	NEG	NEG	NEG	NEG	NEG	NEG	NEG	NEG	0	0
204218	ST0425	ST425-MSSA	NEG	NEG	NEG	NEG	NEG	NEG	NEG	NEG	NEG	NEG	NEG	NEG	NEG	NEG	NEG	NEG	NEG	NEG	NEG	NEG	NEG	NEG	NEG	NEG	NEG	NEG	0	0
204222	ST0425	ST425-MSSA	NEG	NEG	NEG	NEG	NEG	NEG	NEG	NEG	NEG	NEG	NEG	NEG	NEG	NEG	NEG	NEG	NEG	NEG	NEG	NEG	NEG	NEG	NEG	NEG	NEG	NEG	0	0
204226	ST0425	ST425-MSSA	NEG	NEG	NEG	NEG	NEG	NEG	NEG	NEG	NEG	NEG	NEG	NEG	NEG	NEG	NEG	NEG	NEG	NEG	NEG	NEG	NEG	NEG	NEG	NEG	NEG	NEG	0	0
204906	ST0425	ST425-MSSA	NEG	NEG	NEG	NEG	NEG	NEG	NEG	NEG	NEG	NEG	NEG	NEG	NEG	NEG	NEG	NEG	NEG	NEG	NEG	NEG	NEG	NEG	NEG	NEG	NEG	NEG	0	0
204908	ST0425	ST425-MSSA	NEG	NEG	NEG	NEG	NEG	NEG	NEG	NEG	NEG	NEG	NEG	NEG	NEG	NEG	NEG	AMB	NEG	NEG	NEG	NEG	NEG	NEG	NEG	NEG	NEG	NEG	0	0
206170	ST0425	ST425-MSSA	NEG	NEG	NEG	NEG	NEG	NEG	NEG	NEG	NEG	NEG	NEG	NEG	NEG	NEG	NEG	NEG	NEG	NEG	NEG	NEG	NEG	NEG	NEG	NEG	NEG	NEG	0	0
207765	ST0425	ST425-MSSA	NEG	NEG	NEG	NEG	NEG	NEG	NEG	NEG	NEG	NEG	NEG	NEG	NEG	NEG	NEG	NEG	NEG	NEG	NEG	NEG	NEG	NEG	NEG	NEG	NEG	NEG	0	0
240048	CC599	CC599-MRSA-XI	NEG	NEG	NEG	NEG	NEG	NEG	NEG	NEG	NEG	NEG	NEG	NEG	NEG	NEG	NEG	NEG	NEG	NEG	NEG	NEG	NEG	NEG	NEG	NEG	NEG	NEG	POS	POS
225730	CC0692	CC692-MSSA	NEG	NEG	NEG	NEG	NEG	NEG	NEG	NEG	NEG	NEG	NEG	NEG	NEG	NEG	NEG	NEG	NEG	NEG	NEG	NEG	NEG	NEG	NEG	NEG	NEG	NEG	NEG	NEG
112746	CC0692	CC692-MSSA	NEG	NEG	NEG	NEG	NEG	NEG	NEG	NEG	NEG	NEG	NEG	NEG	NEG	NEG	NEG	NEG	NEG	NEG	NEG	NEG	NEG	NEG	NEG	NEG	NEG	NEG	0	0
112754	CC0692	CC692-MSSA	NEG	NEG	NEG	NEG	NEG	NEG	NEG	NEG	NEG	NEG	NEG	NEG	NEG	NEG	NEG	NEG	NEG	NEG	NEG	NEG	NEG	NEG	NEG	NEG	NEG	NEG	NEG	NEG
112758	CC0692	CC692-MSSA	NEG	NEG	NEG	NEG	NEG	NEG	NEG	NEG	NEG	NEG	NEG	NEG	NEG	NEG	NEG	NEG	NEG	NEG	NEG	NEG	NEG	NEG	NEG	NEG	NEG	NEG	0	0
112759	CC0692	CC692-MSSA	NEG	NEG	NEG	NEG	NEG	NEG	NEG	NEG	NEG	NEG	NEG	NEG	NEG	NEG	NEG	NEG	NEG	NEG	NEG	NEG	NEG	NEG	NEG	NEG	NEG	NEG	0	0
124745	CC0692	CC692-MSSA	NEG	NEG	NEG	NEG	NEG	NEG	NEG	NEG	NEG	NEG	NEG	NEG	NEG	NEG	NEG	NEG	NEG	NEG	NEG	NEG	NEG	NEG	NEG	NEG	NEG	NEG	NEG	NEG
170952	CC0692	CC692-MSSA	NEG	NEG	NEG	NEG	NEG	NEG	NEG	NEG	NEG	NEG	NEG	NEG	NEG	NEG	NEG	NEG	NEG	NEG	NEG	NEG	NEG	NEG	NEG	NEG	NEG	NEG	0	0
170953	CC0692	CC692-MSSA	NEG	NEG	NEG	NEG	NEG	NEG	NEG	NEG	NEG	NEG	NEG	NEG	NEG	NEG	NEG	NEG	NEG	NEG	NEG	NEG	NEG	NEG	NEG	NEG	NEG	NEG	0	0
170970	CC0692	CC692-MSSA	NEG	NEG	NEG	NEG	NEG	NEG	NEG	NEG	NEG	NEG	NEG	NEG	NEG	NEG	NEG	NEG	NEG	NEG	NEG	NEG	NEG	NEG	NEG	NEG	NEG	NEG	0	0
170950	CC0707	CC707-MSSA	NEG	NEG	NEG	NEG	NEG	NEG	NEG	NEG	NEG	NEG	NEG	NEG	NEG	NEG	NEG	NEG	NEG	NEG	NEG	NEG	NEG	NEG	NEG	NEG	NEG	NEG	0	0
124948	ST0890	ST890-MSSA	NEG	NEG	NEG	NEG	NEG	NEG	NEG	NEG	NEG	NEG	NEG	NEG	NEG	NEG	NEG	NEG	NEG	NEG	NEG	NEG	NEG	NEG	NEG	NEG	NEG	NEG	NEG	0
220618	CC1956	CC1956-MSSA	NEG	NEG	NEG	NEG	NEG	NEG	NEG	NEG	NEG	NEG	NEG	NEG	NEG	NEG	NEG	NEG	NEG	NEG	NEG	NEG	NEG	NEG	NEG	NEG	NEG	NEG	NEG	NEG
112742	ST2425	ST2425-MSSA	NEG	NEG	NEG	NEG	NEG	NEG	NEG	NEG	NEG	NEG	NEG	NEG	NEG	NEG	NEG	NEG	NEG	NEG	NEG	NEG	NEG	NEG	NEG	NEG	NEG	NEG	NEG	NEG
170957	ST2425	ST2425-MSSA	NEG	NEG	NEG	NEG	NEG	NEG	NEG	NEG	NEG	NEG	NEG	NEG	NEG	NEG	NEG	NEG	NEG	NEG	NEG	NEG	NEG	NEG	NEG	NEG	NEG	NEG	0	0
170947	ST2691	ST2691-MSSA	NEG	NEG	NEG	NEG	NEG	NEG	NEG	NEG	NEG	NEG	NEG	NEG	NEG	NEG	NEG	NEG	NEG	NEG	NEG	NEG	NEG	NEG	NEG	NEG	NEG	NEG	NEG	NEG
170961	ST2691	ST2691-MSSA	NEG	NEG	NEG	NEG	NEG	NEG	NEG	NEG	NEG	NEG	NEG	NEG	NEG	NEG	NEG	NEG	NEG	NEG	NEG	NEG	NEG	NEG	NEG	NEG	NEG	NEG	NEG	NEG
112753	CC2767	ST2279-MSSA	NEG	NEG	NEG	NEG	NEG	NEG	NEG	NEG	NEG	NEG	NEG	NEG	NEG	NEG	NEG	NEG	NEG	NEG	NEG	NEG	NEG	NEG	NEG	NEG	NEG	NEG	NEG	NEG
112761	CC2767	ST2279-MSSA	NEG	NEG	NEG	NEG	NEG	NEG	NEG	NEG	NEG	NEG	NEG	NEG	NEG	NEG	NEG	NEG	NEG	NEG	NEG	NEG	NEG	NEG	NEG	NEG	NEG	NEG	NEG	NEG
228651	ST2963	ST2963-MSSA	NEG	NEG	NEG	NEG	NEG	NEG	NEG	NEG	NEG	NEG	NEG	NEG	NEG	NEG	NEG	NEG	NEG	NEG	NEG	NEG	NEG	NEG	NEG	NEG	NEG	NEG	NEG	NEG
203244	ST3237	ST3237-MSSA	NEG	NEG	NEG	NEG	NEG	NEG	NEG	NEG	NEG	NEG	NEG	NEG	NEG	NEG	NEG	NEG	NEG	NEG	NEG	NEG	NEG	NEG	NEG	NEG	NEG	NEG	NEG	NEG

ISOLATE ID	CLONAL COMPLEX (by array)	STRAIN AFFILIATION (by array)	beta-lactamase	beta lactamase repressor (inhibitor)	beta-lactamase regulatory protein	rRNA adenine N-6-methyl-transferase, erythromycin/clindamycin resistance	erythro-mycin/clindamycin resistance	erythro-mycin/clindamycin resistance	Linco-samid-Nucleo-tidyltrans-ferase	energy-dependent efflux of erythro-mycin	macrolide efflux protein A	probable lysylphos-phatidyl-glycerol synthetase	virginia-mycin A acetyltransferase	acetyl-transferase inactivating streptogramin A	ATP binding protein, streptogramin-A-resistance		virginiamycin B hydrolase	bifunctional enzyme Aac/Aph, gentamicin resistance	amino-glycoside adenyl-transferase,tobramycin resistance	3'5'-aminog-lycoside phospho-transferase, neo-/ kanamycin resistance	strepto-thricine-acetyl-transferase	dihydro-folate reductase type 1	fusidic acid resistence	hypothetical protein associated with fusidic acid resistance	mupirocin resistence protein	tetrazyklin-resistance	tetracycline-resistance
			PENICILLINASE			RESISTANCE : MLS-ANTIBIOTICS												RESISTANCE : AMINOGLYSOSIDES				RESISTANCE : MISCELLANEOUS GENES					
			blaZ	blaI	blaR	ermA	ermB	ermC	linA	msrA	mefA	mpbBM	vatA	vatB	vga	vgaA	vgb	aacA-aphD	aadD	aphA3	sat	dfrA	far1	Q6GD50 (fusC)	mupR	tetK	tetM
203228	CC0001	CC1-MSSA	NEG	NEG	NEG	NEG	NEG	NEG	NEG	NEG	NEG	NEG	NEG	NEG	AMB	NEG	NEG	NEG	NEG	NEG	NEG	NEG	NEG	NEG	NEG	NEG	NEG
203229	CC0001	CC1-MSSA	NEG	NEG	NEG	NEG	NEG	NEG	NEG	NEG	NEG	NEG	NEG	NEG	AMB	NEG	NEG	NEG	NEG	NEG	NEG	AMB	NEG	NEG	NEG	NEG	NEG
203233	CC0001	CC1-MSSA	NEG	NEG	NEG	NEG	NEG	NEG	NEG	NEG	NEG	NEG	NEG	NEG	NEG	NEG	NEG	NEG	NEG	NEG	NEG	NEG	NEG	NEG	NEG	NEG	NEG
220334	CC0001	CC1-MSSA	NEG	NEG	NEG	NEG	NEG	NEG	NEG	NEG	NEG	NEG	NEG	NEG	NEG	NEG	NEG	NEG	NEG	NEG	NEG	NEG	NEG	NEG	NEG	NEG	NEG
223621	CC0001	CC1-MSSA	NEG	NEG	NEG	NEG	NEG	NEG	NEG	NEG	NEG	NEG	NEG	NEG	NEG	NEG	NEG	NEG	NEG	NEG	NEG	NEG	NEG	NEG	NEG	NEG	NEG
289123	CC0001	CC1-MRSA-IV, WA MRSA-1/57	POS	POS	POS	NEG	NEG	NEG	NEG	NEG	NEG	NEG	NEG	NEG	NEG	NEG	NEG	NEG	NEG	POS	POS	NEG	NEG	NEG	NEG	POS	NEG
289124	CC0001	CC1-MRSA-IV, WA MRSA-1/57	POS	POS	POS	NEG	NEG	NEG	NEG	NEG	NEG	NEG	NEG	NEG	NEG	NEG	NEG	NEG	NEG	POS	POS	NEG	NEG	NEG	NEG	POS	NEG
275170	CC0005	CC5-MSSA	NEG	NEG	NEG	NEG	NEG	NEG	NEG	NEG	NEG	NEG	NEG	NEG	NEG	NEG	NEG	NEG	NEG	NEG	NEG	NEG	NEG	NEG	NEG	NEG	NEG
275171	CC0005	CC5-MSSA	NEG	NEG	NEG	NEG	NEG	NEG	NEG	NEG	NEG	NEG	NEG	NEG	NEG	NEG	NEG	NEG	NEG	NEG	NEG	NEG	NEG	NEG	NEG	NEG	NEG
112747	CC0005	CC5-MSSA	NEG	NEG	NEG	NEG	NEG	NEG	NEG	NEG	NEG	NEG	NEG	NEG	NEG	NEG	NEG	NEG	NEG	NEG	NEG	NEG	NEG	NEG	NEG	NEG	NEG
112752	CC0006	CC6-MSSA	POS	POS	POS	NEG	NEG	NEG	NEG	NEG	NEG	NEG	NEG	NEG	NEG	NEG	NEG	NEG	NEG	NEG	NEG	NEG	NEG	NEG	NEG	NEG	NEG
275178	CC0007	CC7-MSSA	POS	POS	POS	POS	NEG	NEG	NEG	NEG	NEG	NEG	NEG	NEG	NEG	NEG	NEG	NEG	NEG	NEG	NEG	NEG	NEG	NEG	NEG	NEG	NEG
275166	CC0008	CC8-MSSA	POS	POS	POS	NEG	NEG	NEG	NEG	NEG	NEG	NEG	NEG	NEG	NEG	NEG	NEG	NEG	NEG	NEG	NEG	NEG	NEG	NEG	NEG	NEG	NEG
275176	CC0008	CC8-MSSA	POS	POS	POS	NEG	NEG	NEG	NEG	NEG	NEG	NEG	NEG	NEG	NEG	NEG	NEG	NEG	NEG	NEG	NEG	NEG	NEG	NEG	NEG	NEG	NEG
275177	CC0008	CC8-MSSA	NEG	NEG	NEG	NEG	NEG	NEG	NEG	NEG	NEG	NEG	NEG	NEG	NEG	NEG	NEG	NEG	NEG	NEG	NEG	NEG	NEG	NEG	NEG	NEG	NEG
275167	CC0009	CC9-MSSA	NEG	NEG	NEG	NEG	NEG	NEG	NEG	NEG	NEG	NEG	NEG	NEG	NEG	NEG	NEG	NEG	NEG	NEG	NEG	NEG	NEG	NEG	NEG	POS	NEG
170968	CC0012	CC12-MSSA	POS	POS	POS	NEG	NEG	NEG	NEG	NEG	NEG	NEG	NEG	NEG	NEG	NEG	NEG	NEG	NEG	NEG	NEG	NEG	NEG	NEG	NEG	NEG	NEG
275175	CC0015	CC15-MSSA	POS	POS	POS	NEG	NEG	NEG	NEG	NEG	NEG	NEG	NEG	NEG	NEG	NEG	NEG	NEG	NEG	NEG	NEG	NEG	NEG	NEG	NEG	NEG	NEG
170949	CC0015	CC15-MSSA	POS	POS	POS	NEG	NEG	NEG	NEG	NEG	NEG	NEG	NEG	NEG	NEG	NEG	NEG	NEG	NEG	NEG	NEG	NEG	NEG	NEG	NEG	NEG	NEG
228658	CC0022	CC22-MSSA	POS	POS	POS	NEG	NEG	NEG	NEG	NEG	NEG	NEG	NEG	NEG	NEG	NEG	NEG	NEG	NEG	NEG	NEG	NEG	NEG	NEG	NEG	NEG	NEG
289125	CC0022	CC22-MRSA-IV (PVL+)	POS	POS	POS	NEG	NEG	NEG	NEG	NEG	NEG	NEG	NEG	NEG	NEG	NEG	NEG	POS	POS	NEG	NEG	POS	NEG	NEG	NEG	NEG	NEG
289126	CC0022	CC22-MRSA-IV (PVL+)	POS	POS	POS	NEG	NEG	NEG	NEG	NEG	NEG	NEG	NEG	NEG	NEG	NEG	NEG	POS	POS	NEG	NEG	POS	NEG	NEG	NEG	NEG	NEG
289127	CC0022	CC22-MRSA-IV (PVL+)	POS	POS	POS	NEG	NEG	NEG	NEG	NEG	NEG	NEG	NEG	NEG	NEG	NEG	NEG	POS	POS	NEG	NEG	POS	NEG	NEG	NEG	NEG	NEG
131939	CC0025	CC25-MSSA	POS	POS	POS	NEG	NEG	NEG	NEG	NEG	NEG	NEG	NEG	NEG	NEG	NEG	NEG	NEG	NEG	NEG	NEG	NEG	NEG	NEG	NEG	NEG	NEG
275165	CC0030	CC30-MSSA (lukF-P83/lukM+)	NEG	NEG	NEG	NEG	NEG	NEG	NEG	NEG	NEG	NEG	NEG	NEG	NEG	NEG	NEG	NEG	NEG	NEG	NEG	NEG	NEG	NEG	NEG	NEG	NEG
275741	CC0030	CC30-MSSA	POS	POS	POS	NEG	NEG	NEG	NEG	NEG	NEG	NEG	NEG	NEG	NEG	NEG	NEG	NEG	NEG	NEG	NEG	NEG	NEG	NEG	NEG	NEG	NEG
124492	CC0049	CC49-MSSA (lukF-P83/lukM+)	NEG	NEG	NEG	NEG	NEG	NEG	NEG	NEG	NEG	NEG	NEG	NEG	NEG	NEG	NEG	NEG	NEG	NEG	NEG	NEG	NEG	NEG	NEG	NEG	NEG
220616	CC0049	CC49-MSSA (lukF-P83/lukM+)	NEG	NEG	NEG	NEG	NEG	NEG	NEG	NEG	NEG	NEG	NEG	NEG	NEG	NEG	NEG	NEG	NEG	NEG	NEG	NEG	NEG	NEG	NEG	NEG	NEG
228649	CC0049	CC49-MSSA (lukF-P83/lukM+)	NEG	NEG	NEG	NEG	NEG	NEG	NEG	NEG	NEG	NEG	NEG	NEG	NEG	NEG	NEG	NEG	NEG	NEG	NEG	NEG	NEG	NEG	NEG	NEG	NEG
190994	CC0059	CC59-MSSA	POS	POS	POS	NEG	NEG	POS	NEG	NEG	NEG	NEG	NEG	NEG	NEG	NEG	NEG	NEG	NEG	NEG	NEG	NEG	NEG	NEG	NEG	NEG	NEG
275172	CC0088	CC88-MSSA	POS	POS	POS	NEG	NEG	NEG	NEG	NEG	NEG	NEG	NEG	NEG	NEG	NEG	NEG	NEG	NEG	NEG	NEG	NEG	NEG	NEG	NEG	NEG	NEG
275173	CC0088	CC88-MSSA	POS	POS	POS	NEG	NEG	NEG	NEG	NEG	NEG	NEG	NEG	NEG	NEG	NEG	NEG	NEG	NEG	NEG	NEG	NEG	NEG	NEG	NEG	NEG	NEG
275174	CC0088	CC88-MSSA	POS	POS	POS	NEG	NEG	NEG	NEG	NEG	NEG	NEG	NEG	NEG	NEG	NEG	NEG	NEG	NEG	NEG	NEG	NEG	NEG	NEG	NEG	NEG	NEG
170971	CC0097	CC97-MSSA	NEG	NEG	NEG	NEG	NEG	NEG	NEG	NEG	NEG	NEG	NEG	NEG	NEG	NEG	NEG	NEG	NEG	NEG	NEG	NEG	NEG	NEG	NEG	NEG	NEG
112745	CC0097	CC97-MSSA	NEG	NEG	NEG	NEG	NEG	NEG	NEG	NEG	NEG	NEG	NEG	NEG	NEG	NEG	NEG	NEG	NEG	NEG	NEG	NEG	NEG	NEG	NEG	NEG	NEG
112748	CC0097	CC97-MSSA	NEG	NEG	NEG	NEG	NEG	NEG	NEG	NEG	NEG	NEG	NEG	NEG	NEG	NEG	NEG	NEG	NEG	NEG	NEG	NEG	NEG	NEG	NEG	NEG	NEG
112750	CC0097	CC97-MSSA	NEG	NEG	NEG	NEG	NEG	NEG	NEG	NEG	NEG	NEG	NEG	NEG	NEG	NEG	NEG	NEG	NEG	NEG	NEG	NEG	NEG	NEG	NEG	NEG	NEG
112762	CC0097	CC97-MSSA	NEG	NEG	NEG	NEG	NEG	NEG	NEG	NEG	NEG	NEG	NEG	NEG	NEG	NEG	NEG	NEG	NEG	NEG	NEG	NEG	NEG	NEG	NEG	NEG	NEG
112763	CC0097	CC97-MSSA	NEG	NEG	NEG	NEG	NEG	NEG	NEG	NEG	NEG	NEG	NEG	NEG	NEG	NEG	NEG	NEG	NEG	NEG	NEG	NEG	NEG	NEG	NEG	NEG	NEG
112764	CC0097	CC97-MSSA	NEG	NEG	NEG	NEG	NEG	NEG	NEG	NEG	NEG	NEG	NEG	NEG	NEG	NEG	NEG	NEG	NEG	NEG	NEG	NEG	NEG	NEG	NEG	NEG	NEG
170945	CC0097	CC97-MSSA	NEG	NEG	NEG	NEG	NEG	NEG	NEG	NEG	NEG	NEG	NEG	NEG	NEG	NEG	NEG	NEG	NEG	NEG	NEG	NEG	NEG	NEG	NEG	NEG	NEG
170954	CC0097	CC97-MSSA	NEG	NEG	NEG	NEG	NEG	NEG	NEG	NEG	NEG	NEG	NEG	NEG	NEG	NEG	NEG	NEG	NEG	NEG	NEG	NEG	NEG	NEG	NEG	NEG	NEG
170958	CC0097	CC97-MSSA	NEG	NEG	NEG	NEG	NEG	NEG	NEG	NEG	NEG	NEG	NEG	NEG	NEG	NEG	NEG	NEG	NEG	NEG	NEG	NEG	NEG	NEG	NEG	NEG	NEG
170963	CC0097	CC97-MSSA	NEG	NEG	NEG	NEG	NEG	NEG	NEG	NEG	NEG	NEG	NEG	NEG	NEG	NEG	NEG	NEG	NEG	NEG	NEG	NEG	NEG	NEG	NEG	NEG	NEG
170964	CC0097	CC97-MSSA	NEG	NEG	NEG	NEG	NEG	NEG	NEG	NEG	NEG	NEG	NEG	NEG	NEG	NEG	NEG	NEG	NEG	NEG	NEG	NEG	NEG	NEG	NEG	NEG	NEG
170965	CC0097	CC97-MSSA	NEG	NEG	NEG	NEG	NEG	NEG	NEG	NEG	NEG	NEG	NEG	NEG	NEG	NEG	NEG	NEG	NEG	NEG	NEG	NEG	NEG	NEG	NEG	NEG	NEG
170966	CC0097	CC97-MSSA	NEG	NEG	NEG	NEG	NEG	NEG	NEG	NEG	NEG	NEG	NEG	NEG	NEG	NEG	NEG	NEG	NEG	NEG	NEG	NEG	NEG	NEG	NEG	NEG	NEG
170974	CC0097	CC97-MSSA	NEG	NEG	NEG	NEG	NEG	NEG	NEG	NEG	NEG	NEG	NEG	NEG	NEG	NEG	NEG	NEG	NEG	NEG	NEG	NEG	NEG	NEG	NEG	NEG	NEG
170976	CC0097	CC97-MSSA	NEG	NEG	NEG	NEG	NEG	NEG	NEG	NEG	NEG	NEG	NEG	NEG	NEG	NEG	NEG	NEG	NEG	NEG	NEG	NEG	NEG	NEG	NEG	NEG	NEG
112744	CC0097	CC97-MSSA	NEG	NEG	NEG	NEG	NEG	NEG	NEG	NEG	NEG	NEG	NEG	NEG	NEG	NEG	NEG	NEG	NEG	NEG	NEG	NEG	NEG	NEG	NEG	NEG	NEG
112749	CC0097	CC97-MSSA	NEG	NEG	NEG	NEG	NEG	NEG	NEG	NEG	NEG	NEG	NEG	NEG	NEG	NEG	NEG	NEG	NEG	NEG	NEG	NEG	NEG	NEG	NEG	NEG	NEG
112757	CC0097	CC97-MSSA	NEG	NEG	NEG	NEG	NEG	NEG	NEG	NEG	NEG	NEG	NEG	NEG	NEG	NEG	NEG	NEG	NEG	NEG	NEG	NEG	NEG	NEG	NEG	NEG	NEG
170973	CC0097	CC97-MSSA	NEG	NEG	NEG	NEG	NEG	NEG	NEG	NEG	NEG	NEG	NEG	NEG	NEG	NEG	NEG	NEG	NEG	NEG	NEG	NEG	NEG	NEG	NEG	NEG	NEG
207756	CC0097	CC97-MSSA	NEG	NEG	NEG	NEG	NEG	NEG	NEG	NEG	NEG	NEG	NEG	NEG	NEG	NEG	NEG	NEG	NEG	NEG	NEG	NEG	NEG	NEG	NEG	NEG	NEG
206171	CC0097	CC97-MSSA	NEG	NEG	NEG	NEG	NEG	NEG	NEG	NEG	NEG	NEG	NEG	NEG	NEG	NEG	NEG	NEG	NEG	NEG	NEG	NEG	NEG	NEG	NEG	NEG	NEG
275169	CC0097	CC97-MSSA-[ccrAA]	POS	POS	POS	NEG	NEG	NEG	NEG	NEG	NEG	NEG	NEG	NEG	NEG	NEG	NEG	NEG	NEG	NEG	NEG	NEG	NEG	NEG	NEG	POS	NEG
275168	CC0097	CC97-MSSA-[ccrAA]	POS	POS	POS	NEG	NEG	NEG	NEG	NEG	NEG	NEG	NEG	NEG	NEG	NEG	NEG	NEG	NEG	NEG	NEG	NEG	NEG	NEG	NEG	POS	NEG
118320	CC0130	CC130-MSSA (lukF-P83/M+)	NEG	NEG	NEG	NEG	NEG	NEG	NEG	NEG	NEG	NEG	NEG	NEG	NEG	NEG	NEG	NEG	NEG	NEG	NEG	NEG	NEG	NEG	NEG	NEG	NEG
203236	CC0130	CC130-MRSA-XI	NEG	NEG	NEG	NEG	NEG	NEG	NEG	NEG	NEG	NEG	NEG	NEG	AMB	NEG	NEG	NEG	NEG	NEG	NEG	NEG	NEG	NEG	NEG	NEG	NEG
204204	CC0130	CC130-MRSA-XI	NEG	NEG	NEG	NEG	NEG	NEG	NEG	NEG	NEG	NEG	NEG	NEG	NEG	NEG	NEG	NEG	NEG	NEG	NEG	NEG	NEG	NEG	NEG	NEG	NEG
289122	CC0130	CC130-MRSA-XI	NEG	NEG	NEG	NEG	NEG	NEG	NEG	NEG	NEG	NEG	NEG	NEG	NEG	NEG	NEG	NEG	NEG	NEG	NEG	NEG	NEG	NEG	NEG	NEG	NEG
225779	CC0130	CC130-MRSA-XI	NEG	NEG	NEG	NEG	NEG	NEG	NEG	NEG	NEG	NEG	NEG	NEG	NEG	NEG	NEG	NEG	NEG	NEG	NEG	NEG	NEG	NEG	NEG	NEG	NEG
225780	CC0130	CC130-MRSA-XI	NEG	NEG	NEG	NEG	NEG	NEG	NEG	NEG	NEG	NEG	NEG	NEG	NEG	NEG	NEG	NEG	NEG	NEG	NEG	NEG	NEG	NEG	NEG	NEG	NEG
170959	CC0130	CC130-MRSA-XI	NEG	NEG	NEG	NEG	NEG	NEG	NEG	NEG	NEG	NEG	NEG	NEG	NEG	NEG	NEG	NEG	NEG	NEG	NEG	NEG	NEG	NEG	NEG	NEG	NEG
179029	CC0130	CC130-MRSA-XI	NEG	NEG	NEG	NEG	NEG	NEG	NEG	NEG	NEG	NEG	NEG	NEG	NEG	NEG	NEG	NEG	NEG	NEG	NEG	NEG	NEG	NEG	NEG	NEG	NEG
116254	CC0133	CC133-MSSA	NEG	NEG	NEG	NEG	NEG	NEG	NEG	NEG	NEG	NEG	NEG	NEG	NEG	NEG	NEG	NEG	NEG	NEG	NEG	NEG	NEG	NEG	NEG	NEG	NEG
207761	CC0133	CC133-MSSA	NEG	NEG	NEG	NEG	NEG	NEG	NEG	NEG	NEG	NEG	NEG	NEG	NEG	NEG	NEG	NEG	NEG	NEG	NEG	NEG	NEG	NEG	NEG	NEG	NEG
213103	CC0133	CC133-MSSA	NEG	NEG	NEG	NEG	NEG	NEG	NEG	NEG	NEG	NEG	NEG	NEG	NEG	NEG	NEG	NEG	NEG	NEG	NEG	NEG	NEG	NEG	NEG	NEG	NEG
275736	CC0133	CC133-MSSA	NEG	NEG	NEG	NEG	NEG	NEG	NEG	NEG	NEG	NEG	NEG	NEG	NEG	NEG	NEG	NEG	NEG	NEG	NEG	NEG	NEG	NEG	NEG	NEG	NEG
275737	CC0133	CC133-MSSA	NEG	NEG	NEG	NEG	NEG	NEG	NEG	NEG	NEG	NEG	NEG	NEG	NEG	NEG	NEG	NEG	NEG	NEG	NEG	NEG	NEG	NEG	NEG	NEG	NEG
275738	CC0133	CC133-MSSA	NEG	NEG	NEG	NEG	NEG	NEG	NEG	NEG	NEG	NEG	NEG	NEG	NEG	NEG	NEG	NEG	NEG	NEG	NEG	NEG	NEG	NEG	NEG	NEG	NEG
275739	CC0133	CC133-MSSA	NEG	NEG	NEG	NEG	NEG	NEG	NEG	NEG	NEG	NEG	NEG	NEG	NEG	NEG	NEG	NEG	NEG	NEG	NEG	NEG	NEG	NEG	NEG	NEG	NEG
112755	CC0133	CC133-MSSA	NEG	NEG	NEG	NEG	NEG	NEG	NEG	NEG	NEG	NEG	NEG	NEG	NEG	NEG	NEG	NEG	NEG	NEG	NEG	NEG	NEG	NEG	NEG	NEG	NEG
225778	CC0398	CC398-MRSA-V/VT	POS	POS	POS	NEG	POS	NEG	NEG	NEG	NEG	NEG	NEG	NEG	NEG	NEG	NEG	NEG	NEG	NEG	NEG	NEG	NEG	NEG	NEG	POS	POS
123159	ST0425	ST425-MSSA	NEG	NEG	NEG	NEG	NEG	NEG	NEG	NEG	NEG	NEG	NEG	NEG	NEG	NEG	NEG	NEG	NEG	NEG	NEG	NEG	NEG	NEG	NEG	NEG	NEG
86991	ST0425	ST425-MSSA	NEG	NEG	NEG	NEG	NEG	NEG	NEG	NEG	NEG	NEG	NEG	NEG	NEG	NEG	NEG	NEG	NEG	NEG	NEG	NEG	NEG	NEG	NEG	NEG	NEG
112756	ST0425	ST425-MSSA	NEG	NEG	NEG	NEG	NEG	NEG	NEG	NEG	NEG	NEG	NEG	NEG	NEG	NEG	NEG	NEG	NEG	NEG	NEG	NEG	NEG	NEG	NEG	NEG	NEG
195207	ST0425	ST425-MSSA	NEG	NEG	NEG	NEG	NEG	NEG	NEG	NEG	NEG	NEG	NEG	NEG	NEG	NEG	NEG	NEG	NEG	NEG	NEG	NEG	NEG	NEG	NEG	NEG	NEG
223609	ST0425	ST425-MSSA	NEG	NEG	NEG	NEG	NEG	NEG	NEG	NEG	NEG	NEG	NEG	NEG	NEG	NEG	NEG	NEG	NEG	NEG	NEG	NEG	NEG	NEG	NEG	NEG	NEG
275740	ST0425	ST425-MSSA	NEG	NEG	NEG	NEG	NEG	NEG	NEG	NEG	NEG	NEG	NEG	NEG	NEG	NEG	NEG	NEG	NEG	NEG	NEG	NEG	NEG	NEG	NEG	NEG	NEG
174889	ST0425	ST425-MSSA	NEG	NEG	NEG	NEG	NEG	NEG	NEG	NEG	NEG	NEG	NEG	NEG	NEG	NEG	NEG	NEG	NEG	NEG	NEG	NEG	NEG	NEG	NEG	NEG	NEG
203230	ST0425	ST425-MSSA	NEG	NEG	NEG	NEG	NEG	NEG	NEG	NEG	NEG	NEG	NEG	NEG	NEG	NEG	NEG	NEG	NEG	NEG	NEG	NEG	NEG	NEG	NEG	NEG	NEG
203242	ST0425	ST425-MSSA	NEG	NEG	NEG	NEG	NEG	NEG	NEG	NEG	NEG	NEG	NEG	NEG	NEG	NEG	NEG	NEG	NEG	NEG	NEG	NEG	NEG	NEG	NEG	NEG	NEG
203243	ST0425	ST425-MSSA	NEG	NEG	NEG	NEG	NEG	NEG	NEG	NEG	NEG	NEG	NEG	NEG	NEG	NEG	NEG	NEG	NEG	NEG	NEG	NEG	NEG	NEG	NEG	NEG	NEG
207951	ST0425	ST425-MSSA	NEG	NEG	NEG	NEG	NEG	NEG	NEG	NEG	NEG	NEG	NEG	NEG	NEG	NEG	NEG	NEG	NEG	NEG	NEG	NEG	NEG	NEG	NEG	NEG	NEG
207981	ST0425	ST425-MSSA	POS	POS	AMB	NEG	NEG	NEG	NEG	NEG	NEG	NEG	NEG	NEG	NEG	NEG	NEG	NEG	NEG	NEG	NEG	NEG	NEG	NEG	NEG	NEG	NEG
195212	ST0425	ST425-MSSA	NEG	NEG	NEG	NEG	NEG	NEG	NEG	NEG	NEG	NEG	NEG	NEG	NEG	NEG	NEG	NEG	NEG	NEG	NEG	NEG	NEG	NEG	NEG	NEG	NEG
195206	ST0425	ST425-MSSA	NEG	NEG	NEG	NEG	NEG	NEG	NEG	NEG	NEG	NEG	NEG	NEG	NEG	NEG	NEG	NEG	NEG	NEG	NEG	NEG	NEG	NEG	NEG	NEG	NEG
195202	ST0425	ST425-MSSA	NEG	NEG	NEG	NEG	NEG	NEG	NEG	NEG	NEG	NEG	NEG	NEG	NEG	NEG	NEG	NEG	NEG	NEG	NEG	NEG	NEG	NEG	NEG	NEG	NEG
216042	ST0425	ST425-MSSA	NEG	NEG	NEG	NEG	NEG	NEG	NEG	NEG	NEG	NEG	NEG	NEG	NEG	NEG	NEG	NEG	NEG	NEG	NEG	NEG	NEG	NEG	NEG	NEG	NEG
223608	ST0425	ST425-MSSA	NEG	NEG	NEG	NEG	NEG	NEG	NEG	NEG	NEG	NEG	NEG	NEG	NEG	NEG	NEG	NEG	NEG	NEG	NEG	NEG	NEG	NEG	NEG	NEG	NEG
223612	ST0425	ST425-MSSA	NEG	NEG	NEG	NEG	NEG	NEG	NEG	NEG	NEG	NEG	NEG	NEG	NEG	NEG	NEG	NEG	NEG	NEG	NEG	NEG	NEG	NEG	NEG	NEG	NEG
225733	ST0425	ST425-MSSA	NEG	NEG	NEG	NEG	NEG	NEG	NEG	NEG	NEG	NEG	NEG	NEG	NEG	NEG	NEG	NEG	NEG	NEG	NEG	NEG	NEG	NEG	NEG	NEG	NEG
230321	ST0425	ST425-MSSA	NEG	NEG	NEG	NEG	NEG	NEG	NEG	NEG	NEG	NEG	NEG	NEG	NEG	NEG	NEG	NEG	NEG	NEG	NEG	NEG	NEG	NEG	NEG	NEG	NEG
232368	ST0425	ST425-MSSA	NEG	NEG	NEG	NEG	NEG	NEG	NEG	NEG	NEG	NEG	NEG	NEG	NEG	NEG	NEG	NEG	NEG	NEG	NEG	NEG	NEG	NEG	NEG	NEG	NEG
204215	ST0425	ST425-MSSA	NEG	NEG	NEG	NEG	NEG	NEG	NEG	NEG	NEG	NEG	NEG	NEG	NEG	NEG	NEG	NEG	NEG	NEG	NEG	NEG	NEG	NEG	NEG	NEG	NEG
204218	ST0425	ST425-MSSA	NEG	NEG	NEG	NEG	NEG	NEG	NEG	NEG	NEG	NEG	NEG	NEG	NEG	NEG	NEG	NEG	NEG	NEG	NEG	NEG	NEG	NEG	NEG	NEG	NEG
204222	ST0425	ST425-MSSA	NEG	NEG	NEG	NEG	NEG	NEG	NEG	NEG	NEG	NEG	NEG	NEG	NEG	NEG	NEG	NEG	NEG	NEG	NEG	NEG	NEG	NEG	NEG	NEG	NEG
204226	ST0425	ST425-MSSA	NEG	NEG	NEG	NEG	NEG	NEG	NEG	NEG	NEG	NEG	NEG	NEG	NEG	NEG	NEG	NEG	NEG	NEG	NEG	NEG	NEG	NEG	NEG	NEG	NEG
204906	ST0425	ST425-MSSA	NEG	NEG	NEG	NEG	NEG	NEG	NEG	NEG	NEG	NEG	NEG	NEG	NEG	NEG	NEG	NEG	NEG	NEG	NEG	NEG	NEG	NEG	NEG	NEG	NEG
204908	ST0425	ST425-MSSA	NEG	NEG	NEG	NEG	NEG	NEG	NEG	NEG	NEG	NEG	NEG	NEG	NEG	NEG	NEG	NEG	NEG	NEG	NEG	NEG	NEG	NEG	NEG	NEG	NEG
206170	ST0425	ST425-MSSA	NEG	NEG	NEG	NEG	NEG	NEG	NEG	NEG	NEG	NEG	NEG	NEG	NEG	NEG	NEG	NEG	NEG	NEG	NEG	NEG	NEG	NEG	NEG	NEG	NEG
207765	ST0425	ST425-MSSA	NEG	NEG	NEG	NEG	NEG	NEG	NEG	NEG	NEG	NEG	NEG	NEG	NEG	NEG	NEG	NEG	NEG	NEG	NEG	NEG	NEG	NEG	NEG	NEG	NEG
240048	CC599	CC599-MRSA-XI	NEG	NEG	NEG	NEG	NEG	NEG	NEG	NEG	NEG	NEG	NEG	NEG	NEG	NEG	NEG	NEG	NEG	NEG	NEG	NEG	NEG	NEG	NEG	NEG	NEG
225730	CC0692	CC692-MSSA	NEG	NEG	NEG	NEG	NEG	NEG	NEG	NEG	NEG	NEG	NEG	NEG	NEG	NEG	NEG	NEG	NEG	NEG	NEG	NEG	NEG	NEG	NEG	NEG	NEG
112746	CC0692	CC692-MSSA	NEG	NEG	NEG	NEG	NEG	NEG	NEG	NEG	NEG	NEG	NEG	NEG	NEG	NEG	NEG	NEG	NEG	NEG	NEG	NEG	NEG	NEG	NEG	NEG	NEG
112754	CC0692	CC692-MSSA	NEG	NEG	NEG	NEG	NEG	NEG	NEG	NEG	NEG	NEG	NEG	NEG	NEG	NEG	NEG	NEG	NEG	NEG	NEG	NEG	NEG	NEG	NEG	NEG	NEG
112758	CC0692	CC692-MSSA	NEG	NEG	NEG	NEG	NEG	NEG	NEG	NEG	NEG	NEG	NEG	NEG	NEG	NEG	NEG	NEG	NEG	NEG	NEG	NEG	NEG	NEG	NEG	NEG	NEG
112759	CC0692	CC692-MSSA	NEG	NEG	NEG	NEG	NEG	NEG	NEG	NEG	NEG	NEG	NEG	NEG	NEG	NEG	NEG	NEG	NEG	NEG	NEG	NEG	NEG	NEG	NEG	NEG	NEG
124745	CC0692	CC692-MSSA	NEG	NEG	NEG	NEG	NEG	NEG	NEG	NEG	NEG	NEG	NEG	NEG	NEG	NEG	NEG	NEG	NEG	NEG	NEG	NEG	NEG	NEG	NEG	NEG	NEG
170952	CC0692	CC692-MSSA	NEG	NEG	NEG	NEG	NEG	NEG	NEG	NEG	NEG	NEG	NEG	NEG	NEG	NEG	NEG	NEG	NEG	NEG	NEG	NEG	NEG	NEG	NEG	NEG	NEG
170953	CC0692	CC692-MSSA	NEG	NEG	NEG	NEG	NEG	NEG	NEG	NEG	NEG	NEG	NEG	NEG	NEG	NEG	NEG	NEG	NEG	NEG	NEG	NEG	NEG	NEG	NEG	NEG	NEG
170970	CC0692	CC692-MSSA	NEG	NEG	NEG	NEG	NEG	NEG	NEG	NEG	NEG	NEG	NEG	NEG	NEG	NEG	NEG	NEG	NEG	NEG	NEG	NEG	NEG	NEG	NEG	NEG	NEG
170950	CC0707	CC707-MSSA	NEG	NEG	NEG	NEG	NEG	NEG	NEG	NEG	NEG	NEG	NEG	NEG	NEG	NEG	NEG	NEG	NEG	NEG	NEG	NEG	NEG	NEG	NEG	NEG	NEG
124948	ST0890	ST890-MSSA	POS	POS	POS	NEG	NEG	NEG	NEG	NEG	NEG	NEG	NEG	NEG	NEG	NEG	NEG	NEG	NEG	NEG	NEG	NEG	NEG	NEG	NEG	NEG	NEG
220618	CC1956	CC1956-MSSA	NEG	NEG	NEG	NEG	NEG	NEG	NEG	NEG	NEG	NEG	NEG	NEG	NEG	NEG	NEG	NEG	NEG	NEG	NEG	NEG	NEG	NEG	NEG	NEG	NEG
112742	ST2425	ST2425-MSSA	NEG	NEG	NEG	NEG	NEG	NEG	NEG	NEG	NEG	NEG	NEG	NEG	NEG	NEG	NEG	NEG	NEG	NEG	NEG	NEG	NEG	NEG	NEG	NEG	NEG
170957	ST2425	ST2425-MSSA	NEG	NEG	NEG	NEG	NEG	NEG	NEG	NEG	NEG	NEG	NEG	NEG	NEG	NEG	NEG	NEG	NEG	NEG	NEG	NEG	NEG	NEG	NEG	NEG	NEG
170947	ST2691	ST2691-MSSA	NEG	NEG	NEG	NEG	NEG	NEG	NEG	NEG	NEG	NEG	NEG	NEG	NEG	NEG	NEG	NEG	NEG	NEG	NEG	NEG	NEG	NEG	NEG	NEG	NEG
170961	ST2691	ST2691-MSSA	NEG	NEG	NEG	NEG	NEG	NEG	NEG	NEG	NEG	NEG	NEG	NEG	NEG	NEG	NEG	NEG	NEG	NEG	NEG	NEG	NEG	NEG	NEG	NEG	NEG
112753	CC2767	ST2279-MSSA	NEG	NEG	NEG	NEG	NEG	NEG	NEG	NEG	NEG	NEG	NEG	NEG	NEG	NEG	NEG	NEG	NEG	NEG	NEG	NEG	NEG	NEG	NEG	NEG	NEG
112761	CC2767	ST2279-MSSA	NEG	NEG	NEG	NEG	NEG	NEG	NEG	NEG	NEG	NEG	NEG	NEG	NEG	NEG	NEG	NEG	NEG	NEG	NEG	NEG	NEG	NEG	NEG	NEG	NEG
228651	ST2963	ST2963-MSSA	NEG	NEG	NEG	NEG	NEG	NEG	NEG	NEG	NEG	NEG	NEG	NEG	NEG	NEG	NEG	NEG	NEG	NEG	NEG	NEG	NEG	NEG	NEG	NEG	NEG
203244	ST3237	ST3237-MSSA	NEG	NEG	NEG	NEG	NEG	NEG	NEG	NEG	NEG	NEG	NEG	NEG	NEG	NEG	NEG	NEG	NEG	NEG	NEG	NEG	NEG	NEG	NEG	NEG	NEG

ISOLATE ID	CLONAL COMPLEX (by array)	STRAIN AFFILIATION (by array)	RESISTANCE : MISCELLANEOUS GENES							RESISTANCE : EFFLUX SYSTEMS			RESISTANCE : GLYCOPEPTIDES			VIRULENCE : TOX.SCHOCK.TOXIN			VIRULENCE : ENTEROTOXINS										
			chloramphenicol acetyltransferase					23S rRNA methyltransferase	chloramphenicol/florfenicol exporter	putative metallothiol transferase	quaternary ammonium compound resistance protein A	quaternary ammonium compound resistance protein C	vancomycin resistance gene	vancomycin resistance gene from enterococci and Clostridium	teicoplanin resistance gene from enterococci	toxic shock syndrome toxin 1			enterotoxin A	enterotoxin A, allele from strain 320E	enterotoxin A, allele from strain N315 = entero-toxin P	enterotoxin B	enterotoxin C	enterotoxin D	enterotoxin E	enterotoxin H	enterotoxin J	enterotoxin K	enterotoxin L
			cat	cat (pC221)	cat (pc223)	cat (pMC524)	cat (pSBK203R)	cfr	fexA	fosB	qacA	qacC	vanA	vanB	vanZ	tst1 (consensus)	tst1 ("human" allele)	tst1 ("bovine" allele)	entA	entA (320E)	entA (N315) / entP	entB	entC	entD	entE	entH	entJ	entK	entL
203228	CC0001	CC1-MSSA	NEG	NEG	NEG	NEG	NEG	NEG	NEG	AMB	NEG	NEG	NEG	NEG	NEG	NEG	NEG	NEG	NEG	NEG	NEG	NEG	NEG	NEG	NEG	POS	NEG	NEG	NEG
203229	CC0001	CC1-MSSA	NEG	NEG	NEG	NEG	NEG	NEG	NEG	AMB	NEG	NEG	NEG	NEG	NEG	NEG	NEG	NEG	NEG	NEG	NEG	AMB	NEG	AMB	NEG	POS	NEG	NEG	NEG
203233	CC0001	CC1-MSSA	NEG	NEG	NEG	NEG	NEG	NEG	NEG	NEG	NEG	NEG	NEG	NEG	NEG	NEG	NEG	NEG	NEG	NEG	NEG	NEG	NEG	NEG	NEG	POS	NEG	NEG	NEG
220334	CC0001	CC1-MSSA	NEG	NEG	NEG	NEG	NEG	NEG	NEG	NEG	NEG	NEG	NEG	NEG	NEG	NEG	NEG	NEG	NEG	NEG	NEG	NEG	NEG	NEG	NEG	POS	NEG	NEG	NEG
223621	CC0001	CC1-MSSA	NEG	NEG	NEG	NEG	NEG	NEG	NEG	NEG	NEG	NEG	NEG	NEG	NEG	NEG	NEG	NEG	NEG	NEG	NEG	NEG	NEG	NEG	NEG	POS	NEG	NEG	NEG
289123	CC0001	CC1-MRSA-IV, WA MRSA-1/57	NEG	NEG	NEG	NEG	NEG	NEG	NEG	NEG	NEG	NEG	NEG	NEG	NEG	NEG	NEG	NEG	NEG	NEG	NEG	NEG	NEG	NEG	NEG	POS	NEG	NEG	NEG
289124	CC0001	CC1-MRSA-IV, WA MRSA-1/57	NEG	NEG	NEG	NEG	NEG	NEG	NEG	NEG	NEG	NEG	NEG	NEG	NEG	NEG	NEG	NEG	NEG	NEG	NEG	NEG	NEG	NEG	NEG	POS	NEG	NEG	NEG
275170	CC0005	CC5-MSSA	NEG	NEG	NEG	NEG	NEG	NEG	NEG	POS	NEG	NEG	NEG	NEG	NEG	NEG	NEG	NEG	POS	NEG	NEG	NEG	NEG	NEG	NEG	NEG	NEG	NEG	NEG
275171	CC0005	CC5-MSSA	NEG	NEG	NEG	NEG	NEG	NEG	NEG	POS	NEG	NEG	NEG	NEG	NEG	NEG	NEG	NEG	POS	NEG	NEG	NEG	NEG	NEG	NEG	NEG	NEG	NEG	NEG
112747	CC0005	CC5-MSSA	NEG	NEG	NEG	NEG	NEG	NEG	NEG	POS	NEG	NEG	NEG	NEG	NEG	NEG	NEG	NEG	NEG	NEG	NEG	NEG	NEG	NEG	NEG	NEG	NEG	NEG	NEG
112752	CC0006	CC6-MSSA	NEG	NEG	NEG	NEG	NEG	NEG	NEG	POS	NEG	NEG	NEG	NEG	NEG	NEG	NEG	NEG	POS	NEG	NEG	NEG	NEG	NEG	NEG	NEG	NEG	NEG	NEG
275178	CC0007	CC7-MSSA	NEG	NEG	NEG	NEG	NEG	NEG	NEG	NEG	NEG	NEG	NEG	NEG	NEG	NEG	NEG	NEG	NEG	NEG	POS	NEG	NEG	NEG	NEG	NEG	NEG	NEG	NEG
275166	CC0008	CC8-MSSA	NEG	NEG	NEG	NEG	NEG	NEG	NEG	POS	NEG	NEG	NEG	NEG	NEG	NEG	NEG	NEG	POS	NEG	NEG	NEG	NEG	POS	NEG	NEG	POS	NEG	NEG
275176	CC0008	CC8-MSSA	NEG	NEG	NEG	NEG	NEG	NEG	NEG	POS	NEG	NEG	NEG	NEG	NEG	NEG	NEG	NEG	POS	NEG	NEG	NEG	NEG	POS	NEG	NEG	POS	NEG	NEG
275177	CC0008	CC8-MSSA	NEG	NEG	NEG	NEG	NEG	NEG	NEG	POS	NEG	NEG	NEG	NEG	NEG	NEG	NEG	NEG	NEG	NEG	NEG	NEG	NEG	NEG	NEG	NEG	NEG	NEG	NEG
275167	CC0009	CC9-MSSA	NEG	NEG	NEG	NEG	NEG	NEG	NEG	POS	NEG	NEG	NEG	NEG	NEG	NEG	NEG	NEG	NEG	NEG	NEG	NEG	NEG	NEG	NEG	NEG	NEG	NEG	NEG
170968	CC0012	CC12-MSSA	NEG	NEG	NEG	NEG	NEG	NEG	NEG	POS	NEG	NEG	NEG	NEG	NEG	NEG	NEG	NEG	NEG	NEG	NEG	NEG	NEG	NEG	NEG	NEG	NEG	NEG	NEG
275175	CC0015	CC15-MSSA	NEG	NEG	NEG	NEG	NEG	NEG	NEG	POS	NEG	NEG	NEG	NEG	NEG	NEG	NEG	NEG	NEG	NEG	NEG	NEG	NEG	NEG	NEG	NEG	NEG	NEG	NEG
170949	CC0015	CC15-MSSA	NEG	NEG	NEG	NEG	NEG	NEG	NEG	POS	NEG	NEG	NEG	NEG	NEG	NEG	NEG	NEG	NEG	NEG	NEG	NEG	NEG	NEG	NEG	NEG	NEG	NEG	NEG
228658	CC0022	CC22-MSSA	NEG	NEG	NEG	NEG	NEG	NEG	NEG	NEG	NEG	NEG	NEG	NEG	NEG	NEG	NEG	NEG	NEG	NEG	NEG	NEG	NEG	NEG	NEG	NEG	NEG	NEG	NEG
289125	CC0022	CC22-MRSA-IV (PVL+)	NEG	NEG	NEG	NEG	NEG	NEG	NEG	NEG	NEG	NEG	NEG	NEG	NEG	NEG	NEG	NEG	NEG	NEG	NEG	NEG	NEG	NEG	NEG	NEG	NEG	NEG	NEG
289126	CC0022	CC22-MRSA-IV (PVL+)	NEG	NEG	NEG	NEG	NEG	NEG	NEG	NEG	NEG	NEG	NEG	NEG	NEG	NEG	NEG	NEG	NEG	NEG	NEG	NEG	NEG	NEG	NEG	NEG	NEG	NEG	NEG
289127	CC0022	CC22-MRSA-IV (PVL+)	NEG	NEG	NEG	NEG	NEG	NEG	NEG	NEG	NEG	NEG	NEG	NEG	NEG	NEG	NEG	NEG	NEG	NEG	NEG	NEG	NEG	NEG	NEG	NEG	NEG	NEG	NEG
131939	CC0025	CC25-MSSA	NEG	NEG	NEG	NEG	NEG	NEG	NEG	POS	NEG	NEG	NEG	NEG	NEG	NEG	NEG	NEG	NEG	NEG	NEG	POS	NEG	NEG	NEG	NEG	NEG	NEG	NEG
275165	CC0030	CC30-MSSA (lukF-P83/lukM+)	NEG	NEG	NEG	NEG	NEG	POS	POS	POS	NEG	NEG	NEG	NEG	NEG	NEG	NEG	NEG	NEG	NEG	NEG	NEG	NEG	NEG	NEG	NEG	NEG	NEG	NEG
275741	CC0030	CC30-MSSA	NEG	NEG	NEG	NEG	NEG	NEG	NEG	POS	NEG	NEG	NEG	NEG	NEG	NEG	NEG	NEG	NEG	NEG	NEG	NEG	NEG	NEG	NEG	NEG	NEG	NEG	NEG
124492	CC0049	CC49-MSSA (lukF-P83/lukM+)	NEG	NEG	NEG	NEG	NEG	NEG	NEG	NEG	NEG	NEG	NEG	NEG	NEG	NEG	NEG	NEG	NEG	NEG	NEG	NEG	NEG	NEG	NEG	NEG	NEG	NEG	NEG
220616	CC0049	CC49-MSSA (lukF-P83/lukM+)	NEG	NEG	NEG	NEG	NEG	NEG	NEG	NEG	NEG	NEG	NEG	NEG	NEG	NEG	NEG	NEG	NEG	NEG	NEG	NEG	NEG	NEG	NEG	NEG	NEG	NEG	NEG
228649	CC0049	CC49-MSSA (lukF-P83/lukM+)	NEG	NEG	NEG	NEG	NEG	NEG	NEG	NEG	NEG	NEG	NEG	NEG	NEG	NEG	NEG	NEG	NEG	NEG	NEG	NEG	NEG	NEG	NEG	NEG	NEG	NEG	NEG
190994	CC0059	CC59-MSSA	NEG	NEG	NEG	NEG	NEG	NEG	NEG	NEG	NEG	NEG	NEG	NEG	NEG	NEG	NEG	NEG	NEG	NEG	NEG	NEG	NEG	NEG	NEG	NEG	NEG	NEG	NEG
275172	CC0088	CC88-MSSA	POS	POS	NEG	NEG	NEG	NEG	NEG	NEG	NEG	NEG	NEG	NEG	NEG	NEG	NEG	NEG	NEG	NEG	NEG	NEG	NEG	NEG	NEG	NEG	NEG	NEG	NEG
275173	CC0088	CC88-MSSA	POS	POS	NEG	NEG	NEG	NEG	NEG	NEG	NEG	NEG	NEG	NEG	NEG	NEG	NEG	NEG	NEG	NEG	NEG	NEG	NEG	NEG	NEG	NEG	NEG	NEG	NEG
275174	CC0088	CC88-MSSA	POS	POS	NEG	NEG	NEG	NEG	NEG	NEG	NEG	NEG	NEG	NEG	NEG	NEG	NEG	NEG	NEG	NEG	NEG	NEG	NEG	NEG	NEG	NEG	NEG	NEG	NEG
170971	CC0097	CC97-MSSA	NEG	NEG	NEG	NEG	NEG	NEG	NEG	NEG	NEG	NEG	NEG	NEG	NEG	NEG	NEG	NEG	NEG	NEG	NEG	NEG	NEG	NEG	NEG	NEG	NEG	NEG	NEG
112745	CC0097	CC97-MSSA	NEG	NEG	NEG	NEG	NEG	NEG	NEG	NEG	NEG	NEG	NEG	NEG	NEG	NEG	NEG	NEG	NEG	NEG	NEG	NEG	NEG	NEG	NEG	NEG	NEG	NEG	NEG
112748	CC0097	CC97-MSSA	NEG	NEG	NEG	NEG	NEG	NEG	NEG	NEG	NEG	NEG	NEG	NEG	NEG	NEG	NEG	NEG	NEG	NEG	NEG	NEG	NEG	NEG	NEG	NEG	NEG	NEG	NEG
112750	CC0097	CC97-MSSA	NEG	NEG	NEG	NEG	NEG	NEG	NEG	NEG	NEG	NEG	NEG	NEG	NEG	NEG	NEG	NEG	NEG	NEG	NEG	NEG	NEG	NEG	NEG	NEG	NEG	NEG	NEG
112762	CC0097	CC97-MSSA	NEG	NEG	NEG	NEG	NEG	NEG	NEG	NEG	NEG	NEG	NEG	NEG	NEG	NEG	NEG	NEG	NEG	NEG	NEG	NEG	NEG	NEG	NEG	NEG	NEG	NEG	NEG
112763	CC0097	CC97-MSSA	NEG	NEG	NEG	NEG	NEG	NEG	NEG	NEG	NEG	NEG	NEG	NEG	NEG	NEG	NEG	NEG	NEG	NEG	NEG	NEG	NEG	NEG	NEG	NEG	NEG	NEG	NEG
112764	CC0097	CC97-MSSA	NEG	NEG	NEG	NEG	NEG	NEG	NEG	NEG	NEG	NEG	NEG	NEG	NEG	NEG	NEG	NEG	NEG	NEG	NEG	NEG	NEG	NEG	NEG	NEG	NEG	NEG	NEG
170945	CC0097	CC97-MSSA	NEG	NEG	NEG	NEG	NEG	NEG	NEG	NEG	NEG	NEG	NEG	NEG	NEG	NEG	NEG	NEG	NEG	NEG	NEG	NEG	NEG	NEG	NEG	NEG	NEG	NEG	NEG
170954	CC0097	CC97-MSSA	NEG	NEG	NEG	NEG	NEG	NEG	NEG	NEG	NEG	NEG	NEG	NEG	NEG	NEG	NEG	NEG	NEG	NEG	NEG	NEG	NEG	NEG	NEG	NEG	NEG	NEG	NEG
170958	CC0097	CC97-MSSA	NEG	NEG	NEG	NEG	NEG	NEG	NEG	NEG	NEG	NEG	NEG	NEG	NEG	NEG	NEG	NEG	NEG	NEG	NEG	NEG	NEG	NEG	NEG	NEG	NEG	NEG	NEG
170963	CC0097	CC97-MSSA	NEG	NEG	NEG	NEG	NEG	NEG	NEG	NEG	NEG	NEG	NEG	NEG	NEG	NEG	NEG	NEG	NEG	NEG	NEG	NEG	NEG	NEG	NEG	NEG	NEG	NEG	NEG
170964	CC0097	CC97-MSSA	NEG	NEG	NEG	NEG	NEG	NEG	NEG	NEG	NEG	NEG	NEG	NEG	NEG	NEG	NEG	NEG	NEG	NEG	NEG	NEG	NEG	NEG	NEG	NEG	NEG	NEG	NEG
170965	CC0097	CC97-MSSA	NEG	NEG	NEG	NEG	NEG	NEG	NEG	NEG	NEG	NEG	NEG	NEG	NEG	NEG	NEG	NEG	NEG	NEG	NEG	NEG	NEG	NEG	NEG	NEG	NEG	NEG	NEG
170966	CC0097	CC97-MSSA	NEG	NEG	NEG	NEG	NEG	NEG	NEG	NEG	NEG	NEG	NEG	NEG	NEG	NEG	NEG	NEG	NEG	NEG	NEG	NEG	NEG	NEG	NEG	NEG	NEG	NEG	NEG
170974	CC0097	CC97-MSSA	NEG	NEG	NEG	NEG	NEG	NEG	NEG	NEG	NEG	NEG	NEG	NEG	NEG	NEG	NEG	NEG	NEG	NEG	NEG	NEG	NEG	NEG	NEG	NEG	NEG	NEG	NEG
170976	CC0097	CC97-MSSA	NEG	NEG	NEG	NEG	NEG	NEG	NEG	NEG	NEG	NEG	NEG	NEG	NEG	NEG	NEG	NEG	NEG	NEG	NEG	NEG	NEG	NEG	NEG	NEG	NEG	NEG	NEG
112744	CC0097	CC97-MSSA	NEG	NEG	NEG	NEG	NEG	NEG	NEG	NEG	NEG	NEG	NEG	NEG	NEG	NEG	NEG	NEG	NEG	NEG	NEG	NEG	NEG	NEG	NEG	NEG	NEG	NEG	NEG
112749	CC0097	CC97-MSSA	NEG	NEG	NEG	NEG	NEG	NEG	NEG	NEG	NEG	NEG	NEG	NEG	NEG	NEG	NEG	NEG	NEG	NEG	NEG	NEG	NEG	NEG	NEG	NEG	NEG	NEG	NEG
112757	CC0097	CC97-MSSA	NEG	NEG	NEG	NEG	NEG	NEG	NEG	NEG	NEG	NEG	NEG	NEG	NEG	NEG	NEG	NEG	NEG	NEG	NEG	NEG	NEG	NEG	NEG	NEG	NEG	NEG	NEG
170973	CC0097	CC97-MSSA	NEG	NEG	NEG	NEG	NEG	NEG	NEG	NEG	NEG	NEG	NEG	NEG	NEG	NEG	NEG	NEG	NEG	NEG	NEG	NEG	NEG	NEG	NEG	NEG	NEG	NEG	NEG
207756	CC0097	CC97-MSSA	NEG	NEG	NEG	NEG	NEG	NEG	NEG	NEG	NEG	NEG	NEG	NEG	NEG	NEG	NEG	NEG	NEG	NEG	NEG	NEG	NEG	NEG	NEG	NEG	NEG	NEG	NEG
206171	CC0097	CC97-MSSA	NEG	NEG	NEG	NEG	NEG	NEG	NEG	NEG	NEG	NEG	NEG	NEG	NEG	NEG	NEG	NEG	NEG	NEG	NEG	NEG	NEG	NEG	NEG	NEG	NEG	NEG	NEG
275169	CC0097	CC97-MSSA-[ccrAA]	NEG	NEG	NEG	NEG	NEG	NEG	NEG	NEG	NEG	NEG	NEG	NEG	NEG	NEG	NEG	NEG	NEG	NEG	NEG	NEG	NEG	NEG	NEG	NEG	NEG	NEG	NEG
275168	CC0097	CC97-MSSA-[ccrAA]	NEG	NEG	NEG	NEG	NEG	NEG	NEG	NEG	NEG	NEG	NEG	NEG	NEG	NEG	NEG	NEG	NEG	NEG	NEG	NEG	NEG	NEG	NEG	NEG	NEG	NEG	NEG
118320	CC0130	CC130-MSSA (lukF-P83/M+)	NEG	NEG	NEG	NEG	NEG	NEG	NEG	NEG	NEG	NEG	NEG	NEG	NEG	NEG	NEG	NEG	NEG	NEG	NEG	NEG	NEG	NEG	NEG	NEG	NEG	NEG	NEG
203236	CC0130	CC130-MRSA-XI	NEG	NEG	NEG	NEG	NEG	NEG	NEG	NEG	NEG	NEG	NEG	NEG	NEG	NEG	NEG	NEG	NEG	NEG	NEG	NEG	NEG	NEG	NEG	NEG	NEG	NEG	NEG
204204	CC0130	CC130-MRSA-XI	NEG	NEG	NEG	NEG	NEG	NEG	NEG	NEG	NEG	NEG	NEG	NEG	NEG	NEG	NEG	NEG	NEG	NEG	NEG	NEG	NEG	NEG	NEG	NEG	NEG	NEG	NEG
289122	CC0130	CC130-MRSA-XI	NEG	NEG	NEG	NEG	NEG	NEG	NEG	NEG	NEG	NEG	NEG	NEG	NEG	NEG	NEG	NEG	NEG	NEG	NEG	NEG	NEG	NEG	NEG	NEG	NEG	NEG	NEG
225779	CC0130	CC130-MRSA-XI	NEG	NEG	NEG	NEG	NEG	NEG	NEG	NEG	NEG	NEG	NEG	NEG	NEG	NEG	NEG	NEG	NEG	NEG	NEG	NEG	NEG	NEG	NEG	NEG	NEG	NEG	NEG
225780	CC0130	CC130-MRSA-XI	NEG	NEG	NEG	NEG	NEG	NEG	NEG	NEG	NEG	NEG	NEG	NEG	NEG	NEG	NEG	NEG	NEG	NEG	NEG	NEG	NEG	NEG	NEG	NEG	NEG	NEG	NEG
170959	CC0130	CC130-MRSA-XI	NEG	NEG	NEG	NEG	NEG	NEG	NEG	NEG	NEG	NEG	NEG	NEG	NEG	NEG	NEG	NEG	NEG	NEG	NEG	NEG	NEG	NEG	NEG	NEG	NEG	NEG	NEG
179029	CC0130	CC130-MRSA-XI	NEG	NEG	NEG	NEG	NEG	NEG	NEG	NEG	NEG	NEG	NEG	NEG	NEG	NEG	NEG	NEG	NEG	NEG	NEG	NEG	NEG	NEG	NEG	NEG	NEG	NEG	NEG
116254	CC0133	CC133-MSSA	NEG	NEG	NEG	NEG	NEG	NEG	NEG	POS	NEG	NEG	NEG	NEG	NEG	NEG	NEG	NEG	NEG	NEG	NEG	NEG	NEG	NEG	NEG	NEG	NEG	NEG	NEG
207761	CC0133	CC133-MSSA	NEG	NEG	NEG	NEG	NEG	NEG	NEG	POS	NEG	NEG	NEG	NEG	NEG	NEG	NEG	NEG	NEG	NEG	NEG	NEG	NEG	NEG	NEG	NEG	NEG	NEG	NEG
213103	CC0133	CC133-MSSA	NEG	NEG	NEG	NEG	NEG	NEG	NEG	POS	NEG	NEG	NEG	NEG	NEG	NEG	NEG	NEG	NEG	NEG	NEG	NEG	NEG	NEG	NEG	NEG	NEG	NEG	NEG
275736	CC0133	CC133-MSSA	NEG	NEG	NEG	NEG	NEG	NEG	NEG	POS	NEG	NEG	NEG	NEG	NEG	NEG	NEG	NEG	NEG	NEG	NEG	NEG	NEG	NEG	NEG	NEG	NEG	NEG	NEG
275737	CC0133	CC133-MSSA	NEG	NEG	NEG	NEG	NEG	NEG	NEG	POS	NEG	NEG	NEG	NEG	NEG	NEG	NEG	NEG	NEG	NEG	NEG	NEG	NEG	NEG	NEG	NEG	NEG	NEG	NEG
275738	CC0133	CC133-MSSA	NEG	NEG	NEG	NEG	NEG	NEG	NEG	POS	NEG	NEG	NEG	NEG	NEG	NEG	NEG	NEG	NEG	NEG	NEG	NEG	NEG	NEG	NEG	NEG	NEG	NEG	NEG
275739	CC0133	CC133-MSSA	NEG	NEG	NEG	NEG	NEG	NEG	NEG	POS	NEG	NEG	NEG	NEG	NEG	NEG	NEG	NEG	NEG	NEG	NEG	NEG	NEG	NEG	NEG	NEG	NEG	NEG	NEG
112755	CC0133	CC133-MSSA	NEG	NEG	NEG	NEG	NEG	NEG	NEG	POS	NEG	NEG	NEG	NEG	NEG	NEG	NEG	NEG	NEG	NEG	NEG	NEG	NEG	NEG	NEG	NEG	NEG	NEG	NEG
225778	CC0398	CC398-MRSA-V/VT	NEG	NEG	NEG	NEG	NEG	NEG	NEG	NEG	NEG	NEG	NEG	NEG	NEG	NEG	NEG	NEG	NEG	NEG	NEG	NEG	NEG	NEG	NEG	NEG	NEG	NEG	NEG
123159	ST0425	ST425-MSSA	NEG	NEG	NEG	NEG	NEG	NEG	NEG	POS	NEG	NEG	NEG	NEG	NEG	NEG	NEG	NEG	NEG	NEG	NEG	NEG	NEG	NEG	NEG	NEG	NEG	NEG	NEG
86991	ST0425	ST425-MSSA	NEG	NEG	NEG	NEG	NEG	NEG	NEG	POS	NEG	NEG	NEG	NEG	NEG	NEG	NEG	NEG	NEG	NEG	NEG	NEG	NEG	NEG	POS	NEG	NEG	NEG	NEG
112756	ST0425	ST425-MSSA	NEG	NEG	NEG	NEG	NEG	NEG	NEG	POS	NEG	NEG	NEG	NEG	NEG	NEG	NEG	NEG	NEG	NEG	NEG	NEG	NEG	NEG	POS	NEG	NEG	NEG	NEG
195207	ST0425	ST425-MSSA	NEG	NEG	NEG	NEG	NEG	NEG	NEG	POS	NEG	NEG	NEG	NEG	NEG	NEG	NEG	NEG	NEG	NEG	NEG	NEG	NEG	NEG	NEG	NEG	NEG	NEG	NEG
223609	ST0425	ST425-MSSA	NEG	NEG	NEG	NEG	NEG	NEG	NEG	POS	NEG	NEG	NEG	NEG	NEG	NEG	NEG	NEG	NEG	NEG	NEG	NEG	NEG	AMB	NEG	NEG	NEG	NEG	NEG
275740	ST0425	ST425-MSSA	NEG	NEG	NEG	NEG	NEG	NEG	NEG	POS	NEG	NEG	NEG	NEG	NEG	NEG	NEG	NEG	NEG	NEG	NEG	NEG	NEG	NEG	NEG	NEG	NEG	NEG	NEG
174889	ST0425	ST425-MSSA	NEG	NEG	NEG	NEG	NEG	NEG	NEG	POS	NEG	NEG	NEG	NEG	NEG	NEG	NEG	NEG	NEG	NEG	NEG	NEG	NEG	NEG	NEG	NEG	NEG	NEG	NEG
203230	ST0425	ST425-MSSA	NEG	NEG	NEG	NEG	NEG	NEG	NEG	POS	NEG	NEG	NEG	NEG	NEG	NEG	NEG	NEG	NEG	NEG	NEG	AMB	NEG	NEG	NEG	NEG	NEG	NEG	NEG
203242	ST0425	ST425-MSSA	NEG	NEG	NEG	NEG	NEG	NEG	NEG	POS	NEG	NEG	NEG	NEG	NEG	NEG	NEG	NEG	NEG	NEG	NEG	NEG	NEG	AMB	NEG	NEG	NEG	NEG	NEG
203243	ST0425	ST425-MSSA	NEG	NEG	NEG	NEG	NEG	NEG	NEG	POS	NEG	NEG	NEG	NEG	NEG	NEG	NEG	NEG	NEG	NEG	NEG	NEG	NEG	AMB	NEG	NEG	NEG	NEG	NEG
207951	ST0425	ST425-MSSA	NEG	NEG	NEG	NEG	NEG	NEG	NEG	POS	NEG	NEG	NEG	NEG	NEG	NEG	NEG	NEG	NEG	NEG	NEG	NEG	NEG	NEG	NEG	NEG	NEG	NEG	NEG
207981	ST0425	ST425-MSSA	NEG	NEG	NEG	NEG	NEG	NEG	NEG	POS	NEG	NEG	NEG	NEG	NEG	NEG	NEG	NEG	NEG	NEG	NEG	NEG	NEG	NEG	POS	NEG	NEG	NEG	NEG
195212	ST0425	ST425-MSSA	NEG	NEG	NEG	NEG	NEG	NEG	NEG	POS	NEG	NEG	NEG	NEG	NEG	NEG	NEG	NEG	NEG	NEG	NEG	NEG	NEG	NEG	NEG	NEG	NEG	NEG	NEG
195206	ST0425	ST425-MSSA	NEG	NEG	NEG	NEG	NEG	NEG	NEG	POS	NEG	NEG	NEG	NEG	NEG	NEG	NEG	NEG	NEG	NEG	NEG	NEG	NEG	NEG	NEG	NEG	NEG	NEG	NEG
195202	ST0425	ST425-MSSA	NEG	NEG	NEG	NEG	NEG	NEG	NEG	POS	NEG	NEG	NEG	NEG	NEG	NEG	NEG	NEG	NEG	NEG	NEG	NEG	NEG	NEG	NEG	NEG	NEG	NEG	NEG
216042	ST0425	ST425-MSSA	NEG	NEG	NEG	NEG	NEG	NEG	NEG	POS	NEG	NEG	NEG	NEG	NEG	NEG	NEG	NEG	NEG	NEG	NEG	NEG	NEG	NEG	NEG	NEG	NEG	NEG	NEG
223608	ST0425	ST425-MSSA	NEG	NEG	NEG	NEG	NEG	NEG	NEG	POS	NEG	NEG	NEG	NEG	NEG	NEG	NEG	NEG	NEG	NEG	NEG	NEG	NEG	AMB	NEG	NEG	NEG	NEG	NEG
223612	ST0425	ST425-MSSA	NEG	NEG	NEG	NEG	NEG	NEG	NEG	POS	NEG	NEG	NEG	NEG	NEG	NEG	NEG	NEG	NEG	NEG	NEG	NEG	NEG	NEG	NEG	NEG	NEG	NEG	NEG
225733	ST0425	ST425-MSSA	NEG	NEG	NEG	NEG	NEG	NEG	NEG	POS	NEG	NEG	NEG	NEG	NEG	NEG	NEG	NEG	NEG	NEG	NEG	NEG	NEG	NEG	NEG	NEG	NEG	NEG	NEG
230321	ST0425	ST425-MSSA	NEG	NEG	NEG	NEG	NEG	NEG	NEG	POS	NEG	NEG	NEG	NEG	NEG	NEG	NEG	NEG	NEG	NEG	NEG	NEG	NEG	NEG	NEG	NEG	NEG	NEG	NEG
232368	ST0425	ST425-MSSA	NEG	NEG	NEG	NEG	NEG	NEG	NEG	POS	NEG	NEG	NEG	NEG	NEG	NEG	NEG	NEG	NEG	NEG	NEG	NEG	NEG	NEG	NEG	NEG	NEG	NEG	NEG
204215	ST0425	ST425-MSSA	NEG	NEG	NEG	NEG	NEG	NEG	NEG	POS	NEG	NEG	NEG	NEG	NEG	NEG	NEG	NEG	NEG	NEG	NEG	NEG	NEG	NEG	NEG	NEG	NEG	NEG	NEG
204218	ST0425	ST425-MSSA	NEG	NEG	NEG	NEG	NEG	NEG	NEG	POS	NEG	NEG	NEG	NEG	NEG	NEG	NEG	NEG	NEG	NEG	NEG	NEG	NEG	NEG	NEG	NEG	NEG	NEG	NEG
204222	ST0425	ST425-MSSA	NEG	NEG	NEG	NEG	NEG	NEG	NEG	POS	NEG	NEG	NEG	NEG	NEG	NEG	NEG	NEG	NEG	NEG	NEG	NEG	NEG	NEG	NEG	NEG	NEG	NEG	NEG
204226	ST0425	ST425-MSSA	NEG	NEG	NEG	NEG	NEG	NEG	NEG	POS	NEG	NEG	NEG	NEG	NEG	NEG	NEG	NEG	NEG	NEG	NEG	NEG	NEG	NEG	NEG	NEG	NEG	NEG	NEG
204906	ST0425	ST425-MSSA	NEG	NEG	NEG	NEG	NEG	NEG	NEG	POS	NEG	NEG	NEG	NEG	NEG	NEG	NEG	NEG	NEG	NEG	NEG	NEG	NEG	NEG	NEG	NEG	NEG	NEG	NEG
204908	ST0425	ST425-MSSA	NEG	NEG	NEG	NEG	NEG	NEG	NEG	POS	NEG	NEG	NEG	NEG	NEG	NEG	NEG	NEG	NEG	NEG	NEG	NEG	NEG	AMB	NEG	NEG	NEG	NEG	NEG
206170	ST0425	ST425-MSSA	NEG	NEG	NEG	NEG	NEG	NEG	NEG	POS	NEG	NEG	NEG	NEG	NEG	NEG	NEG	NEG	NEG	NEG	NEG	NEG	NEG	NEG	NEG	NEG	NEG	NEG	NEG
207765	ST0425	ST425-MSSA	NEG	NEG	NEG	NEG	NEG	NEG	NEG	POS	NEG	NEG	NEG	NEG	NEG	NEG	NEG	NEG	NEG	NEG	NEG	NEG	NEG	NEG	NEG	NEG	NEG	NEG	NEG
240048	CC599	CC599-MRSA-XI	NEG	NEG	NEG	NEG	NEG	NEG	NEG	NEG	NEG	NEG	NEG	NEG	NEG	POS	NEG	POS	NEG	NEG	NEG	NEG	POS	NEG	NEG	NEG	NEG	NEG	POS
225730	CC0692	CC692-MSSA	NEG	NEG	NEG	NEG	NEG	NEG	NEG	POS	NEG	NEG	NEG	NEG	NEG	NEG	NEG	NEG	NEG	NEG	NEG	NEG	NEG	NEG	NEG	NEG	NEG	NEG	NEG
112746	CC0692	CC692-MSSA	NEG	NEG	NEG	NEG	NEG	NEG	NEG	POS	NEG	NEG	NEG	NEG	NEG	NEG	NEG	NEG	NEG	NEG	NEG	NEG	NEG	NEG	NEG	NEG	NEG	NEG	NEG
112754	CC0692	CC692-MSSA	NEG	NEG	NEG	NEG	NEG	NEG	NEG	POS	NEG	NEG	NEG	NEG	NEG	NEG	NEG	NEG	NEG	NEG	NEG	NEG	NEG	NEG	NEG	NEG	NEG	NEG	NEG
112758	CC0692	CC692-MSSA	NEG	NEG	NEG	NEG	NEG	NEG	NEG	POS	NEG	NEG	NEG	NEG	NEG	NEG	NEG	NEG	NEG	NEG	NEG	NEG	NEG	NEG	NEG	NEG	NEG	NEG	NEG
112759	CC0692	CC692-MSSA	NEG	NEG	NEG	NEG	NEG	NEG	NEG	POS	NEG	NEG	NEG	NEG	NEG	NEG	NEG	NEG	NEG	NEG	NEG	NEG	NEG	NEG	NEG	NEG	NEG	NEG	NEG
124745	CC0692	CC692-MSSA	NEG	NEG	NEG	NEG	NEG	NEG	NEG	POS	NEG	NEG	NEG	NEG	NEG	NEG	NEG	NEG	NEG	NEG	NEG	NEG	NEG	NEG	NEG	NEG	NEG	NEG	NEG
170952	CC0692	CC692-MSSA	NEG	NEG	NEG	NEG	NEG	NEG	NEG	POS	NEG	NEG	NEG	NEG	NEG	NEG	NEG	NEG	NEG	NEG	NEG	NEG	NEG	NEG	NEG	NEG	NEG	NEG	NEG
170953	CC0692	CC692-MSSA	NEG	NEG	NEG	NEG	NEG	NEG	NEG	POS	NEG	NEG	NEG	NEG	NEG	NEG	NEG	NEG	NEG	NEG	NEG	NEG	NEG	NEG	NEG	NEG	NEG	NEG	NEG
170970	CC0692	CC692-MSSA	NEG	NEG	NEG	NEG	NEG	NEG	NEG	POS	NEG	NEG	NEG	NEG	NEG	NEG	NEG	NEG	NEG	NEG	NEG	NEG	NEG	NEG	NEG	NEG	NEG	NEG	NEG
170950	CC0707	CC707-MSSA	NEG	NEG	NEG	NEG	NEG	NEG	NEG	NEG	NEG	NEG	NEG	NEG	NEG	NEG	NEG	NEG	NEG	NEG	NEG	NEG	NEG	NEG	NEG	NEG	NEG	NEG	NEG
124948	ST0890	ST890-MSSA	NEG	NEG	NEG	NEG	NEG	NEG	NEG	NEG	NEG	NEG	NEG	NEG	NEG	NEG	NEG	NEG	NEG	NEG	NEG	NEG	NEG	NEG	NEG	NEG	NEG	NEG	NEG
220618	CC1956	CC1956-MSSA	NEG	NEG	NEG	NEG	NEG	NEG	NEG	POS	NEG	NEG	NEG	NEG	NEG	NEG	NEG	NEG	NEG	NEG	NEG	NEG	NEG	NEG	NEG	NEG	NEG	NEG	NEG
112742	ST2425	ST2425-MSSA	NEG	NEG	NEG	NEG	NEG	NEG	NEG	POS	NEG	NEG	NEG	NEG	NEG	POS	AMB	NEG	NEG	NEG	NEG	NEG	POS	NEG	NEG	NEG	NEG	NEG	POS
170957	ST2425	ST2425-MSSA	NEG	NEG	NEG	NEG	NEG	NEG	NEG	POS	NEG	NEG	NEG	NEG	NEG	POS	NEG	POS	NEG	NEG	NEG	NEG	POS	NEG	NEG	NEG	NEG	NEG	POS
170947	ST2691	ST2691-MSSA	NEG	NEG	NEG	NEG	NEG	NEG	NEG	NEG	NEG	NEG	NEG	NEG	NEG	NEG	NEG	NEG	NEG	NEG	NEG	NEG	NEG	NEG	NEG	NEG	NEG	NEG	NEG
170961	ST2691	ST2691-MSSA	NEG	NEG	NEG	NEG	NEG	NEG	NEG	NEG	NEG	NEG	NEG	NEG	NEG	NEG	NEG	NEG	NEG	NEG	NEG	NEG	NEG	NEG	NEG	NEG	NEG	NEG	NEG
112753	CC2767	ST2279-MSSA	NEG	NEG	NEG	NEG	NEG	NEG	NEG	NEG	NEG	NEG	NEG	NEG	NEG	NEG	NEG	NEG	NEG	NEG	NEG	NEG	NEG	NEG	NEG	NEG	NEG	NEG	NEG
112761	CC2767	ST2279-MSSA	NEG	NEG	NEG	NEG	NEG	NEG	NEG	NEG	NEG	NEG	NEG	NEG	NEG	NEG	NEG	NEG	NEG	NEG	NEG	NEG	NEG	NEG	NEG	NEG	NEG	NEG	NEG
228651	ST2963	ST2963-MSSA	NEG	NEG	NEG	NEG	NEG	NEG	NEG	POS	NEG	NEG	NEG	NEG	NEG	NEG	NEG	NEG	NEG	NEG	NEG	NEG	NEG	NEG	NEG	NEG	NEG	NEG	NEG
203244	ST3237	ST3237-MSSA	NEG	NEG	NEG	NEG	NEG	NEG	NEG	NEG	NEG	NEG	NEG	NEG	NEG	NEG	NEG	NEG	NEG	NEG	NEG	NEG	NEG	NEG	NEG	NEG	NEG	NEG	NEG

ISOLATE ID	CLONAL COMPLEX (by array)	STRAIN AFFILIATION (by array)	VIRULENCE : ENTEROTOXINS												VIRULENCE : HLG AND LEUKOCIDINS				VIRULENCE : LEUKOCIDINS						VIRULENCE : LEUKOCIDINS		
			enterotoxin G	enterotoxin I	enterotoxin M	enterotoxin N		enterotoxin O	Enterotoxin U and/or Y	egc cluster	enterotoxin Q	enterotoxin R	enterotoxin-like protein ORF CM14		haemolysin gamma / leukocidin, component B	haemolysin gamma / leukocidin, component C		haemolysin gamma, component A	Panton Valentine leukocidin F component	Panton Valentine leukocidin S component	F component from hypothetical leukocidin from ruminants	S component from hypothetical leukocidin from ruminants	leukocidin D component	leukocidin E component	leukocidin/ haemolysin toxin family protein	leukocidin/haemolysin toxin family protein	
			entG	entI	entM	entN (cons)	entN (other than RF122)	entO	entU	egc (total)	entQ	entR	entCM14 probe1	entCM14 probe2	lukF	lukS	lukS (ST22+ST45)	hlgA	lukF-PV	lukS-PV	lukF-PV (P83)	lukM	lukD	lukE	lukX	lukY	lukY (ST30+ST45)
203228	CC0001	CC1-MSSA	NEG	NEG	NEG	NEG	NEG	NEG	NEG	NEG	NEG	NEG	NEG	NEG	POS	POS	POS	POS	NEG	NEG	POS	NEG	POS	POS	POS	POS	NEG
203229	CC0001	CC1-MSSA	NEG	NEG	NEG	NEG	NEG	NEG	NEG	NEG	NEG	NEG	NEG	NEG	POS	POS	POS	POS	NEG	NEG	POS	NEG	POS	POS	POS	POS	NEG
203233	CC0001	CC1-MSSA	NEG	NEG	NEG	NEG	NEG	NEG	NEG	NEG	NEG	NEG	NEG	NEG	POS	POS	POS	POS	NEG	NEG	NEG	NEG	POS	POS	POS	POS	NEG
220334	CC0001	CC1-MSSA	NEG	NEG	NEG	NEG	NEG	NEG	NEG	NEG	NEG	NEG	NEG	NEG	POS	POS	POS	POS	NEG	NEG	NEG	NEG	POS	POS	POS	POS	NEG
223621	CC0001	CC1-MSSA	NEG	NEG	NEG	NEG	NEG	NEG	NEG	NEG	NEG	NEG	NEG	NEG	POS	POS	POS	POS	NEG	NEG	POS	NEG	POS	POS	POS	POS	NEG
289123	CC0001	CC1-MRSA-IV, WA MRSA-1/57	NEG	NEG	NEG	NEG	NEG	NEG	NEG	NEG	NEG	NEG	NEG	NEG	POS	POS	POS	POS	NEG	NEG	NEG	NEG	POS	POS	POS	POS	NEG
289124	CC0001	CC1-MRSA-IV, WA MRSA-1/57	NEG	NEG	NEG	NEG	NEG	NEG	NEG	NEG	NEG	NEG	NEG	NEG	POS	POS	POS	POS	NEG	NEG	NEG	NEG	POS	POS	POS	POS	NEG
275170	CC0005	CC5-MSSA	POS	POS	POS	POS	POS	POS	POS	POS	NEG	NEG	NEG	NEG	POS	POS	NEG	POS	NEG	NEG	NEG	NEG	POS	POS	POS	POS	NEG
275171	CC0005	CC5-MSSA	POS	POS	POS	POS	POS	POS	POS	POS	NEG	NEG	NEG	NEG	POS	POS	NEG	POS	NEG	NEG	NEG	NEG	POS	POS	AMB	POS	NEG
112747	CC0005	CC5-MSSA	POS	POS	POS	POS	POS	POS	POS	POS	NEG	NEG	NEG	NEG	POS	POS	AMB	POS	NEG	NEG	NEG	NEG	POS	POS	POS	POS	NEG
112752	CC0006	CC6-MSSA	NEG	NEG	NEG	NEG	NEG	NEG	NEG	NEG	NEG	NEG	NEG	NEG	POS	POS	AMB	POS	NEG	NEG	NEG	NEG	POS	POS	POS	POS	NEG
275178	CC0007	CC7-MSSA	NEG	NEG	NEG	NEG	NEG	NEG	NEG	NEG	NEG	NEG	NEG	NEG	POS	NEG	POS	POS	NEG	NEG	NEG	NEG	POS	POS	POS	POS	NEG
275166	CC0008	CC8-MSSA	NEG	NEG	NEG	NEG	NEG	NEG	NEG	NEG	NEG	POS	NEG	NEG	POS	POS	NEG	POS	NEG	NEG	NEG	NEG	POS	POS	POS	POS	NEG
275176	CC0008	CC8-MSSA	NEG	NEG	NEG	NEG	NEG	NEG	NEG	NEG	NEG	POS	NEG	NEG	POS	POS	NEG	POS	NEG	NEG	NEG	NEG	POS	POS	POS	POS	NEG
275177	CC0008	CC8-MSSA	NEG	NEG	NEG	NEG	NEG	NEG	NEG	NEG	NEG	NEG	NEG	NEG	POS	POS	NEG	POS	NEG	NEG	NEG	NEG	POS	POS	POS	POS	NEG
275167	CC0009	CC9-MSSA	POS	POS	POS	POS	POS	POS	POS	POS	NEG	NEG	NEG	NEG	POS	POS	NEG	POS	NEG	NEG	NEG	NEG	NEG	NEG	POS	POS	NEG
170968	CC0012	CC12-MSSA	NEG	NEG	NEG	NEG	NEG	NEG	NEG	NEG	NEG	NEG	POS	POS	POS	POS	AMB	POS	NEG	NEG	NEG	NEG	POS	POS	POS	POS	NEG
275175	CC0015	CC15-MSSA	NEG	NEG	NEG	NEG	NEG	NEG	NEG	NEG	NEG	NEG	NEG	NEG	POS	POS	NEG	POS	NEG	NEG	NEG	NEG	POS	POS	POS	POS	NEG
170949	CC0015	CC15-MSSA	NEG	NEG	NEG	NEG	NEG	NEG	NEG	NEG	NEG	NEG	NEG	NEG	POS	POS	AMB	POS	NEG	NEG	NEG	NEG	POS	AMB	POS	POS	NEG
228658	CC0022	CC22-MSSA	POS	POS	POS	POS	POS	POS	POS	POS	NEG	NEG	NEG	NEG	POS	POS	POS	POS	NEG	NEG	NEG	NEG	NEG	NEG	POS	POS	NEG
289125	CC0022	CC22-MRSA-IV (PVL+)	POS	POS	POS	POS	POS	POS	POS	NEG	NEG	POS	NEG	NEG	POS	POS	POS	POS	POS	POS	NEG	NEG	NEG	NEG	POS	POS	NEG
289126	CC0022	CC22-MRSA-IV (PVL+)	POS	POS	POS	POS	POS	POS	POS	NEG	NEG	POS	NEG	NEG	POS	POS	POS	POS	POS	POS	NEG	NEG	NEG	NEG	POS	POS	NEG
289127	CC0022	CC22-MRSA-IV (PVL+)	POS	POS	POS	POS	POS	POS	POS	NEG	NEG	POS	NEG	NEG	POS	POS	POS	POS	POS	POS	NEG	NEG	NEG	NEG	POS	POS	NEG
131939	CC0025	CC25-MSSA	POS	POS	POS	POS	POS	POS	POS	POS	NEG	NEG	NEG	NEG	POS	POS	AMB	POS	NEG	NEG	NEG	NEG	POS	POS	POS	POS	NEG
275165	CC0030	CC30-MSSA (lukF-P83/lukM+)	POS	POS	POS	POS	POS	POS	POS	POS	NEG	NEG	NEG	NEG	POS	POS	AMB	POS	NEG	NEG	POS	POS	NEG	NEG	POS	NEG	POS
275741	CC0030	CC30-MSSA	POS	POS	POS	POS	POS	POS	POS	POS	NEG	NEG	NEG	NEG	POS	POS	NEG	POS	NEG	NEG	NEG	NEG	NEG	NEG	POS	NEG	POS
124492	CC0049	CC49-MSSA (lukF-P83/lukM+)	NEG	NEG	NEG	NEG	NEG	NEG	NEG	NEG	NEG	NEG	NEG	NEG	POS	POS	NEG	POS	NEG	NEG	POS	POS	POS	POS	AMB	POS	NEG
220616	CC0049	CC49-MSSA (lukF-P83/lukM+)	NEG	NEG	NEG	NEG	NEG	NEG	NEG	NEG	NEG	NEG	NEG	NEG	POS	POS	AMB	POS	NEG	NEG	POS	POS	POS	POS	POS	POS	NEG
228649	CC0049	CC49-MSSA (lukF-P83/lukM+)	NEG	NEG	NEG	NEG	NEG	NEG	NEG	NEG	NEG	NEG	NEG	NEG	POS	POS	POS	POS	NEG	NEG	POS	POS	POS	POS	POS	POS	NEG
190994	CC0059	CC59-MSSA	NEG	NEG	NEG	NEG	NEG	NEG	NEG	NEG	NEG	NEG	NEG	NEG	POS	POS	NEG	POS	NEG	NEG	NEG	NEG	NEG	NEG	NEG	NEG	NEG
275172	CC0088	CC88-MSSA	NEG	NEG	NEG	NEG	NEG	NEG	NEG	NEG	NEG	NEG	NEG	NEG	POS	POS	NEG	POS	NEG	NEG	NEG	NEG	POS	POS	AMB	POS	NEG
275173	CC0088	CC88-MSSA	NEG	NEG	NEG	NEG	NEG	NEG	NEG	NEG	NEG	NEG	NEG	NEG	POS	POS	NEG	POS	NEG	NEG	NEG	NEG	POS	POS	POS	POS	NEG
275174	CC0088	CC88-MSSA	NEG	NEG	NEG	NEG	NEG	NEG	NEG	NEG	NEG	NEG	NEG	NEG	POS	POS	AMB	POS	NEG	NEG	NEG	NEG	POS	POS	POS	POS	NEG
170971	CC0097	CC97-MSSA	NEG	NEG	NEG	NEG	NEG	NEG	NEG	NEG	NEG	NEG	NEG	NEG	POS	POS	AMB	POS	NEG	NEG	NEG	NEG	POS	POS	POS	POS	NEG
112745	CC0097	CC97-MSSA	NEG	NEG	NEG	NEG	NEG	NEG	NEG	NEG	NEG	NEG	NEG	NEG	POS	POS	AMB	POS	NEG	NEG	NEG	NEG	POS	POS	POS	POS	NEG
112748	CC0097	CC97-MSSA	NEG	NEG	NEG	NEG	NEG	NEG	NEG	NEG	NEG	NEG	NEG	NEG	POS	POS	AMB	POS	NEG	NEG	NEG	NEG	POS	POS	POS	POS	NEG
112750	CC0097	CC97-MSSA	NEG	NEG	NEG	NEG	NEG	NEG	NEG	NEG	NEG	NEG	NEG	NEG	POS	POS	AMB	POS	NEG	NEG	NEG	NEG	POS	POS	POS	POS	NEG
112762	CC0097	CC97-MSSA	NEG	NEG	NEG	NEG	NEG	NEG	NEG	NEG	NEG	NEG	NEG	NEG	POS	POS	AMB	POS	NEG	NEG	NEG	NEG	POS	POS	POS	POS	NEG
112763	CC0097	CC97-MSSA	NEG	NEG	NEG	NEG	NEG	NEG	NEG	NEG	NEG	NEG	NEG	NEG	POS	POS	AMB	POS	NEG	NEG	NEG	NEG	POS	POS	POS	POS	NEG
112764	CC0097	CC97-MSSA	NEG	NEG	NEG	NEG	NEG	NEG	NEG	NEG	NEG	NEG	NEG	NEG	POS	POS	AMB	POS	NEG	NEG	NEG	NEG	POS	POS	POS	POS	NEG
170945	CC0097	CC97-MSSA	NEG	NEG	NEG	NEG	NEG	NEG	NEG	NEG	NEG	NEG	NEG	NEG	POS	POS	AMB	POS	NEG	NEG	NEG	NEG	POS	POS	POS	POS	NEG
170954	CC0097	CC97-MSSA	NEG	NEG	NEG	NEG	NEG	NEG	NEG	NEG	NEG	NEG	NEG	NEG	POS	POS	AMB	POS	NEG	NEG	NEG	NEG	POS	POS	POS	POS	NEG
170958	CC0097	CC97-MSSA	NEG	NEG	NEG	NEG	NEG	NEG	NEG	NEG	NEG	NEG	NEG	NEG	POS	POS	NEG	POS	NEG	NEG	NEG	NEG	POS	POS	AMB	POS	NEG
170963	CC0097	CC97-MSSA	NEG	NEG	NEG	NEG	NEG	NEG	NEG	NEG	NEG	NEG	NEG	NEG	POS	POS	NEG	POS	NEG	NEG	NEG	NEG	POS	POS	AMB	POS	NEG
170964	CC0097	CC97-MSSA	NEG	NEG	NEG	NEG	NEG	NEG	NEG	NEG	NEG	NEG	NEG	NEG	POS	POS	AMB	POS	NEG	NEG	NEG	NEG	POS	POS	AMB	POS	NEG
170965	CC0097	CC97-MSSA	NEG	NEG	NEG	NEG	NEG	NEG	NEG	NEG	NEG	NEG	NEG	NEG	POS	POS	NEG	POS	NEG	NEG	NEG	NEG	POS	POS	AMB	POS	NEG
170966	CC0097	CC97-MSSA	NEG	NEG	NEG	NEG	NEG	NEG	NEG	NEG	NEG	NEG	NEG	NEG	POS	POS	NEG	POS	NEG	NEG	NEG	NEG	POS	POS	POS	POS	NEG
170974	CC0097	CC97-MSSA	NEG	NEG	NEG	NEG	NEG	NEG	NEG	NEG	NEG	NEG	NEG	NEG	POS	POS	NEG	POS	NEG	NEG	NEG	NEG	POS	POS	AMB	POS	NEG
170976	CC0097	CC97-MSSA	NEG	NEG	NEG	NEG	NEG	NEG	NEG	NEG	NEG	NEG	NEG	NEG	POS	POS	NEG	POS	NEG	NEG	NEG	NEG	POS	POS	AMB	POS	NEG
112744	CC0097	CC97-MSSA	NEG	NEG	NEG	NEG	NEG	NEG	NEG	NEG	NEG	NEG	NEG	NEG	POS	POS	AMB	POS	NEG	NEG	NEG	NEG	POS	POS	POS	POS	NEG
112749	CC0097	CC97-MSSA	NEG	NEG	NEG	NEG	NEG	NEG	NEG	NEG	NEG	NEG	NEG	NEG	POS	POS	AMB	POS	NEG	NEG	NEG	NEG	POS	POS	POS	POS	NEG
112757	CC0097	CC97-MSSA	NEG	NEG	NEG	NEG	NEG	NEG	NEG	NEG	NEG	NEG	NEG	NEG	POS	POS	AMB	POS	NEG	NEG	NEG	NEG	POS	POS	POS	POS	NEG
170973	CC0097	CC97-MSSA	NEG	NEG	NEG	NEG	NEG	NEG	NEG	NEG	NEG	NEG	NEG	NEG	POS	POS	NEG	POS	NEG	NEG	NEG	NEG	POS	POS	AMB	POS	NEG
207756	CC0097	CC97-MSSA	NEG	NEG	NEG	NEG	NEG	NEG	NEG	NEG	NEG	NEG	NEG	NEG	POS	POS	POS	POS	NEG	NEG	NEG	NEG	POS	POS	POS	POS	NEG
206171	CC0097	CC97-MSSA	NEG	NEG	NEG	NEG	NEG	NEG	NEG	NEG	NEG	NEG	NEG	NEG	POS	POS	AMB	POS	NEG	NEG	NEG	NEG	POS	POS	POS	POS	NEG
275169	CC0097	CC97-MSSA-[ccrAA]	NEG	NEG	NEG	NEG	NEG	NEG	NEG	NEG	NEG	NEG	NEG	NEG	POS	POS	NEG	POS	NEG	NEG	NEG	NEG	POS	POS	POS	POS	NEG
275168	CC0097	CC97-MSSA-[ccrAA]	NEG	NEG	NEG	NEG	NEG	NEG	NEG	NEG	NEG	NEG	NEG	NEG	POS	POS	NEG	POS	NEG	NEG	NEG	NEG	POS	POS	POS	POS	NEG
118320	CC0130	CC130-MSSA (lukF-P83/M+)	NEG	NEG	NEG	NEG	NEG	NEG	NEG	NEG	NEG	NEG	NEG	NEG	POS	POS	POS	POS	NEG	NEG	POS	POS	POS	AMB	POS	POS	NEG
203236	CC0130	CC130-MRSA-XI	NEG	NEG	NEG	NEG	NEG	NEG	NEG	NEG	NEG	NEG	NEG	NEG	POS	POS	POS	POS	NEG	NEG	NEG	NEG	POS	POS	POS	POS	NEG
204204	CC0130	CC130-MRSA-XI	NEG	NEG	NEG	NEG	NEG	NEG	NEG	NEG	NEG	NEG	NEG	NEG	POS	POS	POS	POS	NEG	NEG	NEG	NEG	POS	NEG	POS	POS	NEG
289122	CC0130	CC130-MRSA-XI	NEG	NEG	NEG	NEG	NEG	NEG	NEG	NEG	NEG	NEG	NEG	NEG	POS	POS	POS	POS	NEG	NEG	NEG	NEG	POS	NEG	POS	POS	NEG
225779	CC0130	CC130-MRSA-XI	NEG	NEG	NEG	NEG	NEG	NEG	NEG	NEG	NEG	NEG	NEG	NEG	POS	POS	POS	POS	NEG	NEG	NEG	NEG	POS	AMB	POS	POS	NEG
225780	CC0130	CC130-MRSA-XI	NEG	NEG	NEG	NEG	NEG	NEG	NEG	NEG	NEG	NEG	NEG	NEG	POS	POS	POS	POS	NEG	NEG	NEG	NEG	POS	NEG	POS	POS	NEG
170959	CC0130	CC130-MRSA-XI	NEG	NEG	NEG	NEG	NEG	NEG	NEG	NEG	NEG	NEG	NEG	NEG	POS	POS	NEG	POS	NEG	NEG	NEG	NEG	POS	NEG	POS	POS	NEG
179029	CC0130	CC130-MRSA-XI	NEG	NEG	NEG	NEG	NEG	NEG	NEG	NEG	NEG	NEG	NEG	NEG	POS	POS	AMB	POS	NEG	NEG	NEG	NEG	POS	0	POS	POS	NEG
116254	CC0133	CC133-MSSA	NEG	NEG	NEG	NEG	NEG	NEG	NEG	NEG	NEG	NEG	NEG	NEG	POS	POS	NEG	POS	NEG	NEG	NEG	NEG	POS	NEG	POS	POS	NEG
207761	CC0133	CC133-MSSA	NEG	NEG	NEG	NEG	NEG	NEG	NEG	NEG	NEG	NEG	NEG	NEG	POS	POS	NEG	POS	NEG	NEG	NEG	NEG	POS	NEG	POS	POS	NEG
213103	CC0133	CC133-MSSA	NEG	NEG	NEG	NEG	NEG	NEG	NEG	NEG	NEG	NEG	NEG	NEG	POS	POS	POS	POS	NEG	NEG	AMB	NEG	POS	AMB	POS	POS	NEG
275736	CC0133	CC133-MSSA	NEG	NEG	NEG	NEG	NEG	NEG	NEG	NEG	NEG	NEG	NEG	NEG	POS	POS	NEG	POS	NEG	NEG	NEG	NEG	POS	NEG	POS	POS	NEG
275737	CC0133	CC133-MSSA	NEG	NEG	NEG	NEG	NEG	NEG	NEG	NEG	NEG	NEG	NEG	NEG	POS	POS	NEG	POS	NEG	NEG	NEG	NEG	POS	NEG	POS	POS	NEG
275738	CC0133	CC133-MSSA	NEG	NEG	NEG	NEG	NEG	NEG	NEG	NEG	NEG	NEG	NEG	NEG	POS	POS	NEG	POS	NEG	NEG	NEG	NEG	POS	NEG	POS	POS	NEG
275739	CC0133	CC133-MSSA	NEG	NEG	NEG	NEG	NEG	NEG	NEG	NEG	NEG	NEG	NEG	NEG	POS	POS	NEG	POS	NEG	NEG	NEG	NEG	POS	NEG	POS	POS	NEG
112755	CC0133	CC133-MSSA	NEG	NEG	NEG	NEG	NEG	NEG	NEG	NEG	NEG	NEG	NEG	NEG	POS	POS	AMB	POS	NEG	NEG	NEG	NEG	POS	NEG	POS	POS	NEG
225778	CC0398	CC398-MRSA-V/VT	NEG	NEG	NEG	NEG	NEG	NEG	NEG	NEG	NEG	NEG	NEG	NEG	POS	POS	POS	POS	NEG	NEG	NEG	NEG	NEG	NEG	POS	POS	NEG
123159	ST0425	ST425-MSSA	NEG	NEG	NEG	NEG	NEG	NEG	NEG	NEG	NEG	NEG	NEG	NEG	POS	POS	NEG	POS	NEG	NEG	NEG	NEG	POS	NEG	POS	POS	NEG
86991	ST0425	ST425-MSSA	NEG	NEG	NEG	NEG	NEG	NEG	NEG	NEG	NEG	NEG	NEG	NEG	POS	POS	NEG	POS	NEG	NEG	NEG	NEG	POS	NEG	POS	POS	NEG
112756	ST0425	ST425-MSSA	NEG	NEG	NEG	NEG	NEG	NEG	NEG	NEG	NEG	NEG	NEG	NEG	POS	POS	AMB	POS	NEG	NEG	NEG	NEG	POS	NEG	POS	POS	NEG
195207	ST0425	ST425-MSSA	NEG	NEG	NEG	NEG	NEG	NEG	NEG	NEG	NEG	NEG	NEG	NEG	POS	POS	AMB	POS	NEG	NEG	NEG	NEG	POS	NEG	POS	POS	NEG
223609	ST0425	ST425-MSSA	NEG	NEG	NEG	NEG	NEG	NEG	NEG	NEG	NEG	NEG	NEG	NEG	POS	POS	POS	POS	NEG	NEG	POS	NEG	POS	NEG	POS	POS	NEG
275740	ST0425	ST425-MSSA	NEG	NEG	NEG	NEG	NEG	NEG	NEG	NEG	NEG	NEG	NEG	NEG	POS	POS	AMB	POS	NEG	NEG	NEG	NEG	POS	NEG	POS	POS	NEG
174889	ST0425	ST425-MSSA	NEG	NEG	NEG	NEG	NEG	NEG	NEG	NEG	NEG	NEG	NEG	NEG	POS	POS	AMB	POS	NEG	NEG	NEG	NEG	POS	NEG	POS	POS	NEG
203230	ST0425	ST425-MSSA	NEG	NEG	NEG	NEG	NEG	NEG	NEG	NEG	NEG	NEG	NEG	NEG	POS	POS	POS	POS	NEG	NEG	AMB	NEG	POS	AMB	POS	POS	NEG
203242	ST0425	ST425-MSSA	NEG	NEG	NEG	NEG	NEG	NEG	NEG	NEG	NEG	NEG	NEG	NEG	POS	POS	POS	POS	NEG	NEG	AMB	NEG	POS	AMB	POS	POS	NEG
203243	ST0425	ST425-MSSA	NEG	NEG	NEG	NEG	NEG	NEG	NEG	NEG	NEG	NEG	NEG	NEG	POS	POS	POS	POS	NEG	NEG	AMB	NEG	POS	AMB	POS	POS	NEG
207951	ST0425	ST425-MSSA	NEG	NEG	NEG	NEG	NEG	NEG	NEG	NEG	NEG	NEG	NEG	NEG	POS	POS	NEG	POS	NEG	NEG	NEG	NEG	POS	NEG	AMB	POS	NEG
207981	ST0425	ST425-MSSA	NEG	NEG	NEG	NEG	NEG	NEG	NEG	NEG	NEG	NEG	NEG	NEG	POS	POS	NEG	POS	NEG	NEG	NEG	NEG	POS	NEG	POS	AMB	NEG
195212	ST0425	ST425-MSSA	NEG	NEG	NEG	NEG	NEG	NEG	NEG	NEG	NEG	NEG	NEG	NEG	POS	POS	POS	POS	NEG	NEG	NEG	NEG	POS	NEG	POS	POS	NEG
195206	ST0425	ST425-MSSA	NEG	NEG	NEG	NEG	NEG	NEG	NEG	NEG	NEG	NEG	NEG	NEG	POS	POS	POS	POS	NEG	NEG	NEG	NEG	POS	POS	POS	POS	NEG
195202	ST0425	ST425-MSSA	NEG	NEG	NEG	NEG	NEG	NEG	NEG	NEG	NEG	NEG	NEG	NEG	POS	POS	POS	POS	NEG	NEG	NEG	NEG	POS	NEG	POS	POS	NEG
216042	ST0425	ST425-MSSA	NEG	NEG	NEG	NEG	NEG	NEG	NEG	NEG	NEG	NEG	NEG	NEG	POS	POS	POS	POS	NEG	NEG	NEG	NEG	POS	POS	POS	POS	NEG
223608	ST0425	ST425-MSSA	NEG	NEG	NEG	NEG	NEG	NEG	NEG	NEG	NEG	NEG	AMB	NEG	POS	POS	POS	POS	NEG	NEG	POS	NEG	POS	NEG	POS	POS	NEG
223612	ST0425	ST425-MSSA	NEG	NEG	NEG	NEG	NEG	NEG	NEG	NEG	NEG	NEG	NEG	NEG	POS	POS	POS	POS	NEG	NEG	POS	NEG	POS	NEG	POS	POS	NEG
225733	ST0425	ST425-MSSA	NEG	NEG	NEG	NEG	NEG	NEG	NEG	NEG	NEG	NEG	NEG	NEG	POS	POS	POS	POS	NEG	NEG	NEG	NEG	POS	NEG	POS	POS	NEG
230321	ST0425	ST425-MSSA	NEG	NEG	NEG	NEG	NEG	NEG	NEG	NEG	NEG	NEG	NEG	NEG	POS	POS	POS	POS	NEG	NEG	NEG	NEG	POS	AMB	POS	POS	NEG
232368	ST0425	ST425-MSSA	NEG	NEG	NEG	NEG	NEG	NEG	NEG	NEG	NEG	NEG	NEG	NEG	POS	POS	POS	POS	NEG	NEG	NEG	NEG	POS	NEG	POS	POS	NEG
204215	ST0425	ST425-MSSA	NEG	NEG	NEG	NEG	NEG	NEG	NEG	NEG	NEG	NEG	NEG	NEG	POS	POS	POS	POS	NEG	NEG	NEG	NEG	POS	AMB	POS	POS	NEG
204218	ST0425	ST425-MSSA	NEG	NEG	NEG	NEG	NEG	NEG	NEG	NEG	NEG	NEG	NEG	NEG	POS	POS	POS	POS	NEG	NEG	AMB	NEG	POS	AMB	POS	POS	NEG
204222	ST0425	ST425-MSSA	NEG	NEG	NEG	NEG	NEG	NEG	NEG	NEG	NEG	NEG	NEG	NEG	POS	POS	POS	POS	NEG	NEG	NEG	NEG	POS	AMB	POS	POS	NEG
204226	ST0425	ST425-MSSA	NEG	NEG	NEG	NEG	NEG	NEG	NEG	NEG	NEG	NEG	NEG	NEG	POS	POS	POS	POS	NEG	NEG	NEG	NEG	POS	NEG	POS	POS	NEG
204906	ST0425	ST425-MSSA	NEG	NEG	NEG	NEG	NEG	NEG	NEG	NEG	NEG	NEG	NEG	NEG	POS	POS	POS	POS	NEG	NEG	NEG	NEG	POS	NEG	POS	POS	NEG
204908	ST0425	ST425-MSSA	NEG	NEG	NEG	NEG	NEG	NEG	NEG	NEG	NEG	NEG	NEG	NEG	POS	POS	POS	POS	NEG	NEG	AMB	NEG	POS	AMB	POS	POS	NEG
206170	ST0425	ST425-MSSA	NEG	NEG	NEG	NEG	NEG	NEG	NEG	NEG	NEG	NEG	NEG	NEG	POS	POS	AMB	POS	NEG	NEG	NEG	NEG	POS	NEG	POS	POS	NEG
207765	ST0425	ST425-MSSA	NEG	NEG	NEG	NEG	NEG	NEG	NEG	NEG	NEG	NEG	NEG	NEG	POS	POS	AMB	POS	NEG	NEG	NEG	NEG	POS	NEG	POS	POS	NEG
240048	CC599	CC599-MRSA-XI	NEG	NEG	NEG	NEG	NEG	NEG	NEG	NEG	NEG	NEG	NEG	NEG	POS	POS	AMB	POS	NEG	NEG	NEG	POS	POS	POS	POS	POS	NEG
225730	CC0692	CC692-MSSA	NEG	NEG	NEG	NEG	NEG	NEG	NEG	NEG	NEG	NEG	NEG	NEG	POS	POS	POS	POS	NEG	NEG	NEG	NEG	POS	POS	POS	POS	NEG
112746	CC0692	CC692-MSSA	NEG	NEG	NEG	NEG	NEG	NEG	NEG	NEG	NEG	NEG	NEG	NEG	POS	POS	AMB	POS	NEG	NEG	NEG	NEG	POS	POS	POS	POS	NEG
112754	CC0692	CC692-MSSA	NEG	NEG	NEG	NEG	NEG	NEG	NEG	NEG	NEG	NEG	NEG	NEG	POS	POS	NEG	POS	NEG	NEG	NEG	NEG	POS	POS	POS	POS	NEG
112758	CC0692	CC692-MSSA	NEG	NEG	NEG	NEG	NEG	NEG	NEG	NEG	NEG	NEG	NEG	NEG	POS	POS	AMB	POS	NEG	NEG	NEG	NEG	POS	POS	POS	POS	NEG
112759	CC0692	CC692-MSSA	NEG	NEG	NEG	NEG	NEG	NEG	NEG	NEG	NEG	NEG	NEG	NEG	POS	POS	AMB	POS	NEG	NEG	NEG	NEG	POS	POS	POS	POS	NEG
124745	CC0692	CC692-MSSA	NEG	NEG	NEG	NEG	NEG	NEG	NEG	NEG	NEG	NEG	NEG	NEG	POS	POS	AMB	POS	NEG	NEG	NEG	NEG	POS	POS	POS	POS	NEG
170952	CC0692	CC692-MSSA	NEG	NEG	NEG	NEG	NEG	NEG	NEG	NEG	NEG	NEG	NEG	NEG	POS	POS	POS	POS	NEG	NEG	NEG	NEG	POS	POS	POS	POS	NEG
170953	CC0692	CC692-MSSA	NEG	NEG	NEG	NEG	NEG	NEG	NEG	NEG	NEG	NEG	NEG	NEG	POS	POS	AMB	POS	NEG	NEG	NEG	NEG	POS	POS	POS	POS	NEG
170970	CC0692	CC692-MSSA	NEG	NEG	NEG	NEG	NEG	NEG	NEG	NEG	NEG	NEG	NEG	NEG	POS	POS	AMB	POS	NEG	NEG	NEG	NEG	POS	POS	POS	POS	NEG
170950	CC0707	CC707-MSSA	NEG	NEG	NEG	NEG	NEG	NEG	NEG	NEG	NEG	NEG	POS	POS	POS	POS	AMB	POS	NEG	NEG	NEG	NEG	AMB	POS	POS	POS	NEG
124948	ST0890	ST890-MSSA	NEG	NEG	NEG	NEG	NEG	NEG	NEG	NEG	NEG	NEG	NEG	NEG	POS	POS	NEG	POS	NEG	NEG	NEG	NEG	NEG	POS	NEG	NEG	NEG
220618	CC1956	CC1956-MSSA	NEG	NEG	NEG	NEG	NEG	NEG	NEG	NEG	NEG	NEG	NEG	NEG	POS	POS	POS	POS	NEG	NEG	NEG	NEG	POS	POS	POS	POS	NEG
112742	ST2425	ST2425-MSSA	NEG	NEG	NEG	NEG	NEG	NEG	NEG	NEG	NEG	NEG	POS	POS	POS	POS	AMB	POS	NEG	NEG	NEG	NEG	POS	POS	POS	POS	NEG
170957	ST2425	ST2425-MSSA	NEG	NEG	NEG	NEG	NEG	NEG	NEG	NEG	NEG	NEG	POS	POS	POS	POS	AMB	POS	NEG	NEG	NEG	NEG	POS	POS	POS	POS	NEG
170947	ST2691	ST2691-MSSA	NEG	NEG	NEG	NEG	NEG	NEG	NEG	NEG	NEG	NEG	NEG	NEG	POS	POS	AMB	POS	NEG	NEG	NEG	NEG	POS	AMB	POS	POS	NEG
170961	ST2691	ST2691-MSSA	NEG	NEG	NEG	NEG	NEG	NEG	NEG	NEG	NEG	NEG	NEG	NEG	POS	POS	AMB	POS	NEG	NEG	NEG	NEG	POS	POS	POS	POS	NEG
112753	CC2767	ST2279-MSSA	NEG	NEG	NEG	NEG	NEG	NEG	NEG	NEG	NEG	NEG	NEG	NEG	POS	POS	NEG	POS	NEG	NEG	NEG	NEG	NEG	NEG	NEG	POS	NEG
112761	CC2767	ST2279-MSSA	NEG	NEG	NEG	NEG	NEG	NEG	NEG	NEG	NEG	NEG	NEG	NEG	POS	POS	NEG	POS	NEG	NEG	NEG	NEG	POS	NEG	POS	POS	NEG
228651	ST2963	ST2963-MSSA	NEG	NEG	NEG	NEG	NEG	NEG	NEG	NEG	NEG	NEG	NEG	NEG	POS	POS	POS	POS	NEG	NEG	NEG	NEG	POS	NEG	POS	POS	NEG
203244	ST3237	ST3237-MSSA	NEG	NEG	NEG	NEG	NEG	NEG	NEG	NEG	NEG	NEG	NEG	NEG	POS	AMB	NEG	POS	NEG	NEG	NEG	NEG	POS	AMB	POS	POS	NEG

ISOLATE ID	CLONAL COMPLEX (by array)	STRAIN AFFILIATION (by array)	VIRULENCE : HAEMOLYSINS				VIRULENCE : HLB & HLB-CONV PHAGES							VIRULENCE : EXFOL.TOXINS			VIRULENCE : EPITHEL. DIFF. INHIB			VIRULENCE : ACME LOCUS					VIRULENCE : PROTEASES								VI
			putative membrane protein	haemolysin alpha	putative membrane protein		haemolysin beta				staphylokinase	chemotaxis-inhibiting protein (CHIPS)	Staphyl. Comple-ment inhibitor	exfoliative toxin serotype A	exfoliative toxin serotype B	exfoliative toxin D	epidermal cell differen-tiation inhibitor	epidermal cell differen-tiation inhibitor B	epidermal cell differen-tiation inhibitor C	ACME-locus	ACME-locus	ACME-locus: ornithincarb-amoyltrans-ferase	ACME-locus: carbamat-kinase	ACME-locus: arginine/orni-thine-antiporter	aureolysin			serin- protease A	serin- protease B	serin- protease E	glutamylendopeptidase	staphopain B, protease
			hl	hla	hlIII (cons)	hlIII (other than RF122)	hlb-probe 1	hlb-probe 2	hlb-probe 3	un-truncated hlb	sak	chp	scn	etA	etB	etD	edinA	edinB	edinC	ACME (total)	arcA-SCC	arcB-SCC	arcC-SCC	arcD-SCC	aur (cons)	aur (Other than MRSA252)	aur (MRSA252)	splA	splB	splE	sspA	sspB
203228	CC0001	CC1-MSSA	POS	POS	POS	POS	POS	POS	POS	POS	NEG	NEG	NEG	NEG	NEG	NEG	NEG	NEG	NEG	NEG	NEG	NEG	NEG	NEG	POS	POS	NEG	POS	POS	POS	POS	POS
203229	CC0001	CC1-MSSA	POS	POS	POS	POS	POS	POS	POS	POS	NEG	NEG	NEG	NEG	NEG	NEG	NEG	NEG	NEG	NEG	NEG	NEG	NEG	NEG	POS	POS	NEG	POS	POS	POS	POS	POS
203233	CC0001	CC1-MSSA	POS	POS	POS	POS	POS	POS	POS	POS	NEG	NEG	NEG	NEG	NEG	NEG	NEG	NEG	NEG	NEG	NEG	NEG	NEG	NEG	POS	POS	NEG	POS	POS	POS	POS	POS
220334	CC0001	CC1-MSSA	POS	POS	POS	POS	POS	POS	POS	POS	NEG	NEG	NEG	NEG	NEG	NEG	NEG	NEG	NEG	NEG	NEG	NEG	NEG	NEG	POS	POS	NEG	POS	POS	POS	POS	POS
223621	CC0001	CC1-MSSA	POS	POS	POS	POS	POS	POS	POS	NEG	NEG	NEG	NEG	NEG	NEG	NEG	NEG	NEG	NEG	NEG	NEG	NEG	NEG	NEG	POS	POS	NEG	POS	POS	POS	POS	POS
289123	CC0001	CC1-MRSA-IV, WA MRSA-1/57	POS	POS	POS	POS	POS	POS	POS	NEG	POS	NEG	POS	NEG	NEG	NEG	NEG	NEG	NEG	NEG	NEG	NEG	NEG	NEG	POS	POS	NEG	POS	POS	NEG	POS	POS
289124	CC0001	CC1-MRSA-IV, WA MRSA-1/57	POS	POS	POS	POS	POS	POS	POS	NEG	POS	NEG	POS	NEG	NEG	NEG	NEG	NEG	NEG	NEG	NEG	NEG	NEG	NEG	POS	POS	NEG	POS	POS	NEG	POS	POS
275170	CC0005	CC5-MSSA	POS	POS	POS	POS	POS	POS	POS	NEG	POS	POS	POS	NEG	NEG	NEG	NEG	NEG	NEG	NEG	NEG	NEG	NEG	NEG	POS	POS	NEG	POS	POS	NEG	POS	POS
275171	CC0005	CC5-MSSA	POS	POS	POS	POS	AMB	POS	NEG	NEG	POS	POS	POS	NEG	NEG	NEG	NEG	NEG	NEG	NEG	NEG	NEG	NEG	NEG	AMB	POS	NEG	POS	POS	NEG	POS	POS
112747	CC0005	CC5-MSSA	POS	POS	POS	POS	POS	POS	POS	NEG	NEG	NEG	NEG	NEG	NEG	NEG	NEG	NEG	NEG	NEG	NEG	NEG	NEG	NEG	POS	POS	NEG	POS	POS	NEG	POS	POS
112752	CC0006	CC6-MSSA	POS	POS	POS	POS	POS	POS	POS	AMB	POS	NEG	POS	NEG	NEG	NEG	NEG	NEG	NEG	NEG	NEG	NEG	NEG	NEG	POS	POS	NEG	POS	POS	POS	POS	POS
275178	CC0007	CC7-MSSA	POS	POS	POS	POS	AMB	POS	NEG	NEG	POS	NEG	POS	NEG	NEG	NEG	NEG	NEG	NEG	NEG	NEG	NEG	NEG	NEG	POS	POS	NEG	POS	POS	POS	POS	POS
275166	CC0008	CC8-MSSA	POS	POS	POS	POS	POS	POS	POS	NEG	POS	NEG	POS	NEG	NEG	NEG	NEG	NEG	NEG	NEG	NEG	NEG	NEG	NEG	POS	POS	NEG	POS	POS	POS	POS	POS
275176	CC0008	CC8-MSSA	POS	POS	POS	POS	POS	POS	POS	NEG	POS	NEG	POS	NEG	NEG	NEG	NEG	NEG	NEG	NEG	NEG	NEG	NEG	NEG	POS	POS	NEG	POS	POS	POS	POS	POS
275177	CC0008	CC8-MSSA	POS	POS	POS	POS	POS	POS	POS	NEG	POS	NEG	POS	NEG	NEG	NEG	NEG	NEG	NEG	NEG	NEG	NEG	NEG	NEG	POS	POS	NEG	POS	POS	POS	POS	POS
275167	CC0009	CC9-MSSA	POS	POS	POS	POS	POS	POS	AMB	POS	NEG	NEG	NEG	NEG	NEG	NEG	NEG	NEG	NEG	NEG	NEG	NEG	NEG	NEG	POS	POS	NEG	NEG	NEG	NEG	POS	POS
170968	CC0012	CC12-MSSA	POS	POS	POS	POS	POS	POS	POS	POS	NEG	NEG	NEG	NEG	NEG	NEG	NEG	NEG	NEG	NEG	NEG	NEG	NEG	NEG	POS	POS	NEG	POS	POS	POS	POS	POS
275175	CC0015	CC15-MSSA	POS	POS	POS	POS	NEG	NEG	NEG	NEG	NEG	POS	POS	NEG	NEG	NEG	NEG	NEG	NEG	NEG	NEG	NEG	NEG	NEG	POS	POS	NEG	POS	POS	POS	POS	POS
170949	CC0015	CC15-MSSA	POS	POS	POS	POS	NEG	NEG	AMB	NEG	NEG	POS	POS	NEG	NEG	NEG	NEG	NEG	NEG	NEG	NEG	NEG	NEG	NEG	POS	POS	NEG	POS	POS	NEG	POS	POS
228658	CC0022	CC22-MSSA	POS	POS	NEG	NEG	POS	POS	POS	NEG	POS	POS	POS	NEG	NEG	NEG	NEG	NEG	NEG	NEG	NEG	NEG	NEG	NEG	POS	NEG	POS	NEG	NEG	NEG	POS	POS
289125	CC0022	CC22-MRSA-IV (PVL+)	POS	POS	NEG	NEG	POS	POS	POS	NEG	POS	POS	POS	NEG	NEG	NEG	NEG	NEG	NEG	NEG	NEG	NEG	NEG	NEG	POS	NEG	POS	NEG	NEG	NEG	POS	POS
289126	CC0022	CC22-MRSA-IV (PVL+)	POS	POS	NEG	NEG	POS	POS	POS	NEG	POS	POS	POS	NEG	NEG	NEG	NEG	NEG	NEG	NEG	NEG	NEG	NEG	NEG	POS	NEG	POS	NEG	NEG	NEG	POS	POS
289127	CC0022	CC22-MRSA-IV (PVL+)	POS	POS	NEG	NEG	POS	POS	POS	NEG	POS	POS	POS	NEG	NEG	NEG	NEG	NEG	NEG	NEG	NEG	NEG	NEG	NEG	POS	NEG	POS	NEG	NEG	NEG	POS	POS
131939	CC0025	CC25-MSSA	POS	POS	POS	POS	POS	POS	AMB	NEG	POS	POS	POS	NEG	NEG	POS	NEG	POS	NEG	NEG	NEG	NEG	NEG	NEG	POS	POS	NEG	POS	POS	POS	POS	POS
275165	CC0030	CC30-MSSA (lukF-P83/lukM+)	POS	POS	POS	POS	POS	POS	POS	POS	NEG	NEG	NEG	NEG	NEG	NEG	NEG	NEG	NEG	NEG	NEG	NEG	NEG	NEG	POS	NEG	POS	NEG	NEG	POS	POS	POS
275741	CC0030	CC30-MSSA	POS	POS	POS	POS	POS	POS	NEG	POS	NEG	NEG	NEG	NEG	NEG	NEG	NEG	NEG	NEG	NEG	NEG	NEG	NEG	NEG	POS	NEG	POS	NEG	NEG	POS	POS	POS
124492	CC0049	CC49-MSSA (lukF-P83/lukM+)	POS	POS	POS	NEG	POS	POS	AMB	POS	NEG	NEG	NEG	NEG	NEG	NEG	NEG	NEG	NEG	NEG	NEG	NEG	NEG	NEG	POS	NEG	NEG	NEG	POS	POS	POS	POS
220616	CC0049	CC49-MSSA (lukF-P83/lukM+)	POS	POS	POS	NEG	POS	POS	POS	POS	NEG	NEG	NEG	NEG	NEG	NEG	NEG	NEG	NEG	NEG	NEG	NEG	NEG	NEG	POS	POS	NEG	POS	POS	POS	POS	POS
228649	CC0049	CC49-MSSA (lukF-P83/lukM+)	POS	POS	POS	NEG	POS	POS	POS	POS	NEG	NEG	NEG	NEG	NEG	NEG	NEG	NEG	NEG	NEG	NEG	NEG	NEG	NEG	POS	NEG	NEG	POS	POS	POS	POS	POS
190994	CC0059	CC59-MSSA	POS	AMB	POS	NEG	NEG	NEG	NEG	POS	NEG	POS	AMB	NEG	NEG	NEG	NEG	NEG	NEG	NEG	NEG	NEG	NEG	NEG	NEG	NEG	NEG	NEG	NEG	NEG	POS	POS
275172	CC0088	CC88-MSSA	POS	POS	POS	NEG	POS	POS	POS	NEG	POS	POS	POS	NEG	NEG	NEG	NEG	NEG	NEG	NEG	NEG	NEG	NEG	NEG	NEG	POS	NEG	POS	POS	NEG	POS	POS
275173	CC0088	CC88-MSSA	POS	POS	POS	POS	POS	POS	POS	NEG	POS	POS	POS	NEG	NEG	NEG	NEG	NEG	NEG	NEG	NEG	NEG	NEG	NEG	POS	POS	NEG	POS	POS	NEG	POS	POS
275174	CC0088	CC88-MSSA	POS	POS	POS	POS	POS	POS	POS	NEG	POS	POS	POS	NEG	NEG	NEG	NEG	NEG	NEG	NEG	NEG	NEG	NEG	NEG	POS	POS	NEG	POS	POS	NEG	POS	POS
170971	CC0097	CC97-MSSA	POS	POS	POS	POS	POS	POS	POS	NEG	NEG	NEG	NEG	NEG	NEG	NEG	NEG	NEG	NEG	NEG	NEG	NEG	NEG	NEG	POS	POS	NEG	POS	POS	POS	POS	POS
112745	CC0097	CC97-MSSA	POS	POS	POS	POS	POS	POS	POS	NEG	NEG	NEG	NEG	NEG	NEG	NEG	NEG	NEG	NEG	NEG	NEG	NEG	NEG	NEG	POS	POS	NEG	POS	POS	POS	POS	POS
112748	CC0097	CC97-MSSA	POS	POS	POS	POS	POS	POS	POS	NEG	NEG	NEG	NEG	NEG	NEG	NEG	NEG	NEG	NEG	NEG	NEG	NEG	NEG	NEG	POS	POS	NEG	POS	POS	POS	POS	POS
112750	CC0097	CC97-MSSA	POS	POS	POS	POS	POS	POS	POS	NEG	NEG	NEG	NEG	NEG	NEG	NEG	NEG	NEG	NEG	NEG	NEG	NEG	NEG	NEG	POS	POS	NEG	POS	POS	POS	POS	POS
112762	CC0097	CC97-MSSA	POS	POS	POS	POS	POS	POS	POS	NEG	NEG	NEG	NEG	NEG	NEG	NEG	NEG	NEG	NEG	NEG	NEG	NEG	NEG	NEG	POS	POS	NEG	POS	POS	POS	POS	POS
112763	CC0097	CC97-MSSA	POS	POS	POS	POS	POS	POS	POS	AMB	NEG	NEG	NEG	NEG	NEG	NEG	NEG	NEG	NEG	NEG	NEG	NEG	NEG	NEG	POS	POS	NEG	POS	POS	POS	POS	POS
112764	CC0097	CC97-MSSA	POS	POS	POS	POS	POS	POS	POS	NEG	NEG	NEG	NEG	NEG	NEG	NEG	NEG	NEG	NEG	NEG	NEG	NEG	NEG	NEG	POS	POS	NEG	POS	POS	NEG	POS	POS
170945	CC0097	CC97-MSSA	POS	POS	POS	POS	POS	POS	POS	NEG	NEG	NEG	NEG	NEG	NEG	NEG	NEG	NEG	NEG	NEG	NEG	NEG	NEG	NEG	POS	POS	NEG	POS	POS	POS	POS	POS
170954	CC0097	CC97-MSSA	POS	POS	POS	POS	POS	POS	POS	NEG	NEG	NEG	NEG	NEG	NEG	NEG	NEG	NEG	NEG	NEG	NEG	NEG	NEG	NEG	POS	POS	NEG	POS	POS	POS	POS	POS
170958	CC0097	CC97-MSSA	POS	POS	POS	POS	POS	POS	POS	NEG	NEG	NEG	NEG	NEG	NEG	NEG	NEG	NEG	NEG	NEG	NEG	NEG	NEG	NEG	POS	POS	NEG	POS	POS	POS	POS	POS
170963	CC0097	CC97-MSSA	POS	POS	POS	POS	POS	POS	POS	NEG	NEG	NEG	NEG	NEG	NEG	NEG	NEG	NEG	NEG	NEG	NEG	NEG	NEG	NEG	POS	POS	NEG	POS	POS	POS	POS	POS
170964	CC0097	CC97-MSSA	POS	POS	POS	POS	POS	POS	POS	NEG	NEG	NEG	NEG	NEG	NEG	NEG	NEG	NEG	NEG	NEG	NEG	NEG	NEG	NEG	POS	POS	NEG	POS	POS	POS	POS	POS
170965	CC0097	CC97-MSSA	POS	POS	POS	POS	POS	POS	POS	NEG	NEG	NEG	NEG	NEG	NEG	NEG	NEG	NEG	NEG	NEG	NEG	NEG	NEG	NEG	POS	POS	NEG	POS	POS	POS	POS	POS
170966	CC0097	CC97-MSSA	POS	POS	POS	POS	POS	POS	POS	NEG	NEG	NEG	NEG	NEG	NEG	NEG	NEG	NEG	NEG	NEG	NEG	NEG	NEG	NEG	POS	POS	NEG	POS	POS	POS	POS	POS
170974	CC0097	CC97-MSSA	POS	POS	POS	POS	POS	POS	POS	NEG	NEG	NEG	NEG	NEG	NEG	NEG	NEG	NEG	NEG	NEG	NEG	NEG	NEG	NEG	AMB	POS	NEG	POS	POS	POS	POS	POS
170976	CC0097	CC97-MSSA	POS	POS	POS	POS	POS	POS	POS	NEG	NEG	NEG	NEG	NEG	NEG	NEG	NEG	NEG	NEG	NEG	NEG	NEG	NEG	NEG	POS	POS	NEG	POS	POS	POS	POS	POS
112744	CC0097	CC97-MSSA	POS	POS	POS	POS	POS	POS	POS	NEG	NEG	NEG	NEG	NEG	NEG	NEG	NEG	NEG	NEG	NEG	NEG	NEG	NEG	NEG	POS	POS	NEG	POS	POS	POS	POS	POS
112749	CC0097	CC97-MSSA	POS	POS	POS	POS	POS	POS	POS	NEG	NEG	NEG	NEG	NEG	NEG	NEG	NEG	NEG	NEG	NEG	NEG	NEG	NEG	NEG	POS	POS	NEG	POS	POS	POS	POS	POS
112757	CC0097	CC97-MSSA	POS	POS	POS	POS	POS	POS	POS	AMB	NEG	NEG	NEG	NEG	NEG	NEG	NEG	NEG	NEG	NEG	NEG	NEG	NEG	NEG	POS	POS	NEG	POS	POS	POS	POS	POS
170973	CC0097	CC97-MSSA	POS	POS	POS	POS	POS	POS	POS	NEG	NEG	NEG	NEG	NEG	NEG	NEG	NEG	NEG	NEG	NEG	NEG	NEG	NEG	NEG	POS	POS	NEG	POS	POS	POS	POS	POS
207756	CC0097	CC97-MSSA	POS	POS	POS	POS	POS	POS	POS	AMB	NEG	NEG	NEG	NEG	NEG	NEG	NEG	NEG	NEG	NEG	NEG	NEG	NEG	NEG	POS	POS	NEG	POS	POS	POS	POS	POS
206171	CC0097	CC97-MSSA	POS	POS	POS	POS	POS	POS	POS	POS	NEG	NEG	NEG	NEG	NEG	NEG	NEG	NEG	NEG	NEG	NEG	NEG	NEG	NEG	POS	POS	NEG	POS	POS	POS	POS	POS
275169	CC0097	CC97-MSSA-[ccrAA]	POS	POS	POS	POS	POS	POS	POS	POS	NEG	NEG	NEG	NEG	NEG	NEG	NEG	NEG	NEG	NEG	NEG	NEG	NEG	NEG	POS	POS	NEG	POS	POS	POS	POS	POS
275168	CC0097	CC97-MSSA-[ccrAA]	POS	POS	POS	POS	POS	POS	POS	POS	NEG	NEG	NEG	NEG	NEG	NEG	NEG	NEG	NEG	NEG	NEG	NEG	NEG	NEG	POS	POS	NEG	POS	POS	POS	POS	POS
118320	CC0130	CC130-MSSA (lukF-P83/M+)	POS	POS	POS	NEG	POS	POS	POS	POS	NEG	NEG	NEG	NEG	NEG	NEG	NEG	POS	NEG	NEG	NEG	NEG	NEG	NEG	POS	POS	NEG	POS	POS	POS	POS	POS
203236	CC0130	CC130-MRSA-XI	POS	POS	POS	NEG	POS	POS	POS	POS	NEG	NEG	NEG	NEG	NEG	NEG	NEG	POS	NEG	NEG	NEG	NEG	NEG	NEG	POS	POS	NEG	POS	POS	NEG	POS	POS
204204	CC0130	CC130-MRSA-XI	POS	POS	POS	NEG	POS	POS	POS	POS	NEG	NEG	NEG	NEG	NEG	NEG	NEG	POS	NEG	NEG	NEG	NEG	NEG	NEG	POS	POS	NEG	POS	POS	NEG	POS	POS
289122	CC0130	CC130-MRSA-XI	POS	POS	POS	NEG	POS	POS	POS	POS	NEG	NEG	NEG	NEG	NEG	NEG	NEG	POS	NEG	NEG	NEG	NEG	NEG	NEG	POS	POS	NEG	POS	POS	NEG	POS	POS
225779	CC0130	CC130-MRSA-XI	POS	POS	POS	NEG	POS	POS	POS	POS	NEG	NEG	NEG	NEG	NEG	NEG	NEG	POS	NEG	NEG	NEG	NEG	NEG	NEG	POS	POS	NEG	POS	POS	NEG	POS	POS
225780	CC0130	CC130-MRSA-XI	POS	POS	POS	NEG	POS	POS	POS	POS	NEG	NEG	NEG	NEG	NEG	NEG	NEG	POS	NEG	NEG	NEG	NEG	NEG	NEG	POS	POS	NEG	POS	POS	NEG	POS	POS
170959	CC0130	CC130-MRSA-XI	POS	POS	POS	NEG	POS	POS	POS	POS	NEG	NEG	NEG	NEG	NEG	NEG	NEG	POS	NEG	NEG	NEG	NEG	NEG	NEG	POS	POS	NEG	POS	POS	POS	POS	POS
179029	CC0130	CC130-MRSA-XI	POS	POS	POS	NEG	POS	POS	POS	POS	NEG	NEG	NEG	NEG	NEG	NEG	NEG	POS	NEG	NEG	NEG	NEG	NEG	NEG	POS	POS	NEG	POS	POS	POS	POS	POS
116254	CC0133	CC133-MSSA	POS	POS	POS	NEG	POS	POS	AMB	POS	NEG	NEG	NEG	NEG	NEG	NEG	NEG	NEG	NEG	NEG	NEG	NEG	NEG	NEG	POS	POS	NEG	POS	POS	POS	POS	POS
207761	CC0133	CC133-MSSA	POS	POS	POS	NEG	POS	POS	POS	POS	NEG	NEG	NEG	NEG	NEG	NEG	NEG	NEG	NEG	NEG	NEG	NEG	NEG	NEG	POS	POS	NEG	POS	POS	POS	POS	POS
213103	CC0133	CC133-MSSA	POS	POS	POS	NEG	POS	POS	POS	POS	NEG	NEG	NEG	NEG	NEG	NEG	NEG	NEG	NEG	NEG	NEG	NEG	NEG	NEG	POS	POS	NEG	POS	POS	POS	POS	POS
275736	CC0133	CC133-MSSA	POS	POS	POS	NEG	POS	POS	POS	NEG	NEG	NEG	NEG	NEG	NEG	NEG	NEG	NEG	NEG	NEG	NEG	NEG	NEG	NEG	POS	POS	NEG	POS	POS	NEG	POS	POS
275737	CC0133	CC133-MSSA	POS	POS	POS	NEG	POS	POS	POS	NEG	NEG	NEG	NEG	NEG	NEG	NEG	NEG	NEG	NEG	NEG	NEG	NEG	NEG	NEG	POS	POS	NEG	POS	POS	NEG	POS	POS
275738	CC0133	CC133-MSSA	POS	POS	POS	NEG	POS	POS	POS	NEG	NEG	NEG	NEG	NEG	NEG	NEG	NEG	NEG	NEG	NEG	NEG	NEG	NEG	NEG	POS	POS	NEG	POS	POS	NEG	POS	POS
275739	CC0133	CC133-MSSA	POS	POS	POS	NEG	POS	POS	POS	NEG	NEG	NEG	NEG	NEG	NEG	NEG	NEG	NEG	NEG	NEG	NEG	NEG	NEG	NEG	POS	POS	NEG	POS	POS	NEG	POS	POS
112755	CC0133	CC133-MSSA	POS	POS	POS	NEG	POS	POS	POS	NEG	NEG	NEG	NEG	NEG	NEG	NEG	NEG	NEG	NEG	NEG	NEG	NEG	NEG	NEG	POS	POS	NEG	POS	POS	NEG	POS	POS
225778	CC0398	CC398-MRSA-V/VT	POS	POS	POS	POS	NEG	NEG	NEG	POS	NEG	NEG	NEG	NEG	NEG	NEG	NEG	NEG	NEG	NEG	NEG	NEG	NEG	NEG	POS	NEG	POS	NEG	NEG	NEG	POS	POS
123159	ST0425	ST425-MSSA	POS	AMB	POS	NEG	POS	POS	POS	POS	NEG	NEG	NEG	NEG	NEG	NEG	NEG	NEG	NEG	NEG	NEG	NEG	NEG	NEG	POS	POS	NEG	AMB	POS	POS	POS	POS
86991	ST0425	ST425-MSSA	POS	POS	POS	NEG	POS	POS	POS	NEG	NEG	NEG	NEG	NEG	NEG	NEG	NEG	NEG	NEG	NEG	NEG	NEG	NEG	NEG	POS	POS	NEG	POS	POS	POS	POS	POS
112756	ST0425	ST425-MSSA	POS	POS	POS	NEG	POS	POS	POS	NEG	NEG	NEG	NEG	NEG	NEG	NEG	NEG	NEG	NEG	NEG	NEG	NEG	NEG	NEG	POS	POS	NEG	POS	POS	POS	POS	POS
195207	ST0425	ST425-MSSA	POS	POS	POS	NEG	POS	POS	POS	POS	NEG	NEG	NEG	NEG	NEG	NEG	NEG	NEG	NEG	NEG	NEG	NEG	NEG	NEG	POS	POS	NEG	POS	POS	POS	POS	POS
223609	ST0425	ST425-MSSA	POS	POS	POS	NEG	POS	POS	POS	POS	NEG	NEG	NEG	NEG	NEG	NEG	NEG	NEG	NEG	NEG	NEG	NEG	NEG	NEG	POS	POS	NEG	POS	POS	POS	POS	POS
275740	ST0425	ST425-MSSA	POS	POS	POS	NEG	POS	POS	POS	POS	NEG	NEG	NEG	NEG	NEG	NEG	NEG	NEG	NEG	NEG	NEG	NEG	NEG	NEG	POS	POS	NEG	POS	POS	POS	POS	POS
174889	ST0425	ST425-MSSA	POS	POS	POS	NEG	POS	POS	POS	POS	NEG	NEG	NEG	NEG	NEG	NEG	NEG	NEG	NEG	NEG	NEG	NEG	NEG	NEG	POS	POS	NEG	POS	POS	NEG	POS	POS
203230	ST0425	ST425-MSSA	POS	POS	POS	NEG	POS	POS	POS	POS	NEG	NEG	NEG	NEG	NEG	NEG	NEG	NEG	NEG	NEG	NEG	NEG	NEG	NEG	POS	POS	NEG	POS	POS	NEG	POS	POS
203242	ST0425	ST425-MSSA	POS	POS	POS	NEG	POS	POS	POS	POS	NEG	NEG	NEG	NEG	NEG	NEG	NEG	NEG	NEG	NEG	NEG	NEG	NEG	NEG	POS	POS	NEG	POS	POS	POS	POS	POS
203243	ST0425	ST425-MSSA	POS	POS	POS	NEG	POS	POS	POS	POS	NEG	NEG	NEG	NEG	NEG	NEG	NEG	NEG	NEG	NEG	NEG	NEG	NEG	NEG	POS	POS	NEG	POS	POS	POS	POS	POS
207951	ST0425	ST425-MSSA	POS	AMB	POS	NEG	AMB	POS	AMB	POS	NEG	NEG	NEG	NEG	NEG	NEG	NEG	NEG	NEG	NEG	NEG	NEG	NEG	NEG	POS	POS	NEG	POS	POS	POS	POS	POS
207981	ST0425	ST425-MSSA	POS	AMB	POS	NEG	POS	POS	POS	NEG	NEG	NEG	NEG	NEG	NEG	NEG	NEG	NEG	NEG	NEG	NEG	NEG	NEG	NEG	POS	POS	NEG	POS	POS	POS	POS	POS
195212	ST0425	ST425-MSSA	POS	POS	POS	NEG	POS	POS	POS	POS	NEG	NEG	NEG	NEG	NEG	NEG	NEG	NEG	NEG	NEG	NEG	NEG	NEG	NEG	POS	POS	NEG	POS	POS	POS	POS	POS
195206	ST0425	ST425-MSSA	POS	POS	POS	NEG	POS	POS	POS	POS	NEG	NEG	NEG	NEG	NEG	NEG	NEG	NEG	NEG	NEG	NEG	NEG	NEG	NEG	POS	POS	NEG	POS	POS	POS	POS	POS
195202	ST0425	ST425-MSSA	POS	POS	POS	NEG	POS	POS	POS	POS	NEG	NEG	NEG	NEG	NEG	NEG	NEG	NEG	NEG	NEG	NEG	NEG	NEG	NEG	POS	POS	NEG	POS	POS	POS	POS	POS
216042	ST0425	ST425-MSSA	POS	POS	POS	NEG	POS	POS	POS	POS	NEG	NEG	NEG	NEG	NEG	NEG	NEG	NEG	NEG	NEG	NEG	NEG	NEG	NEG	POS	POS	NEG	POS	POS	POS	POS	POS
223608	ST0425	ST425-MSSA	POS	POS	POS	NEG	POS	POS	POS	POS	NEG	NEG	NEG	NEG	NEG	NEG	NEG	NEG	NEG	NEG	NEG	NEG	NEG	NEG	POS	POS	NEG	POS	POS	POS	POS	POS
223612	ST0425	ST425-MSSA	POS	POS	POS	NEG	POS	POS	POS	POS	NEG	NEG	NEG	NEG	NEG	NEG	NEG	NEG	NEG	NEG	NEG	NEG	NEG	NEG	POS	POS	NEG	POS	POS	POS	POS	POS
225733	ST0425	ST425-MSSA	POS	POS	POS	NEG	POS	POS	POS	POS	NEG	NEG	NEG	NEG	NEG	NEG	NEG	NEG	NEG	NEG	NEG	NEG	NEG	NEG	POS	POS	NEG	POS	POS	POS	POS	POS
230321	ST0425	ST425-MSSA	POS	POS	POS	NEG	POS	POS	POS	POS	NEG	NEG	NEG	NEG	NEG	NEG	NEG	NEG	NEG	NEG	NEG	NEG	NEG	NEG	POS	POS	NEG	POS	POS	POS	POS	POS
232368	ST0425	ST425-MSSA	POS	POS	POS	NEG	POS	POS	POS	POS	NEG	NEG	NEG	NEG	NEG	NEG	NEG	NEG	NEG	NEG	NEG	NEG	NEG	NEG	POS	POS	NEG	POS	POS	POS	POS	POS
204215	ST0425	ST425-MSSA	POS	POS	POS	NEG	POS	POS	POS	POS	NEG	NEG	NEG	NEG	NEG	NEG	NEG	NEG	NEG	NEG	NEG	NEG	NEG	NEG	POS	POS	NEG	POS	POS	POS	POS	POS
204218	ST0425	ST425-MSSA	POS	POS	POS	NEG	POS	POS	POS	POS	NEG	NEG	NEG	NEG	NEG	NEG	NEG	NEG	NEG	NEG	NEG	NEG	NEG	NEG	POS	POS	NEG	POS	POS	POS	POS	POS
204222	ST0425	ST425-MSSA	POS	POS	POS	NEG	POS	POS	POS	POS	NEG	NEG	NEG	NEG	NEG	NEG	NEG	NEG	NEG	NEG	NEG	NEG	NEG	NEG	POS	POS	NEG	POS	POS	POS	POS	POS
204226	ST0425	ST425-MSSA	POS	POS	POS	NEG	POS	POS	POS	POS	NEG	NEG	NEG	NEG	NEG	NEG	NEG	NEG	NEG	NEG	NEG	NEG	NEG	NEG	POS	POS	NEG	POS	POS	POS	POS	POS
204906	ST0425	ST425-MSSA	POS	POS	POS	NEG	POS	POS	POS	POS	NEG	NEG	NEG	NEG	NEG	NEG	NEG	NEG	NEG	NEG	NEG	NEG	NEG	NEG	POS	POS	NEG	POS	POS	POS	POS	POS
204908	ST0425	ST425-MSSA	POS	POS	POS	NEG	POS	POS	POS	POS	NEG	NEG	NEG	NEG	NEG	AMB	NEG	NEG	NEG	NEG	NEG	NEG	NEG	NEG	POS	POS	NEG	POS	POS	POS	POS	POS
206170	ST0425	ST425-MSSA	POS	POS	POS	NEG	POS	POS	POS	POS	NEG	NEG	NEG	NEG	NEG	NEG	NEG	NEG	NEG	NEG	NEG	NEG	NEG	NEG	POS	POS	NEG	POS	POS	POS	POS	POS
207765	ST0425	ST425-MSSA	POS	POS	POS	NEG	POS	POS	POS	POS	NEG	NEG	NEG	NEG	NEG	NEG	NEG	NEG	NEG	NEG	NEG	NEG	NEG	NEG	POS	POS	NEG	POS	POS	POS	POS	POS
240048	CC599	CC599-MRSA-XI	POS	POS	POS	NEG	AMB	POS	AMB	POS	NEG	NEG	NEG	NEG	NEG	NEG	NEG	NEG	NEG	NEG	NEG	NEG	NEG	NEG	POS	POS	NEG	POS	POS	POS	POS	POS
225730	CC0692	CC692-MSSA	POS	POS	POS	POS	POS	POS	POS	NEG	NEG	NEG	NEG	NEG	NEG	NEG	NEG	NEG	NEG	NEG	NEG	NEG	NEG	NEG	POS	POS	NEG	AMB	POS	NEG	POS	POS
112746	CC0692	CC692-MSSA	POS	POS	POS	POS	POS	POS	POS	NEG	NEG	NEG	NEG	NEG	NEG	NEG	NEG	NEG	NEG	NEG	NEG	NEG	NEG	NEG	POS	POS	NEG	NEG	POS	NEG	POS	POS
112754	CC0692	CC692-MSSA	POS	POS	POS	POS	POS	POS	POS	AMB	NEG	NEG	NEG	NEG	NEG	NEG	NEG	NEG	NEG	NEG	NEG	NEG	NEG	NEG	POS	POS	NEG	AMB	POS	NEG	POS	POS
112758	CC0692	CC692-MSSA	POS	POS	POS	POS	POS	POS	POS	NEG	NEG	NEG	NEG	NEG	NEG	NEG	NEG	NEG	NEG	NEG	NEG	NEG	NEG	NEG	POS	POS	NEG	AMB	POS	NEG	POS	POS
112759	CC0692	CC692-MSSA	POS	POS	POS	POS	POS	POS	POS	NEG	NEG	NEG	NEG	NEG	NEG	NEG	NEG	NEG	NEG	NEG	NEG	NEG	NEG	NEG	POS	POS	NEG	NEG	POS	NEG	POS	POS
124745	CC0692	CC692-MSSA	POS	POS	POS	POS	POS	POS	POS	NEG	NEG	NEG	NEG	NEG	NEG	NEG	NEG	NEG	NEG	NEG	NEG	NEG	NEG	NEG	POS	POS	NEG	AMB	POS	NEG	POS	POS
170952	CC0692	CC692-MSSA	POS	POS	POS	POS	POS	POS	POS	NEG	NEG	NEG	NEG	NEG	NEG	NEG	NEG	NEG	NEG	NEG	NEG	NEG	NEG	NEG	POS	POS	NEG	AMB	POS	NEG	POS	POS
170953	CC0692	CC692-MSSA	POS	POS	POS	POS	POS	POS	POS	NEG	NEG	NEG	NEG	NEG	NEG	NEG	NEG	NEG	NEG	NEG	NEG	NEG	NEG	NEG	POS	POS	NEG	NEG	POS	NEG	POS	POS
170970	CC0692	CC692-MSSA	POS	POS	POS	POS	POS	POS	POS	NEG	NEG	NEG	NEG	NEG	NEG	NEG	NEG	NEG	NEG	NEG	NEG	NEG	NEG	NEG	POS	POS	NEG	NEG	POS	NEG	POS	POS
170950	CC0707	CC707-MSSA	POS	POS	POS	NEG	POS	POS	POS	NEG	NEG	NEG	NEG	NEG	NEG	NEG	NEG	NEG	NEG	NEG	NEG	NEG	NEG	NEG	POS	POS	NEG	NEG	POS	NEG	POS	POS
124948	ST0890	ST890-MSSA	POS	POS	POS	NEG	NEG	NEG	NEG	NEG	NEG	NEG	NEG	NEG	NEG	NEG	NEG	POS	NEG	NEG	NEG	NEG	NEG	NEG	POS	POS	NEG	NEG	NEG	NEG	POS	POS
220618	CC1956	CC1956-MSSA	POS	POS	POS	NEG	POS	POS	POS	POS	NEG	NEG	NEG	NEG	NEG	NEG	NEG	NEG	NEG	NEG	NEG	NEG	NEG	NEG	POS	POS	NEG	POS	POS	POS	POS	POS
112742	ST2425	ST2425-MSSA	POS	POS	POS	NEG	POS	POS	POS	NEG	NEG	NEG	NEG	NEG	NEG	NEG	NEG	NEG	NEG	NEG	NEG	NEG	NEG	NEG	POS	POS	NEG	POS	POS	POS	POS	POS
170957	ST2425	ST2425-MSSA	POS	POS	POS	NEG	POS	POS	POS	NEG	NEG	NEG	NEG	NEG	NEG	NEG	NEG	NEG	NEG	NEG	NEG	NEG	NEG	NEG	POS	POS	NEG	POS	POS	AMB	POS	POS
170947	ST2691	ST2691-MSSA	POS	POS	POS	NEG	POS	POS	POS	NEG	NEG	NEG	NEG	NEG	NEG	NEG	NEG	NEG	NEG	NEG	NEG	NEG	NEG	NEG	POS	POS	NEG	NEG	NEG	POS	POS	POS
170961	ST2691	ST2691-MSSA	POS	POS	POS	NEG	POS	POS	POS	AMB	NEG	NEG	NEG	NEG	NEG	NEG	NEG	NEG	NEG	NEG	NEG	NEG	NEG	NEG	POS	POS	NEG	AMB	NEG	POS	POS	POS
112753	CC2767	ST2279-MSSA	POS	POS	POS	NEG	POS	POS	POS	AMB	NEG	NEG	NEG	NEG	NEG	NEG	NEG	NEG	NEG	NEG	NEG	NEG	NEG	NEG	POS	POS	NEG	NEG	NEG	NEG	POS	POS
112761	CC2767	ST2279-MSSA	POS	POS	POS	NEG	POS	POS	AMB	NEG	NEG	NEG	NEG	NEG	NEG	NEG	NEG	NEG	NEG	NEG	NEG	NEG	NEG	NEG	POS	POS	NEG	POS	POS	NEG	POS	POS
228651	ST2963	ST2963-MSSA	POS	POS	POS	NEG	POS	POS	POS	NEG	NEG	NEG	NEG	NEG	NEG	NEG	NEG	NEG	NEG	NEG	NEG	NEG	NEG	NEG	POS	POS	NEG	POS	POS	NEG	POS	POS
203244	ST3237	ST3237-MSSA	POS	POS	POS	NEG	NEG	NEG	POS	POS	NEG	NEG	NEG	NEG	NEG	NEG	NEG	POS	NEG	NEG	NEG	NEG	NEG	NEG	POS	POS	NEG	POS	NEG	POS	POS	POS

ISOLATE ID	CLONAL COMPLEX (by array)	STRAIN AFFILIATION (by array)	RULENCE : PROTEASES		VIRULENCE : STAPHYLOCOCCAL SUPERANTIGEN/ENTEROTOXIN-LIKE GENES (SET/SSL)																							
			staphopain A (staphylopain A), protease		staphyl. exotoxin-like protein	staphylococcal superantigen-like protein 1												staphylococcal superantigen-like protein 2		staphylococcal superantigen-like protein 3			staphylococcal superantigen-like protein 4		staphylococcal superantigen-like protein 5			
			sspP (cons)	sspP (other than ST93)	setC	set6-var1_11	set6-var2_11	set6-var1_12	set6-var2_12	set6-var4_11	ssl01-RF122	ssl01/set6 (COL)	ssl01/set6 (Mu50+N315)	ssl01/set6 (MW2+MSSA476)	ssl01/set6 (MRSA252)	ssl01/set6 (RF122)	ssl01/set6 (other alleles)	ssl02/set7	ssl02/set7 (MRSA252)	ssl03/set8_probe 1	ssl03/set8_probe 2	ssl03/set8 (MRSA252, SAR0424)	ssl04/set9	ssl04/set9 (MRSA252, SAR0425)	ssl05/set3_probe 1	ssl05/set3 (RF122, probe-611)	ssl05/set3_probe 2 (612)	ssl05/set3 (MRSA252)
203228	CC0001	CC1-MSSA	POS	POS	POS	NEG	POS	AMB	POS	NEG	NEG	NEG	NEG	POS	NEG	NEG	NEG	AMB	POS	POS	POS	NEG	POS	AMB	POS	AMB	POS	NEG
203229	CC0001	CC1-MSSA	POS	POS	POS	NEG	POS	AMB	POS	AMB	NEG	NEG	NEG	POS	NEG	NEG	NEG	POS	AMB	POS	POS	NEG	POS	AMB	POS	POS	AMB	NEG
203233	CC0001	CC1-MSSA	POS	POS	POS	NEG	POS	NEG	POS	NEG	NEG	NEG	NEG	POS	NEG	NEG	NEG	POS	AMB	POS	POS	NEG	POS	NEG	POS	POS	AMB	NEG
220334	CC0001	CC1-MSSA	POS	POS	POS	NEG	POS	NEG	POS	NEG	NEG	NEG	NEG	POS	NEG	NEG	NEG	POS	AMB	POS	POS	NEG	POS	NEG	POS	AMB	POS	NEG
223621	CC0001	CC1-MSSA	POS	POS	POS	NEG	POS	NEG	POS	NEG	NEG	NEG	NEG	POS	NEG	NEG	NEG	POS	AMB	POS	POS	NEG	POS	AMB	POS	AMB	POS	NEG
289123	CC0001	CC1-MRSA-IV, WA MRSA-1/57	POS	POS	POS	NEG	POS	NEG	POS	NEG	NEG	NEG	NEG	POS	NEG	NEG	NEG	POS	AMB	POS	POS	NEG	POS	NEG	POS	AMB	POS	NEG
289124	CC0001	CC1-MRSA-IV, WA MRSA-1/57	POS	POS	POS	NEG	POS	NEG	POS	NEG	NEG	NEG	NEG	POS	NEG	NEG	NEG	POS	NEG	POS	POS	NEG	POS	NEG	POS	AMB	POS	NEG
275170	CC0005	CC5-MSSA	POS	POS	POS	POS	NEG	POS	NEG	POS	NEG	NEG	POS	NEG	NEG	NEG	NEG	POS	NEG	POS	POS	NEG	POS	NEG	POS	NEG	POS	NEG
275171	CC0005	CC5-MSSA	POS	POS	POS	POS	NEG	POS	NEG	POS	NEG	NEG	POS	NEG	NEG	NEG	NEG	POS	NEG	POS	POS	NEG	POS	NEG	POS	NEG	POS	NEG
112747	CC0005	CC5-MSSA	POS	POS	POS	POS	NEG	POS	NEG	POS	NEG	NEG	POS	NEG	NEG	0	NEG	POS	AMB	POS	POS	NEG	POS	NEG	POS	AMB	POS	NEG
112752	CC0006	CC6-MSSA	POS	POS	POS	NEG	NEG	POS	NEG	POS	POS	NEG	NEG	NEG	NEG	0	POS	POS	AMB	POS	POS	NEG	POS	NEG	POS	AMB	POS	POS
275178	CC0007	CC7-MSSA	POS	POS	POS	POS	NEG	NEG	POS	NEG	NEG	NEG	NEG	NEG	NEG	NEG	POS	POS	NEG	POS	POS	NEG	POS	NEG	POS	NEG	POS	NEG
275166	CC0008	CC8-MSSA	POS	POS	POS	POS	NEG	NEG	NEG	POS	NEG	POS	AMB	NEG	NEG	NEG	NEG	POS	NEG	POS	POS	NEG	POS	NEG	POS	NEG	POS	NEG
275176	CC0008	CC8-MSSA	POS	POS	POS	POS	NEG	NEG	NEG	POS	NEG	POS	AMB	NEG	NEG	NEG	NEG	POS	NEG	POS	POS	NEG	POS	NEG	POS	NEG	POS	NEG
275177	CC0008	CC8-MSSA	POS	POS	POS	POS	NEG	NEG	NEG	POS	NEG	POS	AMB	NEG	NEG	NEG	NEG	POS	NEG	POS	POS	NEG	POS	NEG	POS	NEG	POS	NEG
275167	CC0009	CC9-MSSA	POS	POS	POS	NEG	NEG	NEG	POS	NEG	POS	NEG	NEG	NEG	NEG	POS	NEG	POS	NEG	AMB	POS	NEG	POS	NEG	NEG	NEG	POS	NEG
170968	CC0012	CC12-MSSA	POS	POS	POS	NEG	NEG	NEG	NEG	POS	POS	NEG	NEG	NEG	NEG	NEG	POS	POS	NEG	POS	POS	NEG	POS	NEG	POS	0	0	NEG
275175	CC0015	CC15-MSSA	POS	POS	POS	NEG	NEG	NEG	NEG	POS	NEG	NEG	NEG	NEG	NEG	NEG	POS	POS	NEG	POS	POS	NEG	POS	NEG	POS	POS	AMB	NEG
170949	CC0015	CC15-MSSA	POS	POS	POS	NEG	NEG	NEG	NEG	POS	NEG	NEG	NEG	NEG	NEG	NEG	POS	POS	NEG	POS	POS	NEG	POS	NEG	POS	NEG	AMB	NEG
228658	CC0022	CC22-MSSA	POS	POS	POS	NEG	POS	POS	NEG	NEG	NEG	NEG	NEG	NEG	POS	NEG	NEG	AMB	POS	NEG	NEG	AMB	NEG	POS	POS	NEG	NEG	POS
289125	CC0022	CC22-MRSA-IV (PVL+)	POS	POS	POS	NEG	POS	POS	NEG	NEG	NEG	NEG	NEG	NEG	POS	NEG	NEG	POS	AMB	NEG	NEG	POS	NEG	POS	POS	NEG	NEG	POS
289126	CC0022	CC22-MRSA-IV (PVL+)	POS	POS	POS	NEG	POS	POS	NEG	NEG	NEG	NEG	NEG	NEG	POS	NEG	NEG	AMB	POS	NEG	NEG	NEG	NEG	POS	POS	NEG	NEG	POS
289127	CC0022	CC22-MRSA-IV (PVL+)	POS	POS	POS	NEG	POS	POS	NEG	NEG	NEG	NEG	NEG	NEG	POS	NEG	NEG	AMB	POS	NEG	NEG	NEG	NEG	POS	POS	NEG	NEG	POS
131939	CC0025	CC25-MSSA	POS	POS	POS	NEG	AMB	NEG	NEG	POS	NEG	NEG	NEG	NEG	NEG	NEG	POS	POS	NEG	POS	POS	NEG	POS	NEG	POS	POS	AMB	NEG
275165	CC0030	CC30-MSSA (lukF-P83/lukM+)	POS	POS	NEG	NEG	POS	POS	NEG	POS	NEG	NEG	NEG	NEG	POS	NEG	NEG	NEG	POS	NEG	NEG	POS	NEG	POS	NEG	NEG	NEG	POS
275741	CC0030	CC30-MSSA	POS	POS	NEG	NEG	POS	POS	NEG	POS	NEG	NEG	NEG	NEG	POS	NEG	NEG	NEG	POS	NEG	NEG	POS	NEG	POS	NEG	NEG	NEG	POS
124492	CC0049	CC49-MSSA (lukF-P83/lukM+)	POS	POS	POS	POS	NEG	NEG	POS	NEG	NEG	NEG	NEG	NEG	NEG	NEG	POS	NEG	NEG	POS	POS	NEG	POS	NEG	NEG	POS	AMB	NEG
220616	CC0049	CC49-MSSA (lukF-P83/lukM+)	POS	POS	POS	POS	NEG	NEG	POS	NEG	NEG	NEG	NEG	NEG	NEG	NEG	POS	POS	NEG	POS	POS	NEG	POS	NEG	NEG	POS	AMB	NEG
228649	CC0049	CC49-MSSA (lukF-P83/lukM+)	POS	POS	POS	POS	NEG	NEG	POS	NEG	NEG	NEG	NEG	NEG	NEG	NEG	POS	POS	NEG	POS	POS	NEG	POS	NEG	NEG	POS	AMB	NEG
190994	CC0059	CC59-MSSA	POS	POS	NEG	POS	NEG	POS	NEG	NEG	NEG	NEG	POS	NEG	NEG	NEG	NEG	NEG	NEG	POS	POS	NEG	POS	NEG	POS	POS	NEG	NEG
275172	CC0088	CC88-MSSA	POS	POS	POS	NEG	NEG	NEG	NEG	POS	NEG	NEG	NEG	NEG	NEG	NEG	POS	POS	NEG	POS	POS	NEG	POS	NEG	NEG	NEG	POS	NEG
275173	CC0088	CC88-MSSA	POS	POS	POS	NEG	NEG	NEG	NEG	POS	NEG	NEG	NEG	NEG	NEG	NEG	POS	POS	NEG	POS	POS	NEG	POS	NEG	NEG	NEG	POS	NEG
275174	CC0088	CC88-MSSA	POS	POS	POS	NEG	NEG	NEG	NEG	POS	NEG	NEG	NEG	NEG	NEG	NEG	POS	POS	NEG	POS	POS	NEG	POS	NEG	NEG	NEG	POS	NEG
170971	CC0097	CC97-MSSA	POS	POS	POS	NEG	NEG	NEG	NEG	NEG	POS	NEG	NEG	NEG	NEG	POS	NEG	POS	NEG	AMB	AMB	NEG	POS	NEG	POS	POS	AMB	NEG
112745	CC0097	CC97-MSSA	POS	POS	POS	NEG	NEG	NEG	NEG	NEG	POS	NEG	NEG	NEG	NEG	0	NEG	POS	NEG	POS	POS	NEG	POS	NEG	POS	AMB	POS	NEG
112748	CC0097	CC97-MSSA	POS	POS	POS	NEG	NEG	NEG	NEG	NEG	POS	NEG	NEG	NEG	NEG	0	NEG	POS	NEG	POS	POS	NEG	NEG	NEG	POS	AMB	POS	NEG
112750	CC0097	CC97-MSSA	POS	POS	POS	NEG	NEG	NEG	NEG	NEG	POS	NEG	NEG	NEG	NEG	0	NEG	POS	NEG	AMB	AMB	NEG	POS	NEG	POS	POS	AMB	NEG
112762	CC0097	CC97-MSSA	POS	POS	POS	NEG	NEG	NEG	NEG	NEG	POS	NEG	NEG	NEG	NEG	0	NEG	POS	NEG	POS	POS	NEG	POS	NEG	POS	POS	AMB	NEG
112763	CC0097	CC97-MSSA	POS	POS	POS	NEG	NEG	NEG	NEG	NEG	POS	NEG	NEG	NEG	NEG	0	NEG	POS	NEG	POS	POS	NEG	POS	NEG	POS	AMB	POS	NEG
112764	CC0097	CC97-MSSA	POS	POS	POS	NEG	NEG	NEG	NEG	NEG	POS	NEG	NEG	NEG	NEG	0	NEG	POS	NEG	POS	POS	NEG	POS	NEG	POS	AMB	POS	NEG
170945	CC0097	CC97-MSSA	POS	POS	POS	NEG	NEG	NEG	NEG	NEG	POS	NEG	NEG	NEG	NEG	POS	NEG	POS	NEG	POS	POS	NEG	POS	NEG	POS	POS	AMB	NEG
170954	CC0097	CC97-MSSA	POS	POS	POS	NEG	NEG	NEG	NEG	NEG	POS	NEG	NEG	NEG	NEG	POS	NEG	POS	NEG	POS	AMB	NEG	POS	NEG	POS	POS	AMB	NEG
170958	CC0097	CC97-MSSA	POS	POS	POS	NEG	NEG	NEG	NEG	NEG	POS	NEG	NEG	NEG	NEG	POS	NEG	POS	NEG	AMB	AMB	NEG	POS	NEG	POS	POS	AMB	NEG
170963	CC0097	CC97-MSSA	POS	POS	POS	NEG	NEG	NEG	NEG	NEG	POS	NEG	NEG	NEG	NEG	POS	NEG	POS	NEG	AMB	AMB	NEG	POS	NEG	POS	POS	AMB	NEG
170964	CC0097	CC97-MSSA	POS	POS	POS	NEG	NEG	NEG	NEG	NEG	POS	NEG	NEG	NEG	NEG	POS	NEG	POS	NEG	AMB	AMB	NEG	POS	NEG	POS	POS	AMB	NEG
170965	CC0097	CC97-MSSA	POS	POS	POS	NEG	NEG	NEG	NEG	NEG	POS	NEG	NEG	NEG	NEG	POS	NEG	POS	NEG	AMB	AMB	NEG	POS	NEG	POS	POS	AMB	NEG
170966	CC0097	CC97-MSSA	POS	POS	POS	NEG	NEG	NEG	NEG	NEG	POS	NEG	NEG	NEG	NEG	POS	NEG	POS	NEG	NEG	NEG	NEG	POS	NEG	POS	POS	AMB	NEG
170974	CC0097	CC97-MSSA	POS	POS	POS	NEG	NEG	NEG	NEG	NEG	POS	NEG	NEG	NEG	NEG	POS	NEG	POS	NEG	AMB	AMB	NEG	POS	NEG	POS	POS	AMB	NEG
170976	CC0097	CC97-MSSA	POS	POS	POS	NEG	NEG	NEG	NEG	NEG	POS	NEG	NEG	NEG	NEG	POS	NEG	POS	NEG	NEG	NEG	NEG	POS	NEG	POS	POS	AMB	NEG
112744	CC0097	CC97-MSSA	POS	POS	POS	NEG	NEG	NEG	NEG	NEG	POS	NEG	NEG	NEG	NEG	0	NEG	POS	NEG	AMB	AMB	NEG	POS	NEG	POS	AMB	POS	NEG
112749	CC0097	CC97-MSSA	POS	POS	POS	NEG	NEG	NEG	NEG	NEG	POS	NEG	NEG	NEG	NEG	0	NEG	POS	NEG	POS	POS	NEG	POS	NEG	POS	AMB	POS	NEG
112757	CC0097	CC97-MSSA	POS	POS	POS	NEG	NEG	NEG	NEG	NEG	POS	NEG	NEG	NEG	NEG	0	NEG	POS	AMB	POS	POS	NEG	POS	NEG	POS	AMB	POS	NEG
170973	CC0097	CC97-MSSA	POS	POS	POS	NEG	NEG	NEG	NEG	NEG	POS	NEG	NEG	NEG	NEG	POS	NEG	POS	NEG	POS	AMB	NEG	POS	NEG	POS	POS	AMB	NEG
207756	CC0097	CC97-MSSA	POS	POS	POS	NEG	NEG	NEG	NEG	NEG	POS	NEG	NEG	NEG	NEG	POS	NEG	POS	AMB	POS	POS	NEG	POS	NEG	POS	AMB	POS	NEG
206171	CC0097	CC97-MSSA	POS	POS	POS	NEG	NEG	NEG	NEG	NEG	POS	NEG	NEG	NEG	NEG	POS	NEG	POS	AMB	POS	POS	NEG	POS	NEG	POS	AMB	POS	NEG
275169	CC0097	CC97-MSSA-[ccrAA]	POS	POS	POS	NEG	NEG	NEG	NEG	NEG	POS	NEG	NEG	NEG	NEG	POS	NEG	POS	NEG	AMB	AMB	NEG	NEG	NEG	POS	POS	AMB	NEG
275168	CC0097	CC97-MSSA-[ccrAA]	POS	POS	POS	NEG	NEG	NEG	NEG	NEG	POS	NEG	NEG	NEG	NEG	POS	NEG	POS	NEG	AMB	AMB	NEG	NEG	NEG	POS	POS	AMB	NEG
118320	CC0130	CC130-MSSA (lukF-P83/M+)	POS	POS	POS	POS	NEG	POS	NEG	POS	NEG	AMB	POS	NEG	NEG	NEG	NEG	POS	NEG	POS	POS	NEG	POS	NEG	NEG	POS	POS	NEG
203236	CC0130	CC130-MRSA-XI	POS	POS	POS	POS	NEG	POS	NEG	NEG	NEG	NEG	POS	NEG	NEG	NEG	NEG	POS	NEG	POS	POS	NEG	POS	AMB	NEG	AMB	POS	NEG
204204	CC0130	CC130-MRSA-XI	POS	POS	POS	POS	NEG	POS	NEG	NEG	NEG	NEG	POS	NEG	NEG	NEG	NEG	POS	NEG	POS	POS	NEG	POS	NEG	NEG	AMB	POS	NEG
289122	CC0130	CC130-MRSA-XI	POS	POS	POS	POS	NEG	POS	NEG	POS	NEG	POS	AMB	NEG	NEG	NEG	NEG	NEG	NEG	NEG	NEG	NEG	POS	NEG	NEG	AMB	POS	NEG
225779	CC0130	CC130-MRSA-XI	POS	POS	POS	POS	NEG	POS	NEG	POS	NEG	POS	AMB	NEG	NEG	NEG	NEG	POS	NEG	POS	POS	NEG	POS	NEG	NEG	AMB	POS	NEG
225780	CC0130	CC130-MRSA-XI	POS	POS	POS	POS	NEG	POS	NEG	POS	NEG	POS	AMB	NEG	NEG	NEG	NEG	POS	NEG	POS	POS	NEG	POS	NEG	NEG	AMB	POS	NEG
170959	CC0130	CC130-MRSA-XI	POS	POS	POS	POS	NEG	POS	NEG	POS	NEG	NEG	POS	NEG	NEG	NEG	NEG	POS	NEG	POS	POS	NEG	POS	NEG	NEG	POS	AMB	NEG
179029	CC0130	CC130-MRSA-XI	POS	POS	POS	POS	NEG	POS	NEG	POS	NEG	NEG	POS	NEG	NEG	0	NEG	POS	NEG	POS	POS	NEG	POS	NEG	NEG	AMB	POS	NEG
116254	CC0133	CC133-MSSA	POS	POS	POS	NEG	NEG	NEG	POS	NEG	NEG	NEG	NEG	NEG	NEG	0	POS	POS	NEG	POS	POS	NEG	POS	NEG	NEG	POS	AMB	NEG
207761	CC0133	CC133-MSSA	POS	POS	POS	NEG	POS	NEG	POS	NEG	NEG	NEG	NEG	POS	NEG	NEG	NEG	POS	NEG	POS	POS	NEG	POS	NEG	NEG	POS	AMB	NEG
213103	CC0133	CC133-MSSA	POS	POS	POS	NEG	POS	NEG	POS	NEG	NEG	NEG	NEG	POS	NEG	NEG	NEG	POS	AMB	POS	POS	NEG	POS	AMB	NEG	AMB	POS	POS
275736	CC0133	CC133-MSSA	POS	POS	POS	NEG	POS	NEG	POS	NEG	NEG	NEG	NEG	POS	NEG	NEG	NEG	POS	NEG	POS	POS	NEG	POS	NEG	NEG	POS	AMB	NEG
275737	CC0133	CC133-MSSA	POS	POS	POS	NEG	POS	NEG	POS	NEG	NEG	NEG	NEG	POS	NEG	NEG	NEG	POS	NEG	POS	POS	NEG	POS	NEG	NEG	POS	AMB	NEG
275738	CC0133	CC133-MSSA	POS	POS	POS	NEG	POS	NEG	POS	NEG	NEG	NEG	NEG	POS	NEG	NEG	NEG	POS	NEG	POS	POS	NEG	POS	NEG	NEG	POS	AMB	NEG
275739	CC0133	CC133-MSSA	POS	POS	POS	NEG	POS	NEG	POS	NEG	NEG	NEG	NEG	POS	NEG	NEG	NEG	POS	NEG	POS	POS	NEG	POS	NEG	NEG	POS	AMB	NEG
112755	CC0133	CC133-MSSA	POS	POS	POS	NEG	AMB	NEG	POS	NEG	NEG	NEG	NEG	AMB	NEG	NEG	AMB	POS	NEG	POS	POS	NEG	POS	NEG	NEG	AMB	POS	NEG
225778	CC0398	CC398-MRSA-V/VT	POS	POS	POS	POS	NEG	POS	NEG	POS	NEG	NEG	POS	NEG	NEG	NEG	NEG	AMB	POS	NEG	NEG	NEG	NEG	POS	NEG	NEG	NEG	POS
123159	ST0425	ST425-MSSA	POS	POS	POS	NEG	NEG	NEG	POS	NEG	POS	NEG	NEG	NEG	NEG	POS	NEG	POS	NEG	NEG	NEG	NEG	POS	NEG	NEG	POS	NEG	NEG
86991	ST0425	ST425-MSSA	POS	POS	POS	NEG	NEG	NEG	POS	NEG	POS	NEG	NEG	NEG	NEG	POS	NEG	POS	NEG	NEG	AMB	NEG	POS	NEG	NEG	POS	NEG	NEG
112756	ST0425	ST425-MSSA	POS	POS	POS	NEG	NEG	NEG	POS	NEG	POS	NEG	NEG	NEG	NEG	POS	NEG	POS	AMB	AMB	AMB	NEG	POS	NEG	NEG	POS	NEG	NEG
195207	ST0425	ST425-MSSA	POS	POS	POS	NEG	NEG	NEG	POS	NEG	POS	NEG	NEG	NEG	NEG	POS	NEG	POS	NEG	AMB	POS	NEG	POS	NEG	NEG	POS	NEG	NEG
223609	ST0425	ST425-MSSA	POS	POS	POS	NEG	NEG	NEG	POS	NEG	POS	NEG	NEG	NEG	NEG	POS	NEG	POS	AMB	POS	POS	NEG	POS	POS	NEG	POS	AMB	POS
275740	ST0425	ST425-MSSA	POS	POS	POS	NEG	NEG	NEG	POS	NEG	POS	NEG	NEG	NEG	NEG	POS	NEG	POS	NEG	NEG	AMB	NEG	POS	NEG	NEG	POS	NEG	NEG
174889	ST0425	ST425-MSSA	POS	POS	POS	NEG	NEG	NEG	POS	NEG	POS	NEG	NEG	NEG	NEG	POS	NEG	POS	NEG	NEG	NEG	NEG	POS	NEG	NEG	POS	NEG	NEG
203230	ST0425	ST425-MSSA	POS	POS	POS	NEG	AMB	NEG	POS	NEG	POS	NEG	NEG	NEG	NEG	NEG	POS	POS	AMB	POS	POS	NEG	POS	NEG	NEG	POS	NEG	NEG
203242	ST0425	ST425-MSSA	POS	POS	POS	NEG	NEG	NEG	POS	NEG	POS	NEG	NEG	NEG	NEG	POS	NEG	POS	AMB	POS	POS	NEG	POS	NEG	NEG	POS	NEG	NEG
203243	ST0425	ST425-MSSA	POS	POS	POS	NEG	AMB	NEG	POS	NEG	POS	NEG	NEG	NEG	NEG	NEG	POS	POS	AMB	POS	POS	NEG	POS	AMB	NEG	POS	AMB	POS
207951	ST0425	ST425-MSSA	POS	POS	POS	NEG	NEG	NEG	POS	NEG	POS	NEG	NEG	NEG	NEG	POS	NEG	POS	NEG	NEG	AMB	NEG	POS	NEG	NEG	POS	NEG	NEG
207981	ST0425	ST425-MSSA	POS	POS	POS	NEG	NEG	NEG	POS	NEG	POS	NEG	NEG	NEG	NEG	POS	NEG	POS	NEG	NEG	AMB	NEG	POS	NEG	NEG	POS	NEG	NEG
195212	ST0425	ST425-MSSA	POS	POS	POS	NEG	NEG	NEG	POS	NEG	POS	NEG	NEG	NEG	NEG	POS	NEG	POS	NEG	AMB	AMB	NEG	POS	NEG	NEG	POS	NEG	NEG
195206	ST0425	ST425-MSSA	POS	POS	POS	NEG	NEG	NEG	POS	NEG	POS	NEG	NEG	NEG	NEG	POS	NEG	POS	AMB	POS	POS	NEG	POS	AMB	NEG	POS	NEG	NEG
195202	ST0425	ST425-MSSA	POS	POS	POS	NEG	NEG	NEG	POS	NEG	POS	NEG	NEG	NEG	NEG	POS	NEG	POS	NEG	NEG	AMB	NEG	POS	NEG	NEG	POS	NEG	NEG
216042	ST0425	ST425-MSSA	POS	POS	POS	NEG	NEG	NEG	POS	NEG	POS	NEG	NEG	NEG	NEG	POS	NEG	POS	AMB	POS	POS	NEG	POS	NEG	NEG	POS	NEG	AMB
223608	ST0425	ST425-MSSA	POS	POS	POS	NEG	NEG	NEG	POS	NEG	POS	NEG	NEG	NEG	NEG	POS	NEG	POS	AMB	POS	POS	NEG	POS	AMB	NEG	POS	AMB	POS
223612	ST0425	ST425-MSSA	POS	POS	POS	NEG	NEG	NEG	POS	NEG	POS	NEG	NEG	NEG	NEG	POS	NEG	POS	AMB	POS	POS	NEG	POS	AMB	NEG	POS	AMB	POS
225733	ST0425	ST425-MSSA	POS	POS	POS	NEG	NEG	NEG	POS	NEG	POS	NEG	NEG	NEG	NEG	POS	NEG	POS	AMB	POS	AMB	NEG	POS	NEG	NEG	POS	NEG	NEG
230321	ST0425	ST425-MSSA	POS	POS	POS	NEG	NEG	NEG	POS	NEG	POS	NEG	NEG	NEG	NEG	POS	NEG	POS	AMB	POS	POS	NEG	POS	NEG	NEG	POS	NEG	NEG
232368	ST0425	ST425-MSSA	POS	POS	POS	NEG	NEG	NEG	POS	NEG	POS	NEG	NEG	NEG	NEG	POS	NEG	POS	AMB	AMB	POS	NEG	POS	NEG	NEG	POS	NEG	NEG
204215	ST0425	ST425-MSSA	POS	POS	POS	NEG	NEG	NEG	POS	NEG	POS	NEG	NEG	NEG	NEG	POS	NEG	POS	AMB	POS	POS	NEG	POS	NEG	NEG	POS	NEG	NEG
204218	ST0425	ST425-MSSA	POS	POS	POS	NEG	NEG	NEG	POS	NEG	POS	NEG	NEG	NEG	NEG	POS	NEG	POS	AMB	POS	POS	NEG	POS	NEG	NEG	POS	NEG	NEG
204222	ST0425	ST425-MSSA	POS	POS	POS	NEG	NEG	NEG	POS	NEG	POS	NEG	NEG	NEG	NEG	POS	NEG	POS	AMB	POS	POS	NEG	POS	NEG	NEG	POS	NEG	NEG
204226	ST0425	ST425-MSSA	POS	POS	POS	NEG	NEG	NEG	POS	NEG	POS	NEG	NEG	NEG	NEG	POS	NEG	POS	AMB	AMB	AMB	NEG	POS	NEG	NEG	POS	NEG	NEG
204906	ST0425	ST425-MSSA	POS	POS	POS	NEG	NEG	NEG	POS	NEG	POS	NEG	NEG	NEG	NEG	POS	NEG	POS	AMB	AMB	POS	NEG	POS	NEG	NEG	POS	NEG	NEG
204908	ST0425	ST425-MSSA	POS	POS	POS	NEG	NEG	NEG	POS	NEG	POS	NEG	NEG	NEG	NEG	POS	NEG	POS	AMB	POS	POS	NEG	POS	NEG	NEG	POS	NEG	AMB
206170	ST0425	ST425-MSSA	POS	POS	POS	NEG	NEG	NEG	POS	NEG	POS	NEG	NEG	NEG	NEG	POS	NEG	POS	AMB	AMB	AMB	NEG	POS	NEG	NEG	POS	NEG	NEG
207765	ST0425	ST425-MSSA	POS	POS	POS	NEG	NEG	NEG	POS	NEG	POS	NEG	NEG	NEG	NEG	POS	NEG	POS	NEG	AMB	POS	NEG	POS	NEG	NEG	POS	NEG	NEG
240048	CC599	CC599-MRSA-XI	POS	POS	POS	NEG	AMB	POS	NEG	POS	NEG	NEG	NEG	NEG	NEG	NEG	AMB	POS	NEG	POS	POS	NEG	POS	NEG	NEG	POS	AMB	NEG
225730	CC0692	CC692-MSSA	POS	POS	POS	POS	AMB	POS	NEG	POS	NEG	POS	AMB	NEG	NEG	NEG	NEG	POS	AMB	POS	POS	NEG	POS	NEG	POS	AMB	POS	NEG
112746	CC0692	CC692-MSSA	POS	POS	POS	POS	NEG	POS	NEG	POS	NEG	POS	AMB	NEG	NEG	0	NEG	POS	NEG	POS	POS	NEG	POS	NEG	POS	POS	AMB	NEG
112754	CC0692	CC692-MSSA	POS	POS	POS	POS	NEG	POS	NEG	POS	NEG	POS	AMB	NEG	NEG	NEG	NEG	POS	NEG	POS	POS	NEG	POS	NEG	POS	POS	POS	NEG
112758	CC0692	CC692-MSSA	POS	POS	POS	POS	NEG	POS	NEG	POS	NEG	POS	AMB	NEG	NEG	0	NEG	POS	AMB	POS	POS	NEG	POS	NEG	POS	AMB	POS	NEG
112759	CC0692	CC692-MSSA	POS	POS	POS	POS	NEG	POS	NEG	POS	NEG	POS	AMB	NEG	NEG	0	NEG	POS	NEG	POS	POS	NEG	POS	NEG	POS	AMB	POS	NEG
124745	CC0692	CC692-MSSA	POS	POS	POS	POS	NEG	POS	NEG	POS	NEG	POS	AMB	NEG	NEG	NEG	NEG	POS	NEG	POS	POS	NEG	POS	NEG	POS	AMB	POS	NEG
170952	CC0692	CC692-MSSA	POS	POS	POS	POS	NEG	AMB	NEG	POS	NEG	POS	AMB	NEG	NEG	NEG	NEG	POS	AMB	POS	POS	NEG	POS	NEG	POS	POS	AMB	NEG
170953	CC0692	CC692-MSSA	POS	POS	POS	POS	NEG	AMB	NEG	POS	NEG	POS	AMB	NEG	NEG	NEG	NEG	POS	AMB	POS	POS	NEG	POS	NEG	POS	POS	AMB	NEG
170970	CC0692	CC692-MSSA	POS	POS	POS	POS	NEG	AMB	NEG	POS	NEG	POS	AMB	NEG	NEG	NEG	NEG	POS	NEG	POS	POS	NEG	POS	NEG	POS	POS	AMB	NEG
170950	CC0707	CC707-MSSA	POS	POS	POS	NEG	NEG	NEG	POS	NEG	POS	NEG	NEG	NEG	NEG	POS	NEG	POS	NEG	POS	POS	NEG	POS	NEG	NEG	POS	AMB	NEG
124948	ST0890	ST890-MSSA	POS	POS	POS	NEG	POS	NEG	POS	NEG	NEG	NEG	NEG	POS	NEG	NEG	NEG	POS	AMB	POS	POS	NEG	POS	NEG	NEG	POS	AMB	NEG
220618	CC1956	CC1956-MSSA	POS	POS	POS	NEG	NEG	NEG	POS	NEG	POS	NEG	NEG	NEG	NEG	POS	NEG	POS	AMB	POS	POS	NEG	POS	AMB	NEG	POS	AMB	AMB
112742	ST2425	ST2425-MSSA	POS	POS	POS	NEG	AMB	NEG	POS	NEG	POS	NEG	NEG	AMB	NEG	NEG	AMB	POS	NEG	POS	POS	NEG	POS	NEG	NEG	AMB	POS	NEG
170957	ST2425	ST2425-MSSA	POS	POS	POS	NEG	POS	NEG	POS	NEG	NEG	NEG	NEG	POS	NEG	NEG	NEG	POS	NEG	POS	POS	NEG	POS	NEG	NEG	POS	AMB	NEG
170947	ST2691	ST2691-MSSA	POS	POS	POS	NEG	NEG	NEG	POS	NEG	POS	NEG	NEG	NEG	NEG	POS	NEG	POS	AMB	NEG	NEG	NEG	POS	NEG	NEG	POS	AMB	NEG
170961	ST2691	ST2691-MSSA	POS	POS	POS	NEG	NEG	NEG	POS	NEG	POS	NEG	NEG	NEG	NEG	POS	NEG	POS	AMB	NEG	NEG	NEG	POS	NEG	NEG	POS	AMB	NEG
112753	CC2767	ST2279-MSSA	POS	POS	POS	NEG	NEG	NEG	POS	NEG	POS	NEG	NEG	NEG	NEG	POS	NEG	POS	NEG	AMB	POS	NEG	POS	NEG	POS	POS	POS	NEG
112761	CC2767	ST2279-MSSA	POS	POS	POS	NEG	NEG	NEG	POS	NEG	POS	NEG	NEG	NEG	NEG	POS	NEG	POS	NEG	NEG	POS	NEG	POS	NEG	POS	POS	NEG	NEG
228651	ST2963	ST2963-MSSA	POS	POS	POS	POS	NEG	NEG	POS	NEG	NEG	NEG	NEG	NEG	NEG	NEG	POS	POS	NEG	NEG	AMB	NEG	POS	NEG	POS	AMB	POS	NEG
203244	ST3237	ST3237-MSSA	POS	POS	POS	NEG	NEG	NEG	POS	NEG	POS	NEG	NEG	NEG	NEG	POS	NEG	POS	AMB	AMB	NEG	NEG	POS	NEG	NEG	AMB	POS	NEG

ISOLATE ID	CLONAL COMPLEX (by array)	STRAIN AFFILIATION (by array)	VIRULENCE : STAPHYLOCOCCAL SUPERANTIGEN/ENTEROTOXIN-LIKE GENES (SET/SSL)																					
			staphylococcal superantigen-like protein 6		staphylococcal superantigen-like protein 7			staphylococcal superantigen-like protein 8		staphylococcal superantigen-like protein 9			staphylococcal superantigen-like protein 10			staphylococcal superantigene-like protein 11				staphylococcal exotoxin-like protein, second locus				
			ssl06/set21	ssl06 (NCTC8325+MW2)	ssl07/set1	ssl07/set1 (MRSA252)	ssl07/set1 (AF188836)	ssl08/set12_probe 1	ssl08/set12_probe 2	ssl09/set5_probe 1	ssl09/set5_probe 2	ssl09/set5 (MRSA252)	ssl10/set4	ssl10 (RF122)	ssl10/set4 (MRSA252)	ssl11/set2 (COL)	ssl11+set2(Mu50+N315)	ssl11+set2(MW2+MSSA476)	ssl11/set2 (MRSA252)	setB3	setB3 (MRSA252)	setB2	setB2 (MRSA252)	setB1
203228	CC0001	CC1-MSSA	POS	POS	AMB	POS	AMB	POS	POS	POS	POS	AMB	POS	AMB	AMB	NEG	NEG	POS	NEG	POS	NEG	POS	NEG	POS
203229	CC0001	CC1-MSSA	POS	POS	AMB	POS	AMB	POS	POS	POS	POS	POS	AMB	AMB	POS	NEG	NEG	POS	NEG	POS	NEG	POS	NEG	POS
203233	CC0001	CC1-MSSA	POS	POS	AMB	POS	AMB	POS	POS	POS	POS	NEG	AMB	AMB	POS	NEG	NEG	POS	NEG	POS	NEG	POS	NEG	POS
220334	CC0001	CC1-MSSA	POS	POS	AMB	POS	AMB	POS	POS	POS	POS	NEG	POS	NEG	AMB	NEG	NEG	POS	NEG	POS	NEG	POS	NEG	POS
223621	CC0001	CC1-MSSA	POS	POS	AMB	POS	AMB	POS	POS	POS	POS	NEG	POS	AMB	AMB	NEG	NEG	POS	NEG	POS	NEG	POS	NEG	POS
289123	CC0001	CC1-MRSA-IV, WA MRSA-1/57	POS	POS	POS	AMB	AMB	POS	POS	POS	POS	NEG	POS	AMB	AMB	NEG	NEG	POS	NEG	POS	NEG	POS	NEG	POS
289124	CC0001	CC1-MRSA-IV, WA MRSA-1/57	POS	POS	POS	AMB	NEG	POS	POS	POS	POS	NEG	POS	NEG	AMB	NEG	NEG	POS	NEG	POS	NEG	POS	NEG	POS
275170	CC0005	CC5-MSSA	NEG	NEG	POS	AMB	NEG	POS	POS	POS	POS	NEG	POS	NEG	AMB	NEG	POS	NEG	NEG	POS	NEG	POS	NEG	POS
275171	CC0005	CC5-MSSA	NEG	NEG	POS	NEG	NEG	POS	POS	POS	POS	NEG	POS	NEG	NEG	NEG	POS	NEG	NEG	POS	NEG	POS	NEG	POS
112747	CC0005	CC5-MSSA	NEG	NEG	POS	AMB	NEG	POS	POS	POS	POS	NEG	POS	NEG	AMB	NEG	POS	NEG	NEG	POS	NEG	POS	NEG	POS
112752	CC0006	CC6-MSSA	NEG	NEG	POS	NEG	NEG	POS	POS	POS	POS	NEG	POS	AMB	AMB	NEG	POS	NEG	NEG	POS	NEG	POS	NEG	POS
275178	CC0007	CC7-MSSA	POS	POS	POS	AMB	NEG	POS	POS	POS	POS	NEG	POS	NEG	AMB	NEG	NEG	NEG	NEG	POS	NEG	POS	NEG	POS
275166	CC0008	CC8-MSSA	POS	POS	POS	AMB	NEG	POS	POS	POS	POS	NEG	POS	NEG	NEG	POS	NEG	NEG	NEG	POS	NEG	POS	NEG	POS
275176	CC0008	CC8-MSSA	POS	POS	POS	AMB	NEG	POS	POS	POS	POS	NEG	POS	NEG	AMB	POS	NEG	NEG	NEG	POS	NEG	POS	NEG	POS
275177	CC0008	CC8-MSSA	POS	POS	POS	NEG	NEG	POS	POS	POS	POS	NEG	POS	NEG	AMB	POS	NEG	NEG	NEG	POS	NEG	POS	NEG	POS
275167	CC0009	CC9-MSSA	POS	POS	POS	NEG	NEG	POS	POS	POS	POS	NEG	POS	NEG	NEG	NEG	NEG	NEG	NEG	POS	NEG	POS	NEG	POS
170968	CC0012	CC12-MSSA	NEG	NEG	POS	NEG	NEG	POS	POS	POS	POS	NEG	POS	NEG	AMB	POS	NEG	NEG	NEG	POS	NEG	POS	NEG	POS
275175	CC0015	CC15-MSSA	POS	POS	POS	AMB	NEG	NEG	POS	POS	POS	NEG	AMB	AMB	NEG	NEG	NEG	NEG	POS	POS	NEG	POS	NEG	POS
170949	CC0015	CC15-MSSA	POS	POS	POS	AMB	NEG	AMB	POS	POS	POS	NEG	AMB	POS	NEG	NEG	NEG	NEG	POS	POS	NEG	AMB	NEG	POS
228658	CC0022	CC22-MSSA	NEG	NEG	AMB	AMB	POS	NEG	NEG	POS	POS	NEG	POS	NEG	AMB	NEG	NEG	NEG	NEG	NEG	NEG	NEG	NEG	NEG
289125	CC0022	CC22-MRSA-IV (PVL+)	NEG	NEG	AMB	AMB	POS	NEG	NEG	POS	POS	NEG	POS	NEG	NEG	NEG	NEG	NEG	NEG	NEG	NEG	NEG	NEG	NEG
289126	CC0022	CC22-MRSA-IV (PVL+)	NEG	NEG	AMB	AMB	POS	NEG	NEG	POS	POS	NEG	POS	NEG	NEG	NEG	NEG	NEG	NEG	NEG	NEG	NEG	NEG	NEG
289127	CC0022	CC22-MRSA-IV (PVL+)	NEG	NEG	AMB	AMB	POS	NEG	NEG	POS	POS	NEG	POS	NEG	NEG	NEG	NEG	NEG	NEG	NEG	NEG	NEG	NEG	NEG
131939	CC0025	CC25-MSSA	NEG	NEG	POS	NEG	NEG	POS	POS	POS	POS	NEG	POS	AMB	AMB	NEG	NEG	NEG	NEG	POS	NEG	POS	NEG	POS
275165	CC0030	CC30-MSSA (lukF-P83/lukM+)	NEG	NEG	NEG	POS	NEG	NEG	NEG	NEG	NEG	POS	AMB	NEG	POS	NEG	NEG	NEG	POS	NEG	POS	NEG	POS	POS
275741	CC0030	CC30-MSSA	NEG	NEG	NEG	POS	NEG	NEG	NEG	NEG	NEG	POS	AMB	NEG	POS	NEG	NEG	NEG	POS	NEG	POS	NEG	POS	POS
124492	CC0049	CC49-MSSA (lukF-P83/lukM+)	POS	POS	POS	AMB	NEG	POS	POS	POS	POS	NEG	AMB	POS	NEG	NEG	NEG	NEG	NEG	POS	NEG	POS	NEG	POS
220616	CC0049	CC49-MSSA (lukF-P83/lukM+)	POS	POS	POS	AMB	AMB	POS	POS	POS	POS	NEG	POS	AMB	AMB	NEG	NEG	NEG	NEG	POS	NEG	POS	NEG	POS
228649	CC0049	CC49-MSSA (lukF-P83/lukM+)	POS	POS	POS	AMB	NEG	POS	POS	POS	POS	NEG	POS	AMB	AMB	NEG	NEG	NEG	NEG	POS	NEG	POS	NEG	POS
190994	CC0059	CC59-MSSA	NEG	NEG	POS	NEG	NEG	POS	POS	POS	POS	NEG	AMB	POS	NEG	NEG	NEG	NEG	NEG	POS	NEG	POS	NEG	POS
275172	CC0088	CC88-MSSA	POS	POS	POS	NEG	NEG	POS	POS	POS	POS	NEG	POS	NEG	NEG	NEG	NEG	NEG	NEG	POS	NEG	POS	NEG	POS
275173	CC0088	CC88-MSSA	POS	POS	POS	AMB	NEG	POS	POS	POS	POS	NEG	POS	NEG	AMB	NEG	NEG	NEG	NEG	POS	NEG	POS	NEG	POS
275174	CC0088	CC88-MSSA	POS	POS	POS	AMB	NEG	POS	POS	POS	POS	NEG	POS	NEG	AMB	NEG	NEG	NEG	NEG	POS	NEG	POS	NEG	POS
170971	CC0097	CC97-MSSA	NEG	NEG	POS	AMB	NEG	POS	POS	POS	POS	NEG	POS	NEG	AMB	NEG	NEG	NEG	NEG	POS	NEG	POS	NEG	POS
112745	CC0097	CC97-MSSA	NEG	NEG	POS	AMB	NEG	POS	POS	POS	POS	NEG	POS	NEG	AMB	NEG	NEG	NEG	NEG	POS	NEG	POS	NEG	POS
112748	CC0097	CC97-MSSA	NEG	NEG	POS	AMB	NEG	POS	POS	POS	POS	NEG	POS	NEG	AMB	NEG	NEG	NEG	NEG	POS	NEG	POS	NEG	POS
112750	CC0097	CC97-MSSA	NEG	NEG	POS	AMB	NEG	POS	POS	POS	POS	NEG	POS	NEG	AMB	NEG	NEG	NEG	NEG	POS	NEG	POS	NEG	POS
112762	CC0097	CC97-MSSA	NEG	NEG	POS	AMB	NEG	POS	POS	POS	POS	NEG	POS	NEG	AMB	NEG	NEG	NEG	NEG	POS	NEG	POS	NEG	POS
112763	CC0097	CC97-MSSA	NEG	AMB	POS	AMB	NEG	POS	POS	POS	POS	NEG	POS	NEG	AMB	NEG	NEG	NEG	NEG	POS	NEG	POS	NEG	POS
112764	CC0097	CC97-MSSA	NEG	NEG	POS	AMB	NEG	POS	POS	POS	POS	NEG	POS	NEG	AMB	NEG	NEG	NEG	NEG	POS	NEG	POS	NEG	POS
170945	CC0097	CC97-MSSA	NEG	NEG	POS	AMB	NEG	POS	POS	POS	POS	NEG	POS	NEG	AMB	NEG	NEG	NEG	NEG	POS	NEG	POS	NEG	POS
170954	CC0097	CC97-MSSA	NEG	NEG	POS	AMB	NEG	POS	POS	POS	POS	NEG	POS	NEG	AMB	NEG	NEG	NEG	NEG	POS	NEG	POS	NEG	POS
170958	CC0097	CC97-MSSA	NEG	NEG	POS	AMB	NEG	POS	POS	POS	POS	NEG	POS	NEG	NEG	NEG	NEG	NEG	NEG	POS	NEG	POS	NEG	POS
170963	CC0097	CC97-MSSA	NEG	NEG	POS	AMB	NEG	POS	POS	POS	POS	NEG	POS	NEG	NEG	NEG	NEG	NEG	NEG	POS	NEG	POS	NEG	POS
170964	CC0097	CC97-MSSA	NEG	NEG	POS	AMB	NEG	POS	POS	POS	POS	NEG	POS	NEG	AMB	NEG	NEG	NEG	NEG	POS	NEG	POS	NEG	POS
170965	CC0097	CC97-MSSA	NEG	NEG	POS	AMB	NEG	POS	POS	POS	POS	NEG	POS	NEG	NEG	NEG	NEG	NEG	NEG	POS	NEG	POS	NEG	POS
170966	CC0097	CC97-MSSA	NEG	NEG	POS	AMB	NEG	POS	POS	POS	POS	NEG	POS	NEG	AMB	NEG	NEG	NEG	NEG	POS	NEG	POS	NEG	POS
170974	CC0097	CC97-MSSA	NEG	NEG	POS	AMB	NEG	POS	POS	POS	POS	NEG	POS	NEG	NEG	NEG	NEG	NEG	NEG	POS	NEG	POS	NEG	POS
170976	CC0097	CC97-MSSA	NEG	NEG	POS	AMB	NEG	POS	POS	POS	POS	NEG	POS	NEG	NEG	NEG	NEG	NEG	NEG	POS	NEG	POS	NEG	POS
112744	CC0097	CC97-MSSA	NEG	NEG	POS	AMB	NEG	POS	POS	POS	POS	NEG	POS	NEG	AMB	NEG	NEG	NEG	NEG	POS	NEG	POS	NEG	POS
112749	CC0097	CC97-MSSA	NEG	AMB	POS	AMB	AMB	POS	POS	POS	POS	NEG	POS	AMB	AMB	NEG	NEG	NEG	NEG	POS	NEG	POS	NEG	POS
112757	CC0097	CC97-MSSA	NEG	AMB	POS	AMB	AMB	POS	POS	POS	POS	NEG	POS	NEG	AMB	NEG	NEG	NEG	NEG	POS	NEG	POS	NEG	POS
170973	CC0097	CC97-MSSA	NEG	NEG	POS	AMB	NEG	POS	POS	POS	POS	NEG	POS	NEG	AMB	NEG	NEG	NEG	NEG	POS	NEG	POS	NEG	POS
207756	CC0097	CC97-MSSA	NEG	NEG	POS	AMB	NEG	POS	POS	POS	POS	NEG	POS	NEG	AMB	NEG	NEG	NEG	NEG	POS	NEG	POS	NEG	POS
206171	CC0097	CC97-MSSA	NEG	NEG	POS	AMB	NEG	POS	POS	POS	POS	NEG	POS	NEG	AMB	NEG	NEG	NEG	NEG	POS	NEG	POS	NEG	POS
275169	CC0097	CC97-MSSA-[ccrAA]	NEG	NEG	POS	AMB	NEG	POS	POS	POS	POS	NEG	POS	NEG	AMB	NEG	NEG	NEG	NEG	POS	NEG	POS	NEG	POS
275168	CC0097	CC97-MSSA-[ccrAA]	NEG	NEG	POS	AMB	NEG	POS	POS	POS	POS	NEG	POS	NEG	AMB	NEG	NEG	NEG	NEG	POS	NEG	POS	NEG	POS
118320	CC0130	CC130-MSSA (lukF-P83/M+)	NEG	NEG	POS	POS	AMB	POS	POS	POS	POS	NEG	POS	POS	AMB	NEG	NEG	NEG	NEG	POS	NEG	POS	NEG	POS
203236	CC0130	CC130-MRSA-XI	NEG	AMB	POS	POS	NEG	POS	POS	POS	POS	NEG	POS	AMB	POS	NEG	NEG	NEG	NEG	POS	NEG	POS	NEG	POS
204204	CC0130	CC130-MRSA-XI	NEG	NEG	POS	AMB	AMB	POS	POS	POS	POS	NEG	POS	AMB	AMB	NEG	NEG	NEG	NEG	POS	NEG	POS	NEG	POS
289122	CC0130	CC130-MRSA-XI	NEG	NEG	AMB	POS	AMB	POS	POS	POS	POS	NEG	POS	AMB	AMB	NEG	NEG	NEG	NEG	POS	NEG	POS	NEG	POS
225779	CC0130	CC130-MRSA-XI	NEG	NEG	AMB	POS	AMB	POS	POS	POS	POS	NEG	POS	AMB	AMB	NEG	NEG	NEG	NEG	POS	NEG	POS	NEG	POS
225780	CC0130	CC130-MRSA-XI	NEG	NEG	POS	AMB	AMB	POS	POS	POS	POS	NEG	POS	AMB	AMB	NEG	NEG	NEG	NEG	POS	NEG	POS	NEG	POS
170959	CC0130	CC130-MRSA-XI	NEG	NEG	POS	AMB	NEG	AMB	AMB	POS	POS	NEG	AMB	POS	NEG	NEG	NEG	NEG	NEG	POS	NEG	POS	NEG	POS
179029	CC0130	CC130-MRSA-XI	NEG	NEG	POS	AMB	AMB	POS	POS	POS	POS	NEG	POS	AMB	NEG	NEG	NEG	NEG	NEG	POS	NEG	POS	NEG	POS
116254	CC0133	CC133-MSSA	POS	POS	POS	NEG	NEG	POS	POS	POS	POS	NEG	AMB	POS	NEG	NEG	NEG	NEG	NEG	POS	NEG	POS	NEG	POS
207761	CC0133	CC133-MSSA	POS	POS	POS	NEG	NEG	POS	POS	POS	POS	NEG	POS	AMB	NEG	NEG	NEG	NEG	NEG	POS	NEG	AMB	NEG	POS
213103	CC0133	CC133-MSSA	NEG	NEG	AMB	POS	AMB	POS	POS	POS	POS	NEG	POS	AMB	AMB	NEG	NEG	NEG	NEG	POS	NEG	POS	NEG	POS
275736	CC0133	CC133-MSSA	POS	POS	POS	NEG	NEG	POS	POS	POS	POS	NEG	AMB	POS	NEG	NEG	NEG	NEG	NEG	POS	NEG	POS	NEG	POS
275737	CC0133	CC133-MSSA	POS	POS	POS	NEG	NEG	POS	POS	POS	POS	NEG	AMB	POS	NEG	NEG	NEG	NEG	NEG	POS	NEG	POS	NEG	POS
275738	CC0133	CC133-MSSA	POS	POS	POS	AMB	NEG	POS	POS	POS	POS	NEG	AMB	POS	NEG	NEG	NEG	NEG	NEG	POS	NEG	POS	NEG	POS
275739	CC0133	CC133-MSSA	POS	POS	POS	NEG	NEG	POS	POS	POS	POS	NEG	AMB	POS	NEG	NEG	NEG	NEG	NEG	POS	NEG	POS	NEG	POS
112755	CC0133	CC133-MSSA	POS	POS	POS	AMB	NEG	POS	POS	POS	POS	NEG	POS	AMB	NEG	NEG	NEG	NEG	NEG	POS	NEG	POS	NEG	POS
225778	CC0398	CC398-MRSA-V/VT	NEG	NEG	AMB	POS	AMB	NEG	NEG	NEG	NEG	POS	POS	NEG	AMB	NEG	NEG	NEG	POS	NEG	POS	NEG	POS	POS
123159	ST0425	ST425-MSSA	NEG	NEG	POS	NEG	NEG	NEG	NEG	POS	POS	NEG	AMB	POS	NEG	NEG	NEG	NEG	NEG	POS	NEG	POS	NEG	POS
86991	ST0425	ST425-MSSA	NEG	NEG	POS	AMB	NEG	POS	NEG	NEG	NEG	NEG	POS	POS	NEG	NEG	NEG	NEG	NEG	POS	NEG	POS	NEG	POS
112756	ST0425	ST425-MSSA	NEG	NEG	POS	AMB	AMB	POS	POS	POS	POS	NEG	POS	AMB	AMB	NEG	NEG	NEG	NEG	POS	NEG	POS	NEG	POS
195207	ST0425	ST425-MSSA	NEG	NEG	POS	AMB	NEG	POS	POS	POS	POS	NEG	POS	AMB	AMB	NEG	NEG	NEG	NEG	POS	NEG	POS	NEG	POS
223609	ST0425	ST425-MSSA	NEG	AMB	AMB	POS	AMB	POS	POS	POS	POS	NEG	POS	AMB	AMB	NEG	NEG	NEG	NEG	POS	NEG	POS	NEG	POS
275740	ST0425	ST425-MSSA	NEG	NEG	POS	AMB	NEG	POS	POS	POS	POS	NEG	POS	AMB	NEG	NEG	NEG	NEG	NEG	POS	NEG	POS	NEG	POS
174889	ST0425	ST425-MSSA	NEG	NEG	POS	AMB	NEG	POS	POS	POS	POS	NEG	AMB	POS	NEG	NEG	NEG	NEG	NEG	POS	NEG	POS	NEG	POS
203230	ST0425	ST425-MSSA	NEG	NEG	AMB	POS	AMB	POS	POS	POS	POS	NEG	POS	AMB	AMB	NEG	NEG	NEG	NEG	POS	NEG	POS	NEG	POS
203242	ST0425	ST425-MSSA	NEG	NEG	AMB	POS	AMB	POS	POS	POS	POS	NEG	POS	AMB	AMB	NEG	NEG	NEG	NEG	POS	NEG	POS	NEG	POS
203243	ST0425	ST425-MSSA	NEG	NEG	AMB	POS	AMB	POS	POS	POS	POS	NEG	POS	AMB	AMB	NEG	NEG	NEG	NEG	POS	NEG	POS	NEG	POS
207951	ST0425	ST425-MSSA	NEG	NEG	POS	NEG	NEG	AMB	AMB	POS	POS	NEG	AMB	POS	NEG	NEG	NEG	NEG	NEG	POS	NEG	POS	NEG	POS
207981	ST0425	ST425-MSSA	NEG	NEG	POS	NEG	NEG	AMB	NEG	NEG	NEG	NEG	AMB	POS	AMB	NEG	NEG	NEG	NEG	POS	NEG	POS	NEG	POS
195212	ST0425	ST425-MSSA	NEG	NEG	POS	AMB	NEG	POS	POS	POS	POS	NEG	POS	AMB	AMB	NEG	NEG	NEG	NEG	POS	NEG	POS	NEG	POS
195206	ST0425	ST425-MSSA	NEG	AMB	AMB	POS	AMB	POS	POS	POS	POS	NEG	AMB	AMB	AMB	NEG	NEG	NEG	NEG	POS	NEG	POS	NEG	POS
195202	ST0425	ST425-MSSA	NEG	NEG	AMB	POS	AMB	POS	POS	POS	POS	NEG	POS	AMB	AMB	NEG	NEG	NEG	NEG	POS	NEG	POS	NEG	POS
216042	ST0425	ST425-MSSA	NEG	NEG	AMB	POS	AMB	POS	POS	POS	POS	NEG	POS	AMB	AMB	NEG	NEG	NEG	NEG	POS	NEG	POS	NEG	POS
223608	ST0425	ST425-MSSA	NEG	POS	AMB	POS	AMB	POS	POS	POS	POS	NEG	POS	AMB	AMB	NEG	NEG	NEG	NEG	POS	NEG	POS	NEG	POS
223612	ST0425	ST425-MSSA	NEG	AMB	AMB	POS	AMB	POS	POS	POS	POS	NEG	POS	AMB	AMB	NEG	NEG	NEG	NEG	POS	NEG	POS	NEG	POS
225733	ST0425	ST425-MSSA	NEG	NEG	AMB	POS	AMB	POS	POS	POS	POS	NEG	POS	AMB	AMB	NEG	NEG	NEG	NEG	POS	NEG	POS	NEG	POS
230321	ST0425	ST425-MSSA	NEG	NEG	POS	AMB	AMB	POS	POS	POS	POS	NEG	AMB	AMB	AMB	NEG	NEG	NEG	NEG	POS	NEG	POS	NEG	POS
232368	ST0425	ST425-MSSA	NEG	NEG	POS	AMB	AMB	POS	POS	POS	POS	NEG	POS	AMB	AMB	NEG	NEG	NEG	NEG	POS	NEG	POS	NEG	POS
204215	ST0425	ST425-MSSA	NEG	NEG	AMB	POS	AMB	POS	POS	POS	POS	NEG	POS	AMB	AMB	NEG	NEG	NEG	NEG	POS	NEG	POS	NEG	POS
204218	ST0425	ST425-MSSA	NEG	NEG	AMB	POS	AMB	POS	POS	POS	POS	NEG	POS	AMB	AMB	NEG	NEG	NEG	NEG	POS	NEG	POS	NEG	POS
204222	ST0425	ST425-MSSA	NEG	NEG	AMB	POS	AMB	POS	POS	POS	POS	NEG	POS	AMB	AMB	NEG	NEG	NEG	NEG	POS	NEG	POS	NEG	POS
204226	ST0425	ST425-MSSA	NEG	NEG	POS	AMB	NEG	POS	POS	POS	POS	NEG	POS	AMB	AMB	NEG	NEG	NEG	NEG	POS	NEG	POS	NEG	POS
204906	ST0425	ST425-MSSA	NEG	NEG	AMB	POS	AMB	POS	POS	POS	POS	NEG	POS	AMB	AMB	NEG	NEG	NEG	NEG	POS	NEG	POS	NEG	POS
204908	ST0425	ST425-MSSA	NEG	NEG	AMB	POS	AMB	POS	POS	POS	POS	NEG	AMB	AMB	AMB	NEG	NEG	NEG	NEG	POS	AMB	POS	NEG	POS
206170	ST0425	ST425-MSSA	NEG	NEG	POS	AMB	NEG	POS	POS	POS	POS	NEG	POS	AMB	AMB	NEG	NEG	NEG	NEG	POS	NEG	POS	NEG	POS
207765	ST0425	ST425-MSSA	NEG	NEG	POS	AMB	NEG	POS	POS	POS	POS	NEG	POS	AMB	AMB	NEG	NEG	NEG	NEG	POS	NEG	POS	NEG	POS
240048	CC599	CC599-MRSA-XI	NEG	NEG	POS	AMB	NEG	POS	POS	POS	POS	NEG	POS	AMB	NEG	NEG	NEG	NEG	NEG	POS	NEG	POS	NEG	POS
225730	CC0692	CC692-MSSA	NEG	AMB	AMB	POS	AMB	POS	POS	POS	POS	NEG	POS	AMB	AMB	NEG	NEG	NEG	AMB	POS	NEG	POS	NEG	POS
112746	CC0692	CC692-MSSA	NEG	NEG	POS	AMB	NEG	POS	POS	POS	POS	NEG	POS	NEG	AMB	NEG	NEG	NEG	NEG	POS	NEG	POS	NEG	POS
112754	CC0692	CC692-MSSA	NEG	NEG	POS	AMB	NEG	POS	POS	POS	POS	NEG	POS	NEG	AMB	NEG	NEG	NEG	NEG	POS	NEG	POS	NEG	POS
112758	CC0692	CC692-MSSA	NEG	NEG	POS	AMB	AMB	POS	POS	POS	POS	NEG	POS	AMB	AMB	NEG	NEG	NEG	AMB	POS	NEG	POS	NEG	POS
112759	CC0692	CC692-MSSA	NEG	NEG	POS	AMB	NEG	POS	POS	POS	POS	NEG	POS	NEG	AMB	NEG	NEG	NEG	AMB	POS	NEG	POS	NEG	POS
124745	CC0692	CC692-MSSA	NEG	NEG	POS	AMB	NEG	POS	POS	POS	POS	NEG	POS	NEG	AMB	NEG	NEG	NEG	NEG	POS	NEG	POS	NEG	POS
170952	CC0692	CC692-MSSA	NEG	NEG	POS	AMB	AMB	POS	POS	POS	POS	NEG	POS	NEG	AMB	NEG	NEG	NEG	NEG	POS	NEG	POS	NEG	POS
170953	CC0692	CC692-MSSA	NEG	NEG	POS	AMB	NEG	POS	POS	POS	POS	NEG	POS	NEG	AMB	NEG	NEG	NEG	NEG	POS	NEG	POS	NEG	POS
170970	CC0692	CC692-MSSA	NEG	NEG	POS	AMB	NEG	POS	POS	POS	POS	NEG	POS	NEG	AMB	NEG	NEG	NEG	NEG	POS	NEG	POS	NEG	POS
170950	CC0707	CC707-MSSA	NEG	NEG	POS	AMB	NEG	AMB	AMB	POS	POS	NEG	AMB	POS	NEG	NEG	POS	NEG	NEG	POS	NEG	POS	NEG	POS
124948	ST0890	ST890-MSSA	NEG	NEG	POS	AMB	NEG	NEG	POS	AMB	AMB	NEG	AMB	POS	NEG	NEG	NEG	NEG	NEG	POS	NEG	POS	NEG	POS
220618	CC1956	CC1956-MSSA	NEG	NEG	AMB	POS	AMB	POS	POS	POS	POS	NEG	POS	AMB	AMB	NEG	NEG	NEG	NEG	POS	NEG	POS	NEG	POS
112742	ST2425	ST2425-MSSA	NEG	NEG	POS	AMB	NEG	POS	POS	POS	POS	NEG	POS	AMB	NEG	NEG	NEG	NEG	NEG	POS	NEG	POS	NEG	POS
170957	ST2425	ST2425-MSSA	NEG	NEG	POS	AMB	NEG	NEG	AMB	POS	POS	NEG	AMB	POS	NEG	NEG	NEG	NEG	NEG	POS	NEG	POS	NEG	POS
170947	ST2691	ST2691-MSSA	NEG	NEG	NEG	POS	NEG	NEG	NEG	POS	POS	NEG	NEG	AMB	NEG	NEG	NEG	NEG	NEG	POS	NEG	POS	NEG	POS
170961	ST2691	ST2691-MSSA	NEG	NEG	NEG	POS	NEG	NEG	NEG	POS	POS	NEG	NEG	AMB	NEG	NEG	NEG	NEG	NEG	POS	NEG	POS	NEG	POS
112753	CC2767	ST2279-MSSA	POS	POS	POS	AMB	NEG	POS	POS	POS	POS	NEG	POS	AMB	NEG	NEG	NEG	NEG	NEG	POS	NEG	POS	NEG	POS
112761	CC2767	ST2279-MSSA	POS	POS	POS	AMB	NEG	POS	POS	POS	POS	NEG	POS	AMB	NEG	NEG	NEG	NEG	NEG	POS	NEG	POS	NEG	POS
228651	ST2963	ST2963-MSSA	NEG	NEG	POS	AMB	NEG	AMB	POS	POS	POS	NEG	POS	AMB	NEG	NEG	NEG	NEG	NEG	POS	NEG	POS	NEG	POS
203244	ST3237	ST3237-MSSA	NEG	NEG	POS	NEG	NEG	AMB	AMB	POS	POS	NEG	POS	NEG	AMB	NEG	NEG	NEG	NEG	POS	NEG	POS	NEG	POS

ISOLATE ID	CLONAL COMPLEX (by array)	STRAIN AFFILIATION (by array)	CAPSULE- AND BIOFILM-ASSOCIATED GENES																	ADHAESION FACTORS / MSCRAMM GENES						
			Capsule type 1	capsular poly-saccharide synthesis enzyme	O-antigen poly-merase	capsular poly-saccharide biosyn-thesis protein	Capsule type 5	capsular poly-saccharide synthesis enzyme	O-antigen poly-merase	capsular poly-saccharide biosyn-thesis protein	Capsule type 8	capsular poly-saccharide synthesis enzyme	capsular poly-saccharide biosyn-thesis protein	O-antigen poly-merase	capsular poly-saccharide biosyn-thesis protein	intercellular adhesion protein A	intercellular adhesion protein C	biofilm PIA synthesis protein D	surface protein involved in biofilm formation	bone sialoprotein-binding protein						
			cap 1	capH1	capJ1	capK1	cap 5	capH5	capJ5	capK5	cap 8	capH8	capI8	capJ8	capK8	icaA	icaC	icaD	bap	bbp	bbp (cons)	bbp (COL+MW2)	bbp (MRSA252)	bbp (Mu50)	bbp (RF122)	bbp (ST45)
203228	CC0001	CC1-MSSA	NEG	NEG	NEG	AMB	NEG	NEG	NEG	NEG	POS	POS	POS	POS	POS	POS	POS	POS	NEG	POS	POS	POS	NEG	AMB	NEG	AMB
203229	CC0001	CC1-MSSA	NEG	NEG	NEG	POS	AMB	AMB	AMB	NEG	POS	POS	POS	POS	POS	POS	POS	POS	NEG	POS	POS	POS	NEG	AMB	NEG	AMB
203233	CC0001	CC1-MSSA	NEG	NEG	NEG	AMB	NEG	NEG	NEG	NEG	POS	POS	POS	POS	POS	POS	POS	POS	NEG	POS	POS	POS	NEG	AMB	NEG	NEG
220334	CC0001	CC1-MSSA	NEG	NEG	NEG	NEG	NEG	NEG	NEG	NEG	POS	POS	POS	POS	POS	POS	POS	POS	NEG	POS	POS	POS	NEG	NEG	NEG	NEG
223621	CC0001	CC1-MSSA	NEG	NEG	NEG	NEG	NEG	NEG	NEG	NEG	POS	POS	POS	POS	POS	POS	POS	POS	NEG	POS	POS	POS	NEG	AMB	AMB	NEG
289123	CC0001	CC1-MRSA-IV, WA MRSA-1/57	NEG	NEG	NEG	NEG	NEG	NEG	NEG	NEG	POS	POS	POS	POS	POS	POS	POS	POS	NEG	POS	POS	POS	NEG	AMB	NEG	NEG
289124	CC0001	CC1-MRSA-IV, WA MRSA-1/57	NEG	NEG	NEG	NEG	NEG	NEG	NEG	NEG	POS	POS	POS	POS	POS	POS	POS	POS	NEG	POS	POS	POS	NEG	NEG	NEG	NEG
275170	CC0005	CC5-MSSA	NEG	NEG	NEG	NEG	POS	POS	POS	POS	NEG	NEG	NEG	NEG	NEG	POS	POS	POS	NEG	POS	POS	NEG	NEG	POS	NEG	NEG
275171	CC0005	CC5-MSSA	NEG	NEG	NEG	NEG	POS	POS	POS	POS	NEG	NEG	NEG	NEG	NEG	POS	POS	POS	NEG	POS	POS	NEG	NEG	POS	NEG	NEG
112747	CC0005	CC5-MSSA	NEG	NEG	NEG	NEG	POS	POS	POS	POS	NEG	NEG	AMB	NEG	NEG	POS	POS	POS	NEG	POS	POS	NEG	NEG	POS	NEG	NEG
112752	CC0006	CC6-MSSA	NEG	NEG	NEG	NEG	NEG	NEG	NEG	NEG	POS	POS	POS	POS	POS	POS	POS	POS	NEG	POS	POS	POS	NEG	NEG	NEG	AMB
275178	CC0007	CC7-MSSA	NEG	NEG	NEG	NEG	NEG	NEG	NEG	NEG	POS	POS	POS	POS	POS	POS	POS	POS	NEG	POS	POS	POS	AMB	NEG	NEG	NEG
275166	CC0008	CC8-MSSA	NEG	NEG	NEG	NEG	POS	POS	POS	POS	NEG	NEG	NEG	NEG	NEG	POS	POS	POS	NEG	NEG	NEG	NEG	NEG	NEG	NEG	NEG
275176	CC0008	CC8-MSSA	NEG	NEG	NEG	NEG	POS	POS	POS	POS	NEG	NEG	NEG	NEG	NEG	POS	POS	POS	NEG	NEG	NEG	NEG	NEG	NEG	NEG	NEG
275177	CC0008	CC8-MSSA	NEG	NEG	NEG	NEG	POS	POS	POS	POS	NEG	NEG	NEG	NEG	NEG	POS	POS	POS	NEG	POS	POS	POS	NEG	NEG	NEG	NEG
275167	CC0009	CC9-MSSA	NEG	NEG	NEG	NEG	POS	POS	POS	POS	NEG	NEG	NEG	NEG	NEG	POS	POS	POS	NEG	POS	POS	NEG	NEG	POS	NEG	NEG
170968	CC0012	CC12-MSSA	NEG	NEG	NEG	NEG	NEG	NEG	NEG	NEG	POS	POS	POS	POS	POS	POS	POS	POS	NEG	POS	POS	NEG	NEG	POS	NEG	NEG
275175	CC0015	CC15-MSSA	NEG	NEG	NEG	NEG	NEG	NEG	NEG	NEG	POS	POS	POS	POS	POS	POS	POS	POS	NEG	POS	POS	NEG	NEG	POS	NEG	NEG
170949	CC0015	CC15-MSSA	NEG	NEG	NEG	NEG	NEG	NEG	NEG	NEG	POS	POS	POS	POS	POS	POS	POS	POS	NEG	POS	POS	NEG	NEG	POS	NEG	NEG
228658	CC0022	CC22-MSSA	NEG	NEG	NEG	NEG	POS	POS	POS	POS	NEG	NEG	NEG	NEG	NEG	POS	POS	POS	NEG	NEG	NEG	NEG	NEG	NEG	NEG	NEG
289125	CC0022	CC22-MRSA-IV (PVL+)	NEG	NEG	NEG	NEG	POS	POS	POS	POS	NEG	NEG	NEG	NEG	NEG	POS	POS	POS	NEG	POS	POS	NEG	NEG	POS	NEG	NEG
289126	CC0022	CC22-MRSA-IV (PVL+)	NEG	NEG	NEG	NEG	POS	POS	POS	POS	NEG	NEG	NEG	NEG	NEG	POS	POS	POS	NEG	POS	POS	NEG	NEG	POS	NEG	NEG
289127	CC0022	CC22-MRSA-IV (PVL+)	NEG	NEG	NEG	NEG	POS	POS	POS	POS	NEG	NEG	NEG	NEG	NEG	POS	POS	POS	NEG	POS	POS	NEG	NEG	POS	NEG	NEG
131939	CC0025	CC25-MSSA	NEG	NEG	NEG	NEG	POS	POS	POS	POS	NEG	NEG	NEG	NEG	NEG	POS	POS	POS	NEG	POS	POS	POS	NEG	NEG	NEG	NEG
275165	CC0030	CC30-MSSA (lukF-P83/lukM+)	NEG	NEG	NEG	NEG	NEG	NEG	NEG	NEG	POS	POS	POS	POS	POS	POS	POS	POS	NEG	POS	POS	NEG	POS	NEG	NEG	NEG
275741	CC0030	CC30-MSSA	NEG	NEG	NEG	NEG	NEG	NEG	NEG	NEG	POS	POS	POS	POS	POS	POS	POS	POS	NEG	POS	POS	NEG	POS	NEG	NEG	NEG
124492	CC0049	CC49-MSSA (lukF-P83/lukM+)	NEG	NEG	NEG	NEG	POS	NEG	POS	POS	NEG	NEG	NEG	NEG	NEG	POS	POS	POS	NEG	POS	POS	NEG	NEG	NEG	NEG	NEG
220616	CC0049	CC49-MSSA (lukF-P83/lukM+)	NEG	NEG	NEG	NEG	POS	POS	POS	POS	NEG	NEG	NEG	NEG	NEG	POS	POS	POS	NEG	POS	POS	NEG	NEG	POS	NEG	NEG
228649	CC0049	CC49-MSSA (lukF-P83/lukM+)	NEG	NEG	NEG	NEG	POS	POS	POS	POS	NEG	NEG	NEG	NEG	NEG	POS	POS	POS	NEG	POS	POS	NEG	NEG	POS	NEG	NEG
190994	CC0059	CC59-MSSA	NEG	NEG	NEG	NEG	NEG	NEG	NEG	NEG	POS	NEG	POS	POS	POS	POS	POS	POS	NEG	POS	POS	NEG	NEG	NEG	NEG	NEG
275172	CC0088	CC88-MSSA	NEG	NEG	NEG	NEG	NEG	NEG	NEG	NEG	POS	POS	POS	POS	POS	POS	POS	POS	NEG	POS	POS	NEG	NEG	POS	NEG	NEG
275173	CC0088	CC88-MSSA	NEG	NEG	NEG	NEG	NEG	NEG	NEG	NEG	POS	POS	POS	POS	POS	POS	POS	POS	NEG	POS	POS	NEG	NEG	POS	NEG	NEG
275174	CC0088	CC88-MSSA	NEG	NEG	NEG	NEG	NEG	NEG	NEG	NEG	POS	POS	POS	POS	POS	POS	POS	POS	NEG	POS	POS	NEG	NEG	POS	NEG	NEG
170971	CC0097	CC97-MSSA	NEG	NEG	NEG	NEG	POS	POS	POS	POS	NEG	NEG	NEG	NEG	NEG	POS	POS	POS	NEG	POS	POS	NEG	NEG	POS	NEG	NEG
112745	CC0097	CC97-MSSA	NEG	NEG	NEG	NEG	POS	POS	POS	POS	NEG	NEG	AMB	NEG	NEG	POS	POS	POS	NEG	POS	POS	NEG	NEG	POS	NEG	NEG
112748	CC0097	CC97-MSSA	NEG	NEG	NEG	NEG	POS	POS	POS	POS	NEG	NEG	AMB	NEG	NEG	POS	POS	POS	NEG	POS	POS	NEG	NEG	POS	NEG	NEG
112750	CC0097	CC97-MSSA	NEG	NEG	NEG	NEG	POS	POS	POS	POS	NEG	NEG	NEG	NEG	NEG	POS	POS	POS	NEG	POS	POS	NEG	NEG	POS	NEG	NEG
112762	CC0097	CC97-MSSA	NEG	NEG	NEG	NEG	POS	POS	POS	POS	NEG	NEG	AMB	NEG	NEG	POS	POS	POS	NEG	POS	POS	NEG	NEG	POS	NEG	NEG
112763	CC0097	CC97-MSSA	NEG	NEG	NEG	NEG	POS	POS	POS	POS	NEG	NEG	AMB	NEG	NEG	POS	POS	POS	NEG	POS	POS	NEG	NEG	POS	NEG	NEG
112764	CC0097	CC97-MSSA	NEG	NEG	NEG	NEG	POS	POS	POS	POS	NEG	NEG	AMB	NEG	NEG	POS	POS	POS	NEG	POS	POS	NEG	NEG	POS	NEG	NEG
170945	CC0097	CC97-MSSA	NEG	NEG	NEG	NEG	POS	POS	POS	POS	NEG	NEG	NEG	NEG	NEG	POS	POS	POS	NEG	POS	POS	NEG	NEG	POS	NEG	NEG
170954	CC0097	CC97-MSSA	NEG	NEG	NEG	NEG	POS	POS	POS	POS	NEG	NEG	NEG	NEG	NEG	POS	POS	POS	NEG	POS	POS	NEG	NEG	POS	NEG	NEG
170958	CC0097	CC97-MSSA	NEG	NEG	NEG	NEG	POS	POS	POS	POS	NEG	NEG	NEG	NEG	NEG	POS	POS	POS	NEG	POS	POS	NEG	NEG	POS	NEG	NEG
170963	CC0097	CC97-MSSA	NEG	NEG	NEG	NEG	POS	POS	POS	POS	NEG	NEG	NEG	NEG	NEG	POS	POS	POS	NEG	POS	POS	NEG	NEG	POS	NEG	NEG
170964	CC0097	CC97-MSSA	NEG	NEG	NEG	NEG	POS	POS	POS	POS	NEG	NEG	NEG	NEG	NEG	POS	POS	POS	NEG	POS	POS	NEG	NEG	POS	NEG	NEG
170965	CC0097	CC97-MSSA	NEG	NEG	NEG	NEG	POS	POS	POS	POS	NEG	NEG	NEG	NEG	NEG	POS	POS	POS	NEG	POS	POS	NEG	NEG	POS	NEG	NEG
170966	CC0097	CC97-MSSA	NEG	NEG	NEG	NEG	POS	POS	POS	POS	NEG	NEG	NEG	NEG	NEG	POS	POS	POS	NEG	POS	POS	NEG	NEG	POS	NEG	NEG
170974	CC0097	CC97-MSSA	NEG	NEG	NEG	NEG	POS	POS	POS	POS	NEG	NEG	NEG	NEG	NEG	POS	POS	POS	NEG	POS	POS	NEG	NEG	POS	NEG	NEG
170976	CC0097	CC97-MSSA	NEG	NEG	NEG	NEG	POS	POS	POS	POS	NEG	NEG	NEG	NEG	NEG	POS	POS	POS	NEG	POS	POS	NEG	NEG	POS	NEG	NEG
112744	CC0097	CC97-MSSA	NEG	NEG	NEG	NEG	POS	POS	POS	POS	NEG	NEG	AMB	NEG	NEG	POS	POS	POS	NEG	POS	POS	NEG	NEG	POS	NEG	NEG
112749	CC0097	CC97-MSSA	NEG	NEG	NEG	NEG	POS	POS	POS	POS	NEG	NEG	NEG	NEG	NEG	POS	POS	POS	NEG	POS	POS	NEG	NEG	POS	NEG	NEG
112757	CC0097	CC97-MSSA	NEG	NEG	NEG	NEG	POS	POS	POS	POS	NEG	NEG	AMB	NEG	NEG	POS	POS	POS	NEG	POS	POS	NEG	NEG	POS	NEG	AMB
170973	CC0097	CC97-MSSA	NEG	NEG	NEG	NEG	POS	POS	POS	POS	NEG	NEG	NEG	NEG	NEG	POS	POS	POS	NEG	POS	POS	NEG	NEG	POS	NEG	NEG
207756	CC0097	CC97-MSSA	NEG	NEG	NEG	NEG	POS	POS	POS	POS	NEG	NEG	NEG	NEG	NEG	POS	POS	POS	NEG	POS	POS	NEG	NEG	POS	NEG	AMB
206171	CC0097	CC97-MSSA	NEG	NEG	NEG	NEG	POS	POS	POS	POS	NEG	NEG	NEG	NEG	NEG	POS	POS	POS	NEG	POS	POS	NEG	NEG	POS	NEG	AMB
275169	CC0097	CC97-MSSA-[ccrAA]	NEG	NEG	NEG	NEG	POS	POS	POS	POS	NEG	NEG	NEG	NEG	NEG	POS	POS	POS	NEG	POS	POS	NEG	NEG	POS	NEG	NEG
275168	CC0097	CC97-MSSA-[ccrAA]	NEG	NEG	NEG	NEG	POS	POS	POS	POS	NEG	NEG	NEG	NEG	NEG	POS	POS	POS	NEG	POS	POS	NEG	NEG	POS	NEG	NEG
118320	CC0130	CC130-MSSA (lukF-P83/M+)	NEG	NEG	NEG	NEG	NEG	NEG	NEG	NEG	POS	POS	POS	POS	POS	POS	POS	POS	NEG	POS	POS	NEG	NEG	POS	NEG	NEG
203236	CC0130	CC130-MRSA-XI	NEG	NEG	NEG	AMB	NEG	NEG	NEG	NEG	POS	POS	POS	POS	POS	POS	POS	POS	NEG	POS	POS	NEG	NEG	POS	NEG	NEG
204204	CC0130	CC130-MRSA-XI	NEG	NEG	NEG	NEG	NEG	NEG	NEG	NEG	POS	POS	POS	POS	POS	POS	POS	POS	NEG	POS	POS	NEG	NEG	NEG	NEG	NEG
289122	CC0130	CC130-MRSA-XI	NEG	NEG	NEG	NEG	NEG	NEG	NEG	NEG	POS	POS	POS	POS	POS	POS	POS	POS	NEG	POS	POS	NEG	NEG	POS	NEG	NEG
225779	CC0130	CC130-MRSA-XI	NEG	NEG	NEG	NEG	NEG	NEG	NEG	NEG	POS	POS	POS	POS	POS	POS	POS	POS	NEG	POS	POS	NEG	NEG	POS	NEG	NEG
225780	CC0130	CC130-MRSA-XI	NEG	NEG	NEG	NEG	NEG	NEG	NEG	NEG	POS	POS	POS	POS	POS	POS	POS	POS	NEG	POS	POS	NEG	NEG	POS	NEG	NEG
170959	CC0130	CC130-MRSA-XI	NEG	NEG	NEG	NEG	NEG	NEG	NEG	NEG	POS	POS	POS	POS	POS	POS	POS	POS	NEG	POS	POS	NEG	NEG	NEG	NEG	NEG
179029	CC0130	CC130-MRSA-XI	NEG	NEG	NEG	NEG	NEG	NEG	NEG	NEG	POS	POS	POS	POS	POS	POS	POS	POS	NEG	POS	POS	NEG	NEG	POS	NEG	NEG
116254	CC0133	CC133-MSSA	NEG	NEG	NEG	NEG	NEG	NEG	NEG	NEG	POS	AMB	POS	POS	POS	POS	POS	POS	NEG	POS	POS	NEG	NEG	NEG	NEG	NEG
207761	CC0133	CC133-MSSA	NEG	NEG	NEG	NEG	NEG	NEG	NEG	NEG	POS	POS	POS	POS	POS	POS	POS	POS	NEG	POS	POS	NEG	NEG	NEG	NEG	POS
213103	CC0133	CC133-MSSA	NEG	NEG	NEG	AMB	NEG	NEG	POS	NEG	POS	POS	POS	POS	POS	POS	POS	POS	NEG	POS	POS	AMB	NEG	POS	NEG	AMB
275736	CC0133	CC133-MSSA	NEG	NEG	NEG	NEG	NEG	NEG	NEG	NEG	POS	POS	POS	POS	POS	POS	POS	POS	NEG	POS	POS	NEG	NEG	NEG	NEG	NEG
275737	CC0133	CC133-MSSA	NEG	NEG	NEG	NEG	NEG	NEG	NEG	NEG	POS	POS	POS	POS	POS	POS	POS	POS	NEG	POS	POS	NEG	NEG	NEG	NEG	NEG
275738	CC0133	CC133-MSSA	NEG	NEG	NEG	NEG	NEG	NEG	NEG	NEG	POS	POS	POS	POS	POS	POS	POS	POS	NEG	POS	POS	NEG	NEG	POS	NEG	NEG
275739	CC0133	CC133-MSSA	NEG	NEG	NEG	NEG	NEG	NEG	NEG	NEG	POS	POS	POS	POS	POS	POS	POS	POS	NEG	POS	POS	NEG	NEG	NEG	NEG	NEG
112755	CC0133	CC133-MSSA	NEG	NEG	NEG	NEG	NEG	NEG	NEG	NEG	POS	POS	POS	POS	POS	POS	POS	POS	NEG	POS	POS	NEG	NEG	NEG	NEG	NEG
225778	CC0398	CC398-MRSA-V/VT	NEG	NEG	NEG	NEG	POS	POS	POS	POS	NEG	NEG	NEG	NEG	NEG	POS	POS	POS	NEG	POS	POS	NEG	NEG	AMB	NEG	POS
123159	ST0425	ST425-MSSA	NEG	NEG	NEG	NEG	POS	POS	POS	POS	NEG	NEG	NEG	NEG	NEG	POS	POS	POS	NEG	POS	POS	NEG	NEG	NEG	NEG	NEG
86991	ST0425	ST425-MSSA	NEG	NEG	NEG	NEG	POS	POS	POS	POS	NEG	NEG	NEG	NEG	NEG	POS	POS	POS	NEG	POS	POS	NEG	NEG	NEG	NEG	NEG
112756	ST0425	ST425-MSSA	NEG	NEG	NEG	NEG	POS	POS	POS	POS	NEG	NEG	AMB	NEG	NEG	POS	POS	POS	NEG	POS	POS	NEG	NEG	NEG	NEG	NEG
195207	ST0425	ST425-MSSA	NEG	NEG	NEG	NEG	POS	POS	POS	POS	NEG	NEG	NEG	NEG	NEG	POS	POS	POS	NEG	POS	POS	NEG	NEG	POS	NEG	NEG
223609	ST0425	ST425-MSSA	NEG	NEG	NEG	NEG	POS	POS	POS	POS	NEG	NEG	NEG	NEG	NEG	POS	POS	POS	NEG	POS	POS	AMB	NEG	AMB	AMB	POS
275740	ST0425	ST425-MSSA	NEG	NEG	NEG	NEG	POS	POS	POS	POS	NEG	NEG	NEG	NEG	NEG	POS	POS	POS	NEG	POS	POS	NEG	NEG	POS	NEG	NEG
174889	ST0425	ST425-MSSA	NEG	NEG	NEG	NEG	POS	POS	POS	POS	NEG	NEG	NEG	NEG	NEG	POS	POS	POS	NEG	POS	POS	NEG	NEG	NEG	NEG	NEG
203230	ST0425	ST425-MSSA	AMB	NEG	AMB	AMB	POS	POS	POS	POS	NEG	NEG	NEG	NEG	NEG	POS	POS	POS	NEG	POS	POS	AMB	NEG	AMB	NEG	POS
203242	ST0425	ST425-MSSA	NEG	NEG	NEG	NEG	POS	POS	POS	POS	NEG	NEG	NEG	NEG	NEG	POS	POS	POS	NEG	POS	POS	AMB	NEG	AMB	NEG	POS
203243	ST0425	ST425-MSSA	NEG	NEG	AMB	NEG	POS	POS	POS	POS	NEG	NEG	NEG	NEG	NEG	POS	POS	POS	NEG	POS	POS	AMB	NEG	AMB	AMB	POS
207951	ST0425	ST425-MSSA	NEG	NEG	NEG	NEG	POS	POS	AMB	POS	NEG	NEG	NEG	NEG	NEG	POS	POS	POS	NEG	POS	POS	NEG	NEG	NEG	NEG	NEG
207981	ST0425	ST425-MSSA	NEG	NEG	NEG	NEG	POS	POS	POS	POS	NEG	NEG	NEG	NEG	NEG	POS	POS	POS	NEG	POS	POS	NEG	NEG	NEG	NEG	NEG
195212	ST0425	ST425-MSSA	NEG	NEG	NEG	NEG	POS	POS	POS	POS	NEG	NEG	NEG	NEG	NEG	POS	POS	POS	NEG	POS	POS	NEG	NEG	POS	NEG	AMB
195206	ST0425	ST425-MSSA	NEG	NEG	AMB	NEG	POS	POS	POS	POS	NEG	NEG	NEG	NEG	NEG	POS	POS	POS	NEG	POS	POS	AMB	NEG	POS	NEG	AMB
195202	ST0425	ST425-MSSA	NEG	NEG	NEG	NEG	POS	POS	POS	POS	NEG	NEG	NEG	NEG	NEG	POS	POS	POS	NEG	POS	POS	NEG	NEG	POS	NEG	NEG
216042	ST0425	ST425-MSSA	NEG	NEG	NEG	NEG	POS	POS	POS	POS	NEG	NEG	NEG	NEG	NEG	POS	POS	POS	NEG	POS	POS	AMB	NEG	POS	NEG	NEG
223608	ST0425	ST425-MSSA	NEG	NEG	NEG	NEG	POS	POS	POS	POS	NEG	NEG	NEG	NEG	NEG	POS	POS	POS	NEG	POS	POS	POS	NEG	AMB	AMB	AMB
223612	ST0425	ST425-MSSA	NEG	NEG	NEG	NEG	POS	POS	POS	POS	NEG	NEG	NEG	NEG	NEG	POS	POS	POS	NEG	POS	POS	AMB	NEG	POS	AMB	AMB
225733	ST0425	ST425-MSSA	NEG	NEG	NEG	NEG	POS	POS	POS	POS	NEG	NEG	NEG	NEG	NEG	POS	POS	POS	NEG	POS	POS	AMB	NEG	POS	NEG	AMB
230321	ST0425	ST425-MSSA	NEG	NEG	NEG	NEG	POS	POS	POS	POS	NEG	NEG	NEG	NEG	NEG	POS	POS	POS	NEG	POS	POS	NEG	NEG	POS	NEG	NEG
232368	ST0425	ST425-MSSA	NEG	NEG	NEG	NEG	POS	POS	POS	POS	NEG	NEG	NEG	NEG	NEG	POS	POS	POS	NEG	NEG	NEG	NEG	NEG	NEG	NEG	NEG
204215	ST0425	ST425-MSSA	NEG	NEG	NEG	NEG	POS	POS	POS	POS	NEG	NEG	NEG	NEG	NEG	POS	POS	POS	NEG	POS	POS	AMB	NEG	POS	NEG	NEG
204218	ST0425	ST425-MSSA	NEG	NEG	NEG	NEG	POS	POS	POS	POS	NEG	NEG	NEG	NEG	NEG	POS	POS	POS	NEG	POS	POS	AMB	NEG	POS	NEG	AMB
204222	ST0425	ST425-MSSA	NEG	NEG	NEG	NEG	POS	POS	POS	POS	NEG	NEG	NEG	NEG	NEG	POS	POS	POS	NEG	POS	POS	NEG	NEG	POS	NEG	AMB
204226	ST0425	ST425-MSSA	NEG	NEG	NEG	NEG	POS	POS	POS	POS	NEG	NEG	NEG	NEG	NEG	POS	POS	POS	NEG	POS	POS	NEG	NEG	POS	NEG	NEG
204906	ST0425	ST425-MSSA	NEG	NEG	NEG	NEG	POS	POS	POS	POS	NEG	NEG	NEG	NEG	NEG	POS	POS	POS	NEG	POS	POS	NEG	NEG	AMB	NEG	POS
204908	ST0425	ST425-MSSA	NEG	NEG	NEG	NEG	POS	POS	POS	POS	NEG	NEG	NEG	NEG	NEG	POS	POS	POS	NEG	POS	POS	AMB	NEG	AMB	NEG	POS
206170	ST0425	ST425-MSSA	NEG	NEG	NEG	NEG	POS	POS	POS	POS	NEG	NEG	NEG	NEG	NEG	POS	POS	POS	NEG	POS	POS	NEG	NEG	POS	NEG	NEG
207765	ST0425	ST425-MSSA	NEG	NEG	NEG	NEG	POS	POS	POS	POS	NEG	NEG	NEG	NEG	NEG	POS	POS	POS	NEG	POS	POS	NEG	NEG	POS	NEG	NEG
240048	CC599	CC599-MRSA-XI	NEG	NEG	NEG	NEG	POS	AMB	POS	POS	NEG	NEG	NEG	NEG	NEG	POS	POS	POS	NEG	AMB	POS	NEG	NEG	NEG	NEG	NEG
225730	CC0692	CC692-MSSA	NEG	NEG	NEG	NEG	POS	POS	POS	POS	NEG	NEG	NEG	NEG	NEG	POS	POS	POS	NEG	POS	POS	AMB	NEG	POS	NEG	AMB
112746	CC0692	CC692-MSSA	NEG	NEG	NEG	NEG	POS	POS	POS	POS	NEG	NEG	NEG	NEG	NEG	POS	POS	POS	NEG	POS	POS	POS	NEG	AMB	NEG	NEG
112754	CC0692	CC692-MSSA	NEG	NEG	NEG	NEG	POS	POS	POS	POS	NEG	NEG	NEG	NEG	NEG	POS	POS	POS	NEG	POS	POS	POS	NEG	AMB	NEG	NEG
112758	CC0692	CC692-MSSA	NEG	NEG	NEG	NEG	POS	POS	POS	POS	NEG	NEG	NEG	NEG	NEG	POS	POS	POS	NEG	POS	POS	POS	NEG	AMB	NEG	NEG
112759	CC0692	CC692-MSSA	NEG	NEG	NEG	NEG	POS	POS	POS	POS	NEG	NEG	AMB	NEG	NEG	POS	POS	POS	NEG	POS	POS	POS	NEG	AMB	NEG	NEG
124745	CC0692	CC692-MSSA	NEG	NEG	NEG	NEG	POS	POS	POS	POS	NEG	NEG	NEG	NEG	NEG	POS	POS	POS	NEG	POS	POS	POS	NEG	POS	NEG	NEG
170952	CC0692	CC692-MSSA	NEG	NEG	NEG	NEG	POS	POS	POS	POS	NEG	NEG	NEG	NEG	NEG	POS	POS	POS	NEG	POS	POS	POS	NEG	AMB	NEG	NEG
170953	CC0692	CC692-MSSA	NEG	NEG	NEG	NEG	POS	POS	POS	POS	NEG	NEG	NEG	NEG	NEG	POS	AMB	POS	NEG	POS	POS	POS	NEG	AMB	NEG	NEG
170970	CC0692	CC692-MSSA	NEG	NEG	NEG	NEG	POS	POS	POS	POS	NEG	NEG	NEG	NEG	NEG	POS	AMB	POS	NEG	POS	POS	POS	NEG	AMB	NEG	NEG
170950	CC0707	CC707-MSSA	NEG	NEG	NEG	NEG	POS	POS	POS	POS	NEG	NEG	NEG	NEG	NEG	POS	POS	POS	NEG	POS	POS	NEG	NEG	NEG	NEG	NEG
124948	ST0890	ST890-MSSA	NEG	NEG	NEG	NEG	NEG	NEG	NEG	NEG	POS	POS	POS	POS	POS	POS	POS	POS	NEG	POS	POS	NEG	NEG	NEG	NEG	NEG
220618	CC1956	CC1956-MSSA	NEG	NEG	NEG	NEG	POS	POS	POS	POS	NEG	NEG	NEG	NEG	NEG	POS	POS	POS	NEG	POS	POS	AMB	NEG	NEG	NEG	POS
112742	ST2425	ST2425-MSSA	NEG	NEG	NEG	NEG	NEG	NEG	NEG	NEG	POS	POS	POS	POS	POS	POS	POS	POS	NEG	POS	POS	NEG	NEG	AMB	NEG	NEG
170957	ST2425	ST2425-MSSA	NEG	NEG	NEG	NEG	NEG	NEG	NEG	NEG	POS	POS	POS	POS	POS	POS	POS	POS	NEG	POS	POS	NEG	NEG	NEG	NEG	NEG
170947	ST2691	ST2691-MSSA	NEG	NEG	NEG	NEG	NEG	NEG	NEG	NEG	POS	POS	POS	POS	POS	POS	POS	POS	NEG	NEG	NEG	NEG	NEG	NEG	NEG	NEG
170961	ST2691	ST2691-MSSA	NEG	NEG	NEG	NEG	NEG	NEG	NEG	NEG	POS	POS	POS	POS	POS	POS	POS	POS	NEG	NEG	NEG	NEG	NEG	NEG	NEG	NEG
112753	CC2767	ST2279-MSSA	NEG	NEG	NEG	NEG	NEG	NEG	NEG	NEG	POS	POS	POS	POS	POS	POS	POS	POS	NEG	POS	POS	NEG	NEG	AMB	NEG	NEG
112761	CC2767	ST2279-MSSA	NEG	NEG	NEG	NEG	NEG	NEG	NEG	NEG	POS	POS	POS	POS	POS	POS	POS	POS	NEG	POS	POS	NEG	NEG	NEG	NEG	NEG
228651	ST2963	ST2963-MSSA	NEG	NEG	NEG	NEG	NEG	NEG	NEG	NEG	POS	POS	POS	POS	POS	POS	POS	POS	NEG	POS	POS	NEG	POS	NEG	NEG	NEG
203244	ST3237	ST3237-MSSA	NEG	NEG	NEG	NEG	NEG	NEG	NEG	NEG	POS	POS	POS	POS	POS	POS	POS	POS	NEG	POS	POS	NEG	NEG	AMB	NEG	POS

ISOLATE ID	CLONAL COMPLEX (by array)	STRAIN AFFILIATION (by array)	ADHAESION FACTORS / GENES ENCODING MICROBIAL SURFACE COMPONENTS RECOGNIZING ADHESIVE MATRIX MOLECULES (MSCRAMM GENES)																			
			clumping factor A					clumping factor B					collagen-binding adhesin	cell wall associated fibronectin-binding protein	cell surface elastin binding protein					enolase	fibrinogen binding protein (19 kDa)	
			clfA	clfA (cons)	clfA (COL+RF122)	clfA (MRSA252)	clfA (Mu50+MW2)	clfB	clfB (cons)	clfB (COL+Mu50)	clfB (MW2)	clfB (RF122)	cna	ebh (cons)	ebpS	ebpS_probe 612	ebpS_probe 614	ebpS (01-1111)	ebpS (COL)	eno	fib	fib (MRSA252)
203228	CC0001	CC1-MSSA	POS	POS	NEG	NEG	POS	POS	POS	NEG	POS	AMB	POS	POS	POS	POS	POS	NEG	AMB	POS	POS	AMB
203229	CC0001	CC1-MSSA	POS	POS	NEG	NEG	POS	POS	POS	AMB	POS	AMB	POS	POS	POS	POS	POS	NEG	NEG	POS	POS	AMB
203233	CC0001	CC1-MSSA	POS	POS	NEG	NEG	POS	POS	POS	NEG	POS	AMB	POS	POS	POS	POS	POS	NEG	NEG	POS	POS	NEG
220334	CC0001	CC1-MSSA	POS	POS	NEG	NEG	POS	POS	POS	NEG	POS	AMB	POS	POS	POS	POS	POS	NEG	NEG	POS	POS	NEG
223621	CC0001	CC1-MSSA	POS	POS	NEG	NEG	POS	POS	POS	NEG	POS	AMB	POS	POS	POS	POS	POS	NEG	NEG	POS	POS	NEG
289123	CC0001	CC1-MRSA-IV, WA MRSA-1/57	POS	POS	NEG	NEG	POS	POS	POS	NEG	POS	AMB	POS	POS	POS	POS	POS	NEG	NEG	POS	POS	NEG
289124	CC0001	CC1-MRSA-IV, WA MRSA-1/57	POS	POS	NEG	NEG	POS	POS	POS	NEG	POS	AMB	POS	POS	POS	POS	POS	NEG	NEG	POS	POS	NEG
275170	CC0005	CC5-MSSA	POS	POS	NEG	NEG	POS	POS	POS	POS	NEG	NEG	NEG	POS	POS	POS	POS	NEG	NEG	POS	POS	NEG
275171	CC0005	CC5-MSSA	POS	POS	NEG	NEG	POS	POS	POS	POS	NEG	NEG	NEG	POS	POS	AMB	POS	NEG	NEG	POS	POS	NEG
112747	CC0005	CC5-MSSA	POS	POS	NEG	NEG	POS	POS	POS	POS	NEG	NEG	NEG	POS	POS	POS	POS	NEG	NEG	POS	POS	NEG
112752	CC0006	CC6-MSSA	POS	POS	NEG	NEG	POS	POS	POS	NEG	NEG	POS	POS	POS	POS	POS	POS	NEG	NEG	POS	POS	NEG
275178	CC0007	CC7-MSSA	POS	POS	POS	AMB	AMB	POS	POS	NEG	NEG	NEG	NEG	POS	POS	AMB	POS	NEG	POS	POS	POS	NEG
275166	CC0008	CC8-MSSA	POS	POS	POS	AMB	AMB	POS	POS	POS	NEG	NEG	NEG	POS	POS	POS	POS	NEG	POS	POS	POS	NEG
275176	CC0008	CC8-MSSA	POS	POS	POS	AMB	AMB	POS	POS	POS	NEG	NEG	NEG	POS	POS	POS	POS	NEG	POS	POS	POS	NEG
275177	CC0008	CC8-MSSA	POS	POS	POS	AMB	AMB	POS	POS	POS	NEG	NEG	NEG	POS	POS	POS	POS	NEG	POS	POS	POS	NEG
275167	CC0009	CC9-MSSA	POS	POS	NEG	NEG	POS	POS	POS	NEG	NEG	NEG	NEG	POS	POS	NEG	POS	NEG	NEG	POS	POS	NEG
170968	CC0012	CC12-MSSA	POS	POS	POS	AMB	AMB	POS	POS	NEG	NEG	NEG	POS	POS	POS	POS	POS	NEG	NEG	POS	POS	NEG
275175	CC0015	CC15-MSSA	POS	POS	NEG	NEG	POS	POS	POS	NEG	POS	AMB	NEG	POS	POS	POS	POS	NEG	POS	POS	POS	NEG
170949	CC0015	CC15-MSSA	POS	POS	NEG	NEG	POS	POS	POS	NEG	POS	AMB	NEG	POS	POS	POS	POS	NEG	POS	POS	POS	NEG
228658	CC0022	CC22-MSSA	POS	POS	NEG	NEG	POS	POS	POS	NEG	NEG	POS	POS	NEG	POS	POS	POS	NEG	POS	POS	NEG	POS
289125	CC0022	CC22-MRSA-IV (PVL+)	POS	POS	NEG	NEG	POS	POS	POS	NEG	NEG	POS	POS	NEG	POS	POS	POS	NEG	POS	POS	NEG	POS
289126	CC0022	CC22-MRSA-IV (PVL+)	POS	POS	NEG	NEG	POS	POS	POS	NEG	NEG	POS	POS	NEG	POS	POS	POS	NEG	POS	POS	NEG	POS
289127	CC0022	CC22-MRSA-IV (PVL+)	POS	POS	NEG	NEG	POS	POS	POS	NEG	NEG	POS	POS	NEG	POS	POS	POS	NEG	POS	POS	NEG	POS
131939	CC0025	CC25-MSSA	POS	POS	NEG	NEG	POS	POS	POS	NEG	NEG	POS	NEG	POS	POS	POS	POS	NEG	POS	POS	POS	NEG
275165	CC0030	CC30-MSSA (lukF-P83/lukM+)	POS	POS	AMB	POS	NEG	POS	POS	NEG	NEG	NEG	POS	POS	POS	POS	POS	NEG	NEG	POS	NEG	POS
275741	CC0030	CC30-MSSA	POS	POS	AMB	POS	NEG	POS	POS	NEG	NEG	NEG	POS	POS	POS	POS	POS	NEG	NEG	POS	NEG	POS
124492	CC0049	CC49-MSSA (lukF-P83/lukM+)	POS	POS	NEG	NEG	POS	POS	POS	NEG	POS	AMB	NEG	POS	POS	POS	POS	NEG	NEG	POS	POS	NEG
220616	CC0049	CC49-MSSA (lukF-P83/lukM+)	POS	POS	NEG	NEG	POS	POS	POS	NEG	POS	AMB	NEG	POS	POS	POS	POS	NEG	NEG	POS	POS	NEG
228649	CC0049	CC49-MSSA (lukF-P83/lukM+)	POS	POS	NEG	NEG	POS	POS	POS	NEG	POS	AMB	NEG	POS	POS	POS	POS	NEG	NEG	POS	POS	NEG
190994	CC0059	CC59-MSSA	POS	POS	POS	NEG	AMB	POS	POS	NEG	NEG	POS	NEG	POS	AMB	NEG	AMB	NEG	NEG	POS	POS	NEG
275172	CC0088	CC88-MSSA	POS	POS	NEG	NEG	POS	POS	POS	NEG	NEG	NEG	NEG	POS	POS	NEG	POS	NEG	POS	POS	POS	NEG
275173	CC0088	CC88-MSSA	POS	POS	NEG	NEG	POS	POS	POS	NEG	NEG	NEG	NEG	POS	POS	AMB	POS	NEG	POS	POS	POS	NEG
275174	CC0088	CC88-MSSA	POS	POS	NEG	NEG	POS	POS	POS	NEG	NEG	NEG	NEG	POS	POS	POS	POS	NEG	POS	POS	POS	NEG
170971	CC0097	CC97-MSSA	POS	POS	POS	AMB	AMB	POS	POS	NEG	NEG	NEG	NEG	POS	POS	POS	POS	NEG	NEG	POS	POS	NEG
112745	CC0097	CC97-MSSA	POS	POS	POS	AMB	AMB	POS	POS	NEG	NEG	POS	NEG	POS	POS	POS	POS	NEG	NEG	POS	POS	NEG
112748	CC0097	CC97-MSSA	POS	POS	POS	AMB	AMB	POS	POS	NEG	NEG	POS	NEG	POS	POS	POS	POS	NEG	NEG	POS	POS	NEG
112750	CC0097	CC97-MSSA	POS	POS	POS	AMB	AMB	POS	POS	NEG	NEG	NEG	NEG	POS	POS	POS	POS	NEG	NEG	POS	POS	NEG
112762	CC0097	CC97-MSSA	POS	POS	POS	AMB	AMB	POS	POS	NEG	NEG	POS	NEG	POS	POS	POS	POS	NEG	NEG	POS	POS	NEG
112763	CC0097	CC97-MSSA	POS	POS	POS	AMB	AMB	POS	POS	NEG	NEG	POS	NEG	POS	POS	POS	POS	NEG	NEG	POS	POS	NEG
112764	CC0097	CC97-MSSA	POS	POS	POS	AMB	AMB	POS	POS	NEG	NEG	POS	NEG	POS	POS	POS	POS	NEG	NEG	POS	POS	NEG
170945	CC0097	CC97-MSSA	POS	POS	POS	AMB	AMB	POS	POS	NEG	NEG	NEG	NEG	POS	POS	POS	POS	NEG	NEG	POS	POS	NEG
170954	CC0097	CC97-MSSA	POS	POS	POS	AMB	AMB	POS	POS	NEG	NEG	NEG	NEG	POS	POS	POS	POS	NEG	NEG	POS	POS	NEG
170958	CC0097	CC97-MSSA	POS	POS	POS	AMB	AMB	POS	POS	NEG	NEG	NEG	NEG	POS	POS	POS	POS	NEG	NEG	POS	POS	NEG
170963	CC0097	CC97-MSSA	POS	POS	POS	AMB	AMB	POS	POS	NEG	NEG	NEG	NEG	POS	POS	POS	POS	NEG	NEG	POS	POS	NEG
170964	CC0097	CC97-MSSA	POS	POS	POS	AMB	AMB	POS	POS	NEG	NEG	NEG	NEG	POS	POS	POS	POS	NEG	NEG	POS	POS	NEG
170965	CC0097	CC97-MSSA	POS	POS	POS	AMB	AMB	POS	POS	NEG	NEG	NEG	NEG	POS	POS	POS	POS	NEG	NEG	POS	POS	NEG
170966	CC0097	CC97-MSSA	POS	POS	POS	AMB	AMB	POS	POS	NEG	NEG	NEG	NEG	POS	POS	POS	POS	NEG	NEG	POS	POS	NEG
170974	CC0097	CC97-MSSA	POS	POS	POS	AMB	AMB	POS	POS	NEG	NEG	NEG	NEG	POS	POS	POS	POS	NEG	NEG	POS	POS	NEG
170976	CC0097	CC97-MSSA	POS	POS	POS	AMB	AMB	POS	POS	NEG	NEG	NEG	NEG	POS	POS	POS	POS	NEG	NEG	POS	POS	NEG
112744	CC0097	CC97-MSSA	POS	POS	POS	AMB	AMB	POS	POS	NEG	NEG	POS	NEG	POS	POS	POS	POS	NEG	NEG	POS	POS	NEG
112749	CC0097	CC97-MSSA	POS	POS	POS	AMB	AMB	POS	POS	NEG	NEG	POS	NEG	POS	POS	POS	POS	NEG	NEG	POS	POS	NEG
112757	CC0097	CC97-MSSA	POS	POS	POS	AMB	AMB	POS	POS	NEG	NEG	POS	NEG	POS	POS	POS	POS	NEG	NEG	POS	POS	NEG
170973	CC0097	CC97-MSSA	POS	POS	POS	AMB	AMB	POS	POS	NEG	NEG	NEG	NEG	POS	POS	POS	POS	NEG	NEG	POS	POS	NEG
207756	CC0097	CC97-MSSA	POS	POS	POS	AMB	AMB	POS	POS	NEG	NEG	POS	NEG	POS	POS	POS	POS	NEG	NEG	POS	POS	NEG
206171	CC0097	CC97-MSSA	POS	POS	AMB	AMB	POS	POS	POS	NEG	NEG	NEG	NEG	POS	POS	POS	POS	NEG	NEG	POS	POS	NEG
275169	CC0097	CC97-MSSA-[ccrAA]	POS	POS	POS	AMB	AMB	POS	POS	NEG	NEG	NEG	NEG	POS	POS	POS	POS	NEG	NEG	POS	POS	NEG
275168	CC0097	CC97-MSSA-[ccrAA]	POS	POS	POS	AMB	AMB	POS	POS	NEG	NEG	NEG	NEG	POS	POS	POS	POS	NEG	NEG	POS	POS	NEG
118320	CC0130	CC130-MSSA (lukF-P83/M+)	POS	POS	POS	AMB	POS	POS	POS	NEG	POS	POS	NEG	POS	POS	POS	POS	NEG	NEG	POS	POS	NEG
203236	CC0130	CC130-MRSA-XI	POS	POS	POS	AMB	AMB	POS	POS	NEG	POS	AMB	NEG	POS	POS	POS	POS	NEG	AMB	POS	POS	NEG
204204	CC0130	CC130-MRSA-XI	POS	POS	POS	AMB	AMB	POS	POS	NEG	AMB	POS	NEG	POS	POS	POS	POS	NEG	NEG	POS	POS	NEG
289122	CC0130	CC130-MRSA-XI	POS	POS	POS	AMB	AMB	POS	POS	NEG	POS	AMB	NEG	POS	POS	POS	POS	NEG	NEG	POS	POS	NEG
225779	CC0130	CC130-MRSA-XI	POS	POS	POS	AMB	AMB	POS	POS	NEG	POS	AMB	NEG	POS	POS	POS	POS	NEG	NEG	POS	POS	NEG
225780	CC0130	CC130-MRSA-XI	POS	POS	POS	AMB	AMB	POS	POS	NEG	POS	AMB	NEG	POS	POS	POS	POS	NEG	NEG	POS	POS	NEG
170959	CC0130	CC130-MRSA-XI	POS	POS	POS	NEG	AMB	POS	POS	NEG	AMB	POS	NEG	POS	POS	POS	POS	NEG	NEG	POS	POS	NEG
179029	CC0130	CC130-MRSA-XI	POS	POS	POS	AMB	AMB	POS	POS	NEG	AMB	POS	AMB	POS	POS	POS	POS	NEG	NEG	POS	POS	NEG
116254	CC0133	CC133-MSSA	POS	POS	NEG	NEG	POS	POS	POS	NEG	NEG	NEG	NEG	POS	POS	NEG	POS	POS	NEG	POS	POS	NEG
207761	CC0133	CC133-MSSA	POS	POS	NEG	NEG	POS	POS	POS	NEG	NEG	NEG	NEG	POS	POS	NEG	POS	POS	NEG	POS	POS	NEG
213103	CC0133	CC133-MSSA	POS	POS	NEG	NEG	POS	POS	POS	NEG	NEG	POS	NEG	POS	POS	NEG	POS	POS	AMB	POS	POS	NEG
275736	CC0133	CC133-MSSA	POS	POS	NEG	NEG	POS	POS	POS	NEG	NEG	NEG	NEG	POS	POS	NEG	POS	POS	NEG	POS	POS	NEG
275737	CC0133	CC133-MSSA	POS	POS	NEG	NEG	POS	POS	POS	NEG	NEG	NEG	NEG	POS	POS	NEG	POS	POS	NEG	POS	POS	NEG
275738	CC0133	CC133-MSSA	POS	POS	NEG	NEG	POS	POS	POS	NEG	NEG	NEG	NEG	POS	POS	NEG	POS	POS	NEG	POS	POS	NEG
275739	CC0133	CC133-MSSA	POS	POS	NEG	NEG	POS	POS	POS	NEG	NEG	NEG	NEG	POS	POS	NEG	POS	POS	NEG	POS	POS	NEG
112755	CC0133	CC133-MSSA	POS	POS	NEG	NEG	POS	POS	POS	NEG	NEG	AMB	POS	POS	POS	NEG	POS	POS	NEG	POS	POS	NEG
225778	CC0398	CC398-MRSA-V/VT	POS	POS	POS	AMB	AMB	POS	POS	NEG	POS	AMB	POS	POS	POS	NEG	POS	POS	POS	POS	NEG	POS
123159	ST0425	ST425-MSSA	POS	POS	NEG	NEG	POS	POS	POS	NEG	NEG	NEG	NEG	POS	POS	NEG	POS	POS	NEG	POS	POS	NEG
86991	ST0425	ST425-MSSA	POS	POS	NEG	NEG	POS	POS	POS	NEG	NEG	NEG	NEG	POS	POS	NEG	POS	POS	NEG	POS	POS	NEG
112756	ST0425	ST425-MSSA	POS	POS	NEG	NEG	POS	POS	POS	NEG	NEG	POS	NEG	POS	POS	NEG	POS	POS	NEG	POS	POS	NEG
195207	ST0425	ST425-MSSA	POS	POS	NEG	NEG	POS	POS	POS	NEG	NEG	POS	NEG	POS	POS	NEG	POS	POS	NEG	POS	POS	NEG
223609	ST0425	ST425-MSSA	POS	POS	NEG	NEG	POS	POS	POS	NEG	NEG	POS	NEG	POS	POS	NEG	POS	POS	NEG	POS	POS	NEG
275740	ST0425	ST425-MSSA	POS	POS	NEG	NEG	POS	POS	POS	NEG	NEG	POS	NEG	POS	POS	NEG	POS	POS	NEG	POS	POS	NEG
174889	ST0425	ST425-MSSA	POS	POS	NEG	NEG	POS	POS	POS	NEG	NEG	AMB	NEG	POS	POS	NEG	POS	POS	NEG	POS	POS	NEG
203230	ST0425	ST425-MSSA	POS	POS	NEG	NEG	POS	POS	POS	NEG	NEG	POS	AMB	POS	POS	NEG	POS	POS	NEG	POS	POS	AMB
203242	ST0425	ST425-MSSA	POS	POS	NEG	NEG	POS	POS	POS	NEG	NEG	POS	NEG	POS	POS	NEG	POS	POS	NEG	POS	POS	NEG
203243	ST0425	ST425-MSSA	POS	POS	NEG	NEG	POS	POS	POS	AMB	NEG	POS	AMB	POS	POS	NEG	POS	POS	NEG	POS	POS	AMB
207951	ST0425	ST425-MSSA	POS	POS	NEG	NEG	POS	POS	POS	NEG	NEG	NEG	NEG	POS	POS	NEG	POS	POS	NEG	POS	POS	NEG
207981	ST0425	ST425-MSSA	POS	POS	NEG	NEG	POS	POS	POS	NEG	NEG	NEG	NEG	POS	POS	NEG	POS	POS	NEG	POS	POS	NEG
195212	ST0425	ST425-MSSA	POS	POS	NEG	NEG	POS	POS	POS	NEG	NEG	POS	NEG	POS	POS	NEG	POS	POS	NEG	POS	POS	NEG
195206	ST0425	ST425-MSSA	POS	POS	NEG	NEG	POS	POS	POS	NEG	NEG	POS	AMB	POS	POS	NEG	POS	POS	NEG	POS	POS	NEG
195202	ST0425	ST425-MSSA	POS	POS	NEG	NEG	POS	POS	POS	NEG	NEG	POS	NEG	POS	POS	NEG	POS	POS	NEG	POS	POS	NEG
216042	ST0425	ST425-MSSA	POS	POS	NEG	NEG	POS	POS	POS	NEG	NEG	POS	AMB	POS	POS	NEG	POS	POS	NEG	POS	POS	NEG
223608	ST0425	ST425-MSSA	POS	POS	NEG	NEG	POS	POS	POS	NEG	NEG	POS	AMB	POS	POS	NEG	POS	POS	NEG	POS	POS	NEG
223612	ST0425	ST425-MSSA	POS	POS	NEG	NEG	POS	POS	POS	NEG	NEG	POS	NEG	POS	POS	NEG	POS	POS	NEG	POS	POS	NEG
225733	ST0425	ST425-MSSA	POS	POS	NEG	NEG	POS	POS	POS	NEG	NEG	POS	NEG	POS	POS	NEG	POS	POS	NEG	POS	POS	NEG
230321	ST0425	ST425-MSSA	POS	POS	NEG	NEG	POS	POS	POS	NEG	NEG	POS	NEG	POS	POS	NEG	POS	POS	NEG	POS	POS	NEG
232368	ST0425	ST425-MSSA	POS	POS	NEG	NEG	POS	POS	POS	NEG	NEG	POS	NEG	POS	POS	NEG	POS	POS	NEG	POS	POS	NEG
204215	ST0425	ST425-MSSA	POS	POS	NEG	NEG	POS	POS	POS	NEG	NEG	POS	NEG	POS	POS	NEG	POS	POS	NEG	POS	POS	NEG
204218	ST0425	ST425-MSSA	POS	POS	NEG	NEG	POS	POS	POS	NEG	NEG	POS	NEG	POS	POS	NEG	POS	POS	NEG	POS	POS	NEG
204222	ST0425	ST425-MSSA	POS	POS	NEG	NEG	POS	POS	POS	NEG	NEG	POS	NEG	POS	POS	NEG	POS	POS	NEG	POS	POS	NEG
204226	ST0425	ST425-MSSA	POS	POS	NEG	NEG	POS	POS	POS	NEG	NEG	POS	NEG	POS	POS	NEG	POS	POS	NEG	POS	POS	NEG
204906	ST0425	ST425-MSSA	POS	POS	NEG	NEG	POS	POS	POS	NEG	NEG	POS	NEG	POS	POS	NEG	POS	POS	NEG	POS	POS	NEG
204908	ST0425	ST425-MSSA	POS	POS	NEG	NEG	POS	POS	POS	NEG	NEG	POS	NEG	POS	POS	NEG	POS	POS	NEG	POS	POS	NEG
206170	ST0425	ST425-MSSA	POS	POS	NEG	NEG	POS	POS	POS	NEG	NEG	NEG	NEG	POS	POS	NEG	POS	POS	NEG	POS	POS	NEG
207765	ST0425	ST425-MSSA	POS	POS	NEG	NEG	POS	POS	POS	NEG	NEG	NEG	NEG	POS	POS	NEG	POS	POS	NEG	POS	POS	NEG
240048	CC599	CC599-MRSA-XI	AMB	POS	POS	AMB	AMB	AMB	POS	NEG	NEG	NEG	NEG	POS	AMB	NEG	POS	POS	NEG	POS	POS	NEG
225730	CC0692	CC692-MSSA	POS	POS	NEG	NEG	POS	POS	POS	NEG	NEG	POS	NEG	POS	POS	NEG	POS	POS	NEG	POS	POS	NEG
112746	CC0692	CC692-MSSA	POS	POS	NEG	NEG	POS	POS	POS	NEG	NEG	NEG	NEG	POS	POS	NEG	POS	POS	NEG	POS	POS	NEG
112754	CC0692	CC692-MSSA	POS	POS	NEG	NEG	POS	POS	POS	NEG	NEG	AMB	NEG	POS	POS	NEG	POS	AMB	NEG	POS	POS	NEG
112758	CC0692	CC692-MSSA	POS	POS	NEG	NEG	POS	POS	POS	NEG	NEG	POS	NEG	POS	POS	NEG	POS	POS	NEG	POS	POS	NEG
112759	CC0692	CC692-MSSA	POS	POS	NEG	NEG	POS	POS	POS	NEG	NEG	POS	NEG	POS	POS	NEG	POS	POS	NEG	POS	POS	NEG
124745	CC0692	CC692-MSSA	POS	POS	NEG	NEG	POS	POS	POS	NEG	NEG	AMB	NEG	POS	POS	NEG	POS	POS	NEG	POS	POS	NEG
170952	CC0692	CC692-MSSA	POS	POS	NEG	NEG	POS	POS	POS	NEG	NEG	POS	NEG	POS	POS	NEG	POS	POS	NEG	POS	POS	NEG
170953	CC0692	CC692-MSSA	POS	POS	NEG	NEG	POS	POS	POS	NEG	NEG	NEG	NEG	POS	POS	NEG	AMB	POS	NEG	POS	POS	NEG
170970	CC0692	CC692-MSSA	POS	POS	NEG	NEG	POS	POS	POS	NEG	NEG	NEG	NEG	POS	POS	NEG	AMB	POS	NEG	POS	POS	NEG
170950	CC0707	CC707-MSSA	POS	POS	POS	AMB	AMB	POS	POS	NEG	NEG	NEG	POS	POS	POS	NEG	POS	POS	NEG	POS	POS	NEG
124948	ST0890	ST890-MSSA	POS	POS	NEG	NEG	POS	POS	POS	NEG	NEG	NEG	POS	POS	POS	POS	POS	NEG	NEG	POS	POS	NEG
220618	CC1956	CC1956-MSSA	POS	POS	NEG	NEG	POS	POS	POS	NEG	NEG	POS	POS	POS	POS	POS	POS	NEG	NEG	POS	POS	NEG
112742	ST2425	ST2425-MSSA	POS	POS	POS	AMB	POS	POS	POS	NEG	NEG	POS	POS	POS	POS	POS	POS	NEG	NEG	POS	POS	NEG
170957	ST2425	ST2425-MSSA	POS	POS	POS	AMB	AMB	POS	POS	NEG	NEG	NEG	POS	POS	POS	POS	POS	NEG	NEG	POS	POS	NEG
170947	ST2691	ST2691-MSSA	POS	POS	NEG	NEG	POS	POS	POS	NEG	NEG	NEG	NEG	POS	POS	POS	POS	NEG	NEG	POS	POS	NEG
170961	ST2691	ST2691-MSSA	POS	POS	NEG	NEG	POS	POS	POS	NEG	NEG	AMB	NEG	POS	POS	POS	POS	NEG	NEG	POS	POS	NEG
112753	CC2767	ST2279-MSSA	POS	POS	POS	AMB	AMB	POS	POS	NEG	NEG	AMB	POS	POS	POS	NEG	POS	POS	NEG	POS	POS	NEG
112761	CC2767	ST2279-MSSA	POS	POS	POS	AMB	AMB	POS	POS	NEG	NEG	NEG	POS	POS	POS	NEG	POS	POS	NEG	POS	POS	NEG
228651	ST2963	ST2963-MSSA	POS	POS	NEG	NEG	POS	POS	POS	NEG	POS	AMB	POS	POS	POS	POS	POS	NEG	NEG	POS	POS	NEG
203244	ST3237	ST3237-MSSA	POS	POS	POS	NEG	AMB	POS	POS	NEG	AMB	POS	NEG	POS	POS	NEG	POS	POS	NEG	POS	POS	NEG

ADHAESION FACTORS / GENES ENCODING MICROBIAL SURFACE COMPONENTS RECOGNIZING ADHESIVE MATRIX MOLECULES (MSCRAMM GENES)

ISOLATE ID	CLONAL COMPLEX (by array)	STRAIN AFFILIATION (by array)	fibronectin-binding protein A						fibronectin-binding protein B							Major histocompatibility complex class II analog protein (=Extracellular adherence protein, eap)				Staphylococcus aureus surface protein G				Ser-Asp rich fibrinogen-/bone sialoprotein-binding protein C						
			fnbA	fnbA (cons)	fnbA (COL)	fnbA (MRSA252)	fnbA (Mu50+MW2)	fnbA (RF122)	fnbB	fnbB (COL)	fnbB (COL+Mu50+MW2)	fnbB (Mu50)	fnbB (MW2)	fnbB (ST15)	fnbB (ST45-2)	map	map (COL)	map (MRSA252)	map (Mu50+MW2)	sasG	sasG (COL+Mu50)	sasG (MW2)	sasG (OtherThan252+122)	sdrC	sdrC (cons)	sdrC (B1)	sdrC (COL)	sdrC (Mu50)	sdrC (MW2+MRSA252+RF122)	sdrC (OtherThan252+RF122)
203228	CC0001	CC1-MSSA	POS	POS	NEG	NEG	POS	NEG	POS	NEG	AMB	AMB	POS	NEG	NEG	POS	POS	AMB	POS	POS	NEG	POS	POS	POS	POS	NEG	NEG	NEG	POS	POS
203229	CC0001	CC1-MSSA	POS	POS	NEG	NEG	POS	NEG	POS	NEG	AMB	NEG	POS	NEG	NEG	POS	POS	AMB	POS	POS	NEG	POS	POS	POS	POS	NEG	NEG	NEG	POS	POS
203233	CC0001	CC1-MSSA	POS	POS	NEG	NEG	POS	NEG	POS	NEG	AMB	NEG	POS	NEG	NEG	POS	AMB	NEG	POS	POS	NEG	POS	POS	POS	POS	NEG	NEG	NEG	POS	POS
220334	CC0001	CC1-MSSA	POS	POS	NEG	NEG	POS	NEG	POS	NEG	AMB	NEG	POS	NEG	NEG	POS	NEG	NEG	POS	POS	NEG	POS	POS	POS	POS	NEG	NEG	NEG	POS	POS
223621	CC0001	CC1-MSSA	POS	POS	NEG	NEG	POS	NEG	POS	NEG	AMB	NEG	POS	NEG	NEG	NEG	NEG	NEG	NEG	POS	NEG	POS	POS	POS	POS	NEG	NEG	NEG	POS	POS
289123	CC0001	CC1-MRSA-IV, WA MRSA-1/57	POS	POS	NEG	NEG	POS	NEG	POS	NEG	AMB	NEG	POS	NEG	NEG	NEG	NEG	NEG	NEG	POS	NEG	POS	POS	POS	POS	NEG	NEG	NEG	POS	POS
289124	CC0001	CC1-MRSA-IV, WA MRSA-1/57	POS	POS	NEG	NEG	POS	NEG	POS	NEG	AMB	NEG	POS	NEG	NEG	NEG	NEG	NEG	NEG	POS	NEG	POS	POS	POS	POS	NEG	NEG	NEG	POS	POS
275170	CC0005	CC5-MSSA	POS	POS	NEG	NEG	POS	NEG	POS	NEG	AMB	POS	NEG	NEG	NEG	POS	POS	NEG	POS	POS	POS	NEG	POS	POS	POS	NEG	NEG	POS	NEG	POS
275171	CC0005	CC5-MSSA	POS	POS	NEG	NEG	POS	NEG	POS	NEG	POS	AMB	NEG	NEG	NEG	POS	NEG	NEG	POS	POS	POS	NEG	POS	POS	POS	NEG	NEG	POS	NEG	POS
112747	CC0005	CC5-MSSA	POS	POS	NEG	NEG	POS	NEG	POS	NEG	POS	AMB	NEG	NEG	NEG	POS	POS	NEG	POS	POS	POS	NEG	POS	POS	POS	NEG	NEG	POS	NEG	POS
112752	CC0006	CC6-MSSA	POS	POS	NEG	POS	NEG	NEG	POS	NEG	AMB	POS	NEG	NEG	NEG	POS	AMB	NEG	POS	POS	NEG	POS	POS	POS	POS	NEG	POS	NEG	NEG	POS
275178	CC0007	CC7-MSSA	POS	POS	NEG	NEG	NEG	NEG	POS	NEG	AMB	NEG	NEG	NEG	POS	POS	NEG	NEG	POS	NEG	NEG	NEG	NEG	POS	POS	NEG	POS	NEG	NEG	POS
275166	CC0008	CC8-MSSA	POS	POS	POS	NEG	NEG	NEG	POS	POS	AMB	NEG	NEG	NEG	NEG	POS	POS	NEG	NEG	POS	POS	NEG	POS	POS	POS	NEG	POS	NEG	NEG	POS
275176	CC0008	CC8-MSSA	POS	POS	POS	NEG	NEG	NEG	POS	POS	AMB	NEG	NEG	NEG	NEG	POS	POS	NEG	NEG	POS	POS	NEG	POS	POS	POS	NEG	POS	NEG	NEG	POS
275177	CC0008	CC8-MSSA	POS	POS	POS	NEG	NEG	NEG	POS	POS	AMB	NEG	NEG	NEG	NEG	POS	POS	NEG	NEG	POS	POS	NEG	POS	POS	POS	NEG	POS	NEG	NEG	POS
275167	CC0009	CC9-MSSA	POS	POS	NEG	POS	NEG	NEG	POS	NEG	POS	AMB	NEG	NEG	NEG	POS	POS	NEG	POS	NEG	NEG	NEG	NEG	POS	POS	NEG	NEG	POS	NEG	POS
170968	CC0012	CC12-MSSA	POS	POS	NEG	NEG	NEG	NEG	POS	NEG	AMB	NEG	NEG	NEG	POS	POS	POS	NEG	POS	NEG	NEG	NEG	NEG	POS	POS	NEG	POS	NEG	NEG	POS
275175	CC0015	CC15-MSSA	POS	POS	POS	NEG	NEG	NEG	POS	NEG	POS	AMB	NEG	AMB	NEG	POS	POS	NEG	POS	POS	NEG	POS	POS	POS	POS	NEG	POS	NEG	NEG	POS
170949	CC0015	CC15-MSSA	POS	POS	POS	NEG	NEG	NEG	POS	NEG	POS	AMB	NEG	AMB	NEG	POS	AMB	NEG	POS	POS	NEG	POS	POS	POS	POS	NEG	POS	NEG	NEG	POS
228658	CC0022	CC22-MSSA	POS	POS	NEG	NEG	POS	NEG	POS	NEG	AMB	NEG	POS	NEG	NEG	POS	NEG	NEG	POS	POS	NEG	POS	POS	POS	POS	NEG	NEG	POS	NEG	POS
289125	CC0022	CC22-MRSA-IV (PVL+)	POS	POS	NEG	NEG	POS	NEG	POS	NEG	AMB	NEG	POS	NEG	NEG	POS	NEG	NEG	POS	POS	NEG	POS	POS	POS	POS	NEG	NEG	POS	NEG	POS
289126	CC0022	CC22-MRSA-IV (PVL+)	POS	POS	NEG	NEG	POS	NEG	POS	NEG	AMB	NEG	POS	NEG	NEG	POS	NEG	NEG	POS	POS	NEG	POS	POS	POS	POS	NEG	NEG	POS	NEG	POS
289127	CC0022	CC22-MRSA-IV (PVL+)	POS	POS	NEG	NEG	POS	NEG	POS	NEG	AMB	NEG	POS	NEG	NEG	POS	NEG	NEG	POS	POS	NEG	POS	POS	POS	POS	NEG	NEG	POS	NEG	POS
131939	CC0025	CC25-MSSA	POS	POS	NEG	NEG	POS	NEG	POS	NEG	AMB	POS	NEG	NEG	NEG	POS	POS	NEG	POS	NEG	NEG	NEG	NEG	POS	POS	NEG	NEG	NEG	POS	POS
275165	CC0030	CC30-MSSA (lukF-P83/lukM+)	POS	POS	NEG	POS	NEG	NEG	POS	NEG	AMB	POS	NEG	NEG	NEG	POS	NEG	POS	NEG	NEG	NEG	NEG	NEG	POS	POS	NEG	NEG	NEG	NEG	NEG
275741	CC0030	CC30-MSSA	POS	POS	NEG	POS	NEG	NEG	NEG	NEG	NEG	NEG	NEG	NEG	NEG	POS	NEG	POS	NEG	NEG	NEG	NEG	NEG	POS	POS	NEG	NEG	NEG	NEG	NEG
124492	CC0049	CC49-MSSA (lukF-P83/lukM+)	POS	POS	NEG	POS	NEG	NEG	POS	NEG	POS	AMB	NEG	NEG	NEG	POS	POS	NEG	NEG	POS	NEG	NEG	POS	POS	POS	NEG	POS	NEG	NEG	NEG
220616	CC0049	CC49-MSSA (lukF-P83/lukM+)	POS	POS	NEG	POS	NEG	NEG	POS	NEG	AMB	POS	NEG	NEG	NEG	POS	POS	NEG	AMB	POS	AMB	NEG	POS	POS	POS	NEG	POS	NEG	NEG	NEG
228649	CC0049	CC49-MSSA (lukF-P83/lukM+)	POS	POS	NEG	POS	NEG	NEG	NEG	NEG	NEG	NEG	NEG	NEG	NEG	POS	POS	NEG	NEG	POS	POS	NEG	POS	POS	POS	NEG	POS	NEG	NEG	NEG
190994	CC0059	CC59-MSSA	POS	POS	NEG	POS	NEG	NEG	POS	NEG	POS	NEG	NEG	NEG	NEG	POS	POS	NEG	NEG	POS	NEG	POS	POS	POS	POS	NEG	NEG	NEG	NEG	NEG
275172	CC0088	CC88-MSSA	POS	POS	NEG	NEG	NEG	NEG	POS	NEG	POS	AMB	NEG	NEG	NEG	POS	NEG	NEG	POS	POS	NEG	POS	POS	POS	POS	NEG	NEG	POS	NEG	POS
275173	CC0088	CC88-MSSA	POS	POS	NEG	NEG	NEG	NEG	POS	NEG	POS	AMB	NEG	NEG	NEG	POS	NEG	NEG	POS	POS	NEG	POS	POS	POS	POS	NEG	NEG	POS	AMB	POS
275174	CC0088	CC88-MSSA	POS	POS	NEG	NEG	NEG	NEG	POS	NEG	AMB	POS	NEG	NEG	NEG	POS	NEG	NEG	POS	POS	NEG	POS	POS	POS	POS	NEG	NEG	POS	POS	POS
170971	CC0097	CC97-MSSA	POS	POS	NEG	NEG	NEG	NEG	POS	NEG	POS	AMB	NEG	NEG	NEG	POS	POS	NEG	POS	POS	POS	NEG	POS	POS	POS	NEG	NEG	POS	POS	POS
112745	CC0097	CC97-MSSA	POS	POS	NEG	NEG	NEG	NEG	NEG	NEG	NEG	NEG	NEG	NEG	NEG	POS	POS	NEG	POS	POS	POS	NEG	POS	POS	POS	NEG	NEG	POS	POS	POS
112748	CC0097	CC97-MSSA	POS	POS	NEG	NEG	NEG	NEG	POS	NEG	POS	AMB	NEG	NEG	NEG	POS	POS	NEG	POS	POS	POS	NEG	POS	POS	POS	NEG	NEG	POS	POS	POS
112750	CC0097	CC97-MSSA	POS	POS	NEG	NEG	NEG	NEG	POS	NEG	POS	AMB	NEG	NEG	NEG	POS	POS	NEG	POS	POS	POS	NEG	POS	POS	POS	NEG	NEG	POS	POS	POS
112762	CC0097	CC97-MSSA	POS	POS	NEG	NEG	NEG	NEG	POS	NEG	AMB	POS	NEG	NEG	NEG	POS	POS	NEG	POS	POS	POS	NEG	POS	POS	POS	NEG	NEG	POS	POS	POS
112763	CC0097	CC97-MSSA	POS	POS	NEG	NEG	NEG	NEG	POS	NEG	AMB	POS	NEG	NEG	NEG	POS	POS	NEG	POS	POS	POS	NEG	POS	POS	POS	NEG	NEG	POS	POS	POS
112764	CC0097	CC97-MSSA	POS	POS	NEG	NEG	NEG	NEG	POS	NEG	AMB	POS	NEG	NEG	NEG	POS	POS	NEG	POS	POS	POS	NEG	POS	POS	POS	NEG	NEG	POS	POS	POS
170945	CC0097	CC97-MSSA	POS	POS	NEG	NEG	NEG	NEG	POS	NEG	POS	AMB	NEG	NEG	NEG	POS	POS	NEG	POS	POS	POS	NEG	POS	POS	POS	NEG	NEG	POS	POS	POS
170954	CC0097	CC97-MSSA	POS	POS	NEG	NEG	NEG	NEG	POS	NEG	POS	AMB	NEG	NEG	NEG	POS	POS	NEG	POS	POS	POS	NEG	POS	POS	POS	NEG	NEG	POS	POS	POS
170958	CC0097	CC97-MSSA	POS	POS	NEG	NEG	NEG	NEG	POS	NEG	POS	AMB	NEG	NEG	NEG	POS	POS	NEG	POS	POS	POS	NEG	POS	POS	POS	NEG	NEG	POS	AMB	POS
170963	CC0097	CC97-MSSA	POS	POS	NEG	NEG	NEG	NEG	POS	NEG	POS	AMB	NEG	NEG	NEG	POS	POS	NEG	POS	POS	POS	NEG	POS	POS	POS	NEG	NEG	POS	AMB	POS
170964	CC0097	CC97-MSSA	POS	POS	NEG	NEG	NEG	NEG	POS	NEG	POS	AMB	NEG	NEG	NEG	POS	POS	NEG	POS	POS	POS	NEG	POS	POS	POS	NEG	NEG	POS	AMB	POS
170965	CC0097	CC97-MSSA	POS	POS	NEG	NEG	NEG	NEG	POS	NEG	POS	AMB	NEG	NEG	NEG	POS	POS	NEG	POS	POS	POS	NEG	POS	POS	POS	NEG	NEG	POS	AMB	POS
170966	CC0097	CC97-MSSA	POS	POS	NEG	NEG	NEG	NEG	POS	NEG	POS	AMB	NEG	NEG	NEG	POS	POS	NEG	POS	POS	POS	NEG	POS	POS	POS	NEG	NEG	POS	POS	POS
170974	CC0097	CC97-MSSA	POS	POS	NEG	NEG	NEG	NEG	POS	NEG	POS	AMB	NEG	NEG	NEG	POS	POS	NEG	POS	POS	POS	NEG	POS	POS	POS	NEG	NEG	POS	AMB	POS
170976	CC0097	CC97-MSSA	POS	POS	NEG	NEG	NEG	NEG	POS	NEG	POS	AMB	NEG	NEG	NEG	POS	POS	NEG	POS	POS	POS	NEG	POS	POS	POS	NEG	NEG	POS	AMB	POS
112744	CC0097	CC97-MSSA	POS	POS	NEG	NEG	NEG	NEG	POS	NEG	POS	AMB	NEG	NEG	NEG	POS	POS	NEG	POS	POS	POS	NEG	POS	POS	POS	NEG	NEG	POS	POS	POS
112749	CC0097	CC97-MSSA	POS	POS	NEG	NEG	NEG	NEG	POS	NEG	AMB	POS	NEG	NEG	NEG	POS	POS	NEG	POS	POS	POS	NEG	POS	POS	POS	NEG	NEG	POS	POS	POS
112757	CC0097	CC97-MSSA	POS	POS	NEG	NEG	NEG	NEG	POS	NEG	AMB	POS	NEG	NEG	NEG	POS	POS	NEG	POS	POS	POS	NEG	POS	POS	POS	NEG	NEG	POS	POS	POS
170973	CC0097	CC97-MSSA	POS	POS	NEG	NEG	NEG	NEG	NEG	NEG	NEG	NEG	NEG	NEG	NEG	POS	POS	NEG	POS	POS	POS	NEG	POS	POS	POS	NEG	NEG	POS	AMB	POS
207756	CC0097	CC97-MSSA	POS	POS	NEG	NEG	NEG	NEG	POS	NEG	AMB	POS	NEG	NEG	NEG	POS	POS	NEG	POS	POS	POS	NEG	POS	POS	POS	NEG	NEG	POS	POS	POS
206171	CC0097	CC97-MSSA	POS	POS	NEG	NEG	NEG	NEG	POS	NEG	AMB	POS	NEG	NEG	NEG	POS	POS	NEG	POS	POS	POS	NEG	POS	POS	POS	NEG	NEG	POS	POS	POS
275169	CC0097	CC97-MSSA-[ccrAA]	POS	POS	NEG	NEG	NEG	NEG	POS	NEG	POS	AMB	NEG	NEG	NEG	POS	POS	NEG	POS	POS	POS	NEG	POS	POS	POS	NEG	NEG	POS	POS	POS
275168	CC0097	CC97-MSSA-[ccrAA]	POS	POS	NEG	NEG	NEG	NEG	POS	NEG	POS	AMB	NEG	NEG	NEG	POS	POS	NEG	POS	POS	POS	NEG	POS	POS	POS	NEG	NEG	POS	POS	POS
118320	CC0130	CC130-MSSA (lukF-P83/M+)	POS	POS	NEG	NEG	NEG	NEG	POS	NEG	POS	NEG	NEG	NEG	NEG	POS	POS	NEG	POS	NEG	NEG	NEG	NEG	POS	POS	NEG	POS	NEG	NEG	NEG
203236	CC0130	CC130-MRSA-XI	POS	POS	NEG	NEG	NEG	NEG	POS	NEG	POS	AMB	NEG	NEG	NEG	POS	POS	NEG	POS	NEG	NEG	NEG	NEG	POS	POS	NEG	POS	NEG	AMB	NEG
204204	CC0130	CC130-MRSA-XI	POS	POS	NEG	NEG	NEG	NEG	POS	NEG	POS	NEG	NEG	NEG	NEG	POS	POS	NEG	AMB	NEG	NEG	NEG	NEG	POS	POS	NEG	POS	NEG	NEG	NEG
289122	CC0130	CC130-MRSA-XI	POS	POS	NEG	NEG	NEG	NEG	POS	NEG	POS	NEG	NEG	NEG	NEG	POS	POS	NEG	POS	NEG	NEG	NEG	NEG	POS	POS	NEG	POS	NEG	NEG	NEG
225779	CC0130	CC130-MRSA-XI	POS	POS	NEG	NEG	NEG	NEG	NEG	NEG	NEG	NEG	NEG	NEG	NEG	POS	POS	NEG	POS	NEG	NEG	NEG	NEG	POS	POS	NEG	POS	NEG	NEG	NEG
225780	CC0130	CC130-MRSA-XI	POS	POS	NEG	NEG	NEG	NEG	NEG	NEG	NEG	NEG	NEG	NEG	NEG	POS	POS	NEG	POS	NEG	NEG	NEG	NEG	POS	POS	NEG	POS	NEG	NEG	NEG
170959	CC0130	CC130-MRSA-XI	POS	POS	NEG	NEG	NEG	NEG	POS	NEG	POS	NEG	NEG	NEG	NEG	POS	POS	NEG	NEG	NEG	NEG	NEG	NEG	POS	POS	NEG	POS	NEG	NEG	NEG
179029	CC0130	CC130-MRSA-XI	POS	POS	NEG	NEG	NEG	NEG	POS	NEG	POS	NEG	NEG	NEG	NEG	POS	POS	NEG	POS	NEG	NEG	NEG	NEG	POS	POS	NEG	POS	NEG	NEG	NEG
116254	CC0133	CC133-MSSA	POS	POS	NEG	NEG	NEG	NEG	POS	NEG	POS	NEG	NEG	NEG	NEG	POS	POS	NEG	NEG	NEG	NEG	NEG	NEG	POS	POS	NEG	POS	NEG	NEG	NEG
207761	CC0133	CC133-MSSA	POS	POS	NEG	NEG	NEG	NEG	POS	NEG	POS	NEG	NEG	NEG	NEG	POS	POS	NEG	NEG	NEG	NEG	NEG	NEG	POS	POS	NEG	POS	NEG	NEG	NEG
213103	CC0133	CC133-MSSA	POS	POS	NEG	NEG	NEG	NEG	POS	NEG	POS	AMB	NEG	NEG	NEG	POS	POS	NEG	POS	NEG	NEG	AMB	NEG	POS	POS	NEG	POS	AMB	AMB	NEG
275736	CC0133	CC133-MSSA	POS	POS	NEG	NEG	NEG	NEG	POS	NEG	POS	NEG	NEG	NEG	NEG	POS	POS	NEG	NEG	NEG	NEG	NEG	NEG	POS	POS	NEG	POS	NEG	NEG	NEG
275737	CC0133	CC133-MSSA	POS	POS	NEG	NEG	NEG	NEG	POS	NEG	POS	NEG	NEG	NEG	NEG	POS	POS	NEG	NEG	NEG	NEG	NEG	NEG	POS	POS	NEG	POS	NEG	NEG	NEG
275738	CC0133	CC133-MSSA	POS	POS	NEG	NEG	NEG	NEG	POS	NEG	POS	NEG	NEG	NEG	NEG	POS	POS	NEG	NEG	NEG	NEG	NEG	NEG	POS	POS	NEG	POS	NEG	NEG	NEG
275739	CC0133	CC133-MSSA	POS	POS	NEG	NEG	NEG	NEG	POS	NEG	POS	NEG	NEG	NEG	NEG	POS	POS	NEG	NEG	NEG	NEG	NEG	NEG	POS	POS	NEG	POS	NEG	NEG	NEG
112755	CC0133	CC133-MSSA	POS	POS	NEG	NEG	NEG	NEG	POS	NEG	POS	NEG	NEG	NEG	NEG	POS	POS	NEG	NEG	NEG	NEG	NEG	NEG	POS	POS	NEG	POS	NEG	NEG	NEG
225778	CC0398	CC398-MRSA-V/VT	POS	POS	NEG	NEG	NEG	NEG	POS	NEG	POS	NEG	NEG	NEG	NEG	POS	POS	POS	NEG	NEG	NEG	NEG	NEG	POS	POS	NEG	NEG	POS	POS	NEG
123159	ST0425	ST425-MSSA	POS	POS	NEG	NEG	NEG	NEG	POS	NEG	POS	AMB	NEG	NEG	NEG	POS	POS	NEG	NEG	NEG	NEG	NEG	NEG	POS	POS	NEG	NEG	NEG	NEG	NEG
86991	ST0425	ST425-MSSA	POS	POS	NEG	NEG	NEG	NEG	POS	NEG	POS	POS	NEG	NEG	NEG	POS	POS	NEG	NEG	NEG	NEG	NEG	NEG	POS	POS	NEG	NEG	NEG	NEG	NEG
112756	ST0425	ST425-MSSA	POS	POS	NEG	NEG	NEG	NEG	POS	NEG	POS	AMB	NEG	NEG	NEG	POS	POS	NEG	NEG	NEG	NEG	NEG	NEG	POS	POS	NEG	NEG	NEG	AMB	NEG
195207	ST0425	ST425-MSSA	POS	POS	NEG	NEG	NEG	NEG	POS	NEG	AMB	POS	NEG	NEG	NEG	POS	POS	NEG	NEG	NEG	NEG	NEG	NEG	POS	POS	NEG	NEG	NEG	AMB	NEG
223609	ST0425	ST425-MSSA	POS	POS	NEG	NEG	NEG	NEG	POS	NEG	POS	AMB	NEG	NEG	NEG	POS	POS	NEG	POS	NEG	NEG	NEG	NEG	POS	POS	NEG	NEG	AMB	NEG	NEG
275740	ST0425	ST425-MSSA	POS	POS	NEG	NEG	NEG	NEG	POS	NEG	POS	AMB	NEG	NEG	NEG	POS	POS	NEG	NEG	NEG	NEG	NEG	NEG	POS	POS	NEG	NEG	NEG	AMB	NEG
174889	ST0425	ST425-MSSA	POS	POS	NEG	NEG	NEG	NEG	POS	NEG	POS	AMB	NEG	NEG	NEG	POS	POS	NEG	NEG	NEG	NEG	NEG	NEG	POS	POS	NEG	NEG	NEG	NEG	NEG
203230	ST0425	ST425-MSSA	POS	POS	NEG	NEG	NEG	NEG	POS	NEG	AMB	POS	NEG	AMB	NEG	POS	POS	NEG	POS	POS	NEG	POS	NEG	POS	POS	NEG	NEG	AMB	POS	NEG
203242	ST0425	ST425-MSSA	POS	POS	NEG	NEG	NEG	NEG	POS	NEG	AMB	POS	NEG	AMB	NEG	POS	POS	NEG	POS	POS	NEG	POS	NEG	POS	POS	NEG	NEG	NEG	POS	NEG
203243	ST0425	ST425-MSSA	POS	POS	NEG	NEG	NEG	NEG	POS	NEG	AMB	POS	NEG	AMB	NEG	POS	POS	NEG	POS	POS	NEG	POS	NEG	POS	POS	NEG	NEG	AMB	POS	NEG
207951	ST0425	ST425-MSSA	POS	POS	NEG	NEG	NEG	NEG	POS	NEG	POS	AMB	NEG	NEG	NEG	POS	POS	NEG	NEG	NEG	NEG	NEG	NEG	POS	POS	NEG	NEG	NEG	NEG	NEG
207981	ST0425	ST425-MSSA	POS	POS	NEG	NEG	NEG	NEG	POS	NEG	POS	AMB	NEG	NEG	NEG	POS	POS	NEG	NEG	NEG	NEG	NEG	NEG	POS	POS	NEG	NEG	NEG	AMB	NEG
195212	ST0425	ST425-MSSA	POS	POS	NEG	NEG	NEG	NEG	POS	NEG	AMB	POS	NEG	NEG	NEG	POS	POS	NEG	AMB	NEG	NEG	AMB	NEG	POS	POS	NEG	NEG	NEG	AMB	NEG
195206	ST0425	ST425-MSSA	POS	POS	NEG	NEG	NEG	NEG	POS	NEG	AMB	POS	NEG	AMB	NEG	POS	POS	NEG	POS	NEG	NEG	AMB	NEG	POS	POS	NEG	NEG	NEG	POS	NEG
195202	ST0425	ST425-MSSA	POS	POS	NEG	NEG	NEG	NEG	POS	NEG	AMB	POS	NEG	NEG	NEG	POS	POS	NEG	NEG	NEG	NEG	NEG	NEG	POS	POS	NEG	NEG	NEG	NEG	NEG
216042	ST0425	ST425-MSSA	POS	POS	NEG	NEG	NEG	NEG	POS	NEG	AMB	POS	NEG	NEG	NEG	POS	POS	NEG	POS	NEG	NEG	AMB	NEG	POS	POS	NEG	NEG	NEG	POS	NEG
223608	ST0425	ST425-MSSA	POS	POS	NEG	NEG	NEG	NEG	POS	NEG	POS	AMB	NEG	AMB	NEG	POS	POS	NEG	POS	NEG	NEG	NEG	NEG	POS	POS	NEG	NEG	AMB	NEG	NEG
223612	ST0425	ST425-MSSA	POS	POS	NEG	NEG	NEG	NEG	POS	NEG	POS	AMB	NEG	NEG	NEG	POS	POS	NEG	POS	NEG	NEG	NEG	NEG	POS	POS	NEG	NEG	NEG	NEG	NEG
225733	ST0425	ST425-MSSA	POS	POS	NEG	NEG	NEG	NEG	POS	NEG	AMB	POS	NEG	AMB	NEG	POS	POS	NEG	POS	NEG	NEG	AMB	NEG	POS	POS	NEG	NEG	NEG	AMB	NEG
230321	ST0425	ST425-MSSA	POS	POS	NEG	NEG	NEG	NEG	POS	NEG	AMB	POS	NEG	NEG	NEG	POS	POS	NEG	POS	NEG	NEG	AMB	NEG	POS	POS	NEG	NEG	NEG	POS	NEG
232368	ST0425	ST425-MSSA	POS	POS	NEG	NEG	NEG	NEG	POS	NEG	AMB	POS	NEG	NEG	NEG	POS	POS	NEG	POS	NEG	NEG	NEG	NEG	POS	POS	NEG	NEG	NEG	AMB	NEG
204215	ST0425	ST425-MSSA	POS	POS	NEG	NEG	NEG	NEG	POS	NEG	AMB	POS	NEG	AMB	NEG	POS	POS	NEG	POS	NEG	NEG	AMB	NEG	POS	POS	NEG	NEG	NEG	POS	NEG
204218	ST0425	ST425-MSSA	POS	POS	NEG	NEG	NEG	NEG	POS	NEG	AMB	POS	NEG	AMB	NEG	POS	POS	NEG	POS	NEG	NEG	AMB	NEG	POS	POS	NEG	NEG	NEG	POS	NEG
204222	ST0425	ST425-MSSA	POS	POS	NEG	NEG	NEG	NEG	POS	NEG	AMB	POS	NEG	NEG	NEG	POS	POS	NEG	POS	NEG	NEG	AMB	NEG	POS	POS	NEG	NEG	NEG	POS	NEG
204226	ST0425	ST425-MSSA	POS	POS	NEG	NEG	NEG	NEG	POS	NEG	AMB	POS	NEG	NEG	NEG	POS	POS	NEG	AMB	NEG	NEG	NEG	NEG	POS	POS	NEG	NEG	NEG	AMB	NEG
204906	ST0425	ST425-MSSA	POS	POS	NEG	NEG	NEG	NEG	POS	NEG	AMB	POS	NEG	NEG	NEG	POS	POS	NEG	POS	NEG	NEG	AMB	NEG	POS	POS	NEG	NEG	NEG	AMB	NEG
204908	ST0425	ST425-MSSA	POS	POS	NEG	NEG	NEG	NEG	POS	NEG	AMB	POS	NEG	AMB	NEG	POS	POS	NEG	POS	POS	NEG	POS	NEG	POS	POS	NEG	NEG	NEG	POS	NEG
206170	ST0425	ST425-MSSA	POS	POS	NEG	NEG	NEG	NEG	POS	NEG	AMB	POS	NEG	NEG	NEG	POS	POS	NEG	AMB	NEG	NEG	NEG	NEG	POS	POS	NEG	NEG	NEG	AMB	NEG
207765	ST0425	ST425-MSSA	POS	POS	NEG	NEG	NEG	NEG	POS	NEG	POS	AMB	NEG	NEG	NEG	POS	POS	NEG	NEG	NEG	NEG	NEG	NEG	POS	POS	NEG	NEG	NEG	AMB	NEG
240048	CC599	CC599-MRSA-XI	POS	POS	NEG	NEG	NEG	NEG	AMB	NEG	AMB	POS	NEG	AMB	NEG	AMB	POS	NEG	AMB	NEG	NEG	NEG	NEG	AMB	POS	NEG	NEG	NEG	NEG	NEG
225730	CC0692	CC692-MSSA	POS	POS	NEG	NEG	NEG	NEG	POS	NEG	AMB	NEG	NEG	POS	NEG	NEG	NEG	NEG	NEG	NEG	NEG	AMB	NEG	POS	NEG	NEG	POS	NEG	NEG	POS
112746	CC0692	CC692-MSSA	POS	POS	NEG	NEG	NEG	NEG	POS	NEG	POS	NEG	NEG	AMB	NEG	NEG	NEG	NEG	NEG	NEG	NEG	NEG	NEG	POS	POS	NEG	POS	NEG	NEG	POS
112754	CC0692	CC692-MSSA	POS	POS	NEG	NEG	NEG	NEG	POS	NEG	POS	NEG	NEG	AMB	NEG	AMB	AMB	NEG	POS	NEG	NEG	NEG	NEG	POS	POS	NEG	POS	NEG	NEG	POS
112758	CC0692	CC692-MSSA	POS	POS	NEG	NEG	NEG	NEG	POS	NEG	POS	NEG	NEG	AMB	NEG	NEG	NEG	NEG	NEG	NEG	NEG	NEG	NEG	POS	POS	NEG	POS	NEG	NEG	POS
112759	CC0692	CC692-MSSA	POS	POS	NEG	NEG	NEG	NEG	POS	NEG	POS	NEG	NEG	AMB	NEG	NEG	NEG	NEG	NEG	NEG	NEG	NEG	NEG	POS	POS	NEG	POS	NEG	NEG	POS
124745	CC0692	CC692-MSSA	POS	POS	NEG	NEG	NEG	NEG	POS	NEG	POS	NEG	NEG	AMB	NEG	NEG	NEG	NEG	AMB	NEG	NEG	NEG	NEG	POS	POS	NEG	POS	NEG	NEG	POS
170952	CC0692	CC692-MSSA	POS	POS	NEG	NEG	NEG	NEG	POS	NEG	POS	NEG	NEG	AMB	NEG	NEG	NEG	NEG	NEG	NEG	NEG	NEG	NEG	POS	POS	NEG	POS	NEG	NEG	POS
170953	CC0692	CC692-MSSA	POS	POS	NEG	NEG	NEG	NEG	POS	NEG	POS	NEG	NEG	AMB	NEG	NEG	NEG	NEG	NEG	NEG	NEG	NEG	NEG	POS	POS	NEG	POS	NEG	NEG	POS
170970	CC0692	CC692-MSSA	POS	POS	NEG	NEG	NEG	NEG	POS	NEG	POS	NEG	NEG	AMB	NEG	NEG	NEG	NEG	NEG	NEG	NEG	NEG	NEG	POS	POS	NEG	POS	NEG	NEG	POS
170950	CC0707	CC707-MSSA	POS	POS	NEG	NEG	AMB	NEG	POS	NEG	POS	AMB	NEG	NEG	NEG	POS	POS	NEG	AMB	NEG	NEG	NEG	NEG	POS	POS	NEG	NEG	NEG	NEG	NEG
124948	ST0890	ST890-MSSA	POS	POS	NEG	NEG	NEG	NEG	NEG	NEG	NEG	NEG	NEG	NEG	NEG	POS	POS	NEG	NEG	NEG	NEG	NEG	NEG	POS	POS	NEG	NEG	POS	NEG	NEG
220618	CC1956	CC1956-MSSA	POS	POS	NEG	POS	NEG	NEG	POS	NEG	AMB	AMB	NEG	POS	NEG	POS	POS	NEG	POS	NEG	NEG	AMB	NEG	POS	POS	NEG	POS	NEG	AMB	NEG
112742	ST2425	ST2425-MSSA	POS	POS	NEG	NEG	NEG	NEG	NEG	NEG	NEG	NEG	NEG	NEG	NEG	POS	POS	NEG	NEG	NEG	NEG	NEG	NEG	POS	POS	NEG	POS	NEG	NEG	NEG
170957	ST2425	ST2425-MSSA	POS	POS	NEG	NEG	NEG	NEG	NEG	NEG	NEG	NEG	NEG	NEG	NEG	POS	POS	NEG	NEG	NEG	NEG	NEG	NEG	POS	POS	NEG	POS	NEG	NEG	NEG
170947	ST2691	ST2691-MSSA	POS	POS	NEG	POS	NEG	NEG	NEG	NEG	NEG	NEG	NEG	NEG	NEG	POS	POS	NEG	NEG	NEG	NEG	NEG	NEG	POS	POS	NEG	POS	NEG	NEG	NEG
170961	ST2691	ST2691-MSSA	POS	POS	NEG	POS	NEG	NEG	NEG	NEG	NEG	NEG	NEG	NEG	NEG	POS	POS	NEG	NEG	NEG	NEG	NEG	NEG	POS	POS	NEG	POS	NEG	NEG	NEG
112753	CC2767	ST2279-MSSA	POS	POS	NEG	NEG	NEG	NEG	POS	NEG	POS	POS	NEG	NEG	NEG	POS	POS	NEG	AMB	NEG	NEG	NEG	NEG	POS	POS	NEG	NEG	NEG	AMB	NEG
112761	CC2767	ST2279-MSSA	POS	POS	NEG	NEG	NEG	NEG	POS	NEG	POS	POS	NEG	NEG	NEG	POS	POS	NEG	NEG	NEG	NEG	NEG	NEG	POS	POS	NEG	NEG	NEG	NEG	NEG
228651	ST2963	ST2963-MSSA	POS	POS	NEG	POS	NEG	NEG	POS	NEG	POS	NEG	NEG	NEG	AMB	POS	POS	NEG	POS	NEG	NEG	NEG	NEG	POS	POS	NEG	NEG	NEG	NEG	NEG
203244	ST3237	ST3237-MSSA	POS	POS	NEG	POS	NEG	NEG	POS	NEG	AMB	POS	NEG	AMB	NEG	POS	POS	NEG	NEG	NEG	NEG	AMB	NEG	POS	POS	NEG	NEG	POS	NEG	NEG

ISOLATE ID	CLONAL COMPLEX (by array)	STRAIN AFFILIATION (by array)	ADHAESION FACTORS / MSCRAMM GENES											IMMUNOD.AG.B		DEFENSIN RESIST.		TRANSFERRIN BINDING PROT			PUTATIVE TRANSPORTERS			
			Ser-Asp rich fibrinogen-/bone sialoprotein-binding protein D					van Willebrand factor binding protein						immunodominant antigen B		defensin resistance protein		transferrin-binding protein			hypothetical protein, similar to integral membrane protein LmrP			
			sdrD	sdrD (cons)	sdrD (COL+MW2)	sdrD (Mu50)	sdrD (other)	vwb	vwb (cons)	vwb (COL+MW2)	vwb (MRSA252)	vwb (Mu50)	vwb (RF122)	isaB	isaB (MRSA252)	mprF (COL+MW2)	mprF (Mu50+252)	isdA (cons)	isdA (MRSA252)	isdA (Other Than MRSA252)	lmrP (OtherThanRF122)	lmrP (OtherThanRF122)	lmrP (RF122)	lmrP (RF122)
203228	CC0001	CC1-MSSA	POS	POS	POS	NEG	NEG	POS	POS	POS	NEG	NEG	NEG	POS	AMB	AMB	POS	POS	AMB	POS	POS	POS	AMB	NEG
203229	CC0001	CC1-MSSA	POS	POS	POS	NEG	NEG	POS	POS	POS	NEG	NEG	NEG	AMB	POS	POS	AMB	POS	AMB	POS	POS	POS	NEG	NEG
203233	CC0001	CC1-MSSA	POS	POS	POS	NEG	NEG	POS	POS	POS	NEG	NEG	NEG	POS	AMB	AMB	POS	POS	AMB	POS	POS	POS	NEG	NEG
220334	CC0001	CC1-MSSA	POS	POS	POS	NEG	NEG	POS	POS	POS	NEG	NEG	NEG	POS	AMB	AMB	POS	POS	AMB	POS	POS	POS	NEG	NEG
223621	CC0001	CC1-MSSA	POS	POS	POS	NEG	NEG	POS	POS	POS	NEG	NEG	NEG	AMB	POS	POS	AMB	POS	AMB	POS	POS	POS	NEG	NEG
289123	CC0001	CC1-MRSA-IV, WA MRSA-1/57	POS	POS	POS	NEG	NEG	POS	POS	POS	NEG	NEG	NEG	AMB	POS	POS	AMB	POS	NEG	POS	POS	POS	NEG	NEG
289124	CC0001	CC1-MRSA-IV, WA MRSA-1/57	POS	POS	POS	NEG	NEG	POS	POS	POS	NEG	NEG	NEG	POS	AMB	POS	AMB	POS	NEG	POS	POS	POS	NEG	NEG
275170	CC0005	CC5-MSSA	POS	POS	NEG	POS	NEG	POS	POS	NEG	NEG	POS	NEG	POS	AMB	AMB	POS	POS	NEG	POS	POS	POS	NEG	NEG
275171	CC0005	CC5-MSSA	POS	POS	NEG	POS	NEG	POS	POS	NEG	NEG	POS	NEG	POS	AMB	AMB	POS	POS	NEG	POS	POS	POS	NEG	NEG
112747	CC0005	CC5-MSSA	NEG	NEG	NEG	NEG	NEG	POS	POS	NEG	NEG	POS	NEG	POS	AMB	AMB	POS	POS	NEG	POS	POS	POS	NEG	NEG
112752	CC0006	CC6-MSSA	POS	POS	NEG	POS	NEG	POS	POS	NEG	POS	NEG	NEG	POS	AMB	POS	AMB	POS	NEG	POS	POS	POS	NEG	NEG
275178	CC0007	CC7-MSSA	POS	POS	NEG	POS	NEG	POS	POS	NEG	NEG	NEG	NEG	POS	AMB	POS	AMB	POS	NEG	POS	POS	POS	NEG	NEG
275166	CC0008	CC8-MSSA	POS	POS	POS	NEG	NEG	POS	POS	POS	NEG	NEG	NEG	POS	AMB	POS	AMB	POS	NEG	POS	POS	POS	NEG	NEG
275176	CC0008	CC8-MSSA	POS	POS	POS	NEG	NEG	POS	POS	POS	NEG	NEG	NEG	POS	AMB	POS	AMB	POS	NEG	POS	POS	POS	NEG	NEG
275177	CC0008	CC8-MSSA	POS	POS	POS	NEG	NEG	POS	POS	POS	NEG	NEG	NEG	POS	AMB	POS	AMB	POS	NEG	POS	POS	POS	NEG	NEG
275167	CC0009	CC9-MSSA	POS	POS	NEG	NEG	POS	POS	POS	NEG	POS	NEG	NEG	POS	AMB	AMB	POS	POS	NEG	POS	POS	POS	NEG	NEG
170968	CC0012	CC12-MSSA	POS	POS	POS	NEG	NEG	POS	POS	NEG	NEG	NEG	NEG	POS	AMB	POS	AMB	POS	NEG	POS	POS	POS	NEG	NEG
275175	CC0015	CC15-MSSA	POS	POS	NEG	POS	NEG	POS	POS	NEG	NEG	NEG	NEG	POS	AMB	POS	AMB	POS	POS	NEG	POS	POS	NEG	NEG
170949	CC0015	CC15-MSSA	POS	POS	NEG	POS	NEG	POS	POS	NEG	NEG	NEG	NEG	POS	AMB	POS	AMB	POS	POS	NEG	POS	POS	NEG	NEG
228658	CC0022	CC22-MSSA	POS	POS	NEG	NEG	POS	POS	POS	NEG	NEG	NEG	POS	NEG	POS	AMB	POS	POS	AMB	POS	POS	POS	NEG	NEG
289125	CC0022	CC22-MRSA-IV (PVL+)	POS	POS	NEG	NEG	POS	POS	POS	NEG	NEG	NEG	POS	NEG	POS	AMB	POS	POS	AMB	POS	POS	POS	NEG	NEG
289126	CC0022	CC22-MRSA-IV (PVL+)	POS	POS	NEG	NEG	POS	POS	POS	NEG	NEG	NEG	POS	NEG	POS	AMB	POS	POS	NEG	POS	POS	POS	NEG	NEG
289127	CC0022	CC22-MRSA-IV (PVL+)	POS	POS	NEG	NEG	POS	POS	POS	NEG	NEG	NEG	POS	NEG	POS	AMB	POS	POS	AMB	POS	POS	POS	NEG	NEG
131939	CC0025	CC25-MSSA	NEG	NEG	NEG	NEG	NEG	POS	POS	NEG	NEG	NEG	NEG	POS	AMB	AMB	POS	POS	NEG	POS	POS	POS	NEG	NEG
275165	CC0030	CC30-MSSA (lukF-P83/lukM+)	POS	POS	NEG	NEG	POS	POS	POS	NEG	POS	NEG	NEG	NEG	POS	AMB	POS	POS	POS	NEG	POS	POS	NEG	NEG
275741	CC0030	CC30-MSSA	POS	POS	NEG	NEG	POS	POS	POS	NEG	POS	NEG	NEG	NEG	POS	AMB	POS	POS	POS	NEG	POS	POS	NEG	NEG
124492	CC0049	CC49-MSSA (lukF-P83/lukM+)	POS	NEG	NEG	NEG	POS	POS	POS	NEG	NEG	NEG	NEG	POS	NEG	POS	NEG	POS	NEG	POS	NEG	NEG	POS	POS
220616	CC0049	CC49-MSSA (lukF-P83/lukM+)	POS	POS	NEG	NEG	POS	POS	POS	NEG	NEG	AMB	NEG	POS	AMB	POS	AMB	POS	NEG	POS	NEG	NEG	POS	POS
228649	CC0049	CC49-MSSA (lukF-P83/lukM+)	POS	POS	NEG	NEG	POS	POS	POS	NEG	NEG	NEG	NEG	POS	NEG	POS	AMB	POS	NEG	POS	NEG	NEG	POS	POS
190994	CC0059	CC59-MSSA	POS	POS	NEG	NEG	NEG	POS	POS	NEG	NEG	NEG	NEG	POS	NEG	POS	NEG	POS	NEG	POS	NEG	NEG	POS	POS
275172	CC0088	CC88-MSSA	POS	POS	NEG	NEG	POS	POS	POS	NEG	NEG	POS	NEG	POS	AMB	POS	NEG	POS	NEG	POS	POS	NEG	NEG	NEG
275173	CC0088	CC88-MSSA	POS	POS	NEG	NEG	POS	POS	POS	NEG	NEG	POS	NEG	POS	AMB	POS	AMB	POS	NEG	POS	POS	POS	NEG	NEG
275174	CC0088	CC88-MSSA	POS	POS	NEG	NEG	POS	POS	POS	NEG	NEG	POS	NEG	POS	AMB	POS	AMB	POS	NEG	POS	POS	POS	NEG	NEG
170971	CC0097	CC97-MSSA	NEG	NEG	NEG	NEG	NEG	POS	POS	NEG	NEG	NEG	POS	POS	AMB	POS	AMB	POS	NEG	POS	POS	POS	NEG	NEG
112745	CC0097	CC97-MSSA	POS	POS	NEG	POS	NEG	POS	POS	NEG	NEG	NEG	POS	POS	AMB	POS	AMB	POS	NEG	POS	POS	POS	NEG	NEG
112748	CC0097	CC97-MSSA	POS	POS	NEG	POS	NEG	POS	POS	NEG	NEG	NEG	POS	POS	AMB	POS	AMB	POS	NEG	POS	POS	POS	NEG	NEG
112750	CC0097	CC97-MSSA	POS	POS	NEG	POS	NEG	POS	POS	NEG	NEG	NEG	POS	POS	AMB	POS	AMB	POS	NEG	POS	POS	POS	NEG	NEG
112762	CC0097	CC97-MSSA	POS	POS	NEG	POS	NEG	POS	POS	NEG	NEG	NEG	POS	POS	AMB	POS	AMB	POS	NEG	POS	POS	POS	NEG	NEG
112763	CC0097	CC97-MSSA	POS	POS	NEG	POS	NEG	POS	POS	NEG	NEG	NEG	POS	POS	AMB	POS	AMB	POS	NEG	POS	POS	POS	NEG	NEG
112764	CC0097	CC97-MSSA	POS	POS	NEG	POS	NEG	POS	POS	NEG	NEG	NEG	POS	POS	AMB	POS	AMB	POS	NEG	POS	POS	POS	NEG	NEG
170945	CC0097	CC97-MSSA	POS	POS	NEG	POS	NEG	POS	POS	NEG	NEG	NEG	POS	POS	AMB	POS	AMB	POS	NEG	POS	POS	POS	NEG	NEG
170954	CC0097	CC97-MSSA	POS	POS	NEG	POS	NEG	POS	POS	NEG	NEG	NEG	POS	POS	AMB	POS	AMB	POS	NEG	POS	POS	POS	NEG	NEG
170958	CC0097	CC97-MSSA	POS	POS	NEG	POS	NEG	POS	POS	NEG	NEG	NEG	POS	POS	AMB	POS	AMB	POS	NEG	POS	POS	POS	NEG	NEG
170963	CC0097	CC97-MSSA	POS	POS	NEG	POS	NEG	POS	POS	NEG	NEG	NEG	POS	POS	AMB	POS	AMB	POS	NEG	POS	POS	POS	NEG	NEG
170964	CC0097	CC97-MSSA	POS	POS	NEG	POS	NEG	POS	POS	NEG	NEG	NEG	POS	POS	AMB	POS	AMB	POS	NEG	POS	POS	POS	NEG	NEG
170965	CC0097	CC97-MSSA	POS	POS	NEG	POS	NEG	POS	POS	NEG	NEG	NEG	POS	POS	AMB	POS	AMB	POS	NEG	POS	POS	POS	NEG	NEG
170966	CC0097	CC97-MSSA	POS	POS	NEG	POS	NEG	POS	POS	NEG	NEG	NEG	POS	POS	AMB	POS	AMB	POS	NEG	POS	POS	POS	NEG	NEG
170974	CC0097	CC97-MSSA	POS	POS	NEG	POS	NEG	POS	POS	NEG	NEG	NEG	POS	POS	AMB	POS	AMB	POS	NEG	POS	POS	POS	NEG	NEG
170976	CC0097	CC97-MSSA	POS	POS	NEG	POS	NEG	POS	POS	NEG	NEG	NEG	POS	POS	AMB	POS	AMB	POS	NEG	POS	POS	POS	NEG	NEG
112744	CC0097	CC97-MSSA	POS	POS	NEG	POS	NEG	POS	POS	NEG	NEG	NEG	POS	POS	AMB	POS	AMB	POS	NEG	POS	POS	POS	NEG	NEG
112749	CC0097	CC97-MSSA	POS	POS	NEG	POS	NEG	POS	POS	NEG	NEG	NEG	POS	POS	AMB	POS	AMB	POS	NEG	POS	POS	POS	NEG	NEG
112757	CC0097	CC97-MSSA	POS	POS	NEG	POS	NEG	POS	POS	NEG	NEG	NEG	POS	POS	AMB	POS	AMB	POS	NEG	POS	POS	POS	NEG	NEG
170973	CC0097	CC97-MSSA	POS	POS	NEG	POS	NEG	POS	POS	NEG	NEG	NEG	POS	POS	AMB	POS	AMB	POS	NEG	POS	POS	POS	NEG	NEG
207756	CC0097	CC97-MSSA	POS	POS	NEG	POS	NEG	POS	POS	NEG	NEG	NEG	POS	AMB	POS	POS	AMB	POS	NEG	POS	POS	POS	NEG	NEG
206171	CC0097	CC97-MSSA	POS	POS	NEG	POS	NEG	POS	POS	NEG	NEG	NEG	POS	AMB	POS	POS	AMB	POS	NEG	POS	POS	POS	NEG	NEG
275169	CC0097	CC97-MSSA-[ccrAA]	POS	POS	NEG	POS	NEG	POS	POS	NEG	NEG	NEG	POS	POS	AMB	POS	AMB	POS	NEG	POS	POS	POS	NEG	NEG
275168	CC0097	CC97-MSSA-[ccrAA]	POS	POS	NEG	POS	NEG	POS	POS	NEG	NEG	NEG	POS	POS	AMB	POS	AMB	POS	NEG	POS	POS	POS	NEG	NEG
118320	CC0130	CC130-MSSA (lukF-P83/M+)	NEG	NEG	NEG	NEG	NEG	POS	POS	NEG	NEG	NEG	NEG	POS	POS	POS	AMB	POS	NEG	POS	NEG	NEG	POS	POS
203236	CC0130	CC130-MRSA-XI	POS	POS	NEG	AMB	NEG	POS	POS	NEG	NEG	NEG	NEG	POS	POS	POS	POS	POS	NEG	POS	NEG	AMB	POS	POS
204204	CC0130	CC130-MRSA-XI	POS	POS	NEG	NEG	NEG	POS	POS	NEG	NEG	NEG	NEG	AMB	POS	POS	AMB	POS	AMB	POS	NEG	NEG	POS	POS
289122	CC0130	CC130-MRSA-XI	POS	POS	NEG	NEG	NEG	POS	POS	NEG	NEG	NEG	NEG	AMB	POS	POS	AMB	POS	AMB	POS	NEG	NEG	POS	POS
225779	CC0130	CC130-MRSA-XI	POS	POS	NEG	NEG	NEG	POS	POS	NEG	NEG	NEG	NEG	AMB	POS	POS	AMB	POS	AMB	POS	NEG	NEG	POS	POS
225780	CC0130	CC130-MRSA-XI	POS	POS	NEG	NEG	NEG	POS	POS	NEG	NEG	NEG	NEG	AMB	POS	POS	AMB	POS	AMB	POS	NEG	NEG	POS	POS
170959	CC0130	CC130-MRSA-XI	POS	POS	NEG	NEG	NEG	POS	POS	NEG	NEG	NEG	NEG	POS	AMB	POS	AMB	POS	NEG	POS	NEG	NEG	POS	POS
179029	CC0130	CC130-MRSA-XI	POS	POS	NEG	NEG	NEG	POS	POS	NEG	NEG	NEG	NEG	POS	AMB	POS	AMB	POS	AMB	POS	NEG	NEG	POS	POS
116254	CC0133	CC133-MSSA	POS	POS	NEG	NEG	NEG	POS	POS	NEG	NEG	NEG	NEG	POS	AMB	NEG	NEG	POS	NEG	POS	NEG	NEG	POS	POS
207761	CC0133	CC133-MSSA	POS	POS	NEG	NEG	NEG	POS	POS	NEG	NEG	NEG	NEG	POS	AMB	NEG	POS	POS	NEG	POS	NEG	NEG	POS	POS
213103	CC0133	CC133-MSSA	NEG	NEG	NEG	NEG	NEG	POS	POS	NEG	NEG	NEG	NEG	POS	AMB	NEG	POS	POS	AMB	POS	NEG	NEG	POS	POS
275736	CC0133	CC133-MSSA	POS	POS	NEG	NEG	NEG	POS	POS	NEG	NEG	NEG	NEG	POS	AMB	NEG	NEG	POS	NEG	POS	NEG	NEG	POS	POS
275737	CC0133	CC133-MSSA	POS	POS	NEG	NEG	NEG	POS	POS	NEG	NEG	NEG	NEG	POS	AMB	NEG	NEG	POS	NEG	POS	NEG	NEG	POS	POS
275738	CC0133	CC133-MSSA	POS	POS	NEG	AMB	NEG	POS	POS	NEG	NEG	NEG	NEG	POS	AMB	NEG	NEG	POS	NEG	POS	NEG	NEG	POS	POS
275739	CC0133	CC133-MSSA	POS	POS	NEG	NEG	NEG	POS	POS	NEG	NEG	NEG	NEG	POS	AMB	NEG	NEG	POS	NEG	POS	NEG	NEG	POS	POS
112755	CC0133	CC133-MSSA	POS	POS	NEG	AMB	NEG	POS	POS	NEG	NEG	NEG	NEG	POS	POS	NEG	AMB	POS	NEG	POS	NEG	NEG	POS	POS
225778	CC0398	CC398-MRSA-V/VT	POS	POS	NEG	NEG	NEG	POS	POS	NEG	NEG	NEG	NEG	NEG	POS	NEG	NEG	POS	POS	NEG	POS	POS	NEG	NEG
123159	ST0425	ST425-MSSA	POS	POS	NEG	NEG	POS	POS	POS	NEG	NEG	NEG	NEG	POS	AMB	NEG	AMB	POS	NEG	POS	NEG	NEG	POS	POS
86991	ST0425	ST425-MSSA	POS	POS	NEG	NEG	POS	POS	POS	NEG	NEG	NEG	NEG	POS	POS	NEG	NEG	POS	NEG	POS	NEG	NEG	POS	POS
112756	ST0425	ST425-MSSA	POS	POS	NEG	NEG	POS	POS	POS	NEG	NEG	NEG	NEG	POS	AMB	NEG	NEG	POS	NEG	POS	NEG	NEG	POS	POS
195207	ST0425	ST425-MSSA	POS	POS	NEG	NEG	POS	POS	POS	NEG	NEG	NEG	NEG	POS	AMB	NEG	POS	POS	AMB	POS	NEG	NEG	POS	POS
223609	ST0425	ST425-MSSA	POS	POS	NEG	NEG	POS	POS	POS	NEG	NEG	NEG	NEG	AMB	POS	NEG	POS	POS	AMB	POS	AMB	NEG	POS	POS
275740	ST0425	ST425-MSSA	NEG	NEG	NEG	NEG	NEG	POS	POS	NEG	NEG	NEG	NEG	POS	AMB	NEG	NEG	POS	NEG	POS	NEG	NEG	POS	POS
174889	ST0425	ST425-MSSA	POS	POS	NEG	NEG	POS	POS	POS	NEG	NEG	NEG	NEG	POS	AMB	NEG	NEG	POS	NEG	POS	NEG	NEG	POS	POS
203230	ST0425	ST425-MSSA	POS	POS	NEG	NEG	POS	POS	POS	NEG	NEG	NEG	NEG	AMB	POS	NEG	POS	POS	AMB	POS	NEG	AMB	POS	POS
203242	ST0425	ST425-MSSA	POS	POS	NEG	NEG	POS	POS	POS	NEG	NEG	NEG	NEG	POS	AMB	AMB	POS	POS	AMB	POS	AMB	NEG	POS	POS
203243	ST0425	ST425-MSSA	POS	POS	NEG	NEG	POS	POS	POS	NEG	NEG	NEG	NEG	POS	AMB	NEG	POS	POS	AMB	POS	AMB	AMB	POS	POS
207951	ST0425	ST425-MSSA	POS	POS	NEG	NEG	POS	POS	POS	NEG	NEG	NEG	NEG	POS	AMB	NEG	NEG	POS	NEG	POS	NEG	NEG	POS	POS
207981	ST0425	ST425-MSSA	POS	POS	NEG	NEG	POS	POS	POS	NEG	NEG	NEG	NEG	POS	AMB	NEG	POS	POS	NEG	POS	NEG	NEG	POS	POS
195212	ST0425	ST425-MSSA	POS	POS	NEG	NEG	POS	POS	POS	NEG	NEG	NEG	NEG	POS	AMB	NEG	NEG	POS	AMB	POS	NEG	NEG	POS	POS
195206	ST0425	ST425-MSSA	NEG	NEG	NEG	NEG	NEG	POS	POS	NEG	NEG	NEG	NEG	POS	AMB	POS	AMB	POS	AMB	POS	NEG	AMB	POS	POS
195202	ST0425	ST425-MSSA	POS	POS	NEG	NEG	POS	POS	POS	NEG	NEG	NEG	NEG	POS	AMB	NEG	NEG	POS	AMB	POS	NEG	NEG	POS	POS
216042	ST0425	ST425-MSSA	POS	POS	NEG	NEG	POS	POS	POS	NEG	NEG	NEG	NEG	POS	AMB	AMB	POS	POS	AMB	POS	NEG	NEG	POS	POS
223608	ST0425	ST425-MSSA	POS	POS	NEG	NEG	POS	POS	POS	NEG	NEG	NEG	NEG	AMB	POS	NEG	NEG	POS	AMB	POS	AMB	NEG	POS	POS
223612	ST0425	ST425-MSSA	POS	NEG	NEG	NEG	POS	POS	POS	NEG	NEG	NEG	NEG	AMB	POS	NEG	NEG	POS	AMB	POS	AMB	NEG	POS	POS
225733	ST0425	ST425-MSSA	POS	POS	NEG	NEG	POS	POS	POS	NEG	NEG	NEG	NEG	POS	AMB	NEG	POS	POS	AMB	POS	NEG	NEG	POS	POS
230321	ST0425	ST425-MSSA	POS	POS	NEG	NEG	POS	POS	POS	NEG	NEG	NEG	NEG	POS	AMB	NEG	POS	POS	AMB	POS	NEG	NEG	POS	POS
232368	ST0425	ST425-MSSA	POS	POS	NEG	NEG	POS	POS	POS	NEG	NEG	NEG	NEG	POS	AMB	NEG	NEG	POS	AMB	POS	NEG	NEG	POS	POS
204215	ST0425	ST425-MSSA	NEG	NEG	NEG	NEG	NEG	POS	POS	NEG	NEG	NEG	NEG	POS	AMB	NEG	POS	POS	AMB	POS	NEG	NEG	POS	POS
204218	ST0425	ST425-MSSA	NEG	NEG	NEG	NEG	NEG	POS	POS	NEG	NEG	NEG	NEG	POS	AMB	NEG	POS	POS	AMB	POS	NEG	NEG	POS	POS
204222	ST0425	ST425-MSSA	POS	POS	NEG	NEG	POS	POS	POS	NEG	NEG	NEG	NEG	POS	AMB	NEG	POS	POS	AMB	POS	NEG	NEG	POS	POS
204226	ST0425	ST425-MSSA	NEG	NEG	NEG	NEG	NEG	POS	POS	NEG	NEG	NEG	NEG	POS	AMB	NEG	NEG	POS	AMB	POS	NEG	NEG	POS	POS
204906	ST0425	ST425-MSSA	POS	POS	NEG	NEG	POS	POS	POS	NEG	NEG	NEG	NEG	AMB	POS	NEG	POS	POS	AMB	POS	NEG	NEG	POS	POS
204908	ST0425	ST425-MSSA	POS	POS	NEG	NEG	POS	POS	POS	NEG	NEG	NEG	NEG	POS	AMB	AMB	POS	POS	AMB	POS	NEG	AMB	POS	POS
206170	ST0425	ST425-MSSA	POS	POS	NEG	NEG	POS	POS	POS	NEG	NEG	NEG	NEG	POS	AMB	NEG	POS	POS	AMB	POS	NEG	NEG	POS	POS
207765	ST0425	ST425-MSSA	POS	POS	NEG	NEG	POS	POS	POS	NEG	NEG	NEG	NEG	POS	AMB	NEG	POS	POS	NEG	POS	NEG	NEG	POS	POS
240048	CC599	CC599-MRSA-XI	AMB	POS	NEG	POS	NEG	AMB	POS	NEG	NEG	NEG	NEG	POS	AMB	NEG	NEG	POS	NEG	POS	NEG	NEG	POS	POS
225730	CC0692	CC692-MSSA	NEG	NEG	NEG	NEG	NEG	POS	POS	NEG	NEG	NEG	NEG	POS	AMB	POS	AMB	POS	NEG	POS	POS	POS	NEG	NEG
112746	CC0692	CC692-MSSA	NEG	NEG	NEG	NEG	NEG	POS	POS	NEG	NEG	NEG	NEG	POS	AMB	POS	AMB	POS	NEG	POS	POS	POS	NEG	NEG
112754	CC0692	CC692-MSSA	NEG	NEG	NEG	NEG	NEG	POS	POS	NEG	NEG	NEG	NEG	POS	AMB	POS	AMB	POS	NEG	POS	POS	POS	NEG	NEG
112758	CC0692	CC692-MSSA	NEG	NEG	NEG	NEG	NEG	POS	POS	NEG	NEG	NEG	NEG	POS	AMB	POS	AMB	POS	NEG	POS	POS	POS	NEG	NEG
112759	CC0692	CC692-MSSA	NEG	NEG	NEG	NEG	NEG	POS	POS	NEG	NEG	NEG	NEG	POS	AMB	POS	AMB	POS	NEG	POS	POS	POS	NEG	NEG
124745	CC0692	CC692-MSSA	NEG	NEG	NEG	NEG	NEG	POS	POS	NEG	NEG	NEG	NEG	POS	POS	POS	AMB	POS	NEG	POS	POS	POS	NEG	NEG
170952	CC0692	CC692-MSSA	NEG	NEG	NEG	NEG	NEG	POS	POS	NEG	NEG	NEG	NEG	POS	AMB	POS	AMB	POS	NEG	POS	POS	POS	NEG	NEG
170953	CC0692	CC692-MSSA	NEG	NEG	NEG	NEG	NEG	POS	POS	NEG	NEG	NEG	NEG	POS	AMB	POS	AMB	POS	NEG	POS	POS	POS	NEG	NEG
170970	CC0692	CC692-MSSA	NEG	NEG	NEG	NEG	NEG	POS	POS	NEG	NEG	NEG	NEG	POS	AMB	POS	AMB	POS	NEG	POS	POS	POS	NEG	NEG
170950	CC0707	CC707-MSSA	POS	POS	NEG	NEG	POS	POS	POS	NEG	NEG	NEG	NEG	POS	AMB	NEG	NEG	POS	POS	NEG	NEG	NEG	POS	POS
124948	ST0890	ST890-MSSA	POS	POS	POS	NEG	NEG	POS	POS	NEG	NEG	NEG	NEG	POS	AMB	NEG	NEG	POS	NEG	POS	NEG	NEG	POS	POS
220618	CC1956	CC1956-MSSA	NEG	NEG	NEG	NEG	NEG	POS	POS	POS	NEG	NEG	NEG	POS	AMB	AMB	POS	POS	AMB	POS	NEG	NEG	POS	POS
112742	ST2425	ST2425-MSSA	NEG	NEG	NEG	NEG	NEG	POS	POS	POS	NEG	NEG	NEG	POS	POS	POS	AMB	POS	NEG	POS	NEG	NEG	POS	POS
170957	ST2425	ST2425-MSSA	NEG	NEG	NEG	NEG	NEG	POS	POS	POS	NEG	NEG	NEG	POS	AMB	POS	AMB	POS	NEG	POS	NEG	NEG	POS	POS
170947	ST2691	ST2691-MSSA	NEG	NEG	NEG	NEG	NEG	POS	POS	NEG	NEG	NEG	NEG	POS	AMB	POS	AMB	POS	NEG	POS	NEG	NEG	POS	POS
170961	ST2691	ST2691-MSSA	NEG	NEG	NEG	NEG	NEG	POS	POS	NEG	NEG	NEG	NEG	POS	POS	POS	AMB	POS	NEG	POS	NEG	NEG	POS	POS
112753	CC2767	ST2279-MSSA	POS	POS	NEG	NEG	POS	POS	POS	NEG	NEG	NEG	NEG	POS	POS	POS	AMB	POS	NEG	POS	NEG	NEG	POS	POS
112761	CC2767	ST2279-MSSA	POS	POS	NEG	NEG	POS	POS	POS	NEG	NEG	NEG	NEG	POS	AMB	POS	AMB	POS	NEG	POS	NEG	NEG	POS	POS
228651	ST2963	ST2963-MSSA	NEG	NEG	NEG	NEG	NEG	POS	POS	NEG	NEG	NEG	NEG	AMB	POS	POS	AMB	POS	AMB	POS	NEG	NEG	POS	POS
203244	ST3237	ST3237-MSSA	POS	POS	NEG	AMB	NEG	POS	POS	NEG	NEG	NEG	NEG	POS	AMB	POS	AMB	POS	NEG	POS	NEG	NEG	POS	POS

ISOLATE ID	CLONAL COMPLEX (by array)	STRAIN AFFILIATION (by array)	TYPE I RESTRICTION-MODIFICATION SYSTEM, SINGLE SEQUENCE SPECIFICITY PROTEIN													MISCELLANEOUS GENES			HYALURONATE LYASE							
			type I site-specific deoxyribo-nuclease subunit, 1st locus	type I site-specific deoxyribonuclease subunit, 2nd locus				type I site-specific deoxyribonuclease subunit, 3rd locus					type I site-specific deoxyribonuclease subunit, unknown locus			hypothetical protein, located next to serine protease operon	Unspecific efflux/trans-porter	hypothetical protein	hyaluronate lyase, first / second locus			hyaluronate lyase, second locus				
			hsdS1-RF122	hsdS2-ST5+ST8	hsdS2-MW2+476	hsdS2-RF122	hsdS2-MRSA252	hsdS3-AllOtherThanRF122+252	hsdS3-ST8+ST1+RF122	hsdS3-Mu50+N315	hsdS3-CC51+252	hsdS3-MRSA252	hsdSx-CC25	hsdSx-CC15	hsdSx-etd	Q2FXC0	Q2YUB3	Q7A4X2	hysA1 (MRSA252)	hysA1 (MRSA252+RF122) and/or hysA2 (cons)	hysA1 (MRSA252+RF122) and/or hysA2 [illegible]	hysA2 (All Other Than MRSA252)	hysA2 (COL+USA300+NCTC)	hysA2 (All Other Than COL+USA300+NCTC)	hysA2-AllOtherThan COL+USA300+NCTC	hysA2 (MRSA252)
203228	CC0001	CC1-MSSA	NEG	NEG	POS	NEG	NEG	POS	POS	NEG	AMB	NEG	POS	POS	NEG	POS	POS	NEG	NEG	POS	NEG	POS	NEG	POS	POS	POS
203229	CC0001	CC1-MSSA	NEG	NEG	POS	NEG	NEG	POS	POS	NEG	AMB	NEG	POS	POS	NEG	POS	POS	NEG	NEG	POS	NEG	POS	NEG	POS	POS	POS
203233	CC0001	CC1-MSSA	NEG	NEG	POS	NEG	NEG	POS	POS	NEG	NEG	NEG	POS	AMB	NEG	POS	AMB	NEG	NEG	POS	NEG	POS	NEG	POS	POS	POS
220334	CC0001	CC1-MSSA	NEG	NEG	POS	NEG	NEG	POS	POS	NEG	NEG	NEG	POS	NEG	NEG	POS	AMB	NEG	NEG	POS	NEG	POS	NEG	POS	POS	POS
223621	CC0001	CC1-MSSA	NEG	NEG	POS	NEG	NEG	POS	POS	NEG	NEG	NEG	POS	AMB	NEG	POS	POS	NEG	NEG	POS	NEG	POS	NEG	POS	POS	POS
289123	CC0001	CC1-MRSA-IV, WA MRSA-1/57	NEG	NEG	POS	NEG	NEG	POS	POS	NEG	NEG	NEG	POS	NEG	NEG	POS	NEG	NEG	NEG	POS	NEG	POS	NEG	POS	POS	NEG
289124	CC0001	CC1-MRSA-IV, WA MRSA-1/57	NEG	NEG	POS	NEG	NEG	POS	POS	NEG	NEG	NEG	POS	NEG	NEG	POS	NEG	NEG	NEG	POS	NEG	POS	NEG	POS	POS	NEG
275170	CC0005	CC5-MSSA	NEG	POS	NEG	NEG	NEG	POS	NEG	POS	NEG	NEG	POS	NEG	NEG	NEG	NEG	POS	NEG	POS	NEG	AMB	NEG	POS	POS	NEG
275171	CC0005	CC5-MSSA	NEG	POS	NEG	NEG	NEG	POS	NEG	POS	NEG	NEG	POS	NEG	NEG	NEG	NEG	POS	NEG	POS	NEG	NEG	NEG	POS	POS	NEG
112747	CC0005	CC5-MSSA	NEG	POS	NEG	NEG	NEG	POS	NEG	POS	NEG	NEG	POS	NEG	NEG	NEG	NEG	POS	NEG	POS	NEG	POS	NEG	POS	POS	NEG
112752	CC0006	CC6-MSSA	NEG	NEG	AMB	NEG	NEG	POS	POS	NEG	POS	NEG	POS	AMB	NEG	POS	NEG	NEG	NEG	POS	NEG	NEG	NEG	POS	POS	NEG
275178	CC0007	CC7-MSSA	NEG	NEG	NEG	NEG	NEG	NEG	NEG	NEG	NEG	NEG	NEG	NEG	POS	NEG	NEG	POS	NEG	POS	NEG	NEG	NEG	POS	POS	NEG
275166	CC0008	CC8-MSSA	NEG	POS	NEG	NEG	NEG	POS	POS	NEG	NEG	NEG	POS	NEG	NEG	POS	NEG	NEG	NEG	POS	POS	NEG	POS	NEG	NEG	NEG
275176	CC0008	CC8-MSSA	NEG	POS	NEG	NEG	NEG	POS	POS	NEG	NEG	NEG	POS	NEG	NEG	POS	NEG	NEG	NEG	POS	POS	AMB	POS	NEG	NEG	NEG
275177	CC0008	CC8-MSSA	NEG	POS	NEG	NEG	NEG	POS	POS	NEG	NEG	NEG	POS	NEG	NEG	POS	NEG	NEG	NEG	POS	POS	AMB	POS	NEG	NEG	NEG
275167	CC0009	CC9-MSSA	NEG	NEG	POS	NEG	NEG	NEG	NEG	NEG	NEG	NEG	NEG	NEG	NEG	NEG	NEG	POS	NEG	POS	NEG	NEG	NEG	POS	POS	NEG
170968	CC0012	CC12-MSSA	NEG	POS	NEG	NEG	NEG	NEG	NEG	NEG	POS	NEG	POS	NEG	NEG	NEG	NEG	NEG	NEG	POS	NEG	NEG	NEG	POS	POS	NEG
275175	CC0015	CC15-MSSA	NEG	NEG	NEG	NEG	NEG	NEG	NEG	NEG	NEG	NEG	NEG	POS	NEG	NEG	NEG	NEG	NEG	POS	POS	AMB	POS	POS	POS	NEG
170949	CC0015	CC15-MSSA	NEG	NEG	NEG	NEG	NEG	NEG	NEG	NEG	NEG	NEG	NEG	POS	NEG	NEG	NEG	NEG	NEG	POS	POS	POS	POS	POS	POS	NEG
228658	CC0022	CC22-MSSA	NEG	POS	NEG	NEG	NEG	NEG	NEG	NEG	NEG	NEG	POS	NEG	NEG	NEG	AMB	POS	NEG	POS	NEG	NEG	NEG	POS	POS	NEG
289125	CC0022	CC22-MRSA-IV (PVL+)	NEG	POS	NEG	NEG	NEG	AMB	NEG	NEG	NEG	NEG	POS	NEG	NEG	NEG	NEG	POS	NEG	POS	NEG	NEG	NEG	POS	POS	NEG
289126	CC0022	CC22-MRSA-IV (PVL+)	NEG	POS	NEG	NEG	NEG	NEG	NEG	NEG	NEG	NEG	POS	NEG	NEG	NEG	NEG	POS	NEG	POS	NEG	NEG	NEG	POS	POS	NEG
289127	CC0022	CC22-MRSA-IV (PVL+)	NEG	POS	NEG	NEG	NEG	NEG	NEG	NEG	NEG	NEG	POS	NEG	NEG	NEG	NEG	POS	NEG	POS	NEG	NEG	NEG	POS	POS	AMB
131939	CC0025	CC25-MSSA	NEG	NEG	NEG	NEG	NEG	POS	NEG	POS	NEG	NEG	POS	NEG	POS	POS	NEG	POS	NEG	POS	NEG	AMB	NEG	POS	POS	NEG
275165	CC0030	CC30-MSSA (lukF-P83/lukM+)	NEG	NEG	NEG	NEG	POS	NEG	NEG	NEG	POS	POS	POS	NEG	NEG	NEG	NEG	POS	POS	POS	POS	NEG	POS	POS	POS	NEG
275741	CC0030	CC30-MSSA	NEG	NEG	NEG	NEG	POS	NEG	NEG	NEG	POS	POS	POS	NEG	NEG	NEG	NEG	POS	POS	POS	POS	NEG	POS	POS	POS	POS
124492	CC0049	CC49-MSSA (lukF-P83/lukM+)	NEG	NEG	NEG	NEG	NEG	NEG	NEG	NEG	POS	NEG	AMB	POS	POS	NEG	NEG	NEG	POS	AMB	NEG	NEG	NEG	POS	POS	NEG
220616	CC0049	CC49-MSSA (lukF-P83/lukM+)	NEG	NEG	NEG	NEG	NEG	NEG	NEG	NEG	POS	NEG	POS	POS	POS	NEG	AMB	NEG	POS	POS	NEG	NEG	NEG	POS	POS	NEG
228649	CC0049	CC49-MSSA (lukF-P83/lukM+)	NEG	NEG	NEG	NEG	NEG	NEG	NEG	NEG	POS	NEG	POS	POS	POS	NEG	NEG	NEG	POS	POS	NEG	NEG	NEG	POS	POS	NEG
190994	CC0059	CC59-MSSA	NEG	NEG	NEG	NEG	NEG	NEG	NEG	NEG	POS	NEG	NEG	AMB	POS	NEG	NEG	NEG	NEG	NEG	NEG	NEG	NEG	POS	POS	NEG
275172	CC0088	CC88-MSSA	NEG	NEG	NEG	NEG	NEG	NEG	NEG	NEG	NEG	POS	NEG	NEG	NEG	NEG	NEG	NEG	NEG	POS	NEG	NEG	NEG	POS	POS	NEG
275173	CC0088	CC88-MSSA	POS	NEG	NEG	NEG	NEG	POS	NEG	NEG	NEG	POS	NEG	NEG	NEG	NEG	NEG	NEG	NEG	POS	NEG	NEG	NEG	POS	POS	NEG
275174	CC0088	CC88-MSSA	POS	NEG	NEG	NEG	NEG	POS	NEG	NEG	NEG	POS	POS	NEG	NEG	NEG	NEG	NEG	NEG	POS	NEG	POS	NEG	POS	POS	NEG
170971	CC0097	CC97-MSSA	NEG	NEG	NEG	NEG	NEG	NEG	NEG	NEG	NEG	NEG	POS	NEG	NEG	NEG	POS	POS	NEG	POS	NEG	NEG	NEG	POS	POS	NEG
112745	CC0097	CC97-MSSA	NEG	NEG	AMB	NEG	NEG	NEG	NEG	NEG	NEG	NEG	POS	NEG	NEG	NEG	POS	POS	NEG	POS	NEG	NEG	NEG	POS	POS	NEG
112748	CC0097	CC97-MSSA	NEG	NEG	AMB	NEG	NEG	AMB	NEG	NEG	NEG	NEG	POS	NEG	NEG	NEG	POS	POS	NEG	POS	NEG	NEG	NEG	POS	POS	NEG
112750	CC0097	CC97-MSSA	NEG	NEG	AMB	NEG	NEG	NEG	NEG	NEG	NEG	NEG	POS	NEG	NEG	NEG	POS	POS	NEG	POS	NEG	NEG	NEG	POS	POS	NEG
112762	CC0097	CC97-MSSA	NEG	NEG	AMB	NEG	NEG	NEG	NEG	NEG	NEG	NEG	POS	NEG	NEG	NEG	NEG	POS	NEG	POS	NEG	NEG	NEG	POS	POS	NEG
112763	CC0097	CC97-MSSA	NEG	NEG	AMB	NEG	NEG	NEG	NEG	NEG	NEG	NEG	POS	AMB	NEG	NEG	POS	POS	NEG	POS	NEG	NEG	NEG	POS	POS	NEG
112764	CC0097	CC97-MSSA	NEG	NEG	AMB	NEG	NEG	AMB	NEG	NEG	NEG	NEG	POS	NEG	NEG	NEG	POS	POS	NEG	POS	NEG	NEG	NEG	POS	POS	NEG
170945	CC0097	CC97-MSSA	NEG	NEG	NEG	NEG	NEG	NEG	NEG	NEG	NEG	NEG	POS	NEG	NEG	NEG	POS	POS	NEG	POS	NEG	NEG	NEG	POS	POS	NEG
170954	CC0097	CC97-MSSA	NEG	NEG	NEG	NEG	NEG	NEG	NEG	NEG	NEG	NEG	POS	NEG	NEG	NEG	POS	POS	NEG	POS	NEG	NEG	NEG	POS	POS	NEG
170958	CC0097	CC97-MSSA	NEG	NEG	NEG	NEG	NEG	NEG	NEG	NEG	NEG	NEG	POS	NEG	NEG	NEG	POS	POS	NEG	POS	NEG	NEG	NEG	POS	POS	NEG
170963	CC0097	CC97-MSSA	NEG	NEG	NEG	NEG	NEG	NEG	NEG	NEG	NEG	NEG	POS	NEG	NEG	NEG	POS	POS	NEG	POS	NEG	NEG	NEG	POS	POS	NEG
170964	CC0097	CC97-MSSA	NEG	NEG	NEG	NEG	NEG	NEG	NEG	NEG	NEG	NEG	POS	NEG	NEG	NEG	POS	POS	NEG	POS	NEG	NEG	NEG	POS	POS	NEG
170965	CC0097	CC97-MSSA	NEG	NEG	NEG	NEG	NEG	NEG	NEG	NEG	NEG	NEG	POS	NEG	NEG	NEG	POS	POS	NEG	POS	NEG	NEG	NEG	POS	POS	NEG
170966	CC0097	CC97-MSSA	NEG	NEG	NEG	NEG	NEG	NEG	NEG	NEG	NEG	NEG	POS	NEG	NEG	NEG	POS	POS	NEG	POS	NEG	NEG	NEG	POS	POS	NEG
170974	CC0097	CC97-MSSA	NEG	NEG	NEG	NEG	NEG	NEG	NEG	NEG	NEG	NEG	POS	NEG	NEG	NEG	POS	POS	NEG	POS	NEG	NEG	NEG	POS	POS	NEG
170976	CC0097	CC97-MSSA	NEG	NEG	NEG	NEG	NEG	NEG	NEG	NEG	NEG	NEG	POS	NEG	NEG	NEG	POS	POS	NEG	POS	NEG	NEG	NEG	POS	POS	NEG
112744	CC0097	CC97-MSSA	NEG	NEG	NEG	NEG	NEG	NEG	NEG	NEG	NEG	NEG	POS	NEG	NEG	NEG	POS	POS	NEG	POS	NEG	NEG	NEG	POS	POS	NEG
112749	CC0097	CC97-MSSA	NEG	NEG	AMB	NEG	NEG	POS	NEG	NEG	NEG	NEG	POS	NEG	NEG	NEG	POS	POS	NEG	POS	NEG	NEG	NEG	POS	POS	NEG
112757	CC0097	CC97-MSSA	NEG	NEG	AMB	NEG	NEG	AMB	NEG	NEG	NEG	NEG	POS	AMB	NEG	NEG	POS	POS	NEG	POS	NEG	NEG	NEG	POS	POS	NEG
170973	CC0097	CC97-MSSA	NEG	NEG	NEG	NEG	NEG	NEG	NEG	NEG	NEG	NEG	POS	NEG	NEG	NEG	POS	POS	NEG	POS	NEG	NEG	NEG	POS	POS	NEG
207756	CC0097	CC97-MSSA	NEG	NEG	NEG	NEG	NEG	NEG	NEG	NEG	NEG	NEG	POS	AMB	NEG	NEG	POS	POS	NEG	POS	NEG	NEG	NEG	POS	POS	NEG
206171	CC0097	CC97-MSSA	NEG	NEG	NEG	NEG	NEG	NEG	NEG	NEG	NEG	NEG	POS	AMB	NEG	NEG	POS	POS	NEG	POS	NEG	AMB	NEG	POS	POS	NEG
275169	CC0097	CC97-MSSA-[ccrAA]	NEG	NEG	NEG	NEG	NEG	NEG	NEG	NEG	NEG	NEG	POS	NEG	NEG	NEG	NEG	POS	NEG	POS	NEG	NEG	NEG	POS	POS	NEG
275168	CC0097	CC97-MSSA-[ccrAA]	NEG	NEG	NEG	NEG	NEG	NEG	NEG	NEG	NEG	NEG	POS	NEG	NEG	NEG	NEG	POS	NEG	POS	NEG	NEG	NEG	POS	POS	NEG
118320	CC0130	CC130-MSSA (lukF-P83/M+)	NEG	NEG	NEG	POS	NEG	POS	POS	NEG	NEG	NEG	POS	NEG	POS	NEG	NEG	NEG	NEG	POS	NEG	NEG	NEG	POS	POS	NEG
203236	CC0130	CC130-MRSA-XI	NEG	NEG	NEG	POS	NEG	AMB	POS	NEG	NEG	NEG	POS	AMB	POS	NEG	AMB	NEG	NEG	POS	NEG	NEG	NEG	POS	POS	NEG
204204	CC0130	CC130-MRSA-XI	NEG	NEG	NEG	POS	NEG	NEG	POS	NEG	NEG	NEG	POS	NEG	POS	NEG	NEG	NEG	NEG	POS	NEG	NEG	NEG	POS	POS	NEG
289122	CC0130	CC130-MRSA-XI	NEG	NEG	NEG	POS	NEG	AMB	POS	NEG	NEG	NEG	POS	NEG	POS	NEG	NEG	NEG	NEG	POS	NEG	NEG	NEG	POS	POS	NEG
225779	CC0130	CC130-MRSA-XI	NEG	NEG	NEG	POS	NEG	AMB	POS	NEG	NEG	NEG	POS	AMB	POS	NEG	AMB	NEG	NEG	POS	NEG	NEG	NEG	POS	POS	NEG
225780	CC0130	CC130-MRSA-XI	NEG	NEG	NEG	POS	NEG	NEG	POS	NEG	NEG	NEG	POS	NEG	POS	NEG	NEG	NEG	NEG	POS	NEG	NEG	NEG	POS	POS	NEG
170959	CC0130	CC130-MRSA-XI	NEG	NEG	NEG	POS	NEG	NEG	POS	NEG	NEG	NEG	POS	NEG	POS	NEG	NEG	NEG	NEG	POS	NEG	NEG	NEG	POS	POS	NEG
179029	CC0130	CC130-MRSA-XI	NEG	NEG	AMB	POS	NEG	NEG	POS	NEG	NEG	NEG	POS	NEG	POS	NEG	NEG	NEG	NEG	POS	NEG	NEG	NEG	POS	POS	NEG
116254	CC0133	CC133-MSSA	NEG	NEG	AMB	NEG	NEG	NEG	NEG	NEG	POS	NEG	NEG	POS	NEG	NEG	NEG	POS	NEG	POS	NEG	NEG	NEG	POS	POS	NEG
207761	CC0133	CC133-MSSA	NEG	NEG	NEG	NEG	NEG	AMB	NEG	NEG	POS	NEG	AMB	POS	NEG	NEG	NEG	POS	NEG	POS	NEG	NEG	NEG	POS	POS	NEG
213103	CC0133	CC133-MSSA	NEG	NEG	NEG	NEG	NEG	POS	NEG	NEG	POS	NEG	POS	POS	NEG	NEG	POS	POS	NEG	POS	NEG	NEG	NEG	POS	POS	NEG
275736	CC0133	CC133-MSSA	NEG	NEG	NEG	NEG	NEG	NEG	NEG	NEG	POS	NEG	NEG	POS	NEG	NEG	NEG	POS	NEG	POS	NEG	NEG	NEG	POS	POS	NEG
275737	CC0133	CC133-MSSA	NEG	NEG	NEG	NEG	NEG	NEG	NEG	NEG	POS	NEG	NEG	POS	NEG	NEG	NEG	POS	NEG	POS	NEG	NEG	NEG	POS	POS	NEG
275738	CC0133	CC133-MSSA	NEG	NEG	NEG	NEG	NEG	NEG	NEG	NEG	POS	NEG	NEG	POS	NEG	NEG	NEG	POS	NEG	POS	NEG	NEG	NEG	POS	POS	NEG
275739	CC0133	CC133-MSSA	NEG	NEG	NEG	NEG	NEG	NEG	NEG	NEG	POS	NEG	NEG	POS	NEG	NEG	NEG	POS	NEG	POS	NEG	NEG	NEG	POS	POS	NEG
112755	CC0133	CC133-MSSA	NEG	NEG	NEG	NEG	NEG	POS	NEG	NEG	POS	NEG	NEG	POS	NEG	NEG	NEG	POS	NEG	POS	NEG	NEG	NEG	POS	POS	NEG
225778	CC0398	CC398-MRSA-V/VT	NEG	NEG	NEG	NEG	NEG	NEG	NEG	NEG	NEG	NEG	NEG	POS	NEG	NEG	NEG	NEG	NEG	POS	POS	NEG	POS	NEG	NEG	NEG
123159	ST0425	ST425-MSSA	NEG	NEG	NEG	NEG	POS	NEG	NEG	NEG	NEG	NEG	NEG	POS	POS	NEG	NEG	POS	POS	POS	POS	NEG	POS	POS	POS	NEG
86991	ST0425	ST425-MSSA	NEG	NEG	NEG	NEG	POS	NEG	NEG	NEG	NEG	NEG	NEG	POS	POS	NEG	NEG	POS	AMB	POS	POS	NEG	POS	POS	POS	NEG
112756	ST0425	ST425-MSSA	NEG	NEG	AMB	NEG	POS	NEG	NEG	NEG	NEG	NEG	NEG	POS	POS	NEG	NEG	POS	POS	POS	POS	NEG	POS	POS	POS	NEG
195207	ST0425	ST425-MSSA	NEG	NEG	AMB	NEG	POS	NEG	NEG	NEG	NEG	NEG	NEG	POS	POS	NEG	NEG	POS	POS	POS	POS	NEG	POS	POS	POS	NEG
223609	ST0425	ST425-MSSA	NEG	NEG	POS	NEG	POS	AMB	NEG	NEG	AMB	NEG	POS	POS	POS	NEG	POS	POS	POS	POS	POS	NEG	POS	POS	POS	NEG
275740	ST0425	ST425-MSSA	NEG	NEG	NEG	NEG	POS	NEG	NEG	NEG	NEG	NEG	NEG	POS	POS	NEG	NEG	POS	POS	POS	POS	NEG	POS	POS	POS	NEG
174889	ST0425	ST425-MSSA	NEG	NEG	NEG	NEG	POS	NEG	NEG	NEG	NEG	NEG	NEG	POS	POS	NEG	NEG	POS	POS	POS	POS	NEG	POS	POS	POS	NEG
203230	ST0425	ST425-MSSA	NEG	NEG	NEG	NEG	POS	POS	NEG	NEG	NEG	NEG	POS	POS	POS	NEG	POS	POS	POS	POS	POS	NEG	POS	POS	POS	AMB
203242	ST0425	ST425-MSSA	NEG	NEG	AMB	NEG	POS	AMB	NEG	NEG	NEG	NEG	POS	POS	POS	NEG	POS	POS	POS	POS	POS	NEG	POS	POS	POS	NEG
203243	ST0425	ST425-MSSA	NEG	NEG	AMB	NEG	POS	POS	NEG	NEG	NEG	NEG	POS	POS	POS	NEG	POS	POS	POS	POS	POS	NEG	POS	POS	POS	AMB
207951	ST0425	ST425-MSSA	NEG	NEG	NEG	NEG	POS	NEG	NEG	NEG	NEG	NEG	NEG	POS	POS	NEG	NEG	POS	POS	POS	POS	NEG	AMB	POS	POS	NEG
207981	ST0425	ST425-MSSA	NEG	NEG	NEG	NEG	POS	NEG	NEG	NEG	NEG	NEG	NEG	POS	POS	NEG	NEG	POS	POS	POS	POS	NEG	AMB	POS	POS	NEG
195212	ST0425	ST425-MSSA	NEG	NEG	NEG	NEG	POS	NEG	NEG	NEG	NEG	NEG	NEG	POS	POS	NEG	AMB	POS	POS	POS	POS	NEG	POS	POS	POS	NEG
195206	ST0425	ST425-MSSA	NEG	NEG	AMB	NEG	POS	AMB	NEG	NEG	NEG	NEG	POS	POS	POS	NEG	POS	POS	POS	POS	POS	NEG	POS	POS	POS	NEG
195202	ST0425	ST425-MSSA	NEG	NEG	POS	NEG	POS	NEG	NEG	NEG	NEG	NEG	NEG	POS	POS	NEG	POS	POS	POS	POS	POS	NEG	POS	POS	POS	NEG
216042	ST0425	ST425-MSSA	NEG	NEG	NEG	NEG	POS	AMB	NEG	NEG	NEG	NEG	AMB	POS	POS	NEG	POS	POS	POS	POS	POS	AMB	POS	POS	POS	AMB
223608	ST0425	ST425-MSSA	NEG	NEG	NEG	NEG	POS	POS	NEG	NEG	AMB	NEG	AMB	POS	POS	NEG	POS	POS	POS	POS	POS	AMB	POS	POS	POS	AMB
223612	ST0425	ST425-MSSA	NEG	NEG	NEG	NEG	POS	AMB	NEG	NEG	NEG	NEG	NEG	POS	POS	NEG	POS	POS	POS	POS	POS	AMB	POS	POS	POS	AMB
225733	ST0425	ST425-MSSA	NEG	NEG	NEG	NEG	POS	NEG	NEG	NEG	NEG	NEG	NEG	POS	POS	NEG	AMB	POS	POS	POS	POS	NEG	POS	POS	POS	NEG
230321	ST0425	ST425-MSSA	NEG	NEG	POS	NEG	POS	NEG	NEG	NEG	NEG	NEG	NEG	POS	POS	NEG	NEG	POS	POS	POS	POS	NEG	POS	POS	POS	NEG
232368	ST0425	ST425-MSSA	NEG	NEG	AMB	NEG	POS	NEG	NEG	NEG	NEG	NEG	NEG	POS	POS	NEG	NEG	POS	POS	POS	POS	NEG	POS	POS	POS	NEG
204215	ST0425	ST425-MSSA	NEG	NEG	NEG	NEG	POS	NEG	NEG	NEG	NEG	NEG	AMB	POS	POS	NEG	AMB	POS	POS	POS	POS	NEG	POS	POS	POS	NEG
204218	ST0425	ST425-MSSA	NEG	NEG	NEG	NEG	POS	NEG	NEG	NEG	NEG	NEG	AMB	POS	POS	NEG	POS	POS	POS	POS	POS	NEG	POS	POS	POS	NEG
204222	ST0425	ST425-MSSA	NEG	NEG	AMB	NEG	POS	NEG	NEG	NEG	NEG	NEG	AMB	POS	POS	NEG	AMB	POS	POS	POS	POS	NEG	POS	POS	POS	NEG
204226	ST0425	ST425-MSSA	NEG	NEG	AMB	NEG	POS	NEG	NEG	NEG	NEG	NEG	NEG	POS	POS	NEG	NEG	POS	POS	POS	POS	NEG	POS	POS	POS	NEG
204906	ST0425	ST425-MSSA	NEG	NEG	NEG	NEG	POS	NEG	NEG	NEG	NEG	NEG	AMB	POS	POS	NEG	POS	POS	POS	POS	POS	NEG	POS	POS	POS	NEG
204908	ST0425	ST425-MSSA	NEG	NEG	NEG	NEG	POS	AMB	NEG	NEG	NEG	NEG	POS	POS	POS	NEG	POS	POS	POS	POS	POS	NEG	POS	POS	POS	NEG
206170	ST0425	ST425-MSSA	NEG	NEG	NEG	NEG	POS	NEG	NEG	NEG	NEG	NEG	NEG	POS	POS	NEG	AMB	POS	POS	POS	POS	NEG	POS	POS	POS	NEG
207765	ST0425	ST425-MSSA	NEG	NEG	NEG	NEG	POS	NEG	NEG	NEG	NEG	NEG	NEG	POS	POS	NEG	NEG	POS	POS	POS	POS	NEG	POS	POS	POS	NEG
240048	CC599	CC599-MRSA-XI	NEG	NEG	NEG	NEG	NEG	AMB	NEG	NEG	POS	NEG	NEG	POS	NEG	NEG	NEG	NEG	POS	POS	POS	NEG	POS	POS	POS	NEG
225730	CC0692	CC692-MSSA	NEG	NEG	NEG	NEG	NEG	NEG	NEG	NEG	NEG	NEG	POS	AMB	NEG	NEG	POS	POS	POS	POS	POS	NEG	POS	POS	POS	NEG
112746	CC0692	CC692-MSSA	NEG	NEG	AMB	NEG	NEG	NEG	NEG	NEG	NEG	NEG	POS	NEG	NEG	NEG	NEG	POS	POS	POS	POS	NEG	POS	POS	POS	NEG
112754	CC0692	CC692-MSSA	NEG	NEG	POS	NEG	NEG	NEG	NEG	NEG	NEG	NEG	POS	POS	NEG	NEG	AMB	POS	POS	POS	POS	NEG	POS	POS	POS	NEG
112758	CC0692	CC692-MSSA	NEG	NEG	AMB	NEG	NEG	NEG	NEG	NEG	NEG	NEG	POS	AMB	NEG	NEG	AMB	POS	POS	POS	POS	NEG	POS	POS	POS	NEG
112759	CC0692	CC692-MSSA	NEG	NEG	AMB	NEG	NEG	NEG	NEG	NEG	NEG	NEG	POS	NEG	NEG	NEG	NEG	POS	POS	POS	POS	NEG	POS	POS	POS	NEG
124745	CC0692	CC692-MSSA	NEG	NEG	AMB	NEG	NEG	NEG	NEG	NEG	NEG	NEG	POS	NEG	NEG	NEG	NEG	POS	POS	POS	POS	NEG	POS	POS	POS	NEG
170952	CC0692	CC692-MSSA	NEG	NEG	NEG	NEG	NEG	NEG	NEG	NEG	NEG	NEG	POS	NEG	NEG	NEG	AMB	POS	POS	POS	POS	NEG	POS	POS	POS	NEG
170953	CC0692	CC692-MSSA	NEG	NEG	AMB	NEG	NEG	NEG	NEG	NEG	NEG	NEG	POS	NEG	NEG	NEG	AMB	POS	POS	POS	POS	NEG	POS	POS	POS	NEG
170970	CC0692	CC692-MSSA	NEG	NEG	NEG	NEG	NEG	NEG	NEG	NEG	NEG	NEG	POS	NEG	NEG	NEG	NEG	POS	POS	POS	POS	NEG	POS	POS	POS	NEG
170950	CC0707	CC707-MSSA	NEG	AMB	NEG	NEG	NEG	NEG	NEG	NEG	NEG	NEG	POS	NEG	NEG	NEG	NEG	NEG	NEG	POS	NEG	NEG	NEG	POS	POS	NEG
124948	ST0890	ST890-MSSA	NEG	NEG	NEG	NEG	POS	AMB	NEG	NEG	POS	NEG	NEG	POS	POS	NEG	NEG	NEG	NEG	POS	NEG	NEG	NEG	POS	POS	NEG
220618	CC1956	CC1956-MSSA	NEG	NEG	AMB	NEG	NEG	POS	NEG	NEG	POS	NEG	POS	POS	POS	NEG	POS	NEG	POS	POS	POS	NEG	POS	NEG	NEG	NEG
112742	ST2425	ST2425-MSSA	NEG	NEG	NEG	NEG	NEG	NEG	NEG	NEG	NEG	NEG	POS	POS	POS	NEG	NEG	POS	NEG	POS	NEG	NEG	NEG	POS	POS	NEG
170957	ST2425	ST2425-MSSA	NEG	NEG	NEG	NEG	NEG	NEG	NEG	NEG	NEG	NEG	NEG	POS	POS	NEG	NEG	POS	NEG	POS	NEG	NEG	NEG	POS	POS	NEG
170947	ST2691	ST2691-MSSA	NEG	NEG	NEG	NEG	NEG	POS	POS	NEG	NEG	NEG	POS	NEG	NEG	NEG	POS	NEG	NEG	POS	POS	NEG	POS	POS	POS	NEG
170961	ST2691	ST2691-MSSA	NEG	NEG	NEG	NEG	NEG	POS	POS	NEG	NEG	NEG	POS	NEG	NEG	NEG	POS	NEG	NEG	POS	POS	NEG	POS	POS	POS	NEG
112753	CC2767	ST2279-MSSA	NEG	NEG	NEG	NEG	NEG	NEG	NEG	NEG	NEG	NEG	NEG	NEG	NEG	NEG	NEG	NEG	NEG	POS	POS	AMB	POS	NEG	NEG	NEG
112761	CC2767	ST2279-MSSA	NEG	NEG	NEG	NEG	NEG	NEG	NEG	NEG	NEG	NEG	NEG	NEG	NEG	NEG	NEG	NEG	NEG	POS	NEG	NEG	NEG	NEG	NEG	NEG
228651	ST2963	ST2963-MSSA	NEG	NEG	NEG	NEG	NEG	POS	NEG	NEG	NEG	NEG	POS	NEG	NEG	NEG	NEG	POS	POS	POS	POS	NEG	POS	POS	POS	NEG
203244	ST3237	ST3237-MSSA	NEG	NEG	NEG	NEG	NEG	NEG	NEG	NEG	POS	POS	POS	POS	NEG	NEG	POS	NEG	POS	POS	POS	AMB	POS	POS	POS	AMB
